# Exploring the bidirectional relationship between pain and mental disorders: a comprehensive Mendelian randomization study

**DOI:** 10.1186/s10194-023-01612-2

**Published:** 2023-07-07

**Authors:** Chongjie Yao, Yuchen Zhang, Ping Lu, Bin Xiao, Pingping Sun, Jiming Tao, Yanbin Cheng, Lingjun Kong, Dongsheng Xu, Min Fang

**Affiliations:** 1 Shuguang Hospital, Shanghai University of Traditional Chinese Medicine, Shanghai, 201203 People’s Republic of China; 2 School of Acupuncture-Moxibustion and Tuina, Shanghai University of Traditional Chinese Medicine, Shanghai, 201203 People’s Republic of China; 3 School of Rehabilitation Science, Shanghai University of Traditional Chinese Medicine, Shanghai, 201203 People’s Republic of China; 4 Yueyang Hospital of Integrated Traditional Chinese and Western Medicine, Shanghai University of Traditional Chinese Medicine, Shanghai, 200437 People’s Republic of China; 5 Research Institute of Tuina, Shanghai Academy of Traditional Chinese Medicine, Shanghai, 200437 People’s Republic of China

**Keywords:** Pain, Mental disorder, Psychology, Bidirectional relationship, Mendelian randomization

## Abstract

**Background:**

The close relationship between pain and mental health problems is well-known, and psychological intervention can provide an effective alternative to medication-based pain relief. However, previous studies on the connection between pain and psychological problems, the findings thus far have been inconclusive, limiting the potential for translating psychological interventions into clinical practice. To complement the gap, this study utilized genetic data and Mendelian randomization (MR) to examine the potential relationship between pain in different parts and common mental disorders.

**Methods:**

Based on the instrumental variables selected from the Genome-wide association study summary statistics of localized pain and mental disorders, we conducted bidirectional two-sample MR analyses to infer bidirectional causal associations between pain and mental disorders. The inverse-variance weighted MR method and MR-Egger were used as the primary statistical method according to the horizontal pleiotropy and heterogeneity level. We reported the odds ratio to infer the causal effect between pain and mental disorders. *F* statistic was calculated to measure the statistical efficacy of the analyses.

**Results:**

Insomnia is causally related to the genetic susceptibility of multisite pain including head (OR = 1.09, 95% CI: 1.06–1.12), neck/shoulder (OR = 1.12, 95% CI: 1.07–1.16), back (OR = 1.12, 95% CI: 1.07–1.18) and hip (OR = 1.08, 95% CI: 1.05–1.10). Reversely, headache (OR = 1.14, 95% CI: 1.05–1.24), neck/shoulder pain (OR = 1.95, 95% CI: 1.03–3.68), back pain (OR = 1.40, 95% CI: 1.22–1.60), and hip pain (OR = 2.29, 95% CI: 1.18–4.45) promote the genetic liability of insomnia. Depression is strongly associated with the predisposition of multisite pain including headache (OR = 1.28, 95% CI: 1.08–1.52), neck/shoulder pain (OR = 1.32, 95% CI: 1.16–1.50), back pain (OR = 1.35, 95% CI: 1.10–1.66) and stomach/abdominal pain (OR = 1.14, 95% CI: 1.05–1.25), while headache (OR = 1.06, 95% CI: 1.03–1.08), neck/shoulder (OR = 1.09, 95% CI: 1.01–1.17), back (OR = 1.08, 95% CI: 1.03–1.14), and stomach/abdominal pain (OR = 1.19, 95% CI: 1.11–1.26) are predisposing factors for depression. Additionally, insomnia is associated with the predisposition of facial, stomach/abdominal, and knee pain, anxiety was associated with the predisposition of neck/shoulder and back pain, while the susceptibilities of hip and facial pain are influenced by depression, but these associations were unidirectional.

**Conclusions:**

Our results enhance the understanding of the complex interplay between pain and mental health and highlight the importance of a holistic approach to pain management that addresses both physical and psychological factors.

**Supplementary Information:**

The online version contains supplementary material available at 10.1186/s10194-023-01612-2.

## Background

Pain, as defined by the International Association for the Study of Pain (IASP), is an unpleasant subjective and emotional experience associated with tissue damage or potential tissue damage [1]. With economic development and changes in the living environment, the incidence of pain has increased significantly in recent years. Globally, the prevalence of back pain has been reported at 18.1%, headache at 42%, pelvic pain at 2% to 16%, and musculoskeletal pain at 25% [2]. These figures underscore the significant burden of pain on individuals and society, highlighting the need for effective pain management strategies. Regrettably, the existing healthcare services to manage pain are severely insufficient in low- and middle-income countries, where the majority of patients continue to suffer without proper treatment [3]. However, even in developed countries such as the United States, pain remains a significant issue, with as many as 30% of patients misusing opioids and an alarming surge in drug overdose deaths [4].

Sustained pain can result in maladaptive cognition and behaviors, impair daily function, increase psychological stress, and even exacerbate the pain itself [5]. In contrast, individuals who are in a good mental state, despite suffering from chronic pain, have a slight but noticeable association with pain reduction and mitigation of physical dysfunction [6]. Therefore, researchers are increasingly recognizing the intricate comorbidities and interactions between pain and mental disorders, where the two diseases often coexist and promote each other, and may be partially mediated by shared neural mechanisms [7]. Addressing both the physical and psychological aspects of pain is crucial for managing this condition effectively and improving patients' quality of life. It has been reported [8] that there is a strong correlation between the prevalence of depression and the severity of pain in patients with lower back pain. Patients with severe pain experience higher levels of mental distress than those with mild or moderate pain. Moreover, observational studies [9, 10] have also found that the use of opioids is highly comorbid with depression, anxiety, and stress related disorders. These findings underscore the importance of psychological interventions as an integral component of pain management, which can improve patients' mental health, help alleviate pain, and reduce reliance on opioids, and may be a potential alternative to medication analgesia in some cases [11].

Despite previous research on the relationship between pain and psychological problems, the findings have been inconclusive, and our understanding of this complex interaction remains limited. A recent MR study [12] has identified depression as a risk factor for pain localized in specific regions, including the head, neck/shoulder, back, and abdomen/stomach. However, whether there are causal associations between other mental disorders and pain remain uncertain, which may pose challenges for further clinical application of psychological intervention. Addressing this gap in research through rigorous and systematic investigation of the complexities of the relationship between pain and mental health is necessary to develop effective and targeted psychological interventions that can benefit patients in need.

Mendelian randomization (MR) involves the use of single nucleotide polymorphism (SNP) as instrumental variables to estimate the causal effect of exposure factors on outcomes [13]. This method is advantageous because genetic variants are randomly allocated during meiosis, minimizing confounding, measurement error, and reverse causation that can afflict conventional multivariable regression approaches [14]. Moreover, the genetic variations used in MR analyses are unrelated to confounding variables in the exposure-outcome relation, reducing the risk of bias, and enhancing the validity of causal inference [15]. Given the critical public health implications of assessing the bidirectional relationship between pain and mental disorders, obtaining robust causal inferences about these associations is critical for developing effective prevention and treatment strategies. The primary aim of the study is to update and expand upon previous research by conducting a bidirectional MR analysis of self-reported data from the genome-wide association studies (GWAS) of pain and mental disorders conducted in the UK Biobank (UKB). This study is to deepen the understanding of the complex interplay between pain and mental disorders, which can inform the development of more effective interventions for these conditions.

## Methods

### Study design

We conduct the current MR study applying a bidirectional framework where the instrumental variables (IV)-exposure and IV-outcome associations are from 11 genome-wide association studies. First, associated data for exposure variables were obtained from a GWAS database to identify SNPs associated with exposure factors. Then another GWAS database was used to obtain the associated data with the outcome variables, and the existence of related SNPs was confirmed. Finally, qualified SNPs were selected, and a variety of statistical methods were used to comprehensively determine the causal association between exposure factors and the risk of morbidity of outcome variables. Exposure factors and outcome variables in each analysis will be interchanged to determine whether there is a reverse causality relationship between the two.

### Data source for localized pain and mental disorders

Summary—level data of SNPs associated with the localized pain as genetic instruments were taken from eight large-scale GWASs with a total of 461,857 European individuals. Information was collected through a specific pain-related questionnaire about the pain type(s) experienced in last month. The options were: (1) headache; (2) facial pain; (3) neck or shoulder pain; (4) back pain; (5) stomach or abdominal pain; (6) hip pain; (7) knee pain; (8) none of the above. The public databases for above-mentioned GWAS were available from the IEU GWAS database (https://gwas.mrcieu.ac.uk/).

The summary genetic statistics for mental disorders were obtained from the IEU GWAS database (available from the IEU GWAS database: https://gwas.mrcieu.ac.uk/). Three GWASs related to sleeplessness/insomnia, anxiety/panic attacks and depression were selected as mental disorders with a combined total of 1,137,057 European individuals.

The UK Biobank is a cohort study including 500,000 adults, aged 40 to 69 years. In this study, data in UKB were extracted from the first round of genome‐wide association analyses by the Neale Lab and the IEU analysis of UKB phenotypes, where individuals of non‐European ancestry, closely related individuals, individuals with sex chromosome aneuploidies, and individuals who had withdrawn consent were excluded [16]. The specific questions used to define these conditions was shown in supplementary file 1.

### Genetic instruments selections

In this MR study, SNPs that were identified to be associated with exposure factor at the genome-wide significance level (*P* value < 5 × 10^–8^) in the publicly available GWASs and were not in linkage disequilibrium (LD) with other SNPs (r^2^ < 0.01 within a clumping window of 10,000 kb) were used as instruments for these diseases. If a particular exposure SNP is not present in an outcome dataset, proxy SNPs were used instead through LD tagging. Moreover, some MR sensitivity analyses required at least 3 SNPs related to exposure as the genetic instrument, so the selection threshold P value will be adjusted to 5 × 10^–6^ if the number of SNPs available for analysis is less than 3. Finally, a total of 82 mental disorder-associated SNPs and 181 localized pain-related SNPs were included in the MR analysis. A simplified description of the data concerning the SNPs used as instruments in this MR study is listed in the supplementary file 2.

### Statistical analysis

All MR analyses were performed using the MR-Base web app and TwoSampleMR R packages in R software version 4.2.2 [17].

In the analysis, we applied the inverse-variance weighted (IVW) MR method to estimate the associations between localized pain and mental disorders. The heterogeneity among genetic instruments was evaluated by Cochran’s Q test [18]. If heterogeneity existed (*P* value < 0.05), a multiplicative random effect IVW model was used; otherwise, a fixed-effect IVW model was used [19]. The MR-Egger regression was applied to access possible horizontal pleiotropy via the intercept term, and used to estimate the associations between SNPs if pleiotropic effects exist (*P* value < 0.05) [20]. The weight median method was also calculated as supplementary statistical method to verify the stability of the results. Leave-one-out (LOO) analysis was performed to assess if the causal association was dominated by a single SNP that had a large horizontal pleiotropy. To avoid potential weak instrumental bias, the *F* statistic ($F=\frac{{beta}^{2}}{{se}^{2}}$) was used to assess the strength of IV. If *F* > 10, the correlation between IV and exposure is considered to be strong enough that the results of the MR analysis can be protected from weak instrumental bias [21]. As the SNPs used to derive the localized pain and mental disorders instruments were constructed using GWAS of UKB, established methods were applied to calculate the extent to which genetic effect sizes were biased because of participants overlap [22].

All results are presented as odds ratios (ORs) with their 95% confidence intervals (CIs) of outcomes.

## Results

Overall, GWASs of 8 localized pain (including cases reporting no pain) and 3 mental disorders were analyzed in the present MR analysis (Table 1). Detailed information of IVs for each exposure factor was shown in supplementary file 2.

**Table 1:** Descriptions of GWAS data used for analyses

GWAS ID	Traits	Consortium	Number of cases	Number of controls	Sample size	Sex	Population
ukb-a-13	Sleeplessness / insomnia	Neale Lab	NA	NA	336965	Males and Females	European
ukb-a-82	Anxiety/panic attacks	Neale Lab	4611	332548	337159	Males and Females	European
ukb-b-12064	Depression	MRC-IEU	26595	436338	462933	Males and Females	European
ukb-b-12181	Headache	MRC-IEU	93308	368549	461857	Males and Females	European
ukb-b-17107	Facial pain	MRC-IEU	8595	453262	461857	Males and Females	European
ukb-b-18596	Neck or shoulder pain	MRC-IEU	106521	355336	461857	Males and Females	European
ukb-b-9838	Back pain	MRC-IEU	118471	343386	461857	Males and Females	European
ukb-b-11413	Stomach or abdominal pain	MRC-IEU	39646	422211	461857	Males and Females	European
ukb-b-7289	Hip pain	MRC-IEU	52087	409770	461857	Males and Females	European
ukb-b-16254	Knee pain	MRC-IEU	98704	363153	461857	Males and Females	European
ukb-b-9130	None of the above	MRC-IEU	184616	277241	461857	Males and Females	European

*GWAS* genome-wide association studies

### MR results of localized pain on the risk of mental disorders

**Fig. 1:**
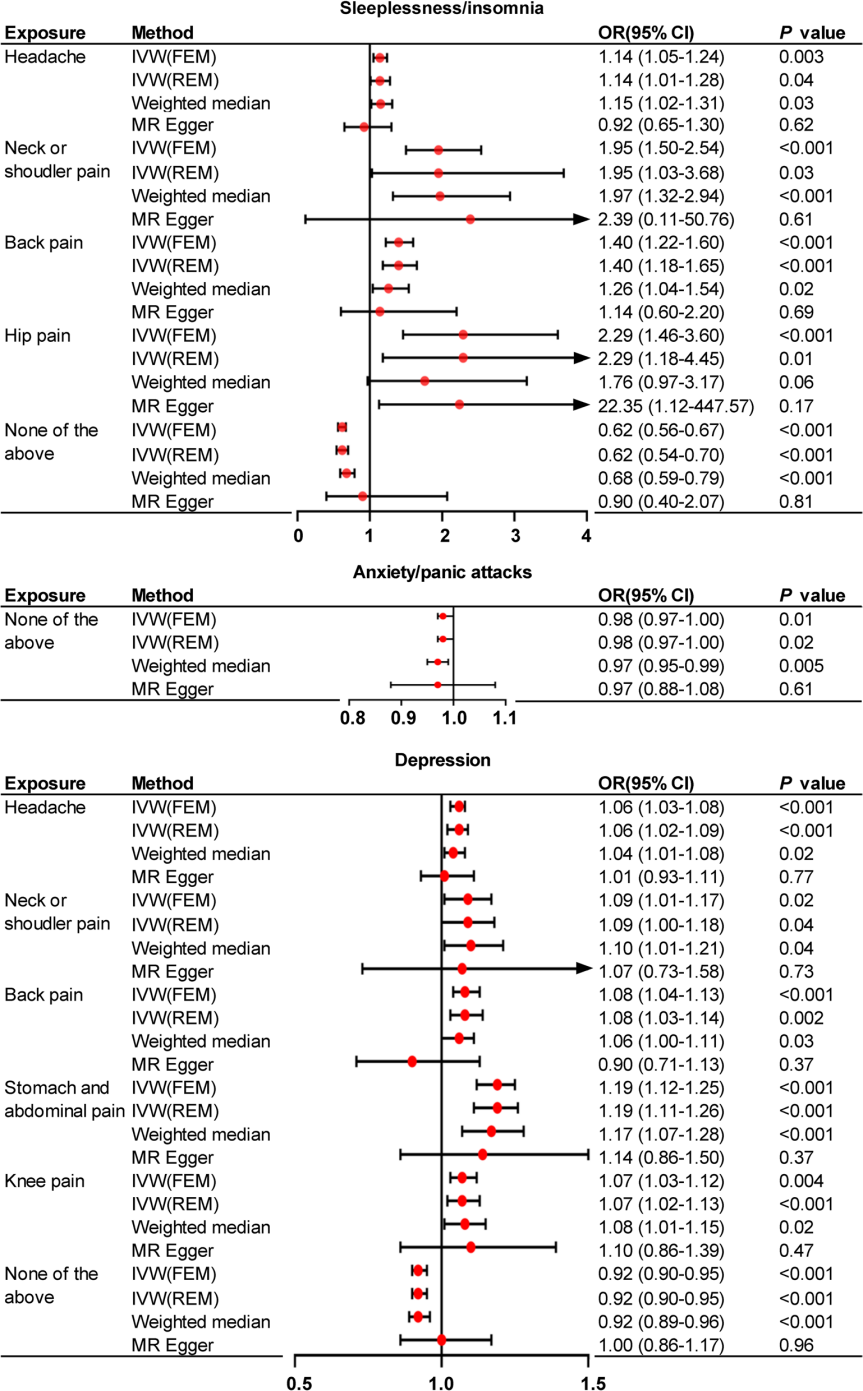
The putative causal effect of localized pain on mental disorders. The results from Mendelian randomization analysis using the IVW approach, weighted median approach or MR-Egger approach (when horizontal pleiotropy exists). Circles and horizontal bars represent the odds ratios and confidence intervals of factor with the risk of pain, respectively. CI, confidence interval; IVW, inverse-variance weighted approach; MR, mendelian randomization; OR, odds ratio

As shown in Fig. 1, the predispositions of several localized pains are significantly associated with the risk of sleeplessness/insomnia and depression. Headaches (OR = 1.14, 95% CI: 1.05–1.24, *P* = 0.04), neck/shoulder pain (OR = 1.95, 95% CI: 1.03–3.68, *P* = 0.03), back pain (OR = 1.40, 95% CI: 1.22–1.60, *P* < 0.001), and hip pain (OR = 2.29, 95% CI: 1.18–4.45, *P* < 0.001) are significant factors predisposing to sleeplessness/insomnia. Headaches (OR = 1.06, 95% CI: 1.03–1.08, *P* < 0.001), neck/shoulder pain (OR = 1.09, 95% CI: 1.01–1.17, *P* = 0.02), back pain (OR = 1.08, 95% CI: 1.03–1.14, *P* = 0.02), stomach/abdominal pain (OR = 1.19, 95% CI: 1.11–1.26, *P* < 0.001), and knee pain (OR = 1.07, 95% CI: 1.02–1.13, *P* = 0.004) are significant contributors to depression.

Conversely, not all localized pain is a predisposing factor for psychological disorders. For example, no localized pain relates to the genetic liability of anxiety/panic attacks, and facial pain does not increase the predisposition of the mental disorders analyzed. In addition, stomach/abdominal pain and knee pain are not associated with insomnia, and hip pain is not significantly associated with depression.

It is worth noting that the absence of any pain is associated with significantly lower genetic susceptibility of sleeplessness/insomnia (OR = 0.62, 95% CI: 0.54–0.70, *P* < 0.001), anxiety/panic attacks (OR = 0.98, 95% CI: 0.97–1.00,* P* = 0.01) and depression (OR = 0.92, 95% CI: 0.90–0.95, *P* < 0.001) (Fig. 1).

Detailed MR results of localized pain on the risk of mental disorders with scatter plots can be found in supplementary file 3.

### MR Results of mental disorders on the risk of localized pain

**Fig. 2:**
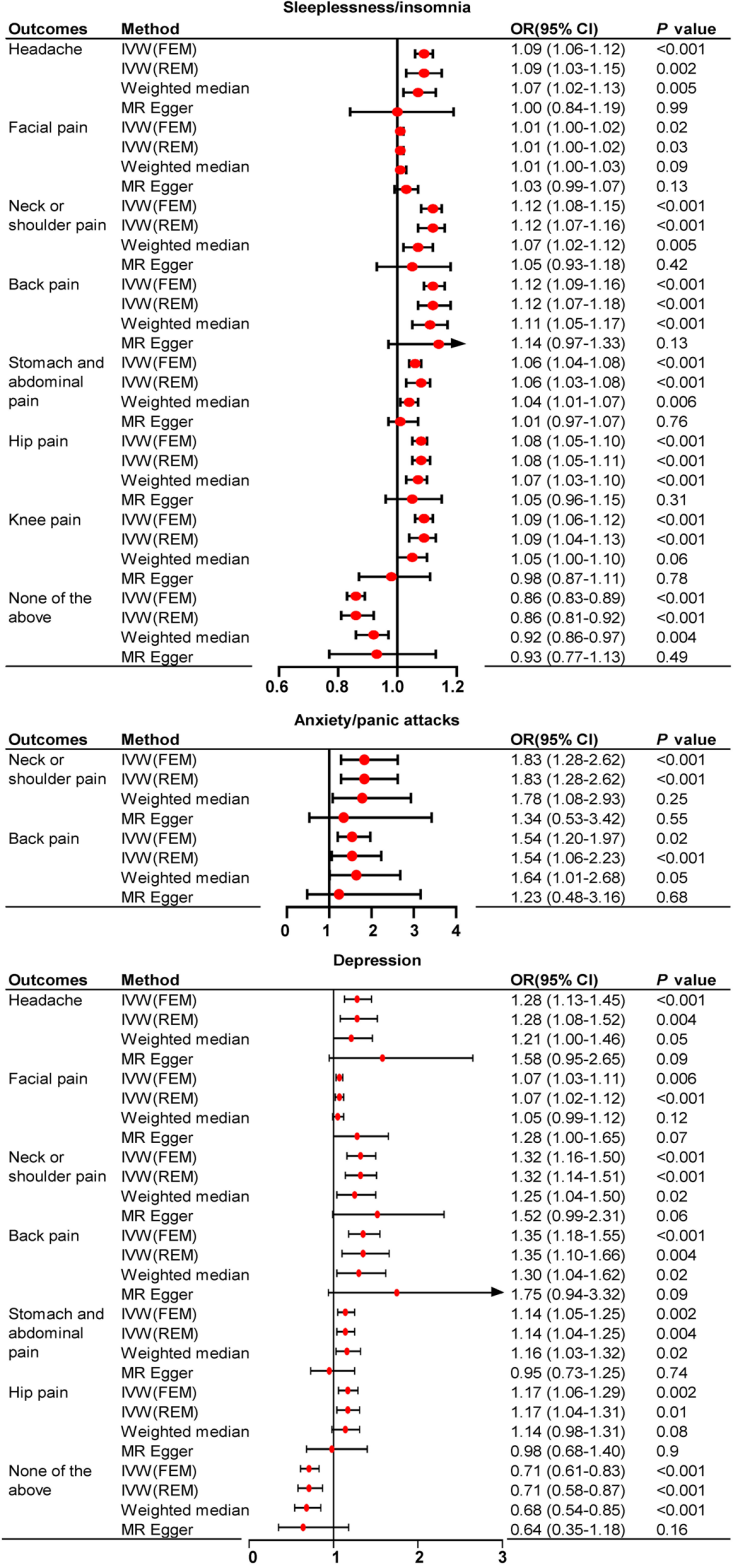
The putative causal effect of mental disorders on localized pain. The results from Mendelian randomization analysis using the IVW approach, weighted median approach or MR-Egger approach (when horizontal pleiotropy exists). Circles and horizontal bars represent the odds ratios and confidence intervals of factor with the risk of pain, respectively. CI, confidence interval; IVW, inverse-variance weighted approach; MR, mendelian randomization; OR, odds ratio

Reverse directional MR revealed a significant causal relationship between mental disorders and several localized pain (Fig. 2). Sleeplessness/insomnia increases the genetic susceptibility of pain in all sites, including headache (OR = 1.09, 95% CI: 1.06–1.12, *P* < 0.001), facial pain (OR = 1.01, 95% CI: 1.00–1.02, *P* = 0.02), neck/shoulder pain (OR = 1.12, 95% CI: 1.07–1.16, *P* < 0.001), back pain (OR = 1.12, 95% CI: 1.07–1.18, *P* < 0.001), stomach/abdominal pain (OR = 1.06, 95% CI: 1.04–1.08, P < 0.001), hip pain (OR = 1.08, 95% CI: 1.05–1.10, *P* < 0.001), and knee pain (OR = 1.09, 95% CI: 1.04–1.13, *P* < 0.001). Anxiety/panic attacks was associated with an increased genetic liability of neck and shoulder pain (OR = 1.83, 95% CI: 1.28–2.62,* P* < 0.001), and back pain (OR = 1.54, 95% CI: 1.06–2.23, *P* = 0.02). Depression was identified as a predisposing contributor for the risk of headache (OR = 1.28, 95% CI: 1.08–1.52, *P* = 0.004), facial pain (OR = 1.07, 95% CI: 1.02–1.12, *P* = 0.006), neck or shoulder pain (OR = 1.32, 95% CI: 1.16–1.50, *P* < 0.001), back pain (OR = 1.35, 95% CI: 1.10–1.66, *P* = 0.004), stomach and abdominal pain (OR = 1.14, 95% CI: 1.05–1.25, *P* = 0.002), and hip pain (OR = 1.17, 95% CI: 1.04–1.31, *P* = 0.01).

In contrast, anxiety is not associated with the genetic susceptibility of pain localized at head, face, stomach and abdomen, hip, and knee, while the onset of knee pain is not associated with depression. Suffering anxiety/panic attacks does not significantly reduce the incidence of being pain-free.

At the same time, not surprisingly, sleeplessness/insomnia (OR = 0.86, 95% CI: 0.81–0.92, *P* < 0.001) and depression (OR = 0.71, 95% CI: 0.58–0.87, *P* < 0.001) significantly reduced the predisposition of being pain-free (Fig. 2).

Detailed MR results of mental disorders on the risk of localized pain with scatter plots can be found in supplementary file 4.

### Sensitivity analyses

The heterogeneity and horizontal pleiotropy among genetic instruments were presented in supplementary file 5. LOO analyses demonstrated that the causal estimates were not driven by any single SNP (supplementary file 6). In addition, the *F* statistics for the IVs of the ranged from 20.85 to 269.39, suggesting that this MR study did not have weak instrument bias. Estimated biases due to sample overlap were small: absolute bias < 0.005; type-1 error rate = 0.05 for all outcomes (supplementary file 7).

## Discussion

The prevalence and cost of pain has become a major physical and mental health care problem worldwide today. With the explosion of research on pain recent years, significant advances have been made in its etiology, assessment, and treatment, many factors related to psychological and psychiatric problems have also been suggested to increase the risk of pain [23]. In this situation, the biopsychosocial model is widely recognized as the most heuristic approach to pain, as it can benefit both pain management and mental disorders due to the overlapping and complementary biological pathways that they share [24]. However, the causal relationship between pain and psychological problems has not been clearly explained due to the limitations of randomized controlled trials (RCTs) in real-world situations. MR provides a groundbreaking way of exploring the cause-and-effect relationships between risk factors and disease outcomes, making it a crucial approach for conducting causal inference in situations where randomized controlled trials (RCTs) are either unfeasible or unethical [25]. Due to this, it is anticipated to play an imperative role in the domain of genetic epidemiology in the future.

In this study, seven forms of localized pain and three types of mental disorders were selected as exposure factors and outcome indicators, respectively. Localized pain in different parts of the body has been shown to have distinct pathogenic effects [12]. Depressive and anxiety disorders are the most prevalent mental health disorders in the general population, while insomnia is closely linked with psychological disorders and can be indicative of one's mental health status [26, 27]. For this MR analysis, IVW and MR-Egger were utilized as the primary statistical methods, and both were fitted using the inverse of the outcome variance as weight. These methods exhibited varied statistical efficacy based on specific SNP conditions. The IVW method, which does not account for intercept terms in regression analysis, demonstrated greater statistical power when horizontal multiplicity of SNPs was not present. Resultantly, it serves as the leading statistical method in this study. In situations where there is horizontal multiplicity of SNPs, IVW analysis results may be inaccurate. To address this, we employed the MR-Egger method, taking into account the intercept term in regression analysis and conducting statistical analysis on SNPs with horizontal multiplicity. The outcome demonstrated a robust and significant bidirectional association between the genetic susceptibility of pain and insomnia, as well as between pain and depression. Headache, neck/shoulder pain, back pain, hip pain, and insomnia exhibited mutually predisposing relationships. Additionally, headache, neck/shoulder pain, back pain, and stomach/abdominal pain are bidirectional predisposing contributors with depression. Furthermore, insomnia was cited as a contributing factor for the genetic susceptibility of facial pain, stomach/abdominal pain, and knee pain. Meanwhile, depression was revealed to be a risk factor for facial pain and hip pain, yet the association was unidirectional. The association between anxiety and localized pain was relatively weak. Our study only confirmed that anxiety could increase the predisposition of neck/shoulder pain and back pain. Furthermore, the absence of pain appeared to mildly reduce the predisposition of depression.

Our findings are in line with a previous report [12] that has identified a causal connection between depression and pain at specific body sites such as head, neck/shoulder, back, and abdominal/stomach. However, our findings suggest a causal relationship between more localized pain and depression. For instance, knee pain is a high-risk factor for developing depression, and depression may contribute to the development of facial pain and hip pain. On a statistical level, the possible reason is that the two studies chose different statistical methods for data analysis. Regarding data sources, the depression GWAS used in this study was from UKB, while the other also incorporated 23andMe and Psychiatric Genomics Consortium, two US databases, as GWAS sources. It has been proposed that even the same ethnic group may have different prevalence of emotional disorders under the influence of different social environments [28]. Therefore, data sources from different regions may lead to different analysis results. Similarly, this study reached an alike conclusion to a previous MR analysis [29] that investigated pain and neuropsychiatric disorders, that multisite pain was associated with the occurrence of insomnia. But our analysis also found an association between knee pain and genetic susceptibility to insomnia after applying a more stringent *P* value threshold to select candidate IVs. Additionally, we selected more detailed multisite pain as exposure and outcome factors in the MR analysis, further complementing previous findings [30, 31] of the bidirectional association between insomnia, depression, and pain. The present study indicated that localized pain in a single or few sites, besides widespread pain, may also be mutual predisposing factors with insomnia and depression.

In general, the present MR study provides evidence to support a putative causal relationship between pain at different sites and typical mental disorders. The main etiological hypothesis is that these 2 disorders are linked via common underlying neurobiological mechanisms. As the highest-level center for sensory signal transmission, the cerebrum is the neurobiological basis for the comorbidity of pain and mental problems. At the tissue level, studies [32–34] have shown that pain and mental status are closely related due to the fact that pain and feelings such as anxiety and depression share the same brain regions, such as the prefrontal cortex, anterior cingulate, anterior insula, amygdala, as well as hippocampus. When long-term chronic pain stimulates biological individuals, certain physiological and structural changes occur in emotional related brain regions, such as the orbitofrontal lobe, prefrontal cortex, and insular lobe, which are key central regions that can simultaneously participate in chronic pain, negative emotions, and cognitive functions. Mental problems, in turn, may also increase pain perception by altering brain structures such as the hippocampal and hypothalamus [35]. At the molecular level, pain and psychological problems both occur through the sharing of abnormalities in multiple neurotransmitters, such as serotonin, substance P, dopamine, norepinephrine, γ- aminobutyric acid, and brain-derived neurotrophic factor [36–38]. Moreover, injury induced neuroinflammatory changes also play an important role in the pathophysiology of pain and psychological dysregulation, and an increasing number of studies [38, 39] have focused on the roles of proinflammatory cytokines in physical and mental comorbidities. Our study confirms a bidirectional causal relationship between headache, neck and shoulder pain, back pain, and various mental disorders, including sleeplessness/insomnia and depression, by a mechanism that may be related to the above studies. Similar relationships also exist between stomach / abdominal pain and depression, hip pain and insomnia. However, not all types of localized pain have a vicious cycle with mental disorders. For example, some previous studies [40, 41] have found a causal relationship between headaches and anxiety or that headaches can aggravate psychological disorders in patients with anxiety, but these conclusions were not further confirmed by the current study. Therefore, whether there are differences in neurobiological mechanisms between different sites of pain remains to be further investigated. Overall, our findings support the positive significance of psychotherapy in alleviating pain and advocate for creating greater self-efficacy and empowerment in patients [42].

Our study has several advantages. Firstly, we used MR analysis to investigate the association between localized pain and three mental disorders comprehensively. Instead of focusing on a specific emotional disorder, we explored the correlation between pain and mental disorders broadly. Additionally, we included pain-free as an exposure factor and outcome indicator, which helped us confirm that there is a negative correlation between pain-free and psychological disorders. Finally, we applied a strict *P* value threshold to screen SNPs, which ensured that the SNPs used in the analysis have a good correlation with the exposure factors, and thus have a great test efficiency.

Nevertheless, it is essential to acknowledge the limitations of our study. Firstly, the GWAS used in this MR analysis were obtained from the UKB. In this program, the determination of whether each participant was a positive case was based on volunteers' self-reports through questionnaires. This method may have its biases as people who lack medical knowledge might not be able to identify the specific site of pain accurately or may mistake short-lived mood swings for mental disorders. In addition, there is a risk of bias in the pain data set, and the psychological disorder data set itself. On the one hand, the questionnaire used to gather data for the GWAS concerning localized pain had some ambiguity, as participants were asked to identify the location of pain experienced in the past month. However, this question may be interpreted as either transient pain in the last month or chronic pain that persisted into the last month, which have different pathophysiological mechanisms and may lead to different effects on mental disorders [43]. Also, each participant may have multiple sites of pain simultaneously, suggesting that the effects of each localized pain in the statistical analysis were not completely independent. A previous study [44] had suggested that the accumulation of pain sites may also be an important factor contributing to functional problems. This interference between exposure factors was not corrected in the current study. On the other hand, the data sets used in the study in the category of psychological disorders all divided positive and control cases by dichotomous variables and failed to assess the severity of psychological disorders, which means that we could not assess the effect of pain on people already who have psychological disorders and vice versa. The participants in the original experiments may have been receiving anti-psychotic or analgesic treatment at the same time as the interview, which, as inferred from the results of this MR analysis, may have masked the pain problems of those with psychological disorders and the psychological disorders of those troubled by pain. It is also worth noting that multiple testing correction was not used to correct *P* value threshold in this study, since it may seriously impair the test efficacy of multifactor-to-multifactor MR analysis. Therefore, the probability of false positives in the analysis results increased. Finally, the participants in this study were all UK residents, so the generalizability of the results of this experiment is limited to populations of European or Western European ancestry.

## Conclusions

Our two-sample Mendelian randomization (MR) analyses revealed a bidirectional relationship between insomnia and pain localized in the head, neck/shoulder, back, and hip regions. Additionally, we found a significant predisposing relationship between depression and headache, neck/shoulder pain, back pain, and stomach/abdominal pain. However, we did not find any evidence that localized pain may increase the genetic susceptibility of anxiety but confirmed that anxiety is a unidirectional high-risk factor for neck/shoulder pain and back pain. Additionally, the study has identified a bidirectional predisposing relationship between the absence of pain and maintaining good mental health. The results further support the existence of causal relationships between localized pain and mental disorders. These findings have significant implications for improving our understanding of the complex comorbidities between pain and mental disorders and highlight the importance of a comprehensive and integrated approach to pain management.

## Supplementary Information

**Additional file 1: Supplementary file 1.** Definition of the exposure factors.

**Additional file 2: Supplementary file 2.** Descriptions of instrument variables in the MR analysis.

**Additional file 3: Supplementary file 3.** MR results of localized pain on risk of sleeplessness/insomnia, anxiety/panic attacks and depression with scatter plots.

**Additional file 4: Supplementary file 4. **MR Results of sleeplessness/insomnia, anxiety/panic attacks and depression on the risk of localized pain with scatter plots.

**Additional file 5: Supplementary file 5.** The heterogeneity and horizontal pleiotropy among genetic instruments.

**Additional file 6: Supplementary file 6.** Results of Leave-one-out analysis.

**Additional file 7: Supplementary file 7. **Estimated biases due to sample overlap.

## Data Availability

The GWAS summary statistics can be obtained from https://openwin.ox.ac.uk/ukbiobank/big40 and https://gwas.mrcieu.ac.uk/. The extracted data used for the analyses are included in this published article and its supplementary information files.

## Abbreviations

CIs: Confidence intervals
GWAS: Genome-wide association study
MR: Mendelian randomization
IASP: International Association for the Study of Pain
IV: Instrumental variables
IVW: Inverse-variance weighted
LD: Linkage disequilibrium
LOO: Leave-one-out
OR: Odds ratio
RCT: Randomized controlled trial
SNP: Single nucleotide polymorphism
UKB: UK Biobank
