# Supplementary material for: Exploring the bidirectional relationship between pain and mental disorders: a comprehensive Mendelian randomization study
Source: J Headache Pain. 2023 Jul 7;24(1):82. doi: 10.1186/s10194-023-01612-2 (PMC10326936; doi:10.1186/s10194-023-01612-2)
Supplement: Supplementary file 1 — Additional file 1: Supplementary file 1. Definition of the exposure factors. [file 10194_2023_1612_MOESM1_ESM.docx]

**Supplementary file 1**

Definition of the exposure factors

| **Traits** | **Definition** |
| --- | --- |
| Sleeplessness/insomnia | Defined by question "Do you have trouble falling asleep at night or do you wake up in the middle of the night?"  If the participant activated the Help button they were shown the message: If this varies a lot, answer this question in relation to the last 4 weeks. |
| Anxiety/panic attacks | Self-reported illness. If the participant was uncertain of the type of illness they had had, then they described it to the interviewer (a trained nurse) who attempted to place it within the coding tree. If the illness could not be located in the coding tree then the interviewer entered a free-text description of it. These free-text descriptions were subsequently examined by a doctor and, where possible, matched to entries in the coding tree. |
| Depression |  |
| Headache | Defined by question "In the last month have you experienced any of the following that interfered with your usual activities? (You can select more than one answer)"  If “None of the above” was selected, then no additional choices were allowed. |
| Facial pain |  |
| Neck and shoulder pain |  |
| Back pain |  |
| Stomach and abdominal pain |  |
| Hip pain |  |
| Knee pain |  |
| None of the above |  |
