# Supplementary material for: Exploring the bidirectional relationship between pain and mental disorders: a comprehensive Mendelian randomization study
Source: J Headache Pain. 2023 Jul 7;24(1):82. doi: 10.1186/s10194-023-01612-2 (PMC10326936; doi:10.1186/s10194-023-01612-2)
Supplement: Supplementary file 2 — Additional file 2: Supplementary file 2. Descriptions of instrument variables in the MR analysis. [file 10194_2023_1612_MOESM2_ESM.docx]

**Supplementary file 2**

Descriptions of instrument variables in the MR analysis

**Characteristics of the 30 sleeplessness / insomnia associated instruments**

| SNP | A1 | A2 | EAF | Beta | SE | *P*-value | *F*-statistic |
| --- | --- | --- | --- | --- | --- | --- | --- |
| rs6690017 | G | T | 0.408391 | -0.00985 | 0.001771 | 2.66E-08 | 30.94 |
| rs11804386 | A | G | 0.333176 | 0.010171 | 0.001846 | 3.59E-08 | 30.36 |
| rs2644128 | G | C | 0.5511 | 0.010901 | 0.001751 | 4.82E-10 | 38.75 |
| rs7572387 | C | A | 0.429282 | 0.01093 | 0.001767 | 6.21E-10 | 38.26 |
| rs2863957 | A | C | 0.219489 | -0.01299 | 0.002104 | 6.67E-10 | 38.12 |
| rs113851554 | T | G | 0.056659 | 0.047777 | 0.00389 | 1.16E-34 | 150.83 |
| rs6744461 | C | A | 0.680005 | 0.010883 | 0.001883 | 7.48E-09 | 33.41 |
| rs4688760 | T | C | 0.691885 | 0.011506 | 0.001886 | 1.06E-09 | 37.21 |
| rs9878792 | T | G | 0.037493 | -0.0267 | 0.004658 | 9.95E-09 | 32.85 |
| rs9815484 | G | A | 0.817741 | 0.013168 | 0.00226 | 5.64E-09 | 33.96 |
| rs2132083 | C | T | 0.660134 | -0.01012 | 0.001844 | 4.04E-08 | 30.13 |
| rs1592757 | C | G | 0.355901 | 0.010353 | 0.001818 | 1.24E-08 | 32.43 |
| rs13186678 | T | C | 0.307241 | 0.011048 | 0.001888 | 4.91E-09 | 34.23 |
| rs3817576 | G | A | 0.525328 | -0.01038 | 0.001747 | 2.81E-09 | 35.32 |
| rs10280045 | G | C | 0.572428 | 0.009726 | 0.001772 | 4.04E-08 | 30.13 |
| rs10087341 | C | T | 0.158749 | 0.014712 | 0.002392 | 7.74E-10 | 37.83 |
| rs10156602 | G | A | 0.363532 | -0.01019 | 0.001823 | 2.32E-08 | 31.21 |
| rs224071 | A | G | 0.550307 | 0.009726 | 0.001749 | 2.67E-08 | 30.94 |
| rs3808937 | T | C | 0.207802 | -0.01431 | 0.002146 | 2.59E-11 | 44.47 |
| rs17879819 | T | C | 0.076456 | -0.01874 | 0.003269 | 9.83E-09 | 32.88 |
| rs2956278 | G | A | 0.214265 | 0.01191 | 0.002123 | 2.03E-08 | 31.37 |
| rs324017 | C | A | 0.70472 | -0.01172 | 0.001912 | 8.83E-10 | 37.57 |
| rs4886140 | G | A | 0.667148 | 0.011077 | 0.001862 | 2.69E-09 | 35.39 |
| rs4943439 | T | C | 0.386641 | 0.010451 | 0.001785 | 4.75E-09 | 34.29 |
| rs6561715 | A | T | 0.628887 | -0.01228 | 0.001807 | 1.06E-11 | 46.21 |
| rs1547630 | A | G | 0.651732 | 0.010442 | 0.001833 | 1.23E-08 | 32.43 |
| rs11635495 | C | T | 0.513921 | 0.009731 | 0.001742 | 2.34E-08 | 31.19 |
| rs1925501 | A | T | 0.220428 | -0.01191 | 0.002106 | 1.54E-08 | 32.00 |
| rs71373536 | A | G | 0.253453 | 0.01345 | 0.002004 | 1.92E-11 | 45.05 |
| rs11152363 | A | G | 0.184703 | 0.014311 | 0.002263 | 2.54E-10 | 40.00 |

SNP: single nucleotide polymorphism; A1: effect allele; A2: other allele; EAF: effect allele frequency.

**Characteristics of the 39 depression associated instruments**

| SNP | A1 | A2 | EAF | Beta | SE | *P*-value | *F*-statistic |
| --- | --- | --- | --- | --- | --- | --- | --- |
| rs114990209 | A | G | 0.020381 | -0.00793 | 0.001719 | 4.00E-06 | 21.28 |
| rs7514305 | T | G | 0.631049 | -0.00232 | 0.000505 | 4.60E-06 | 21.01 |
| rs12034885 | T | A | 0.49715 | -0.00254 | 0.000484 | 1.40E-07 | 27.68 |
| rs2356121 | A | G | 0.439999 | -0.00228 | 0.000486 | 2.80E-06 | 21.94 |
| rs4405712 | G | A | 0.2673 | 0.002544 | 0.000552 | 4.10E-06 | 21.24 |
| rs78943553 | A | C | 0.074505 | -0.00462 | 0.000919 | 5.10E-07 | 25.24 |
| rs6761052 | C | G | 0.197101 | -0.00283 | 0.000607 | 3.00E-06 | 21.80 |
| rs1430674 | G | A | 0.851012 | -0.00322 | 0.000679 | 2.10E-06 | 22.50 |
| rs12473198 | G | A | 0.491207 | -0.00235 | 0.000484 | 1.20E-06 | 23.54 |
| rs12487736 | T | C | 0.574571 | -0.00228 | 0.000488 | 3.10E-06 | 21.73 |
| rs73064834 | G | A | 0.037832 | 0.005883 | 0.001276 | 4.00E-06 | 21.24 |
| rs12507345 | G | A | 0.509235 | 0.002344 | 0.000483 | 1.20E-06 | 23.59 |
| rs10942927 | T | A | 0.367495 | 0.002601 | 0.000502 | 2.20E-07 | 26.86 |
| rs58457416 | A | G | 0.140221 | 0.003232 | 0.000704 | 4.50E-06 | 21.06 |
| rs12153097 | T | G | 0.173619 | 0.003096 | 0.000638 | 1.20E-06 | 23.55 |
| rs115214645 | T | C | 0.021757 | -0.00779 | 0.001668 | 3.00E-06 | 21.81 |
| rs853676 | T | C | 0.163359 | -0.00321 | 0.000653 | 9.00E-07 | 24.14 |
| rs4947339 | T | C | 0.374816 | 0.002246 | 0.00048 | 2.90E-06 | 21.90 |
| rs9398376 | T | C | 0.194638 | -0.00316 | 0.00061 | 2.30E-07 | 26.76 |
| rs13192131 | A | G | 0.019235 | -0.00863 | 0.001763 | 9.80E-07 | 23.96 |
| rs10225041 | C | T | 0.378437 | -0.00244 | 0.000498 | 9.30E-07 | 24.08 |
| rs13229801 | G | A | 0.630736 | -0.00262 | 0.000502 | 1.80E-07 | 27.19 |
| rs3807866 | A | G | 0.410759 | 0.002917 | 0.00049 | 2.70E-09 | 35.38 |
| rs361457 | A | C | 0.3938 | -0.00228 | 0.000497 | 4.40E-06 | 21.08 |
| rs4736824 | T | C | 0.551344 | -0.00243 | 0.000486 | 6.00E-07 | 24.92 |
| rs2054064 | T | A | 0.143524 | -0.0032 | 0.000695 | 4.20E-06 | 21.16 |
| rs75530143 | A | G | 0.019522 | 0.008277 | 0.001788 | 3.70E-06 | 21.43 |
| rs7045289 | G | A | 0.283887 | 0.002558 | 0.000537 | 1.90E-06 | 22.69 |
| rs10897304 | G | A | 0.328459 | -0.00247 | 0.000516 | 1.60E-06 | 22.99 |
| rs77216882 | A | G | 0.039871 | -0.00568 | 0.001235 | 4.30E-06 | 21.13 |
| rs73173375 | T | A | 0.083736 | 0.005252 | 0.001025 | 3.00E-07 | 26.24 |
| rs144560187 | T | C | 0.027194 | 0.007187 | 0.001555 | 3.80E-06 | 21.37 |
| rs7186155 | G | A | 0.553543 | 0.002248 | 0.000486 | 3.80E-06 | 21.37 |
| rs72769061 | C | T | 0.023799 | -0.00755 | 0.0016 | 2.40E-06 | 22.26 |
| rs1125313 | C | A | 0.499867 | 0.002379 | 0.000485 | 9.20E-07 | 24.08 |
| rs60383544 | A | G | 0.053709 | -0.00496 | 0.001072 | 3.80E-06 | 21.38 |
| rs8111479 | A | G | 0.799572 | 0.002759 | 0.000604 | 4.90E-06 | 20.86 |
| rs6041529 | C | T | 0.105693 | 0.003625 | 0.000788 | 4.30E-06 | 21.15 |
| rs4434089 | A | G | 0.293972 | -0.00251 | 0.00053 | 2.30E-06 | 22.33 |

SNP: single nucleotide polymorphism; A1: effect allele; A2: other allele; EAF: effect allele frequency.

**Characteristics of the 12 anxiety/panic attacks associated instruments**

| SNP | A1 | A2 | EAF | Beta | SE | *P*-value | *F*-statistic |
| --- | --- | --- | --- | --- | --- | --- | --- |
| rs6782224 | T | C | 0.034308 | 0.003837 | 0.000781 | 8.89E-07 | 24.16 |
| rs75391409 | T | C | 0.014156 | 0.005972 | 0.00127 | 2.58E-06 | 22.11 |
| rs6892146 | G | C | 0.149216 | 0.001817 | 0.000397 | 4.76E-06 | 20.93 |
| rs7735764 | C | T | 0.548175 | 0.001558 | 0.000288 | 5.98E-08 | 29.37 |
| rs2896248 | T | C | 0.021674 | 0.004726 | 0.000974 | 1.22E-06 | 23.54 |
| rs17790824 | G | A | 0.06397 | 0.002668 | 0.000578 | 3.88E-06 | 21.32 |
| rs74514677 | A | C | 0.061642 | 0.002852 | 0.000613 | 3.31E-06 | 21.63 |
| rs4945276 | G | T | 0.337357 | -0.00144 | 0.0003 | 1.53E-06 | 23.11 |
| rs4910383 | T | C | 0.037714 | 0.003559 | 0.000756 | 2.46E-06 | 22.19 |
| rs9509122 | C | T | 0.391128 | 0.001347 | 0.000292 | 3.98E-06 | 21.28 |
| rs4517635 | A | G | 0.193934 | 0.002086 | 0.000358 | 5.86E-09 | 33.88 |
| rs2831780 | T | C | 0.671206 | 0.001416 | 0.000301 | 2.63E-06 | 22.07 |

SNP: single nucleotide polymorphism; A1: effect allele; A2: other allele; EAF: effect allele frequency.

**Characteristics of the 48 headache associated instruments**

| SNP | A1 | A2 | EAF | Beta | SE | *P*-value | *F*-statistic |
| --- | --- | --- | --- | --- | --- | --- | --- |
| rs4648469 | G | A | 0.781563 | -0.00553 | 0.001009 | 4.20E-08 | 30.04 |
| rs12058508 | A | G | 0.263807 | 0.006366 | 0.000952 | 2.30E-11 | 44.71 |
| rs56304645 | T | C | 0.226394 | 0.007657 | 0.000994 | 1.30E-14 | 59.31 |
| rs10927726 | C | T | 0.370592 | 0.004941 | 0.000864 | 1.10E-08 | 32.73 |
| rs12038786 | T | C | 0.366322 | 0.005078 | 0.000861 | 3.60E-09 | 34.81 |
| rs12134493 | A | C | 0.118634 | 0.012144 | 0.001293 | 5.70E-21 | 88.27 |
| rs1050316 | T | G | 0.652618 | -0.00714 | 0.000874 | 2.90E-16 | 66.88 |
| rs4926395 | C | T | 0.612523 | -0.00545 | 0.000851 | 1.50E-10 | 41.01 |
| rs2297829 | A | C | 0.359269 | -0.0048 | 0.000872 | 3.70E-08 | 30.31 |
| rs715 | C | T | 0.311466 | 0.005176 | 0.0009 | 8.90E-09 | 33.06 |
| rs72938315 | G | C | 0.145116 | -0.00673 | 0.001177 | 1.10E-08 | 32.67 |
| rs1003540 | G | A | 0.194849 | -0.01067 | 0.001048 | 2.40E-24 | 103.67 |
| rs34097149 | C | T | 0.02397 | -0.01886 | 0.002768 | 9.40E-12 | 46.44 |
| rs4075749 | G | A | 0.314872 | 0.004932 | 0.000893 | 3.40E-08 | 30.49 |
| rs7610856 | A | C | 0.429055 | -0.00519 | 0.000843 | 7.20E-10 | 37.96 |
| rs7684253 | T | C | 0.551051 | 0.005307 | 0.000839 | 2.50E-10 | 40.02 |
| rs342459 | A | G | 0.391841 | 0.005363 | 0.000852 | 3.00E-10 | 39.66 |
| rs17433120 | A | T | 0.17643 | 0.0061 | 0.001091 | 2.30E-08 | 31.25 |
| rs9349379 | G | A | 0.405516 | -0.00839 | 0.000846 | 3.40E-23 | 98.42 |
| rs34555420 | T | G | 0.097836 | -0.00786 | 0.001398 | 1.90E-08 | 31.61 |
| rs9486715 | C | A | 0.321733 | 0.012458 | 0.00089 | 1.40E-44 | 196.15 |
| rs927985 | A | G | 0.848794 | 0.006752 | 0.001163 | 6.40E-09 | 33.72 |
| rs3131051 | A | G | 0.421059 | -0.00645 | 0.000841 | 1.60E-14 | 58.96 |
| rs7757975 | T | G | 0.155875 | 0.009427 | 0.001144 | 1.80E-16 | 67.85 |
| rs12532479 | C | T | 0.104973 | 0.009738 | 0.001358 | 7.60E-13 | 51.38 |
| rs10267593 | A | G | 0.170818 | -0.00621 | 0.001106 | 2.00E-08 | 31.52 |
| rs2481601 | G | T | 0.560994 | -0.00528 | 0.000836 | 2.70E-10 | 39.85 |
| rs7858153 | A | G | 0.230988 | 0.007236 | 0.000986 | 2.10E-13 | 53.89 |
| rs1444802 | C | T | 0.079898 | -0.01066 | 0.001535 | 3.70E-12 | 48.26 |
| rs78438709 | G | A | 0.070048 | -0.01233 | 0.001631 | 4.10E-14 | 57.13 |
| rs4909945 | C | T | 0.689955 | 0.005735 | 0.000898 | 1.70E-10 | 40.84 |
| rs3758919 | A | G | 0.090627 | 0.00801 | 0.001446 | 3.00E-08 | 30.69 |
| rs57295447 | A | G | 0.270319 | -0.00523 | 0.000935 | 2.20E-08 | 31.33 |
| rs11172113 | C | T | 0.411988 | -0.01385 | 0.000844 | 1.50E-60 | 269.39 |
| rs6539808 | C | G | 0.381806 | 0.004717 | 0.00086 | 4.20E-08 | 30.07 |
| rs4766241 | G | A | 0.511834 | -0.00521 | 0.000835 | 4.30E-10 | 38.98 |
| rs73206853 | C | A | 0.119497 | -0.00847 | 0.001279 | 3.60E-11 | 43.83 |
| rs943722 | G | T | 0.910409 | 0.008005 | 0.001467 | 4.90E-08 | 29.76 |
| rs1535792 | A | T | 0.756297 | -0.00559 | 0.000967 | 7.40E-09 | 33.43 |
| rs28540738 | G | A | 0.322015 | -0.00517 | 0.00089 | 6.30E-09 | 33.73 |
| rs1011121 | G | A | 0.60033 | -0.00566 | 0.000848 | 2.60E-11 | 44.47 |
| rs12445022 | A | G | 0.338796 | -0.00541 | 0.000889 | 1.10E-09 | 37.08 |
| rs17652520 | A | G | 0.229955 | 0.009295 | 0.000989 | 5.70E-21 | 88.27 |
| rs3899444 | C | T | 0.683765 | 0.004952 | 0.000894 | 3.10E-08 | 30.67 |
| rs11662585 | G | A | 0.529403 | -0.00522 | 0.000834 | 3.80E-10 | 39.20 |
| rs74182635 | G | A | 0.053411 | 0.010397 | 0.001845 | 1.70E-08 | 31.76 |
| rs1555132 | A | C | 0.353545 | 0.005014 | 0.000878 | 1.10E-08 | 32.62 |
| rs9610533 | A | G | 0.466323 | -0.00473 | 0.000839 | 1.70E-08 | 31.76 |

SNP: single nucleotide polymorphism; A1: effect allele; A2: other allele; EAF: effect allele frequency.

**Characteristics of the 21** **back pain associated instruments**

| SNP | A1 | A2 | EAF | Beta | SE | *P*-value | *F*-statistic |
| --- | --- | --- | --- | --- | --- | --- | --- |
| rs6684344 | C | T | 0.183107 | 0.006823 | 0.001197 | 1.20E-08 | 32.51 |
| rs13132174 | C | G | 0.693643 | 0.005572 | 0.000982 | 1.40E-08 | 32.22 |
| rs13107325 | T | C | 0.074888 | 0.009634 | 0.001725 | 2.30E-08 | 31.19 |
| rs2066928 | G | A | 0.529331 | 0.005313 | 0.000924 | 8.90E-09 | 33.07 |
| rs10475978 | C | G | 0.475774 | -0.00534 | 0.000909 | 4.30E-09 | 34.50 |
| rs2910576 | T | A | 0.809763 | -0.00634 | 0.001159 | 4.50E-08 | 29.93 |
| rs4703253 | C | T | 0.682844 | 0.005693 | 0.000976 | 5.30E-09 | 34.06 |
| rs6907508 | G | A | 0.115595 | 0.008391 | 0.00142 | 3.40E-09 | 34.93 |
| rs77884325 | A | T | 0.136098 | 0.00748 | 0.001356 | 3.50E-08 | 30.43 |
| rs1865442 | T | C | 0.180639 | 0.008927 | 0.001184 | 4.70E-14 | 56.87 |
| rs7814941 | G | A | 0.203015 | -0.00867 | 0.001128 | 1.50E-14 | 59.11 |
| rs11507683 | T | C | 0.122042 | 0.007675 | 0.001384 | 2.90E-08 | 30.75 |
| rs10870267 | T | C | 0.55696 | -0.00539 | 0.000917 | 4.10E-09 | 34.58 |
| rs2219837 | C | T | 0.600837 | 0.006213 | 0.000927 | 2.10E-11 | 44.92 |
| rs2672596 | A | G | 0.254786 | -0.00679 | 0.001044 | 7.70E-11 | 42.33 |
| rs56290807 | A | C | 0.172829 | 0.010079 | 0.001205 | 6.00E-17 | 69.98 |
| rs146700983 | T | C | 0.011296 | -0.02831 | 0.004733 | 2.20E-09 | 35.78 |
| rs8018823 | A | G | 0.28693 | -0.00575 | 0.001005 | 1.00E-08 | 32.75 |
| rs10502971 | A | G | 0.428692 | 0.005003 | 0.000917 | 4.90E-08 | 29.76 |
| rs13169 | G | C | 0.138234 | 0.007322 | 0.001311 | 2.40E-08 | 31.19 |
| rs35318830 | G | T | 0.115728 | 0.007835 | 0.001426 | 3.90E-08 | 30.18 |

SNP: single nucleotide polymorphism; A1: effect allele; A2: other allele; EAF: effect allele frequency.

**Characteristics of the 6** **neck or shoulder pain associated instruments**

| SNP | A1 | A2 | EAF | Beta | SE | *P*-value | *F*-statistic |
| --- | --- | --- | --- | --- | --- | --- | --- |
| rs61779314 | T | C | 0.185979 | 0.006177 | 0.001128 | 4.30E-08 | 30.00 |
| rs13107325 | T | C | 0.074888 | 0.010907 | 0.001665 | 5.70E-11 | 42.93 |
| rs1476535 | T | C | 0.561666 | 0.00489 | 0.000888 | 3.70E-08 | 30.32 |
| rs62056490 | A | C | 0.794202 | -0.0062 | 0.001089 | 1.20E-08 | 32.46 |
| rs9889282 | C | A | 0.387918 | 0.006232 | 0.000902 | 4.80E-12 | 47.75 |
| rs7248205 | T | C | 0.600262 | -0.00502 | 0.000896 | 2.10E-08 | 31.42 |

SNP: single nucleotide polymorphism; A1: effect allele; A2: other allele; EAF: effect allele frequency.

**Characteristics of the 4** **hip pain associated instruments**

| SNP | A1 | A2 | EAF | Beta | SE | *P*-value | *F*-statistic |
| --- | --- | --- | --- | --- | --- | --- | --- |
| rs1260378 | A | G | 0.395749 | -0.00402 | 0.00067 | 1.90E-09 | 36.03 |
| rs2910298 | A | T | 0.882445 | 0.005675 | 0.00102 | 2.60E-08 | 30.98 |
| rs4724523 | G | A | 0.405368 | -0.00376 | 0.000672 | 2.10E-08 | 31.40 |
| rs77641763 | T | C | 0.12249 | 0.00571 | 0.001002 | 1.20E-08 | 32.51 |

SNP: single nucleotide polymorphism; A1: effect allele; A2: other allele; EAF: effect allele frequency.

**Characteristics of the 11** **knee pain associated instruments**

| SNP | A1 | A2 | EAF | Beta | SE | *P*-value | *F-statistic* |
| --- | --- | --- | --- | --- | --- | --- | --- |
| rs1782815 | A | C | 0.834357 | -0.00679 | 0.001146 | 3.10E-09 | 35.12 |
| rs3771501 | G | A | 0.524179 | -0.00475 | 0.000855 | 2.80E-08 | 30.81 |
| rs7627910 | C | T | 0.460539 | 0.004783 | 0.000856 | 2.30E-08 | 31.25 |
| rs13107325 | T | C | 0.074888 | 0.009456 | 0.00162 | 5.30E-09 | 34.07 |
| rs1799907 | T | A | 0.313386 | 0.006752 | 0.000919 | 2.00E-13 | 54.02 |
| rs3892354 | G | T | 0.585366 | 0.00506 | 0.000866 | 5.10E-09 | 34.14 |
| rs919642 | T | A | 0.268319 | 0.007717 | 0.000984 | 4.40E-15 | 61.49 |
| rs922160 | G | A | 0.644882 | 0.00519 | 0.000891 | 5.60E-09 | 33.97 |
| rs2899611 | G | T | 0.506529 | -0.00568 | 0.000855 | 3.10E-11 | 44.10 |
| rs892087 | T | C | 0.632201 | -0.00625 | 0.000883 | 1.50E-12 | 50.03 |
| rs143384 | G | A | 0.404402 | -0.0083 | 0.000867 | 1.00E-21 | 91.68 |

SNP: single nucleotide polymorphism; A1: effect allele; A2: other allele; EAF: effect allele frequency.

**Characteristics of the 18 facial pain associated instruments**

| SNP | A1 | A2 | EAF | Beta | SE | *P*-value | *F*-statistic |
| --- | --- | --- | --- | --- | --- | --- | --- |
| rs72694537 | T | C | 0.199463 | 0.00164 | 0.000355 | 3.80E-06 | 21.34 |
| rs9645327 | T | C | 0.117246 | 0.002054 | 0.00044 | 3.00E-06 | 21.83 |
| rs4416176 | C | T | 0.095787 | 0.002218 | 0.000477 | 3.30E-06 | 21.63 |
| rs12712737 | G | A | 0.420497 | 0.001344 | 0.000287 | 2.70E-06 | 22.00 |
| rs3773416 | G | C | 0.047162 | 0.003035 | 0.000665 | 5.00E-06 | 20.85 |
| rs2199214 | C | T | 0.172202 | 0.001719 | 0.000374 | 4.40E-06 | 21.08 |
| rs6967391 | T | A | 0.195562 | 0.001753 | 0.00036 | 1.10E-06 | 23.68 |
| rs62446221 | C | T | 0.100643 | 0.002152 | 0.000472 | 5.00E-06 | 20.82 |
| rs34311004 | C | T | 0.042334 | 0.003352 | 0.000711 | 2.40E-06 | 22.24 |
| rs9643685 | T | C | 0.765689 | -0.00153 | 0.000333 | 4.30E-06 | 21.13 |
| rs72711207 | C | T | 0.0976 | -0.00228 | 0.000476 | 1.70E-06 | 22.95 |
| rs7937459 | A | C | 0.402997 | 0.001399 | 0.000291 | 1.60E-06 | 23.06 |
| rs7965652 | A | G | 0.354152 | 0.001379 | 0.000293 | 2.60E-06 | 22.10 |
| rs8005612 | C | T | 0.207409 | -0.00166 | 0.000349 | 1.90E-06 | 22.70 |
| rs8003983 | A | C | 0.1504 | -0.00193 | 0.000407 | 2.20E-06 | 22.37 |
| rs12445269 | T | C | 0.41534 | -0.00147 | 0.000286 | 2.90E-07 | 26.33 |
| rs76714322 | T | C | 0.105959 | 0.002189 | 0.000458 | 1.70E-06 | 22.88 |
| rs34234617 | G | C | 0.051931 | -0.00305 | 0.000648 | 2.50E-06 | 22.21 |

SNP: single nucleotide polymorphism; A1: effect allele; A2: other allele; EAF: effect allele frequency.

**Characteristics of the 35 stomach or abdominal pain associated instruments**

| SNP | A1 | A2 | EAF | Beta | SE | *P*-value | *F-statistic* |
| --- | --- | --- | --- | --- | --- | --- | --- |
| rs12410770 | C | T | 0.433997 | 0.002935 | 0.000589 | 6.20E-07 | 24.84 |
| rs41265901 | G | A | 0.051992 | -0.00605 | 0.00131 | 4.00E-06 | 21.28 |
| rs604149 | C | A | 0.307857 | 0.003056 | 0.000631 | 1.30E-06 | 23.43 |
| rs7524680 | A | G | 0.276389 | 0.003076 | 0.000651 | 2.30E-06 | 22.30 |
| rs12743193 | G | C | 0.095805 | 0.005582 | 0.000994 | 2.00E-08 | 31.54 |
| rs140894039 | T | C | 0.010016 | 0.014834 | 0.002976 | 6.20E-07 | 24.84 |
| rs62137126 | G | A | 0.120679 | -0.00436 | 0.000899 | 1.20E-06 | 23.58 |
| rs11718858 | T | G | 0.212451 | 0.003275 | 0.000715 | 4.70E-06 | 20.97 |
| rs767824 | G | A | 0.692684 | -0.003 | 0.000631 | 2.00E-06 | 22.63 |
| rs2713862 | A | T | 0.454856 | -0.00281 | 0.000587 | 1.70E-06 | 22.90 |
| rs4835870 | A | G | 0.37486 | 0.002838 | 0.000601 | 2.40E-06 | 22.28 |
| rs4276378 | G | A | 0.410709 | 0.002774 | 0.000593 | 2.90E-06 | 21.87 |
| rs4868269 | T | C | 0.447958 | 0.003009 | 0.000586 | 2.80E-07 | 26.37 |
| rs8283 | G | A | 0.171547 | 0.004032 | 0.000772 | 1.70E-07 | 27.31 |
| rs34544636 | T | C | 0.53626 | 0.003002 | 0.000646 | 3.40E-06 | 21.58 |
| rs7742523 | A | G | 0.135346 | 0.004131 | 0.000851 | 1.20E-06 | 23.56 |
| rs200468 | G | C | 0.195506 | -0.00339 | 0.000734 | 3.90E-06 | 21.29 |
| rs62462670 | C | A | 0.136047 | -0.00435 | 0.000852 | 3.20E-07 | 26.13 |
| rs7781732 | A | T | 0.690777 | -0.00333 | 0.000633 | 1.50E-07 | 27.60 |
| rs143466526 | T | C | 0.011935 | -0.01327 | 0.0029 | 4.70E-06 | 20.95 |
| rs7012413 | C | T | 0.696705 | -0.00311 | 0.000633 | 8.90E-07 | 24.16 |
| rs4392872 | C | T | 0.4843 | 0.003075 | 0.000584 | 1.40E-07 | 27.68 |
| rs10156602 | G | A | 0.361068 | -0.0031 | 0.000611 | 3.90E-07 | 25.72 |
| rs11053654 | C | T | 0.548868 | 0.002824 | 0.000585 | 1.40E-06 | 23.31 |
| rs7133979 | A | G | 0.618904 | -0.00299 | 0.000599 | 6.00E-07 | 24.90 |
| rs2368831 | T | C | 0.568414 | 0.002738 | 0.000594 | 4.10E-06 | 21.23 |
| rs454790 | G | A | 0.626585 | -0.00312 | 0.00061 | 3.10E-07 | 26.16 |
| rs9531936 | G | A | 0.287679 | 0.003073 | 0.000647 | 2.10E-06 | 22.55 |
| rs35630916 | T | G | 0.075641 | -0.0053 | 0.001101 | 1.50E-06 | 23.14 |
| rs2683126 | G | A | 0.587945 | 0.002885 | 0.000593 | 1.10E-06 | 23.69 |
| rs1459959 | A | C | 0.380447 | 0.002827 | 0.000602 | 2.70E-06 | 22.01 |
| rs11073967 | G | A | 0.377094 | 0.003058 | 0.000603 | 4.00E-07 | 25.69 |
| rs79201173 | T | A | 0.030976 | 0.007809 | 0.00168 | 3.40E-06 | 21.60 |
| rs149421151 | A | G | 0.041737 | -0.00731 | 0.001463 | 5.80E-07 | 24.98 |
| rs11664989 | A | C | 0.506865 | -0.00274 | 0.000583 | 2.50E-06 | 22.18 |

SNP: single nucleotide polymorphism; A1: effect allele; A2: other allele; EAF: effect allele frequency.

**Characteristics of the 38 “None of the above” associated instruments**

| SNP | A1 | A2 | EAF | Beta | SE | *P*-value | *F*-statistic |
| --- | --- | --- | --- | --- | --- | --- | --- |
| rs12096443 | T | C | 0.434814 | -0.00687 | 0.001025 | 2.10E-11 | 44.91 |
| rs127204 | C | A | 0.399707 | -0.00649 | 0.001037 | 4.00E-10 | 39.13 |
| rs61779314 | T | C | 0.185979 | -0.00987 | 0.001308 | 4.50E-14 | 56.95 |
| rs565337 | G | A | 0.783866 | -0.00692 | 0.001234 | 2.00E-08 | 31.47 |
| rs2216172 | A | G | 0.574689 | 0.005723 | 0.001027 | 2.50E-08 | 31.05 |
| rs2111592 | A | G | 0.311981 | -0.00602 | 0.001095 | 4.00E-08 | 30.15 |
| rs28428925 | A | G | 0.136667 | -0.00927 | 0.001486 | 4.40E-10 | 38.94 |
| rs10640 | A | G | 0.306245 | 0.007619 | 0.001101 | 4.50E-12 | 47.88 |
| rs11729080 | A | G | 0.171271 | 0.007452 | 0.001346 | 3.10E-08 | 30.64 |
| rs13107325 | T | C | 0.074888 | -0.01249 | 0.001931 | 1.00E-10 | 41.79 |
| rs13136239 | A | G | 0.343549 | 0.006104 | 0.001071 | 1.20E-08 | 32.48 |
| rs4240390 | C | G | 0.339538 | -0.00624 | 0.001075 | 6.40E-09 | 33.71 |
| rs2983897 | A | G | 0.221662 | -0.00707 | 0.001225 | 7.70E-09 | 33.35 |
| rs17600945 | C | A | 0.385543 | 0.006188 | 0.001042 | 2.90E-09 | 35.27 |
| rs78640489 | T | G | 0.135172 | -0.00846 | 0.001486 | 1.30E-08 | 32.37 |
| rs7797335 | A | T | 0.663003 | -0.00684 | 0.001076 | 2.10E-10 | 40.36 |
| rs11782074 | T | G | 0.383657 | -0.00643 | 0.001062 | 1.40E-09 | 36.74 |
| rs588818 | G | A | 0.31293 | 0.007233 | 0.001098 | 4.60E-11 | 43.36 |
| rs7905192 | T | C | 0.558017 | 0.005762 | 0.001022 | 1.70E-08 | 31.79 |
| rs7121047 | T | A | 0.387732 | -0.00655 | 0.001042 | 3.30E-10 | 39.48 |
| rs7112291 | A | T | 0.379042 | 0.006565 | 0.001046 | 3.40E-10 | 39.42 |
| rs10768286 | A | G | 0.206339 | -0.0073 | 0.001297 | 1.80E-08 | 31.72 |
| rs2292239 | G | T | 0.653666 | -0.00582 | 0.001066 | 4.80E-08 | 29.78 |
| rs7973253 | G | A | 0.369084 | -0.00603 | 0.001054 | 1.00E-08 | 32.79 |
| rs11172113 | C | T | 0.411988 | 0.007149 | 0.001032 | 4.30E-12 | 48.00 |
| rs7996330 | T | C | 0.434926 | 0.006569 | 0.001035 | 2.20E-10 | 40.32 |
| rs11158800 | A | G | 0.501523 | -0.00563 | 0.001019 | 3.30E-08 | 30.51 |
| rs7162685 | T | C | 0.374927 | -0.00585 | 0.001057 | 3.10E-08 | 30.67 |
| rs7204708 | G | A | 0.736823 | 0.007703 | 0.001152 | 2.30E-11 | 44.71 |
| rs1635298 | A | T | 0.756306 | 0.006804 | 0.001176 | 7.20E-09 | 33.47 |
| rs8067800 | G | A | 0.614752 | 0.005923 | 0.001043 | 1.40E-08 | 32.25 |
| rs8614 | A | C | 0.182487 | -0.00731 | 0.001317 | 2.80E-08 | 30.84 |
| rs12453010 | T | C | 0.393344 | -0.00689 | 0.001045 | 4.20E-11 | 43.54 |
| rs11665567 | A | G | 0.209197 | -0.00695 | 0.001247 | 2.50E-08 | 31.09 |
| rs4423491 | G | C | 0.476341 | 0.005576 | 0.001018 | 4.30E-08 | 30.02 |
| rs910889 | C | T | 0.222634 | -0.0076 | 0.001224 | 5.50E-10 | 38.48 |
| rs2424245 | T | C | 0.126656 | 0.008452 | 0.001532 | 3.40E-08 | 30.45 |
| rs66486049 | C | G | 0.070619 | 0.011517 | 0.002022 | 1.20E-08 | 32.44 |

SNP: single nucleotide polymorphism; A1: effect allele; A2: other allele; EAF: effect allele frequency.
