# Supplementary material for: Exploring the bidirectional relationship between pain and mental disorders: a comprehensive Mendelian randomization study
Source: J Headache Pain. 2023 Jul 7;24(1):82. doi: 10.1186/s10194-023-01612-2 (PMC10326936; doi:10.1186/s10194-023-01612-2)
Supplement: Supplementary file 3 — Additional file 3: Supplementary file 3. MR results of localized pain on risk of sleeplessness/insomnia, anxiety/panic attacks and depression with scatter plots. [file 10194_2023_1612_MOESM3_ESM.pdf]

# Supplementary file 3

MR results of localized pain on risk of sleeplessness/insomnia,

anxiety/panic attacks and depression with scatter plots

**Single-variable MR results of localized pain on risk of sleeplessness/insomnia, anxiety/panic attacks and depression**

| Exposure | Method | Sleeplessness / insomnia | | | Anxiety/panic attacks | | | Depression | | |
|---|---|---|---|---|---|---|---|---|---|---|
| | | N SNV | OR (95%CI) | *P* value | N SNV | OR (95%CI) | *P* value | N SNV | OR (95%CI) | *P* value |
| Headache | IVW (FEM) | 45 | 1.14 (1.05-1.24) | 0.003 | 45 | 1.00 (0.99-1.02) | 0.56 | 45 | 1.06 (1.03-1.08) | <0.001 |
| | IVW (REM) | 45 | 1.14 (1.01-1.28) | 0.04 | 45 | 1.00 (0.99-1.02) | 0.63 | 45 | 1.06 (1.02-1.09) | <0.001 |
| | Weighted median | 45 | 1.15 (1.02-1.31) | 0.03 | 45 | 1.00 (0.98-1.03) | 0.69 | 45 | 1.04 (1.01-1.08) | 0.02 |
| | MR Egger | 45 | 0.92 (0.65-1.30) | 0.62 | 45 | 1.00 (0.95-1.05) | 0.91 | 45 | 1.01 (0.93-1.11) | 0.77 |
| Facial pain* | IVW (FEM) | 16 | 1.31 (0.69-2.49) | 0.41 | 16 | 1.00 (0.90-1.11) | 0.98 | 16 | 1.13 (0.95-1.35) | 0.16 |
| | IVW (REM) | 16 | 1.31 (0.68-2.54) | 0.42 | 16 | 1.00 (0.88-1.14) | 0.99 | 16 | 1.13 (0.94-1.36) | 0.18 |
| | Weighted median | 16 | 2.01 (0.82-4.91) | 0.13 | 16 | 1.01 (0.87-1.17) | 0.91 | 16 | 1.08 (0.84-1.39) | 0.55 |
| | MR Egger | 16 | 5.22 (0.30-90.81) | 0.28 | 16 | 0.80 (0.45-1.41) | 0.45 | 16 | 1.08 (0.47-2.45) | 0.86 |
| Neck or shoulder pain | IVW (FEM) | 6 | 1.95 (1.50-2.54) | <0.001 | 6 | 1.00 (0.96-1.04) | 0.96 | 6 | 1.09 (1.01-1.17) | 0.02 |
| | IVW (REM) | 6 | 1.95 (1.03-3.68) | 0.03 | 6 | 1.00 (0.96-1.04) | 0.96 | 6 | 1.09 (1.00-1.18) | 0.04 |
| | Weighted median | 6 | 1.97 (1.32-2.94) | <0.001 | 6 | 1.00 (0.95-1.06) | 0.95 | 6 | 1.10 (1.01-1.21) | 0.04 |
| | MR Egger | 6 | 2.39 (0.11-50.76) | 0.61 | 6 | 1.07 (0.89-1.30) | 0.50 | 6 | 1.07 (0.73-1.58) | 0.73 |
| Back pain | IVW (FEM) | 20 | 1.40 (1.22-1.60) | <0.001 | 20 | 1.00 (0.98-1.02) | 0.94 | 19 | 1.08 (1.04-1.13) | <0.001 |
| | IVW (REM) | 20 | 1.40 (1.18-1.65) | <0.001 | 20 | 1.00 (0.98-1.02) | 0.94 | 19 | 1.08 (1.03-1.14) | 0.002 |
| | Weighted median | 20 | 1.26 (1.04-1.54) | 0.02 | 20 | 1.01 (0.98-1.05) | 0.41 | 19 | 1.06 (1.00-1.11) | 0.03 |
| | MR Egger | 20 | 1.14 (0.60-2.20) | 0.69 | 20 | 0.97 (0.89-1.06) | 0.51 | 19 | 0.90 (0.71-1.13) | 0.37 |
| Stomach and abdominal pain* | IVW (FEM) | 30 | 1.79 (1.44-2.23) | <0.001 | 30 | 1.03 (1.00-1.07) | 0.06 | 28 | 1.19 (1.12-1.25) | <0.001 |
| | IVW (REM) | 30 | 1.79 (1.25-2.56) | 0.001 | 30 | 1.03 (0.99-1.08) | 0.13 | 28 | 1.19 (1.11-1.26) | <0.001 |
| | Weighted median | 30 | 2.10 (1.50-2.95) | <0.001 | 30 | 1.03 (0.98-1.09) | 0.24 | 28 | 1.17 (1.07-1.28) | <0.001 |
| | MR Egger | 30 | 0.44 (0.14-1.37) | 0.17 | 30 | 0.91 (0.79-1.06) | 0.23 | 28 | 1.14 (0.86-1.50) | 0.37 |
| Hip pain | IVW (FEM) | 4 | 2.29 (1.18-4.45) | <0.001 | 4 | 1.01 (0.97-1.05) | 0.83 | 4 | 1.17 (1.03-1.33) | 0.01 |
| | IVW (REM) | 4 | 2.29 (1.46-3.60) | 0.01 | 4 | 1.01 (0.94-1.08) | 0.69 | 4 | 1.17 (0.94-1.46) | 0.16 |
| | Weighted median | 4 | 1.76 (0.97-3.17) | 0.06 | 4 | 0.99 (0.91-1.07) | 0.81 | 4 | 1.13 (0.95-1.33) | 0.17 |
| | MR Egger | 4 | 22.35 (1.12-447.57) | 0.17 | 4 | 1.12 (0.75-1.67) | 0.62 | 4 | 1.37 (0.32-5.89) | 0.71 |
| Knee pain | IVW (FEM) | 11 | 1.19 (0.99-1.42) | 0.09 | 11 | 0.99 (0.97-1.01) | 0.44 | 11 | 1.07 (1.03-1.12) | 0.004 |
| | IVW (REM) | 11 | 1.19 (0.83-1.71) | 0.35 | 11 | 0.99 (0.96-1.02) | 0.29 | 11 | 1.07 (1.02-1.13) | <0.001 |
| | Weighted median | 11 | 0.99 (0.76-1.30) | 0.96 | 11 | 0.99 (0.95-1.03) | 0.55 | 11 | 1.08 (1.01-1.15) | 0.02 |
| | MR Egger | 11 | 0.41 (0.08-2.16) | 0.32 | 11 | 1.01 (0.88-1.17) | 0.84 | 11 | 1.10 (0.86-1.39) | 0.47 |
| None of above | IVW (FEM) | 37 | 0.62 (0.56-0.67) | <0.001 | 37 | 0.98 (0.97-1.00) | 0.01 | 37 | 0.92 (0.90-0.95) | <0.001 |
| | IVW (REM) | 37 | 0.62 (0.54-0.70) | <0.001 | 37 | 0.98 (0.97-1.00) | 0.02 | 37 | 0.92 (0.90-0.95) | <0.001 |
| | Weighted median | 37 | 0.68 (0.59-0.79) | <0.001 | 37 | 0.97 (0.95-0.99) | 0.005 | 37 | 0.92 (0.89-0.96) | <0.001 |
| | MR Egger | 37 | 0.90 (0.40-2.07) | 0.81 | 37 | 0.97 (0.88-1.08) | 0.61 | 37 | 1.00 (0.86-1.17) | 0.96 |

Abbreviations: MR, mendelian randomization; N SNV, number of single-nucleotide variants; IVW, inverse-variance weighted; FEM, fixed effects models; REM, random effects models; OR, odds ratio; GWAS, genome-wide association studies.

Genetic instruments selected from localized pain GWASs, selection threshold *P* less than $5\times10^{-8}$, pruned at linkage disequilibrium $R^2$ less than 0.001 (10 000 kilobase pair window).

* If the number of SNPs available for analysis is less than 3, the selection threshold *P* will be adjusted to $5\times10^{-6}$.

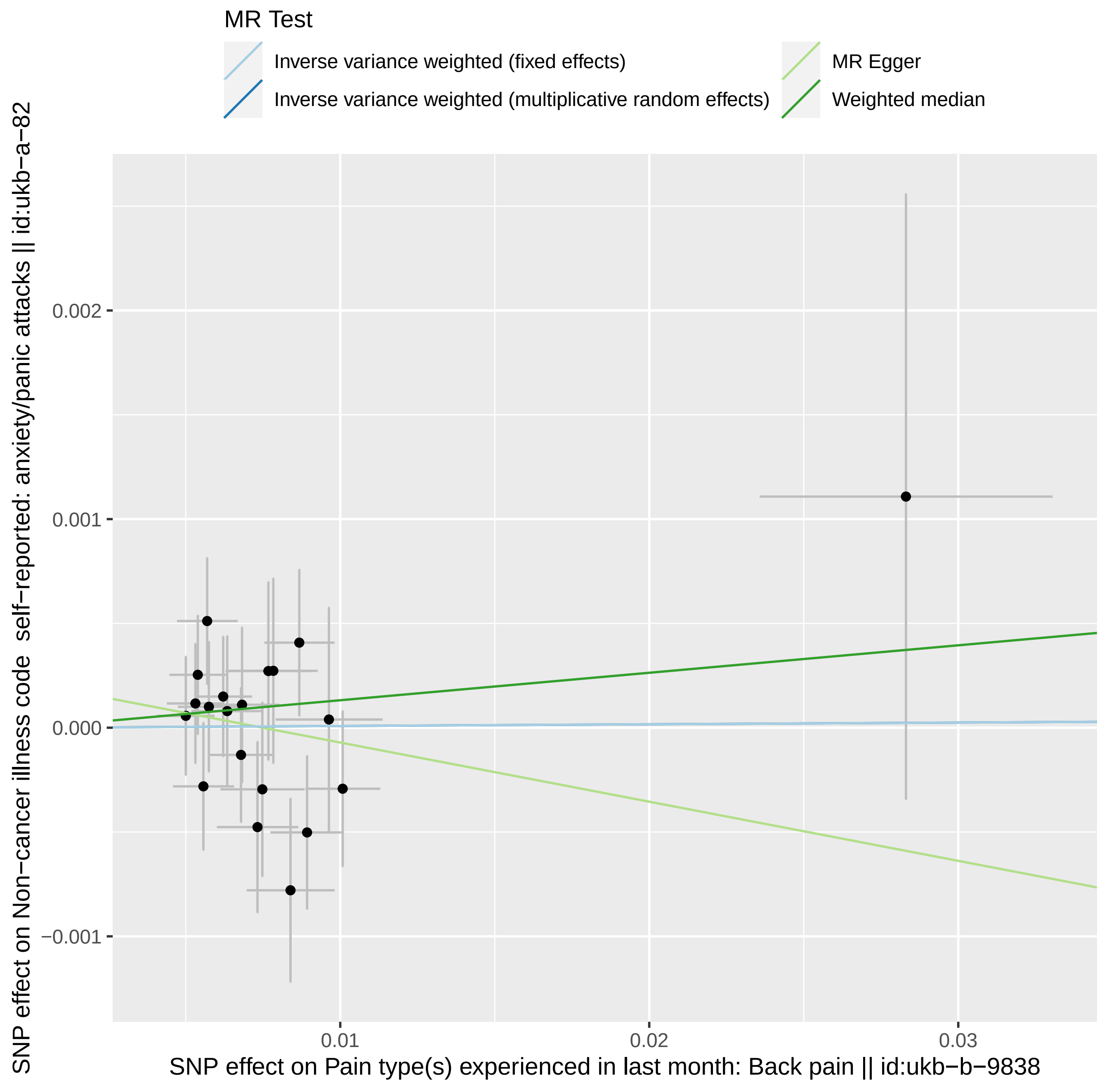

MR Test
Inverse variance weighted (fixed effects)
Inverse variance weighted (multiplicative random effects)
MR Egger
Weighted median
0.002
0.001
0.000
−0.001
0.01
0.02
0.03
SNP effect on Non−cancer illness code  self−reported: anxiety/panic attacks || id:ukb−a−82
SNP effect on Pain type(s) experienced in last month: Back pain || id:ukb−b−9838

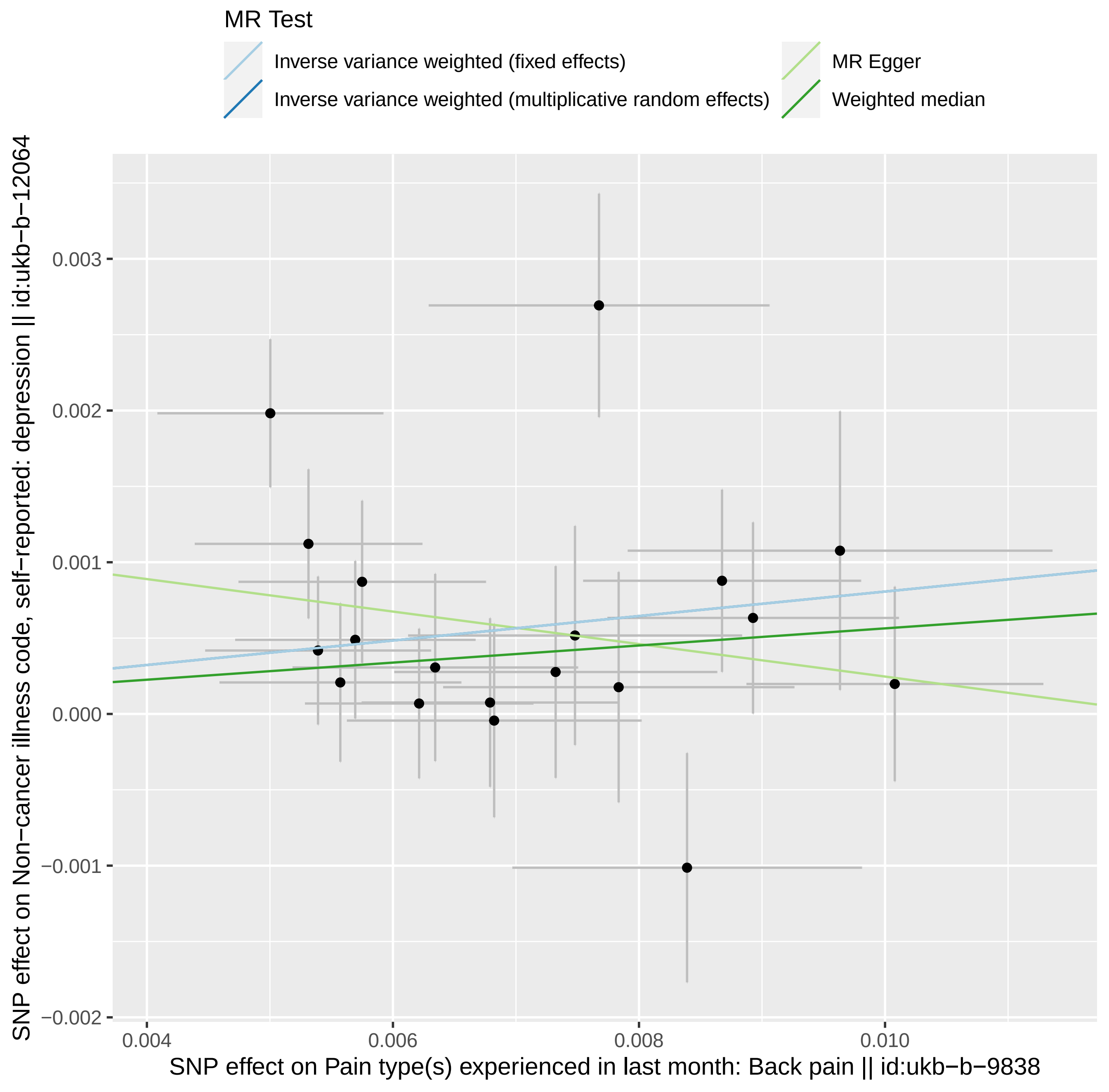
MR Test
Inverse variance weighted (fixed effects)
Inverse variance weighted (multiplicative random effects)
MR Egger
Weighted median
SNP effect on Non−cancer illness code, self−reported: depression || id:ukb−b−12064
0.003
0.002
0.001
0.000
−0.001
−0.002
0.004
0.006
0.008
0.010
SNP effect on Pain type(s) experienced in last month: Back pain || id:ukb−b−9838

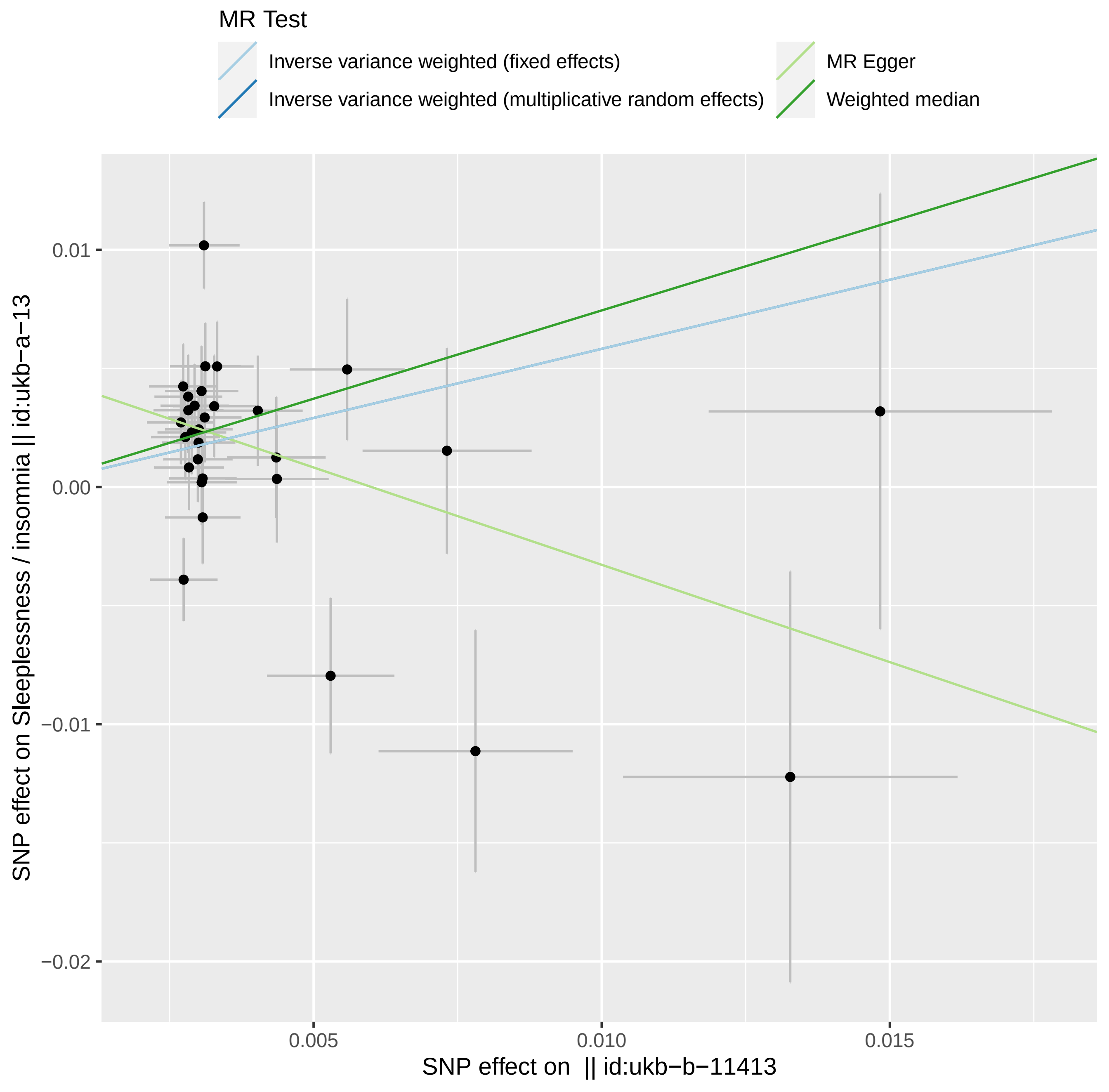
MR Test
Inverse variance weighted (fixed effects)
Inverse variance weighted (multiplicative random effects)
MR Egger
Weighted median
0.01
0.00
−0.01
−0.02
0.005
0.010
0.015
SNP effect on Sleeplessness / insomnia || id:ukb−a−13
SNP effect on || id:ukb−b−11413

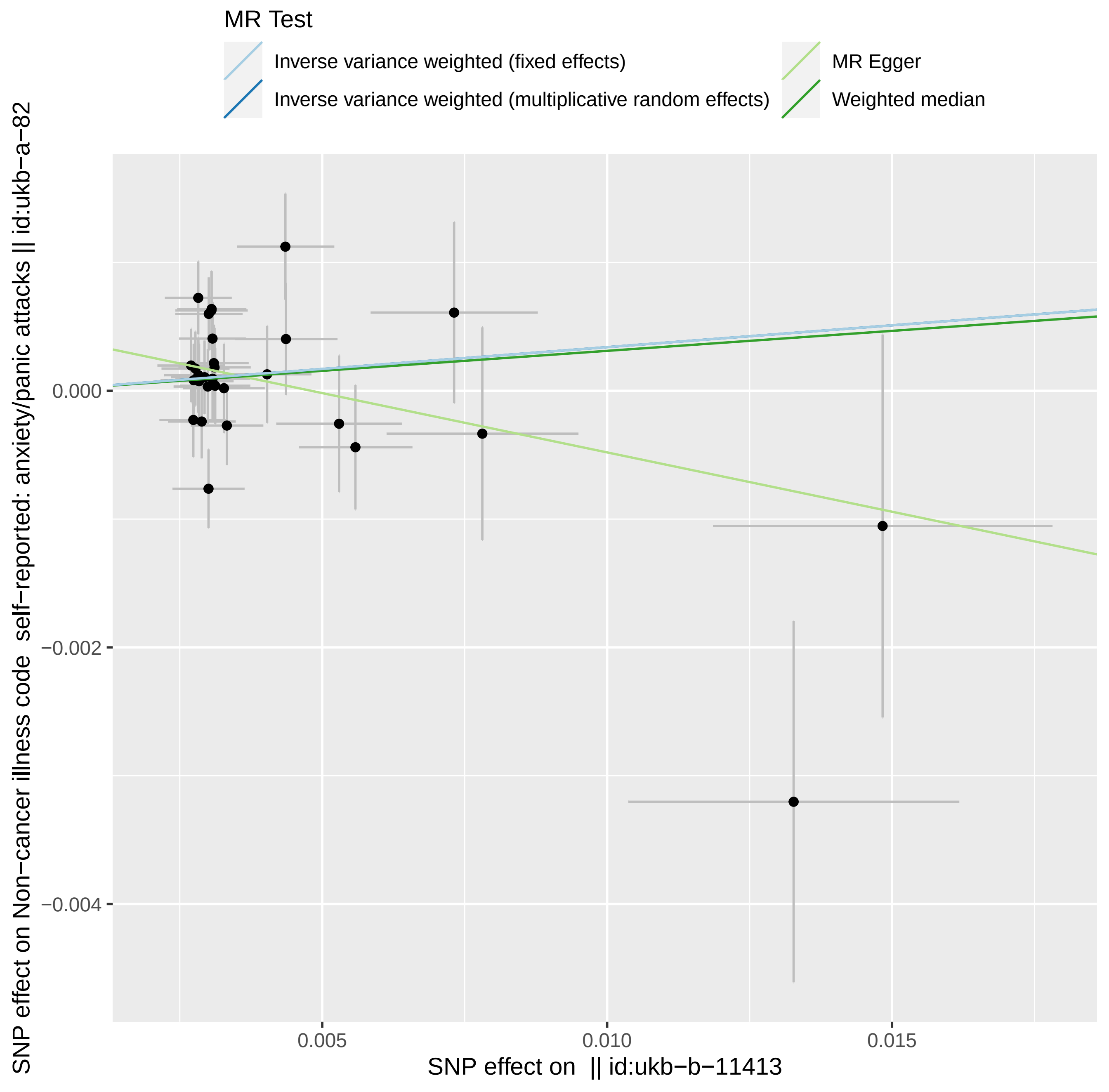

MR Test
Inverse variance weighted (fixed effects)
Inverse variance weighted (multiplicative random effects)
MR Egger
Weighted median
SNP effect on Non−cancer illness code self−reported: anxiety/panic attacks || id:ukb−a−82
0.000
−0.002
−0.004
0.005
0.010
0.015
SNP effect on || id:ukb−b−11413

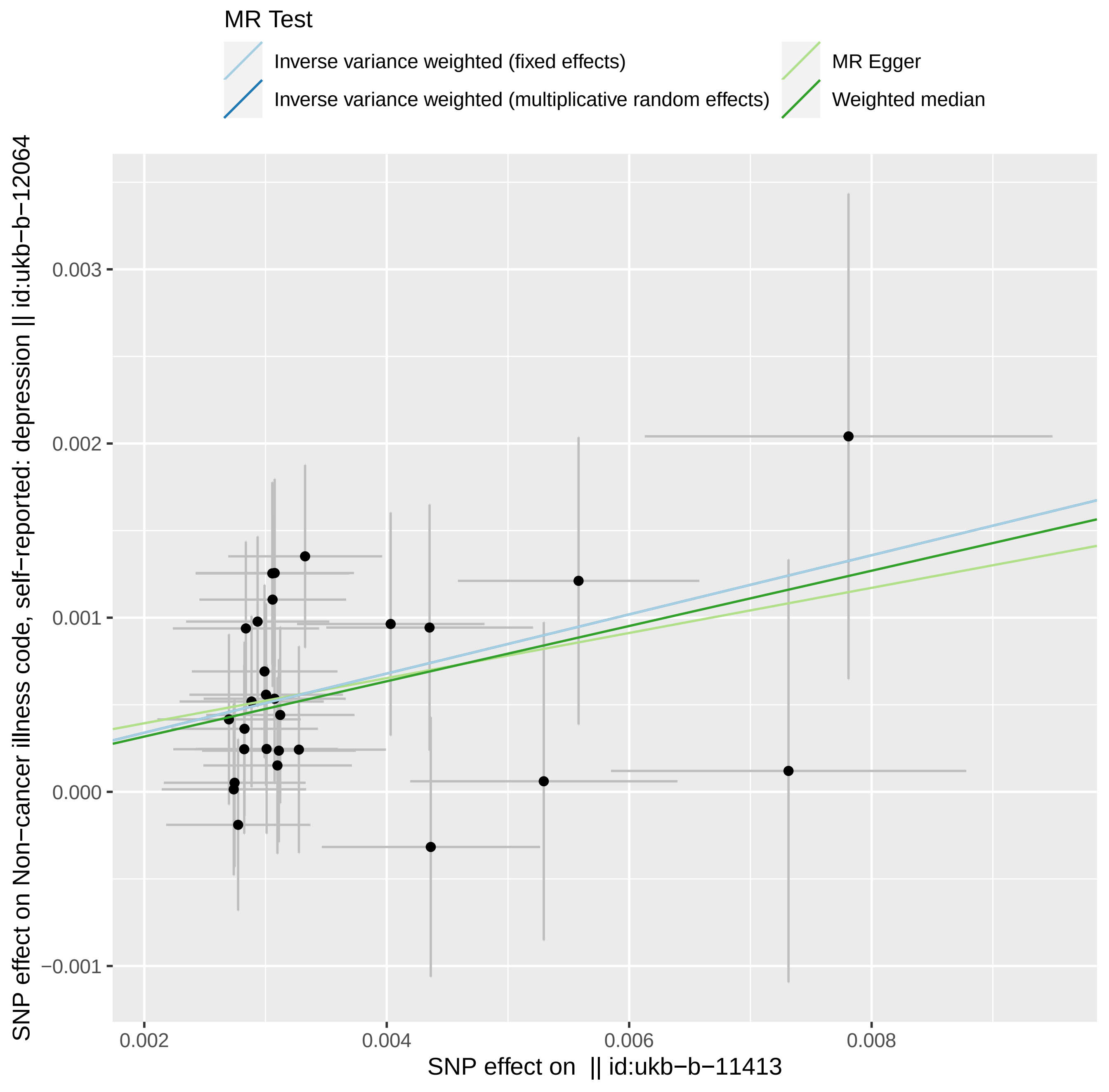
MR Test
Inverse variance weighted (fixed effects)
Inverse variance weighted (multiplicative random effects)
MR Egger
Weighted median
SNP effect on Non−cancer illness code, self−reported: depression || id:ukb−b−12064
0.003
0.002
0.001
0.000
−0.001
0.002
0.004
0.006
0.008
SNP effect on || id:ukb−b−11413

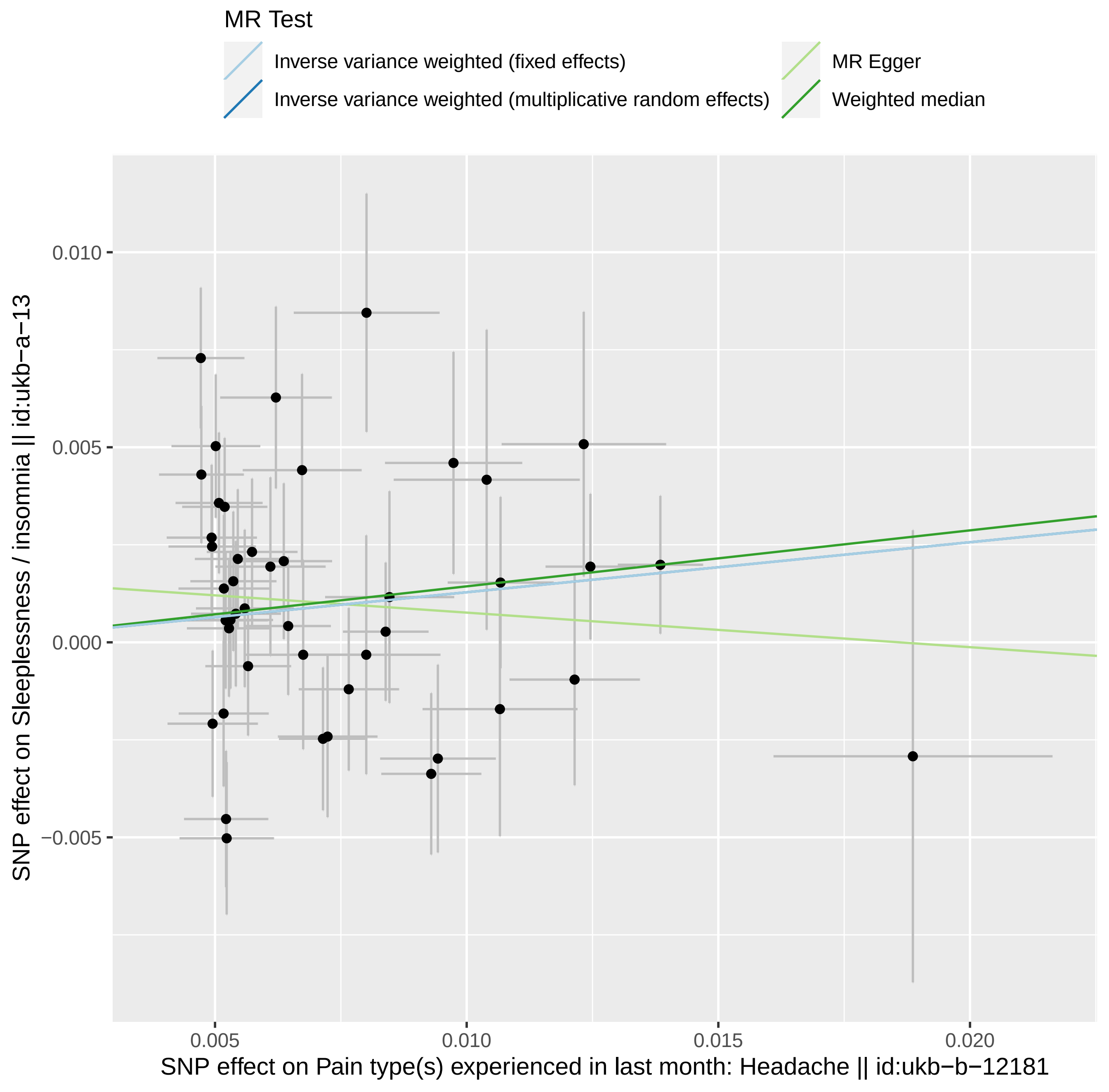

MR Test
Inverse variance weighted (fixed effects)
Inverse variance weighted (multiplicative random effects)
MR Egger
Weighted median
0.010
0.005
0.000
−0.005
0.005
0.010
0.015
0.020
SNP effect on Sleeplessness / insomnia || id:ukb−a−13
SNP effect on Pain type(s) experienced in last month: Headache || id:ukb−b−12181

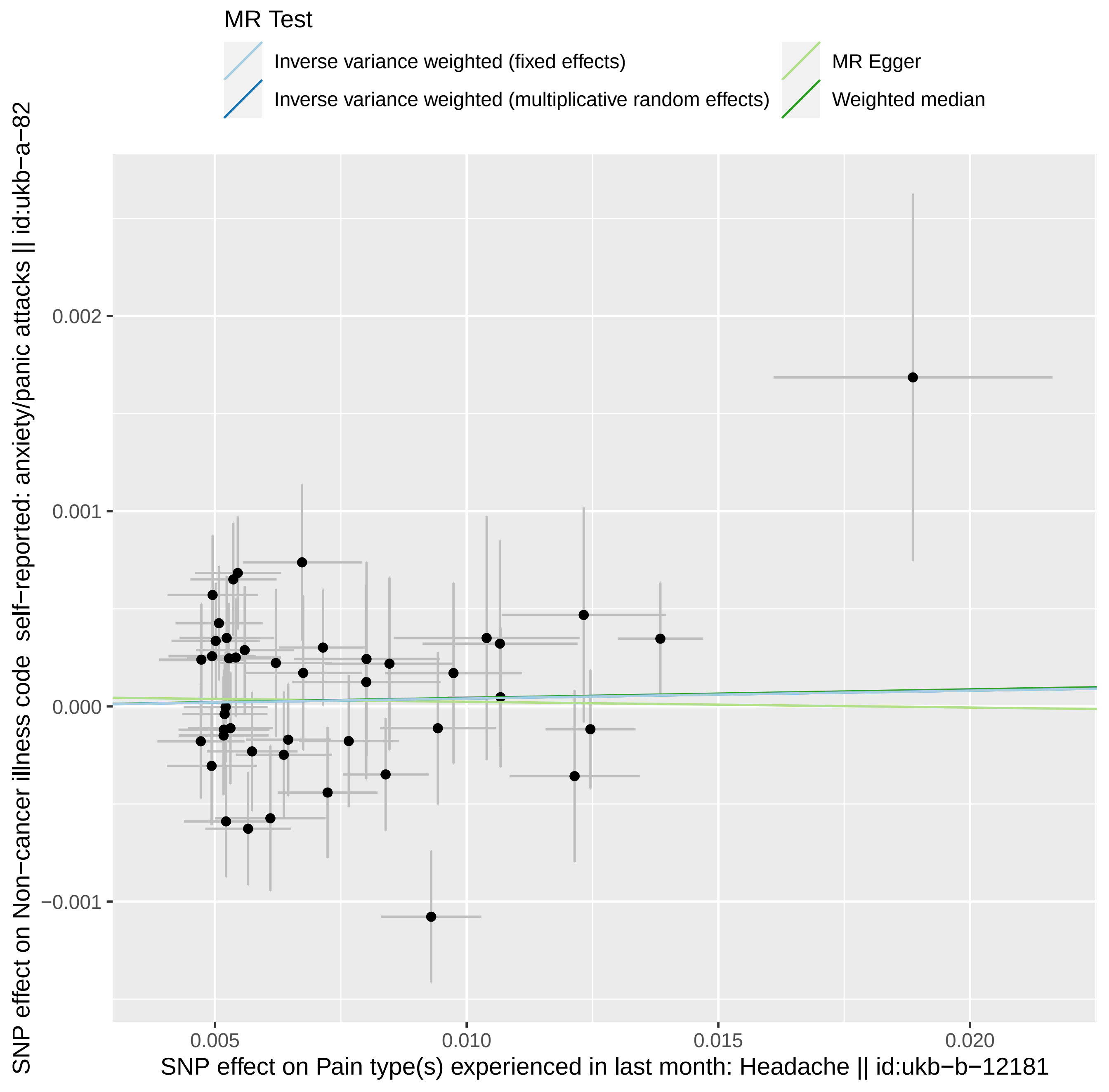

MR Test
Inverse variance weighted (fixed effects)
Inverse variance weighted (multiplicative random effects)
MR Egger
Weighted median
0.002
0.001
0.000
−0.001
0.005
0.010
0.015
0.020
SNP effect on Non−cancer illness code self−reported: anxiety/panic attacks || id:ukb−a−82
SNP effect on Pain type(s) experienced in last month: Headache || id:ukb−b−12181

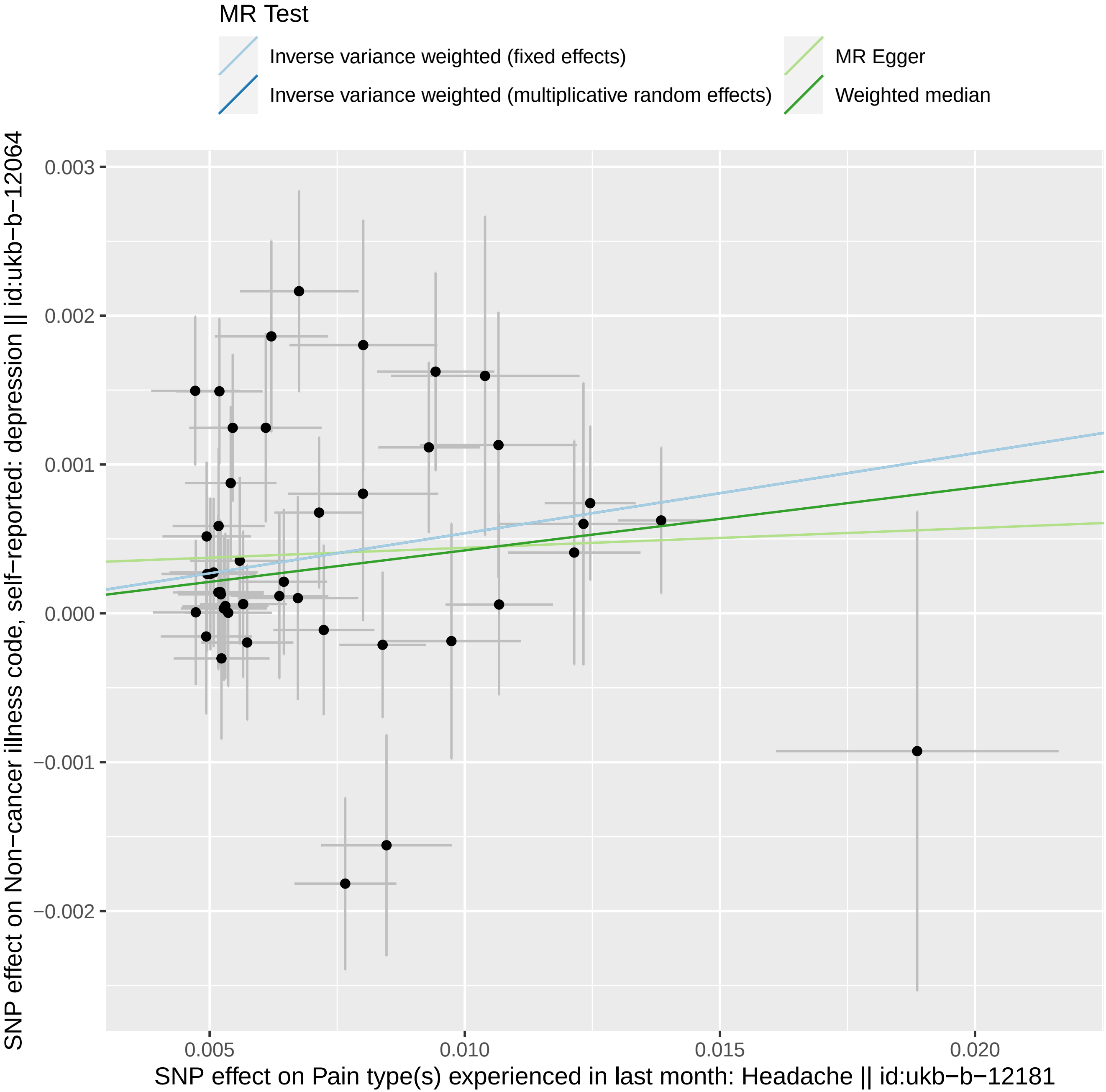
MR Test
Inverse variance weighted (fixed effects)
Inverse variance weighted (multiplicative random effects)
MR Egger
Weighted median
SNP effect on Non−cancer illness code, self−reported: depression || id:ukb−b−12064
0.003
0.002
0.001
0.000
−0.001
−0.002
0.005
0.010
0.015
0.020
SNP effect on Pain type(s) experienced in last month: Headache || id:ukb−b−12181

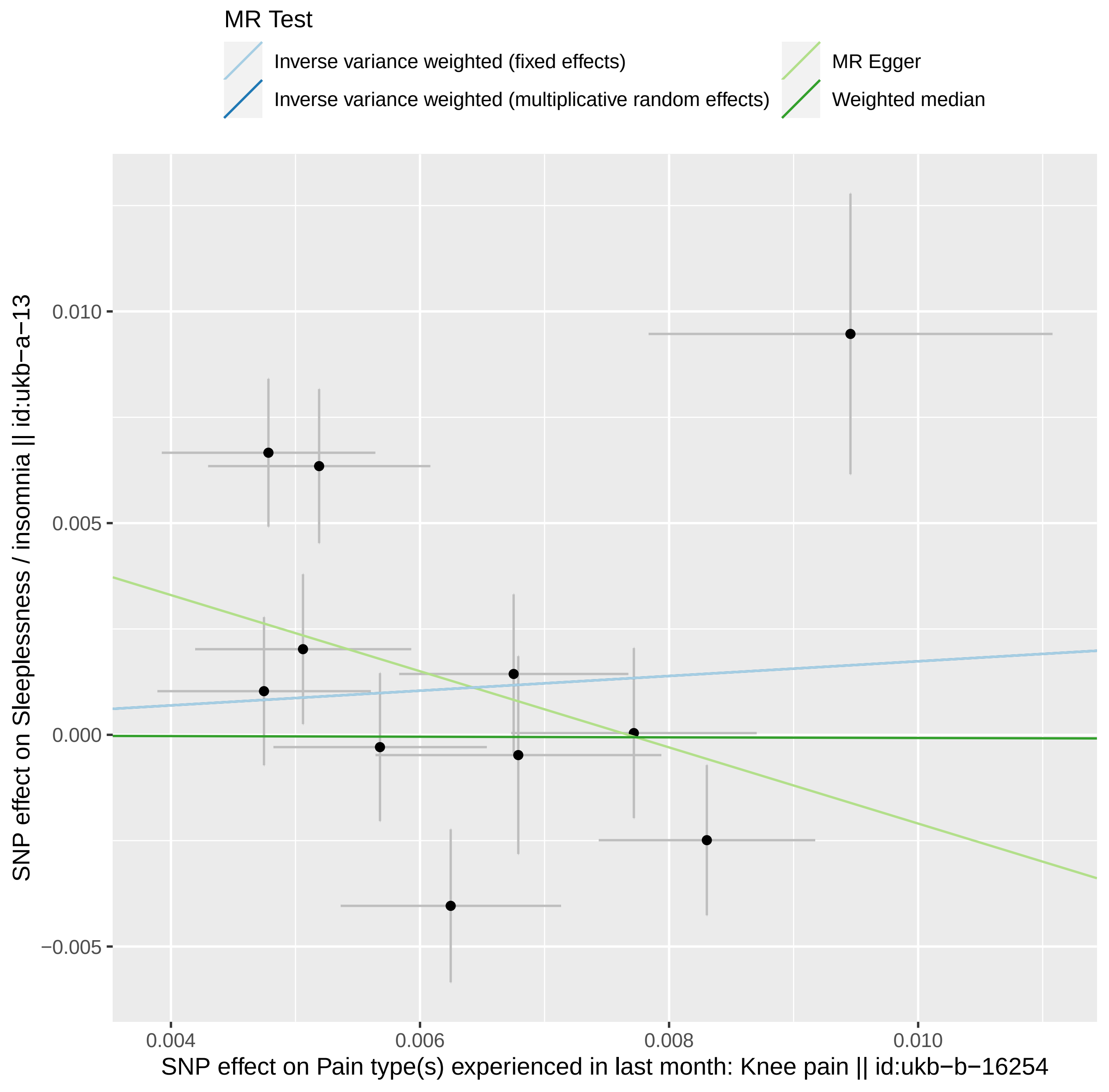

MR Test
Inverse variance weighted (fixed effects)
Inverse variance weighted (multiplicative random effects)
MR Egger
Weighted median
0.010
0.005
0.000
−0.005
0.004
0.006
0.008
0.010
SNP effect on Sleeplessness / insomnia || id:ukb−a−13
SNP effect on Pain type(s) experienced in last month: Knee pain || id:ukb−b−16254

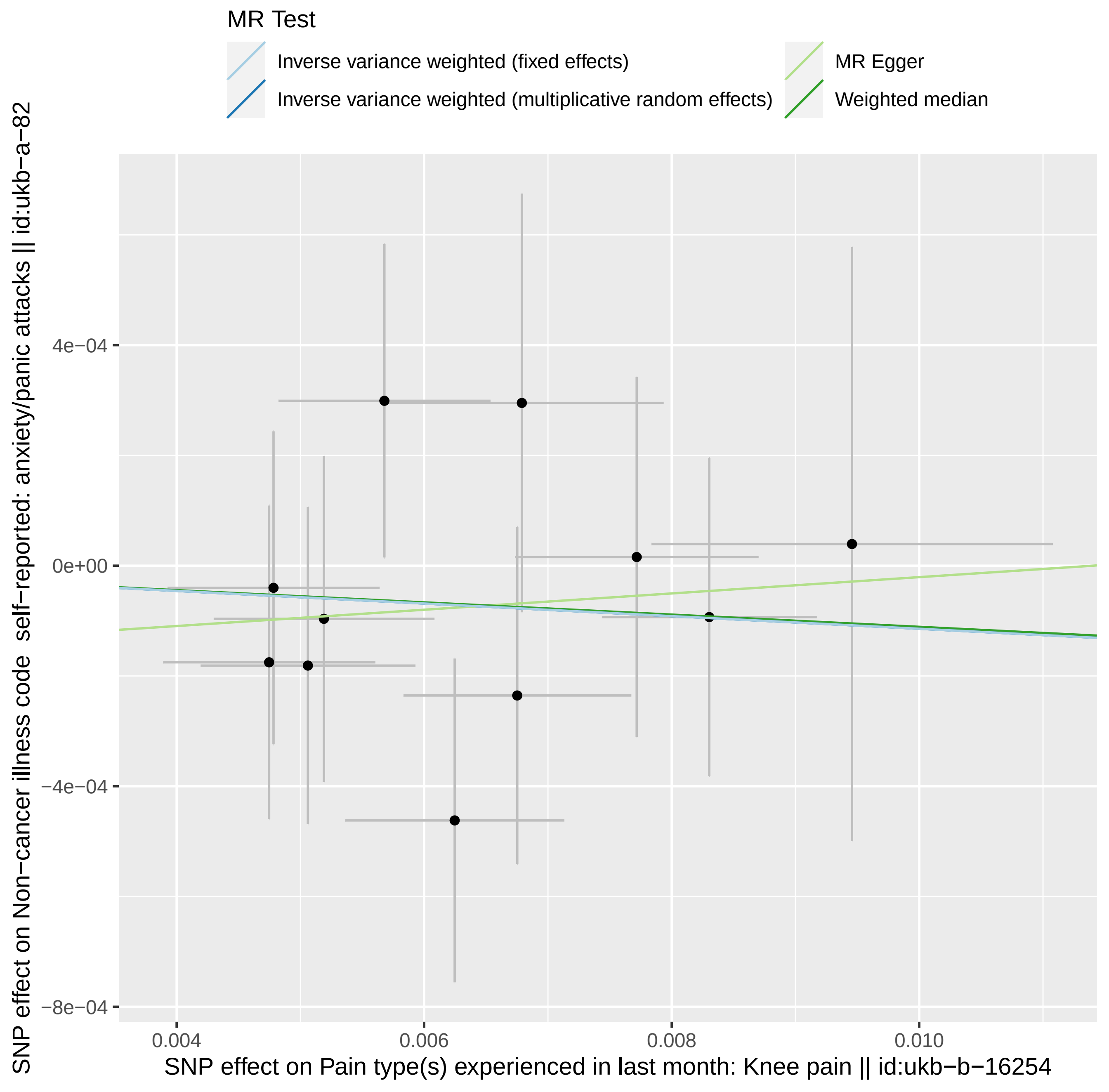
MR Test
Inverse variance weighted (fixed effects)
Inverse variance weighted (multiplicative random effects)
MR Egger
Weighted median
SNP effect on Non−cancer illness code  self−reported: anxiety/panic attacks || id:ukb−a−82
4e−04
0e+00
−4e−04
−8e−04
0.004
0.006
0.008
0.010
SNP effect on Pain type(s) experienced in last month: Knee pain || id:ukb−b−16254

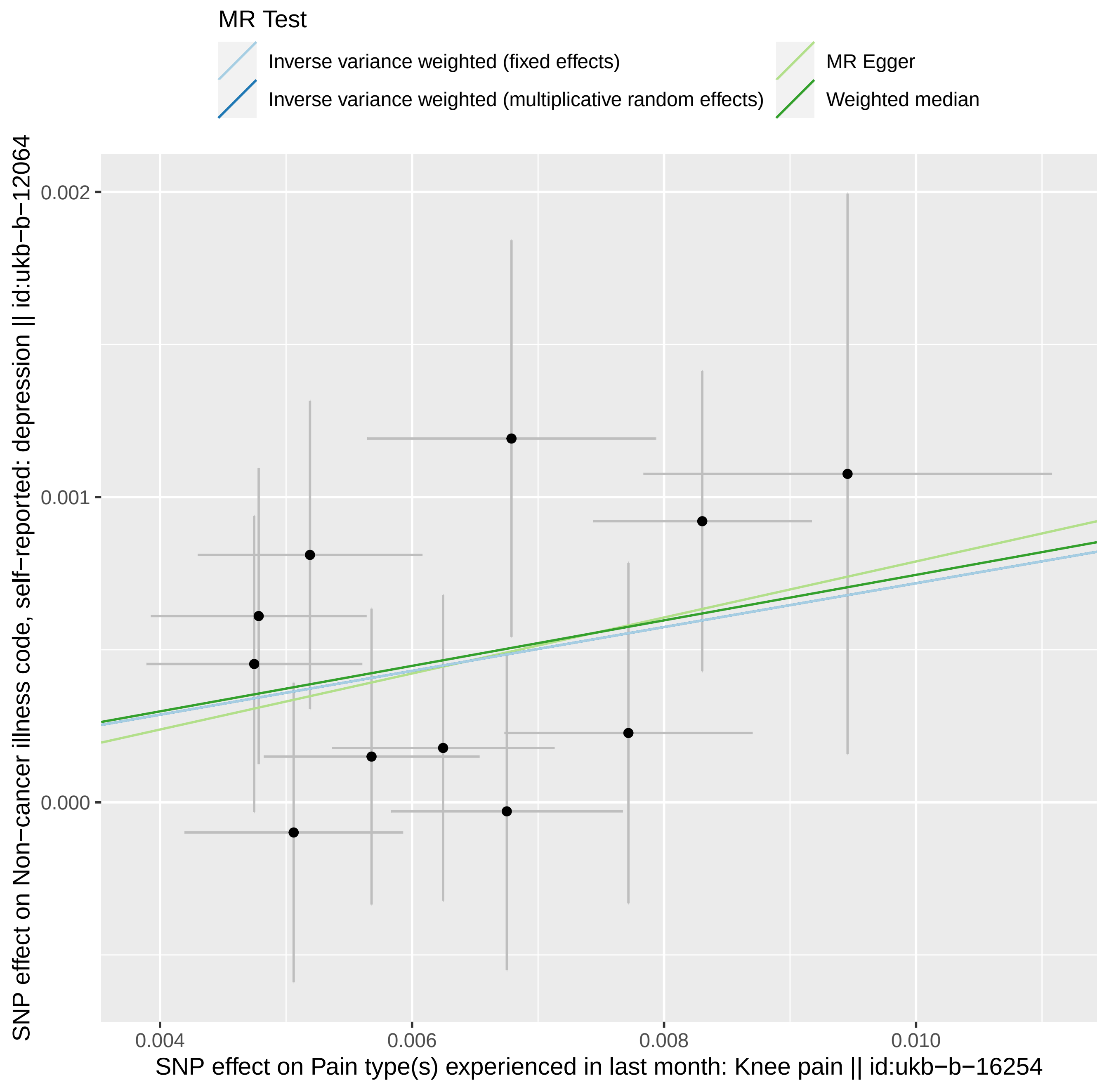
MR Test
Inverse variance weighted (fixed effects)
Inverse variance weighted (multiplicative random effects)
MR Egger
Weighted median
0.002
0.001
0.000
0.004
0.006
0.008
0.010
SNP effect on Non−cancer illness code, self−reported: depression || id:ukb−b−12064
SNP effect on Pain type(s) experienced in last month: Knee pain || id:ukb−b−16254

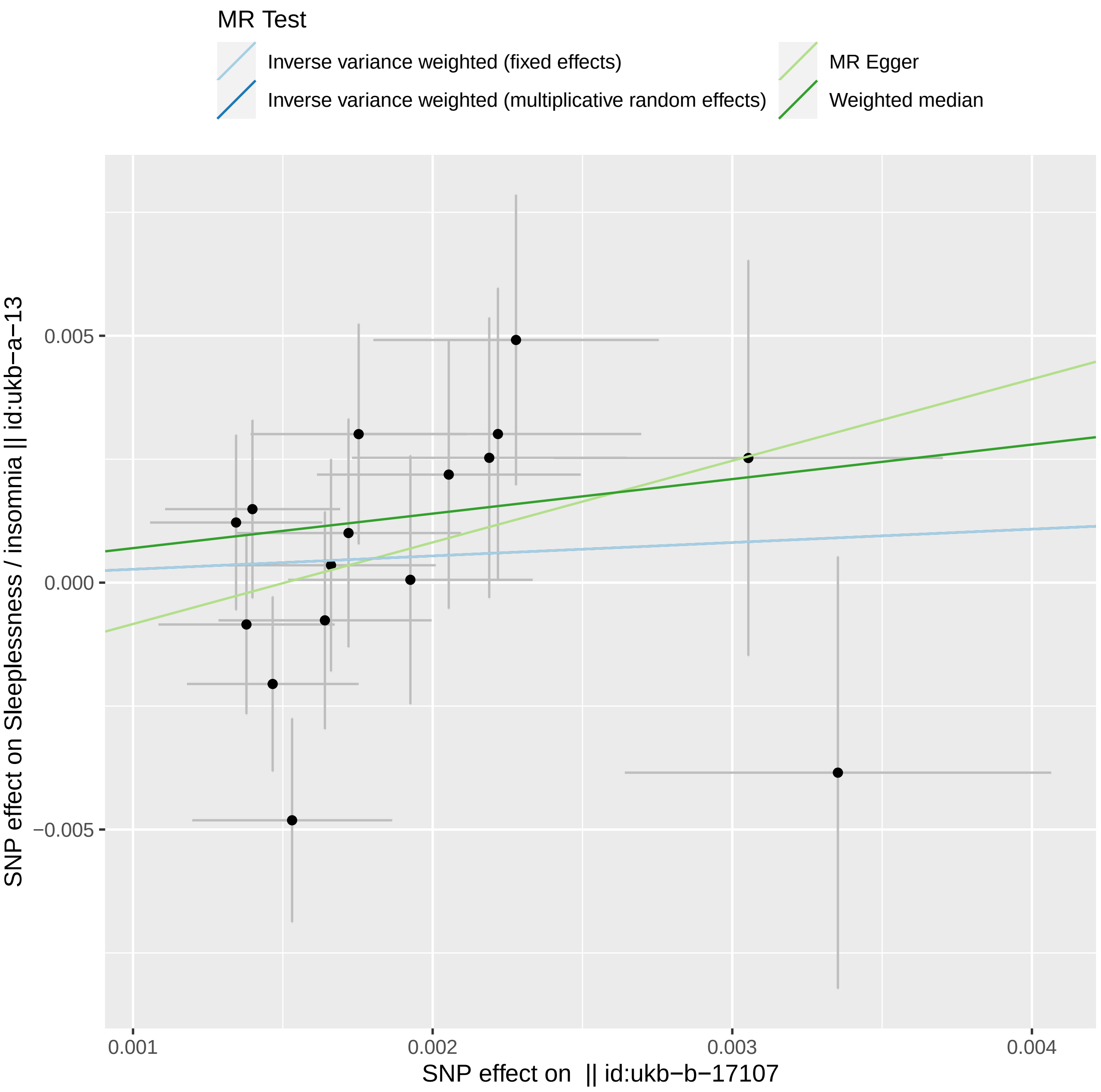

MR Test
Inverse variance weighted (fixed effects)
Inverse variance weighted (multiplicative random effects)
MR Egger
Weighted median
0.005
0.000
−0.005
0.001
0.002
0.003
0.004
SNP effect on Sleeplessness / insomnia || id:ukb−a−13
SNP effect on || id:ukb−b−17107

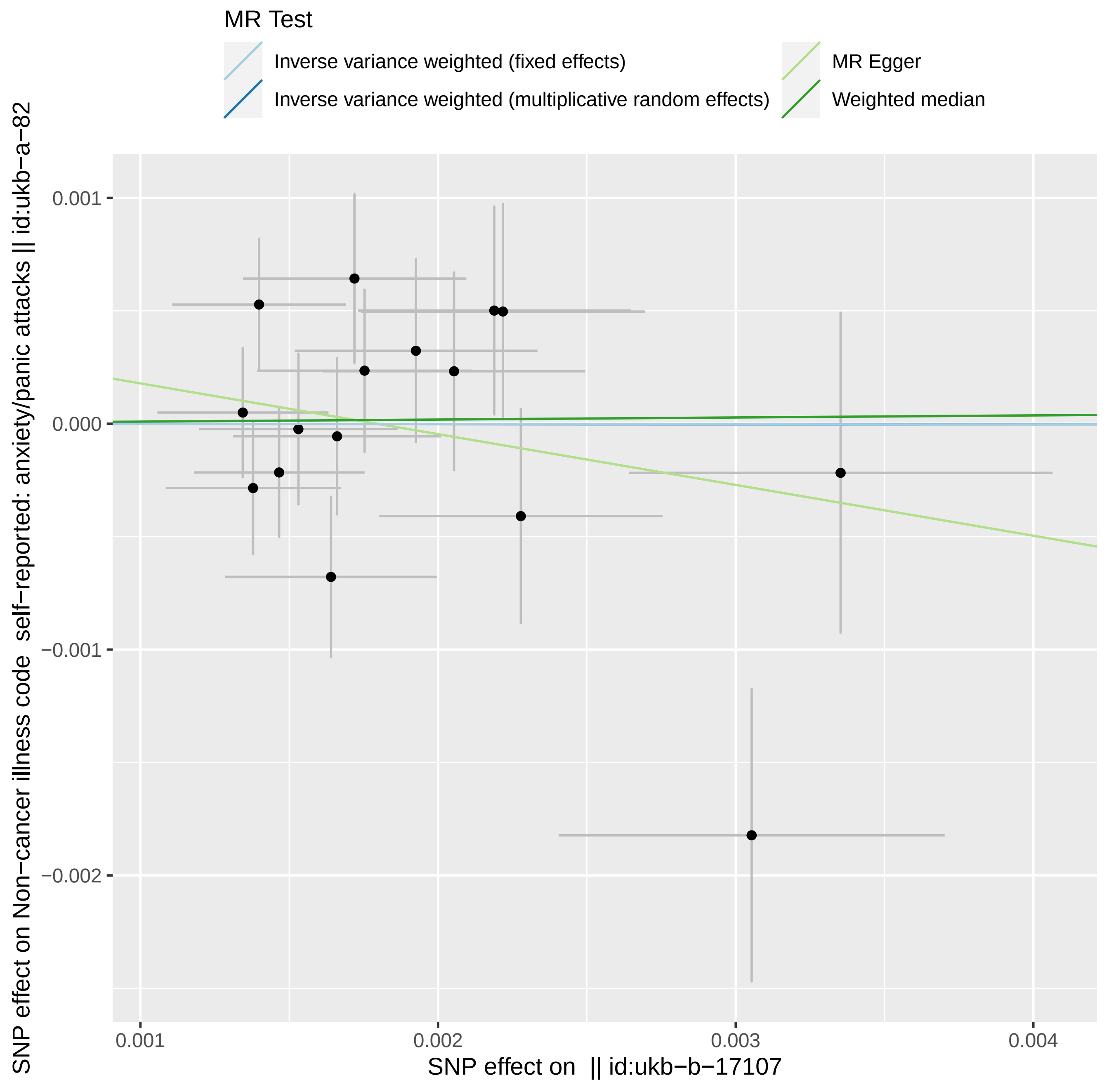

MR Test
Inverse variance weighted (fixed effects)
Inverse variance weighted (multiplicative random effects)
MR Egger
Weighted median
SNP effect on Non−cancer illness code self−reported: anxiety/panic attacks || id:ukb−a−82
0.001
0.000
−0.001
−0.002
0.001
0.002
0.003
0.004
SNP effect on || id:ukb−b−17107

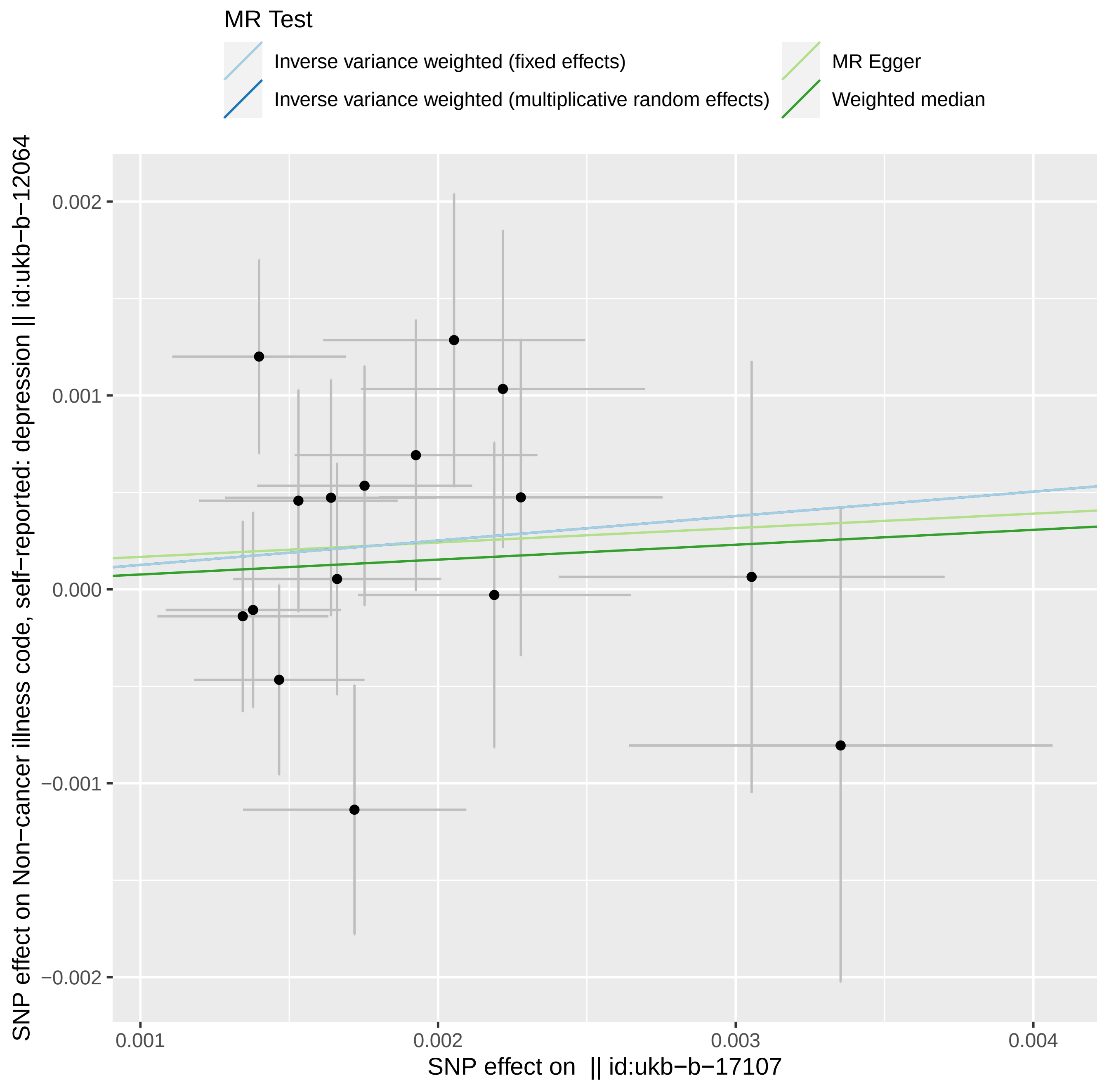
MR Test
Inverse variance weighted (fixed effects)
Inverse variance weighted (multiplicative random effects)
MR Egger
Weighted median
SNP effect on Non−cancer illness code, self−reported: depression || id:ukb−b−12064
0.002
0.001
0.000
−0.001
−0.002
0.001
0.002
0.003
0.004
SNP effect on  || id:ukb−b−17107

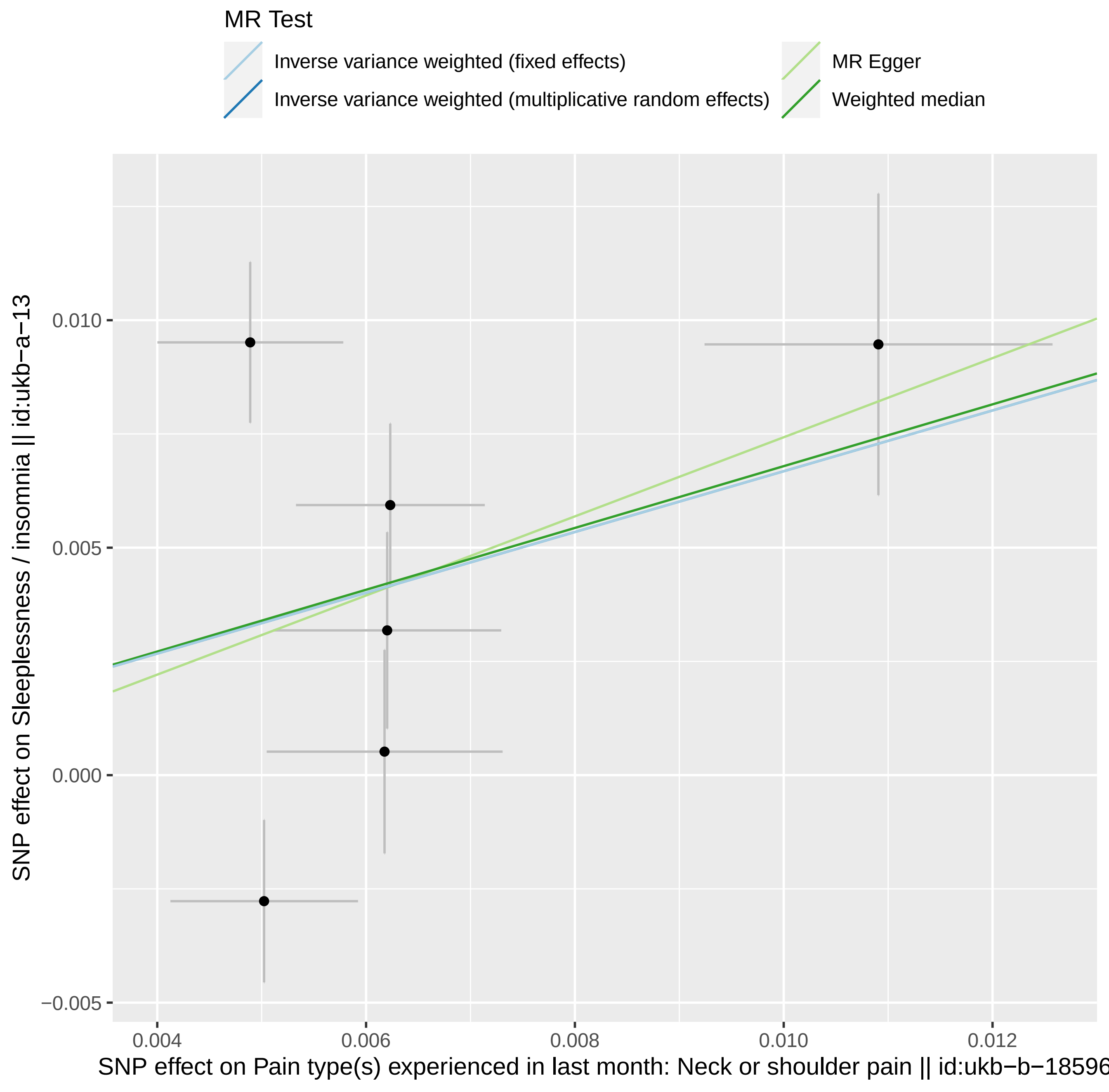
MR Test
Inverse variance weighted (fixed effects)
Inverse variance weighted (multiplicative random effects)
MR Egger
Weighted median
0.010
0.005
0.000
−0.005
0.004
0.006
0.008
0.010
0.012
SNP effect on Sleeplessness / insomnia || id:ukb−a−13
SNP effect on Pain type(s) experienced in last month: Neck or shoulder pain || id:ukb−b−18596

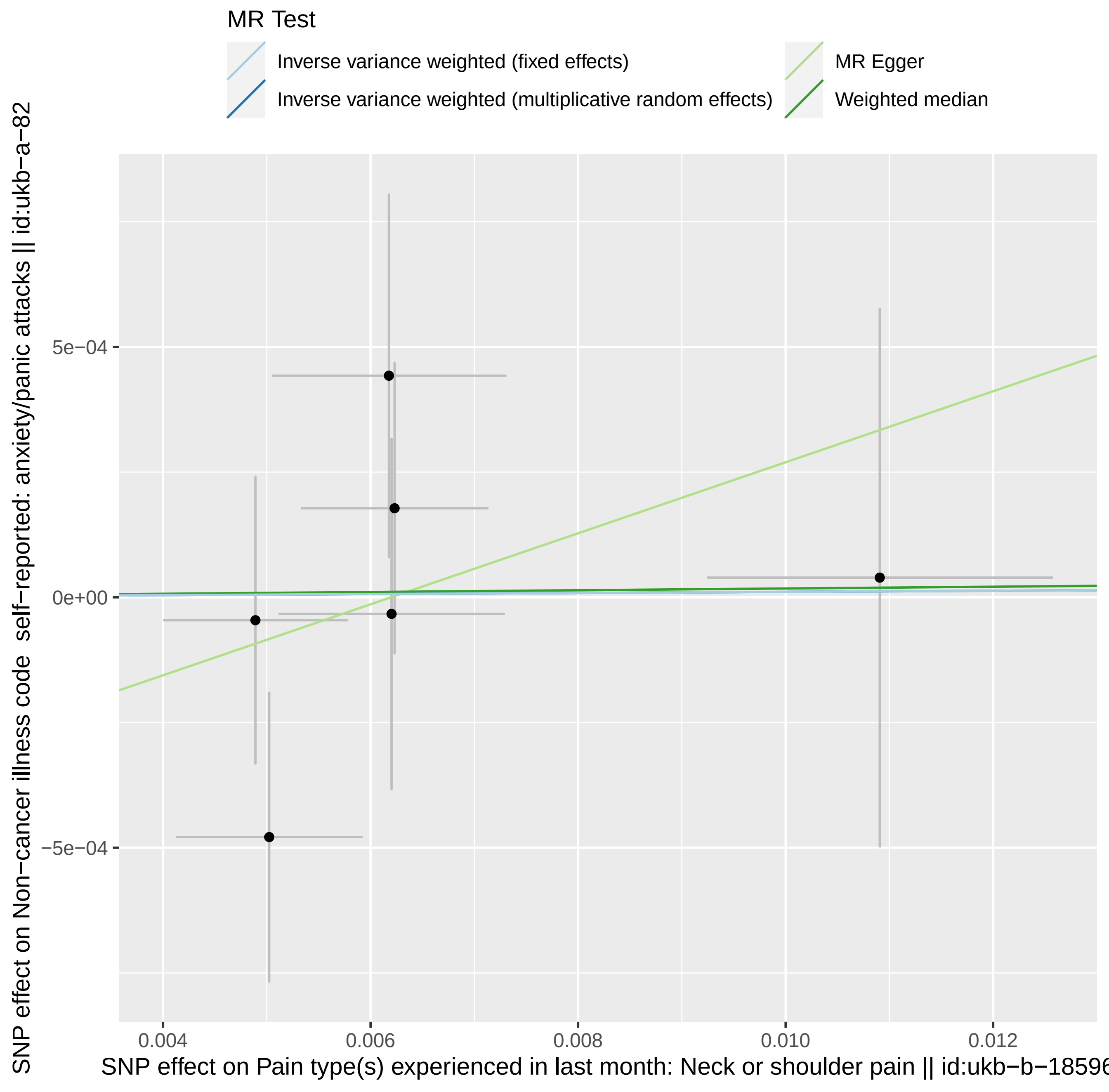
MR Test
Inverse variance weighted (fixed effects)
Inverse variance weighted (multiplicative random effects)
MR Egger
Weighted median
5e−04
0e+00
−5e−04
0.004
0.006
0.008
0.010
0.012
SNP effect on Non−cancer illness code self−reported: anxiety/panic attacks || id:ukb−a−82
SNP effect on Pain type(s) experienced in last month: Neck or shoulder pain || id:ukb−b−18596

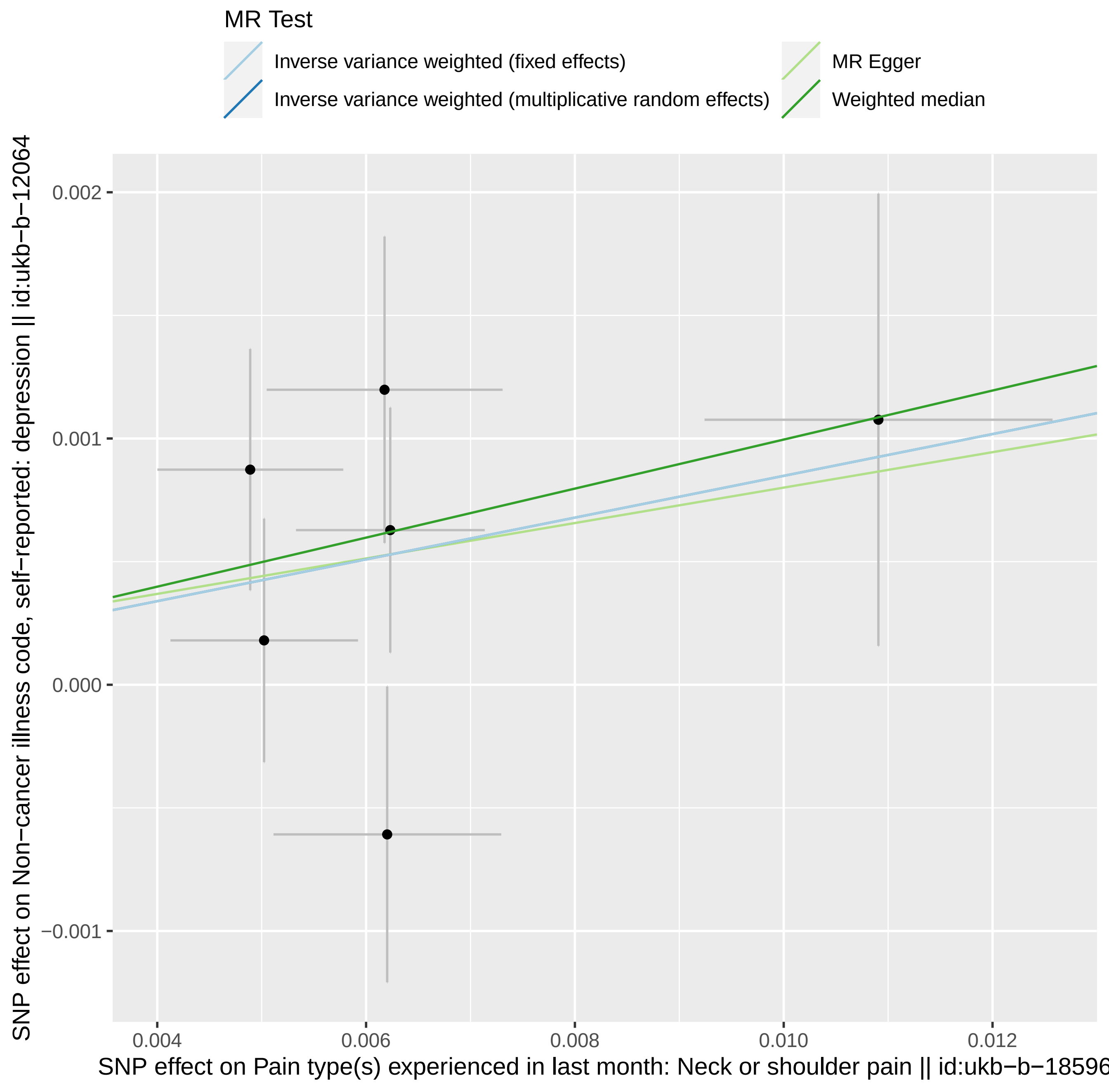

MR Test
Inverse variance weighted (fixed effects)
Inverse variance weighted (multiplicative random effects)
MR Egger
Weighted median
SNP effect on Non−cancer illness code, self−reported: depression || id:ukb−b−12064
0.002
0.001
0.000
−0.001
0.004
0.006
0.008
0.010
0.012
SNP effect on Pain type(s) experienced in last month: Neck or shoulder pain || id:ukb−b−18596

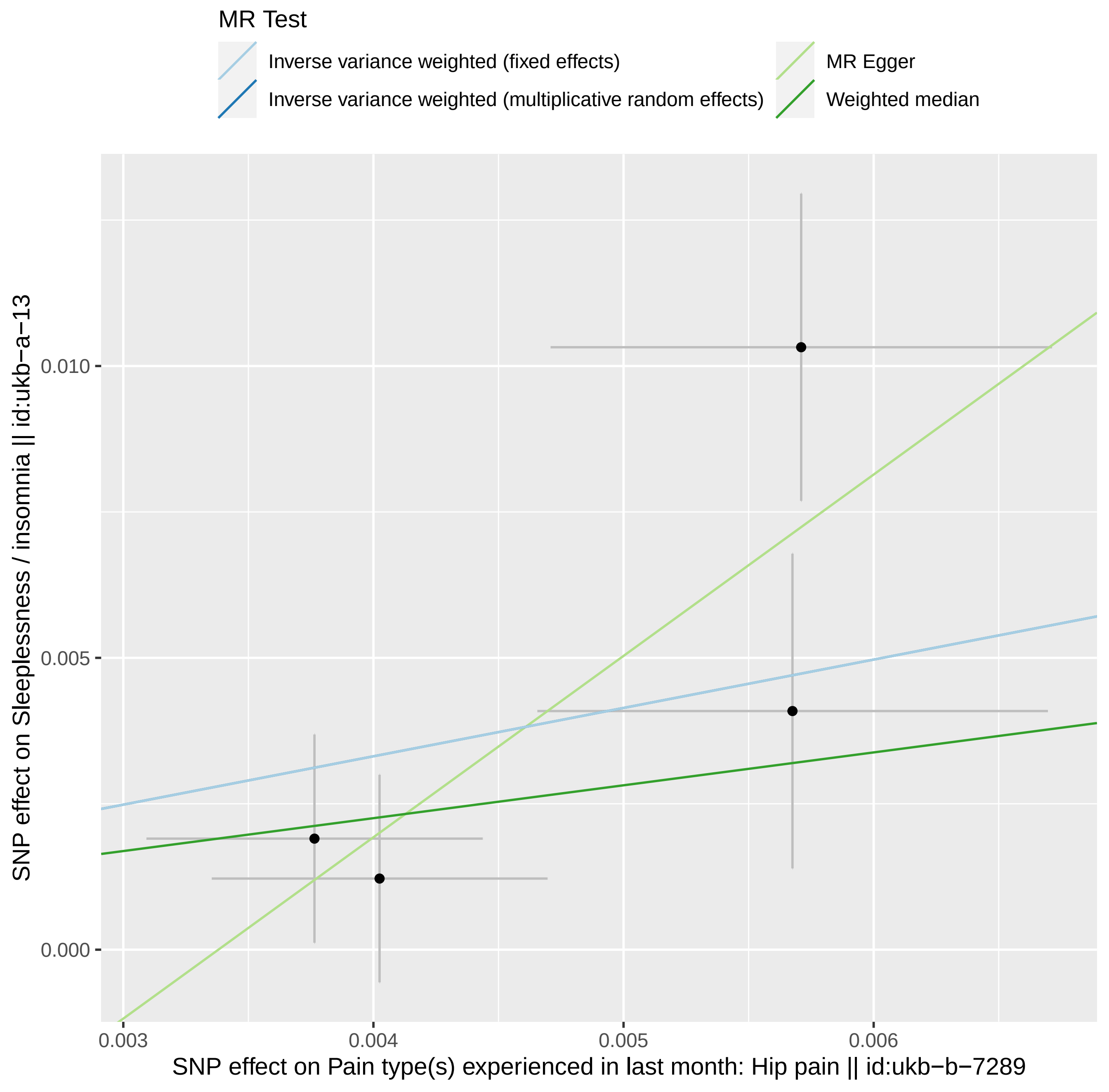
MR Test
Inverse variance weighted (fixed effects)
Inverse variance weighted (multiplicative random effects)
MR Egger
Weighted median
SNP effect on Sleeplessness / insomnia || id:ukb−a−13
0.010
0.005
0.000
0.003
0.004
0.005
0.006
SNP effect on Pain type(s) experienced in last month: Hip pain || id:ukb−b−7289

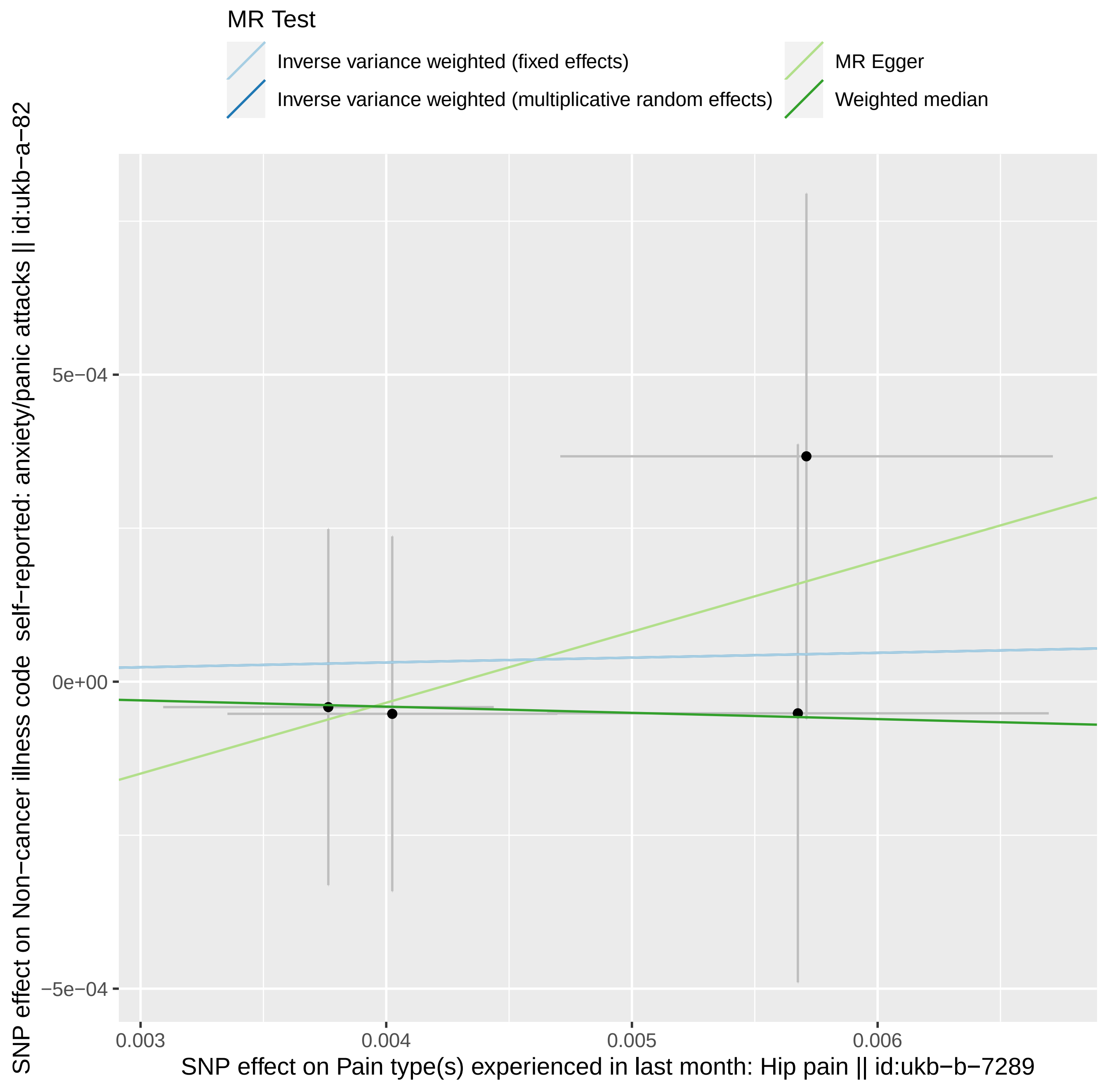

MR Test
Inverse variance weighted (fixed effects)
Inverse variance weighted (multiplicative random effects)
MR Egger
Weighted median
5e−04
0e+00
−5e−04
0.003
0.004
0.005
0.006
SNP effect on Non−cancer illness code  self−reported: anxiety/panic attacks || id:ukb−a−82
SNP effect on Pain type(s) experienced in last month: Hip pain || id:ukb−b−7289

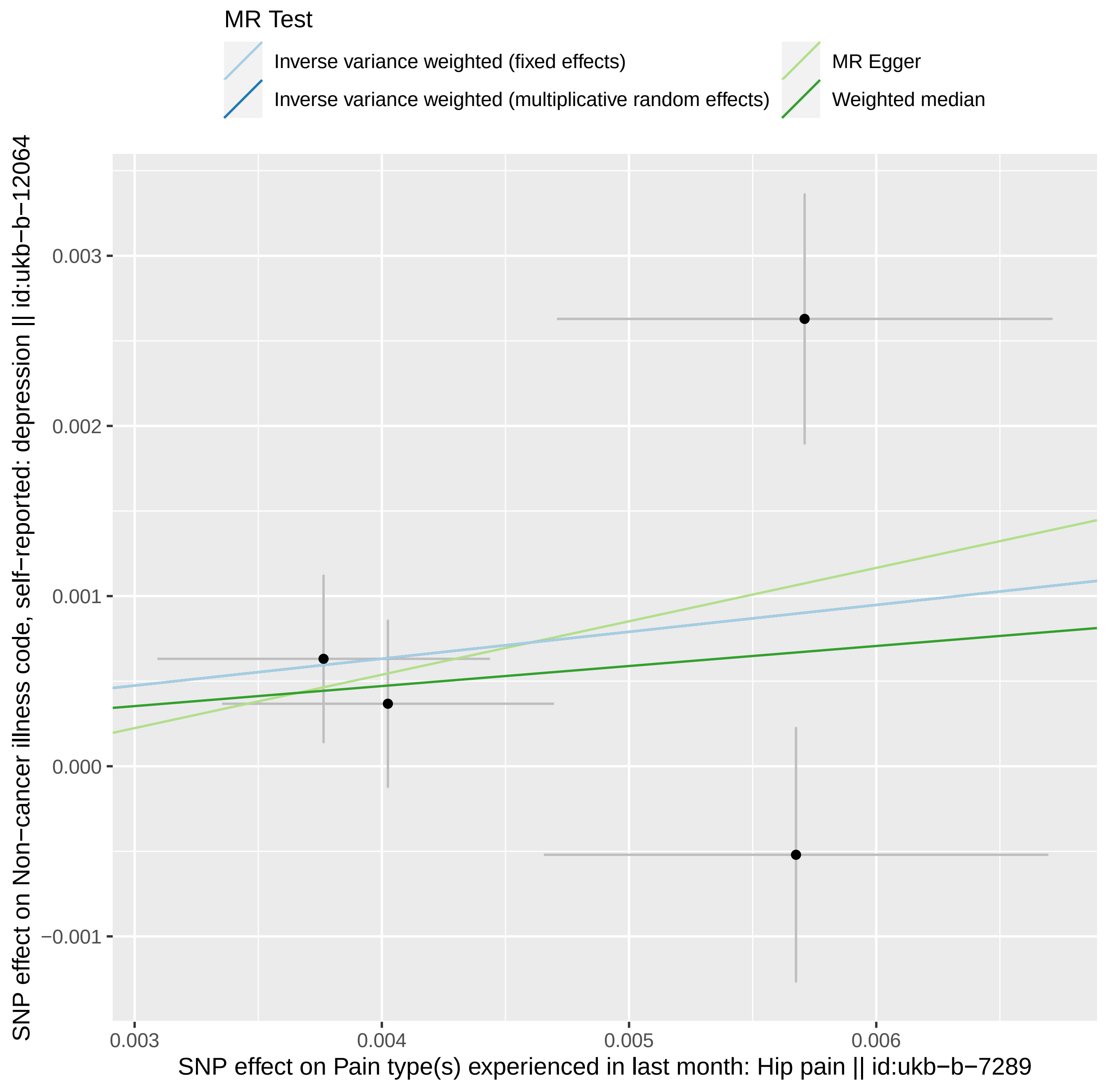
MR Test
Inverse variance weighted (fixed effects)
Inverse variance weighted (multiplicative random effects)
MR Egger
Weighted median
SNP effect on Non−cancer illness code, self−reported: depression || id:ukb−b−12064
0.003
0.002
0.001
0.000
−0.001
0.003
0.004
0.005
0.006
SNP effect on Pain type(s) experienced in last month: Hip pain || id:ukb−b−7289

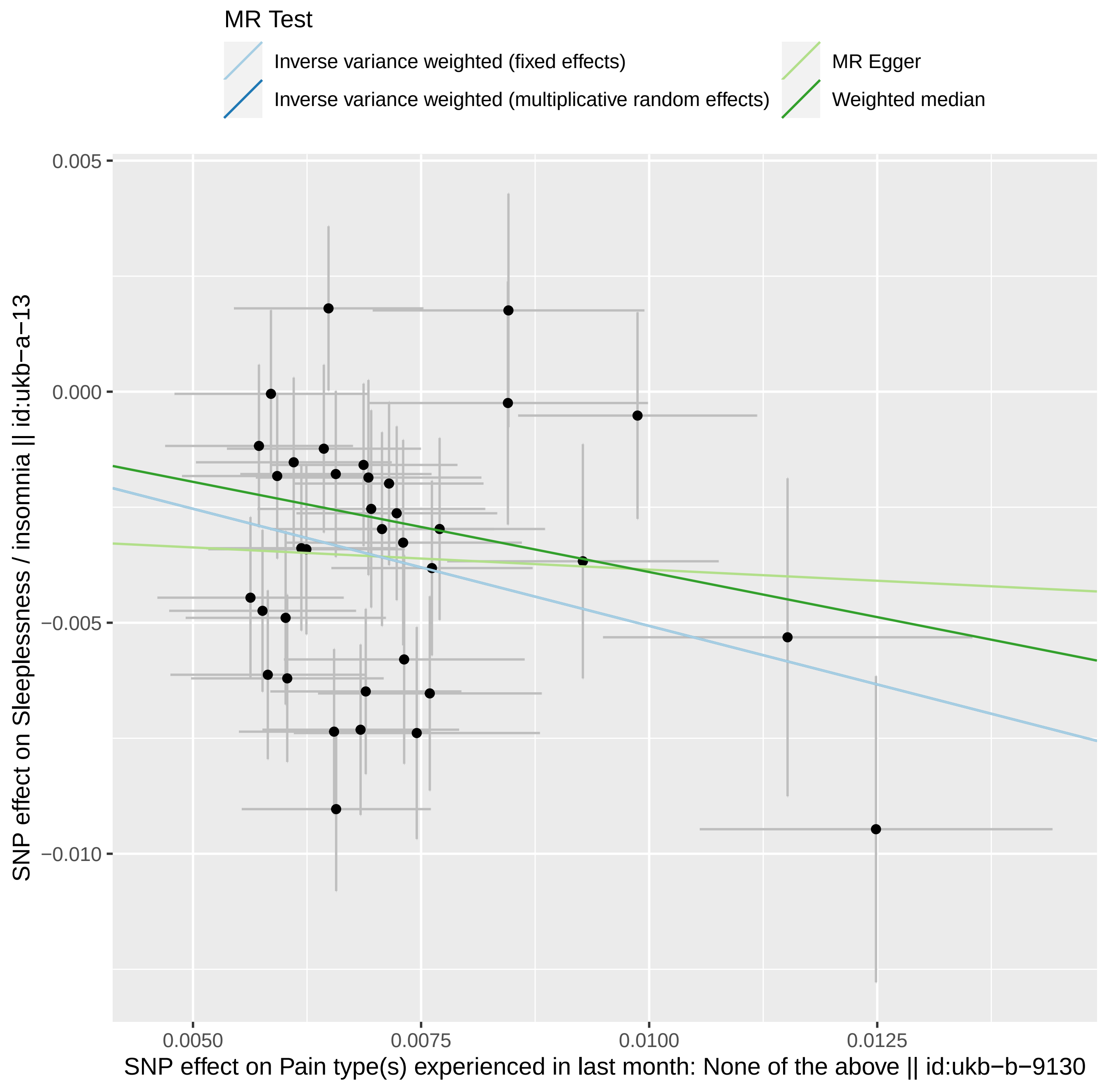
MR Test
Inverse variance weighted (fixed effects)
Inverse variance weighted (multiplicative random effects)
MR Egger
Weighted median
0.005
0.000
−0.005
−0.010
0.0050
0.0075
0.0100
0.0125
SNP effect on Sleeplessness / insomnia || id:ukb−a−13
SNP effect on Pain type(s) experienced in last month: None of the above || id:ukb−b−9130

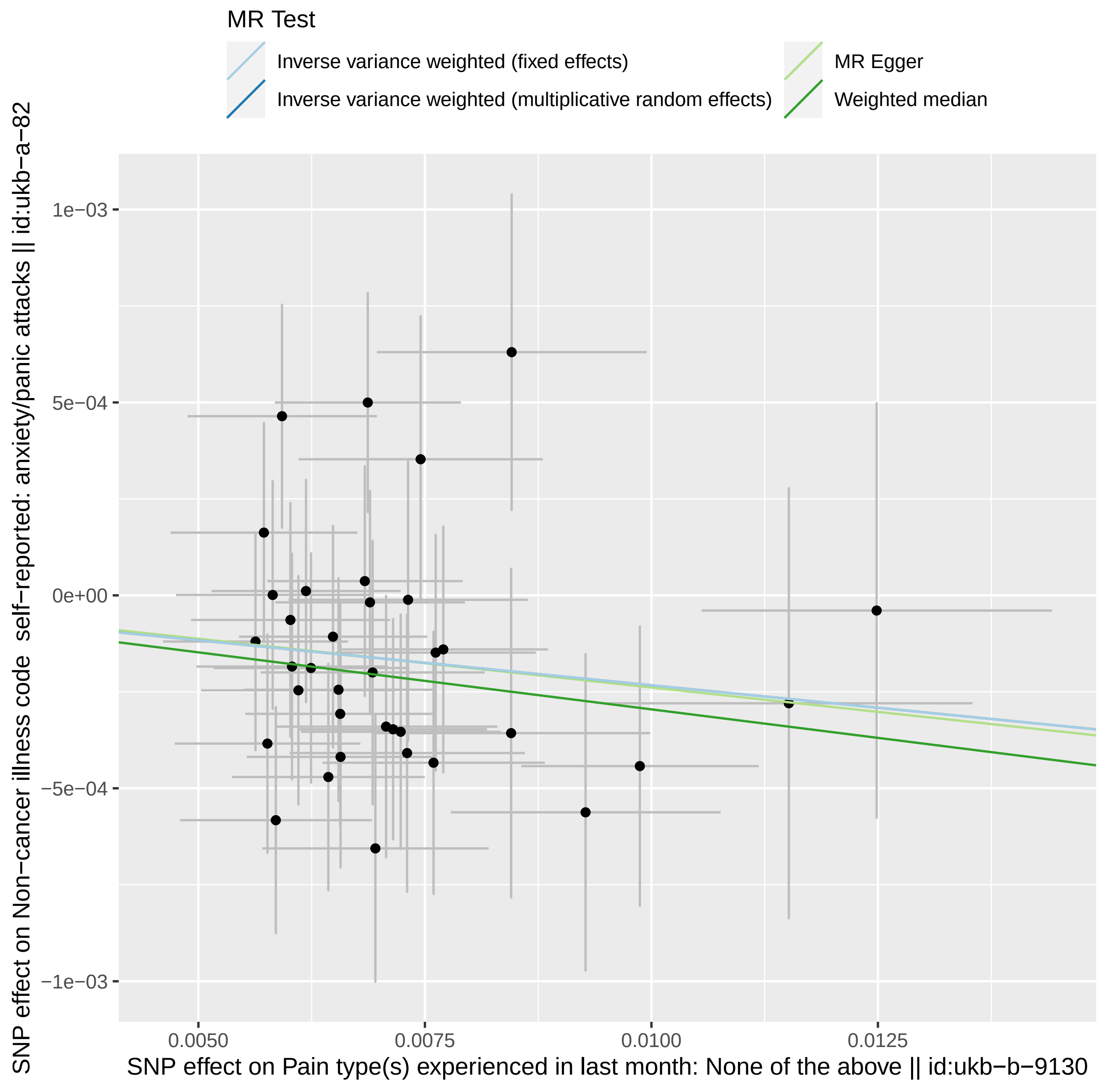
MR Test
Inverse variance weighted (fixed effects)
Inverse variance weighted (multiplicative random effects)
MR Egger
Weighted median
1e−03
5e−04
0e+00
−5e−04
−1e−03
0.0050
0.0075
0.0100
0.0125
SNP effect on Pain type(s) experienced in last month: None of the above || id:ukb−b−9130
SNP effect on Non−cancer illness code self−reported: anxiety/panic attacks || id:ukb−a−82

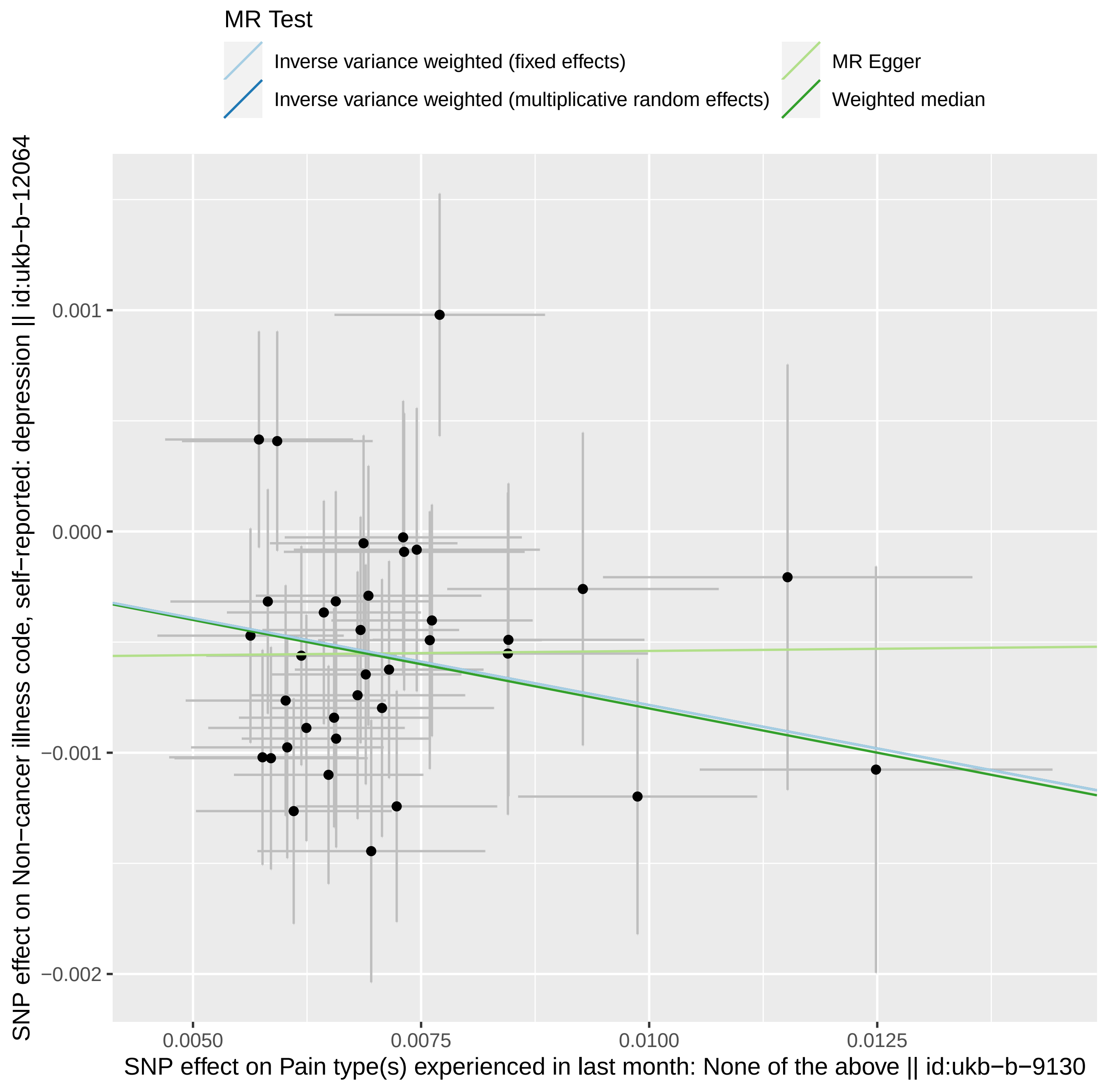
MR Test
Inverse variance weighted (fixed effects)
Inverse variance weighted (multiplicative random effects)
MR Egger
Weighted median
0.001
0.000
−0.001
−0.002
0.0050
0.0075
0.0100
0.0125
SNP effect on Non−cancer illness code, self−reported: depression || id:ukb−b−12064
SNP effect on Pain type(s) experienced in last month: None of the above || id:ukb−b−9130

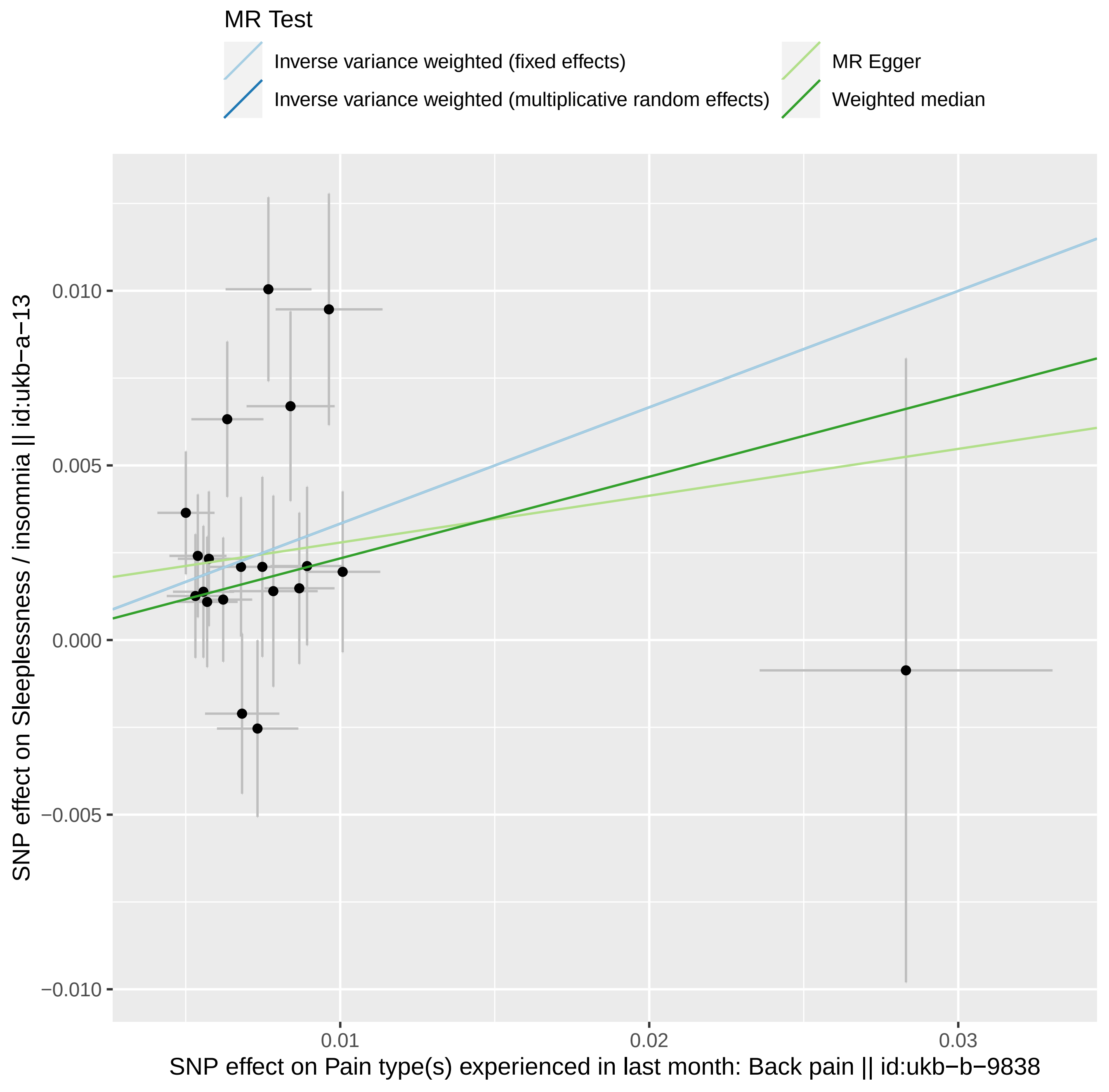
MR Test
Inverse variance weighted (fixed effects)
Inverse variance weighted (multiplicative random effects)
MR Egger
Weighted median
SNP effect on Sleeplessness / insomnia || id:ukb−a−13
0.010
0.005
0.000
−0.005
−0.010
0.01
0.02
0.03
SNP effect on Pain type(s) experienced in last month: Back pain || id:ukb−b−9838
