# Supplementary material for: Exploring the bidirectional relationship between pain and mental disorders: a comprehensive Mendelian randomization study
Source: J Headache Pain. 2023 Jul 7;24(1):82. doi: 10.1186/s10194-023-01612-2 (PMC10326936; doi:10.1186/s10194-023-01612-2)
Supplement: Supplementary file 4 — Additional file 4: Supplementary file 4. MR Results of sleeplessness/insomnia, anxiety/panic attacks and depression on the risk of localized pain with scatter plots. [file 10194_2023_1612_MOESM4_ESM.pdf]

Supplementary file 4

MR Results of sleeplessness/insomnia, anxiety/panic attacks and depression on the risk of localized pain with scatter plots

Single-Variable MR Results of sleeplessness/insomnia, anxiety/panic attacks and depression on the risk of localized pain

		Sleeplessness / insomnia			Anxiety/panic attacks*			Depression*		
Outcome	Method	N SNV	OR (95%CI)	*P* value	N SNV	OR (95%CI)	*P* value	N SNV	OR (95%CI)	*P* value
Headache	IVW (FEM)	27	1.09 (1.06-1.12)	<0.001	12	1.98 (1.41-2.78)	<0.001	32	1.28 (1.13-1.45)	<0.001
	IVW (REM)	27	1.09 (1.03-1.15)	0.002	12	1.98 (1.40-2.81)	<0.001	32	1.28 (1.08-1.52)	0.004
	Weighted median	27	1.07 (1.02-1.13)	0.005	12	2.31 (1.44-3.69)	<0.001	32	1.21 (1.00-1.46)	0.05
	MR Egger	27	1.00 (0.84-1.19)	0.99	12	0.65 (0.28-1.55)	0.36	32	1.58 (0.95-2.65)	0.09
Facial pain	IVW (FEM)	26	1.01 (1.00-1.02)	0.02	8	1.12 (0.97-1.28)	0.12	25	1.07 (1.02-1.12)	0.006
	IVW (REM)	26	1.01 (1.00-1.02)	0.03	8	1.12 (0.98-1.27)	0.09	25	1.07 (1.03-1.11)	<0.001
	Weighted median	26	1.01 (1.00-1.03)	0.09	8	1.11 (0.92-1.33)	0.29	25	1.05 (0.99-1.12)	0.12
	MR Egger	26	1.03 (0.99-1.07)	0.13	8	0.89 (0.49-1.60)	0.70	25	1.28 (1.00-1.65)	0.07
Neck or shoulder pain	IVW (FEM)	27	1.12 (1.08-1.15)	<0.001	12	1.83 (1.28-2.62)	<0.001	32	1.32 (1.16-1.50)	<0.001
	IVW (REM)	27	1.12 (1.07-1.16)	<0.001	12	1.83 (1.28-2.62)	<0.001	32	1.32 (1.14-1.51)	<0.001
	Weighted median	27	1.07 (1.02-1.12)	0.005	12	1.78 (1.08-2.93)	0.25	32	1.25 (1.04-1.50)	0.02
	MR Egger	27	1.05 (0.93-1.18)	0.42	12	1.34 (0.53-3.42)	0.55	32	1.52 (0.99-2.31)	0.06
Back pain	IVW (FEM)	27	1.12 (1.09-1.16)	<0.001	12	1.54 (1.20-1.97)	0.02	32	1.35 (1.18-1.55)	<0.001
	IVW (REM)	27	1.12 (1.07-1.18)	<0.001	12	1.54 (1.06-2.23)	<0.001	32	1.35 (1.10-1.66)	0.004
	Weighted median	27	1.11 (1.05-1.17)	<0.001	12	1.64 (1.01-2.68)	0.05	32	1.30 (1.04-1.62)	0.02
	MR Egger	27	1.14 (0.97-1.33)	0.13	12	1.23 (0.48-3.16)	0.68	32	1.75 (0.94-3.32)	0.09
Stomach and abdominal pain	IVW (FEM)	27	1.06 (1.04-1.08)	<0.001	12	0.97 (0.76-1.23)	0.80	32	1.14 (1.05-1.25)	0.002
	IVW (REM)	27	1.06 (1.03-1.08)	<0.001	12	0.97 (0.78-1.20)	0.78	32	1.14 (1.04-1.25)	0.004
	Weighted median	27	1.04 (1.01-1.07)	0.006	12	1.05 (0.76-1.45)	0.77	32	1.16 (1.03-1.32)	0.02
	MR Egger	27	1.01 (0.97-1.07)	0.76	12	1.27 (0.69-2.32)	0.46	32	0.95 (0.73-1.25)	0.74
Hip pain	IVW (FEM)	27	1.08 (1.05-1.10)	<0.001	12	1.20 (0.91-1.57)	0.19	32	1.17 (1.06-1.29)	0.002
	IVW (REM)	27	1.08 (1.05-1.11)	<0.001	12	1.20 (0.80-1.80)	0.39	32	1.17 (1.04-1.31)	0.01
	Weighted median	27	1.07 (1.03-1.10)	<0.001	12	1.26 (0.83-1.92)	0.27	32	1.14 (0.98-1.31)	0.08
	MR Egger	27	1.05 (0.96-1.15)	0.31	12	0.67 (0.24-1.85)	0.46	32	0.98 (0.68-1.40)	0.90
Knee pain	IVW (FEM)	27	1.09 (1.06-1.12)	<0.001	12	1.14 (0.80-1.62)	0.46	32	1.16 (1.02-1.32)	0.02
	IVW (REM)	27	1.09 (1.04-1.13)	<0.001	12	1.14 (0.78-1.66)	0.49	32	1.16 (0.99-1.36)	0.06
	Weighted median	27	1.05 (1.00-1.10)	0.06	12	1.23 (0.76-1.98)	0.40	32	1.20 (0.99-1.46)	0.06
	MR Egger	27	0.98 (0.87-1.11)	0.78	12	0.65 (0.26-1.66)	0.39	32	1.00 (0.62-1.60)	1.00
None of above	IVW (FEM)	27	0.86 (0.83-0.89)	<0.001	12	0.55 (0.37-0.84)	0.005	32	0.71 (0.61-0.83)	<0.001
	IVW (REM)	27	0.86 (0.81-0.92)	<0.001	12	0.55 (0.36-0.86)	0.008	32	0.71 (0.58-0.87)	<0.001
	Weighted median	27	0.92 (0.86-0.97)	0.004	12	0.44 (0.25-0.77)	0.004	32	0.68 (0.54-0.85)	<0.001
	MR Egger	27	0.93 (0.77-1.13)	0.49	12	1.83 (0.64-5.28)	0.29	32	0.64 (0.35-1.18)	0.16

Abbreviations: N SNV, number of single-nucleotide variants; IVW, inverse-variance weighted; FEM, fixed effects models; REM, random effects models; OR, odds ratio; GWAS, genome-wide association studies.

Genetic instruments selected from sleeplessness/insomnia, anxiety/panic attacks and depression GWASs, selection threshold *P* less than 5×10^{-8}, pruned at linkage disequilibrium R^2 less than 0.001 (10 000 kilobase pair window).

* If the number of SNPs available for analysis is less than 3, the selection threshold *P* will be adjusted to 5×10^{-6}.

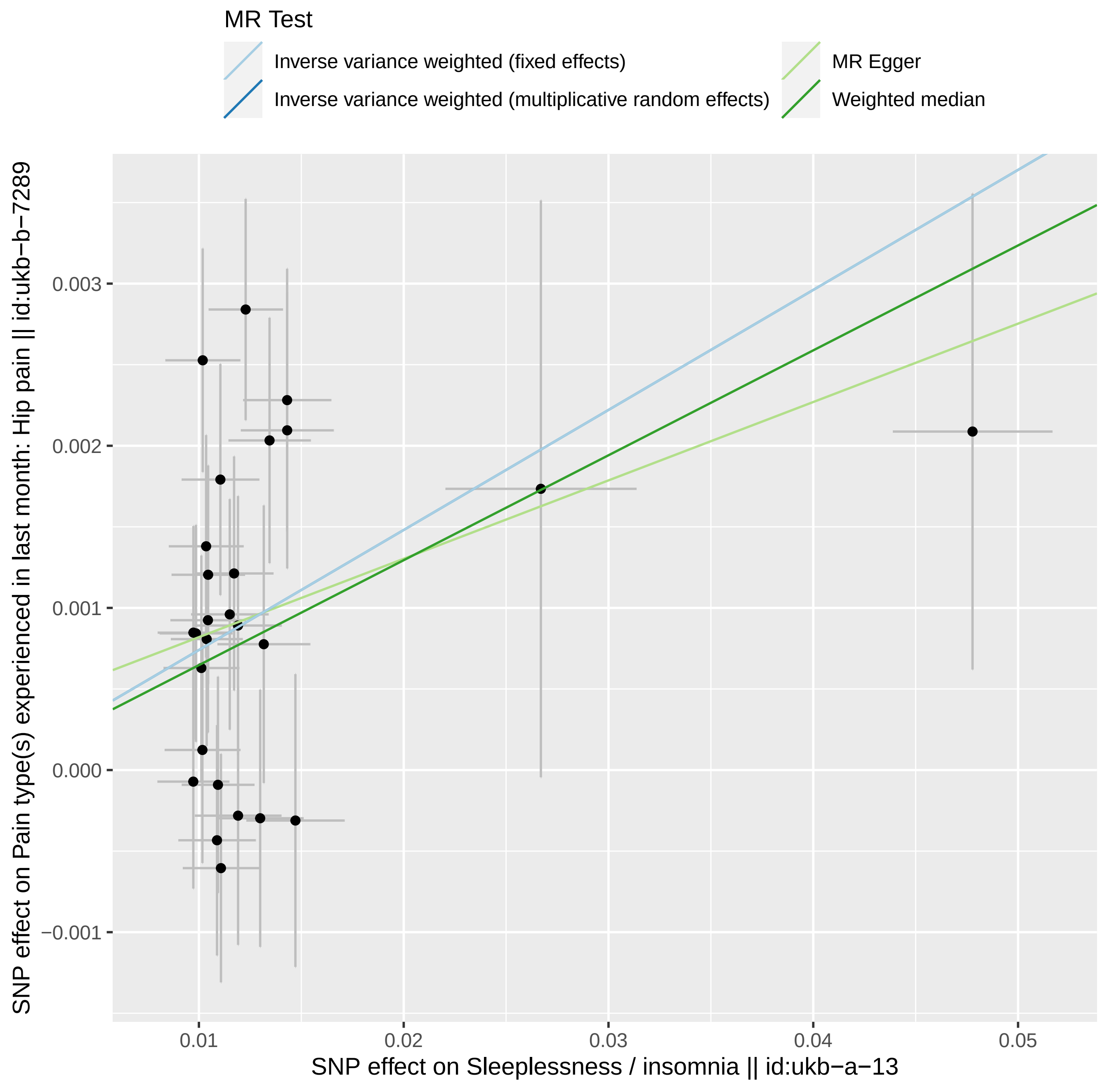
MR Test
Inverse variance weighted (fixed effects)
Inverse variance weighted (multiplicative random effects)
MR Egger
Weighted median
SNP effect on Pain type(s) experienced in last month: Hip pain || id:ukb−b−7289
0.003
0.002
0.001
0.000
−0.001
0.01
0.02
0.03
0.04
0.05
SNP effect on Sleeplessness / insomnia || id:ukb−a−13

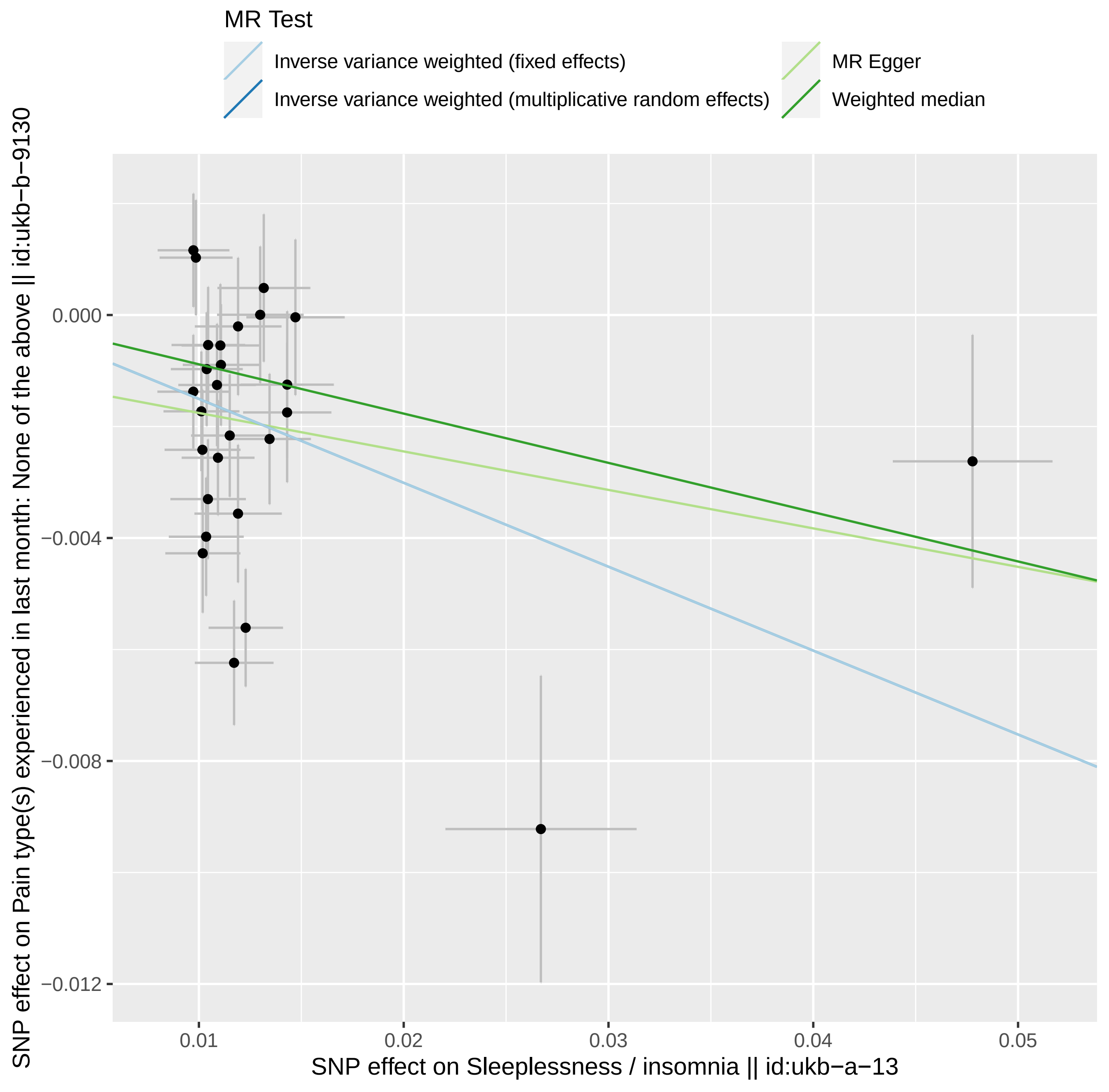

MR Test
Inverse variance weighted (fixed effects)
Inverse variance weighted (multiplicative random effects)
MR Egger
Weighted median
SNP effect on Pain type(s) experienced in last month: None of the above || id:ukb-b-9130
0.000
−0.004
−0.008
−0.012
0.01
0.02
0.03
0.04
0.05
SNP effect on Sleeplessness / insomnia || id:ukb-a-13

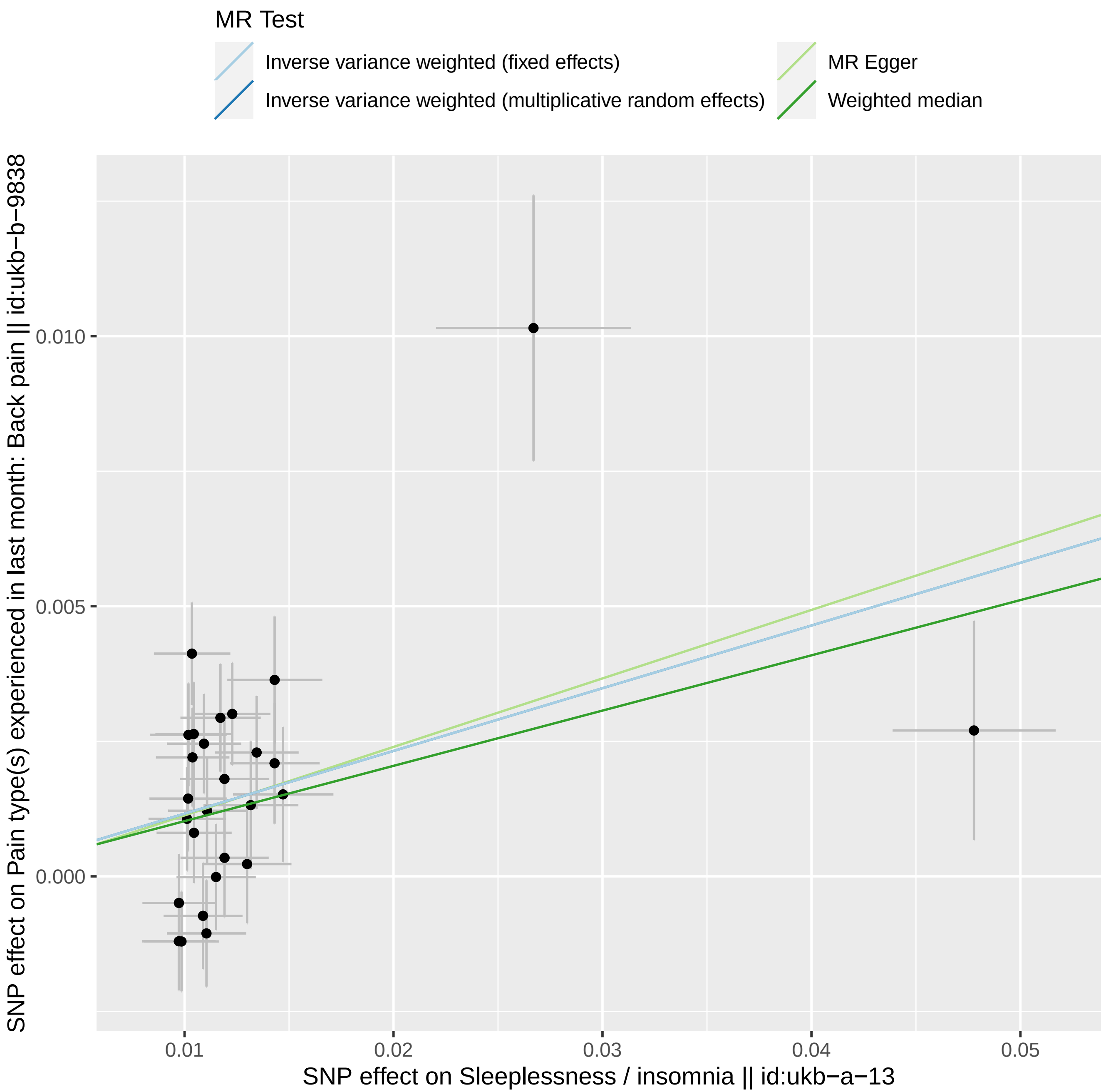
MR Test
Inverse variance weighted (fixed effects)
Inverse variance weighted (multiplicative random effects)
MR Egger
Weighted median
SNP effect on Pain type(s) experienced in last month: Back pain || id:ukb−b−9838
0.010
0.005
0.000
0.01
0.02
0.03
0.04
0.05
SNP effect on Sleeplessness / insomnia || id:ukb−a−13

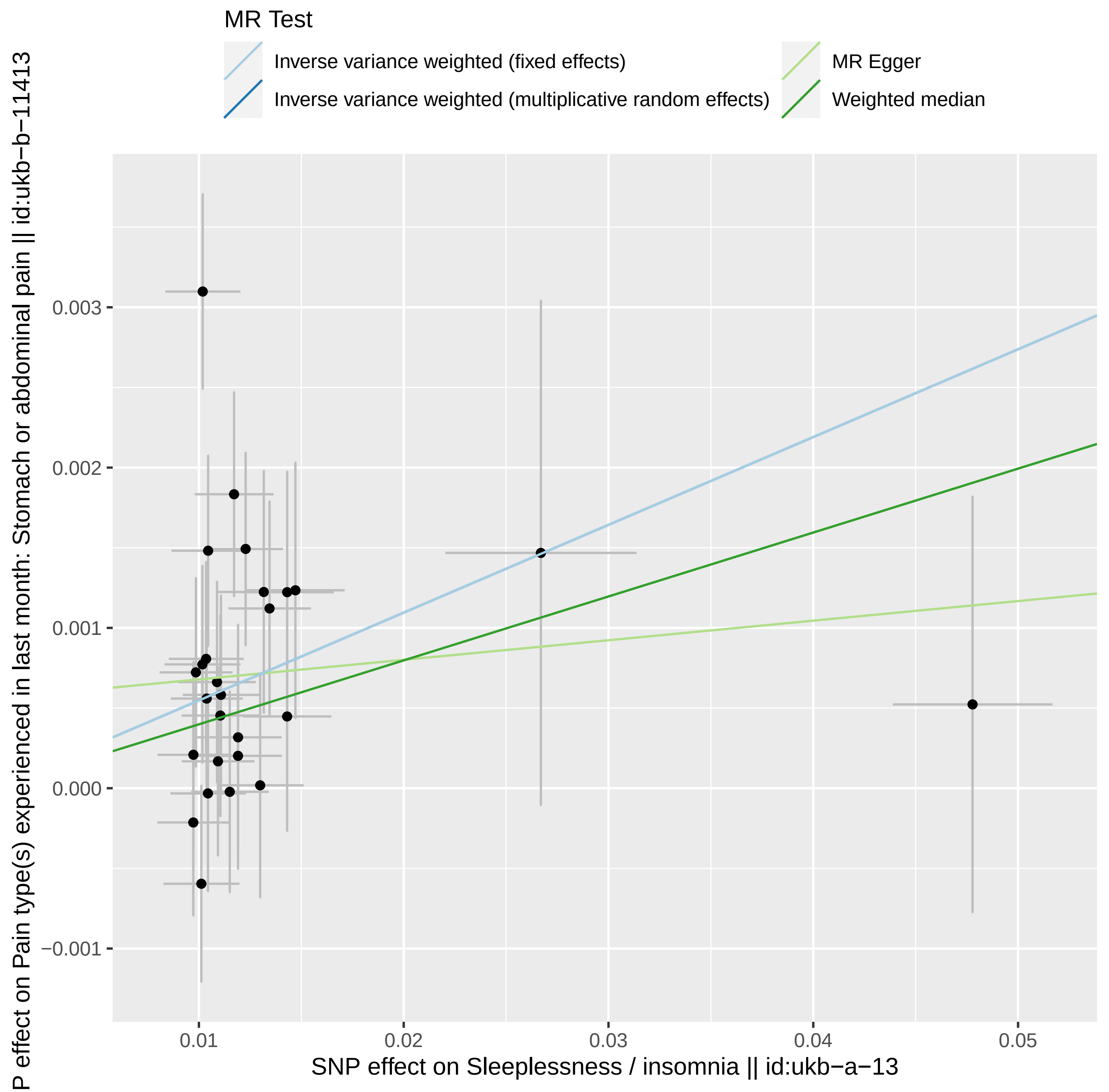

MR Test
Inverse variance weighted (fixed effects)
Inverse variance weighted (multiplicative random effects)
MR Egger
Weighted median
0.003
0.002
0.001
0.000
−0.001
0.01
0.02
0.03
0.04
0.05
SNP effect on Sleeplessness / insomnia || id:ukb−a−13
P effect on Pain type(s) experienced in last month: Stomach or abdominal pain || id:ukb−b−11413

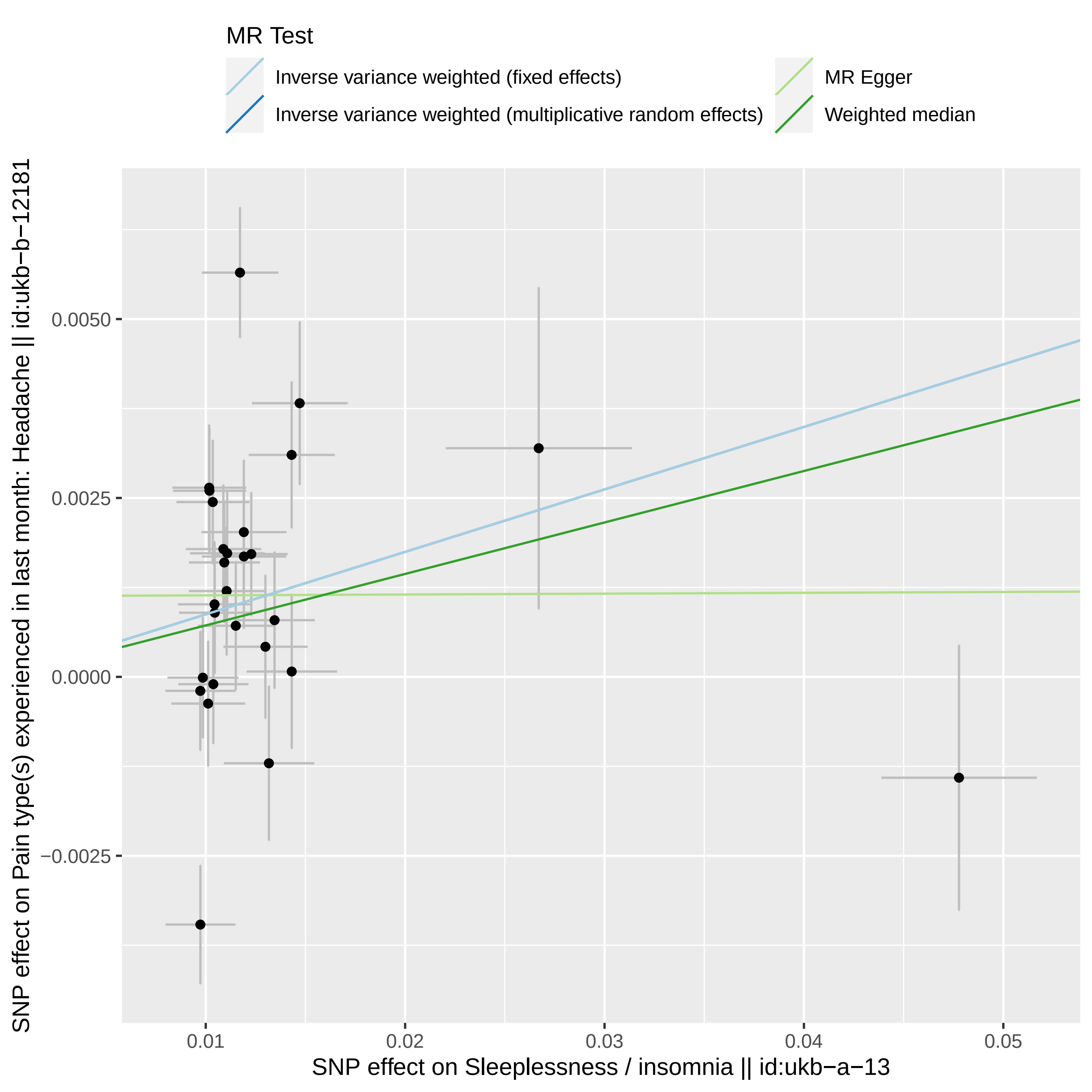
MR Test
Inverse variance weighted (fixed effects)
Inverse variance weighted (multiplicative random effects)
MR Egger
Weighted median
SNP effect on Pain type(s) experienced in last month: Headache || id:ukb−b−12181
0.0050
0.0025
0.0000
−0.0025
0.01
0.02
0.03
0.04
0.05
SNP effect on Sleeplessness / insomnia || id:ukb−a−13

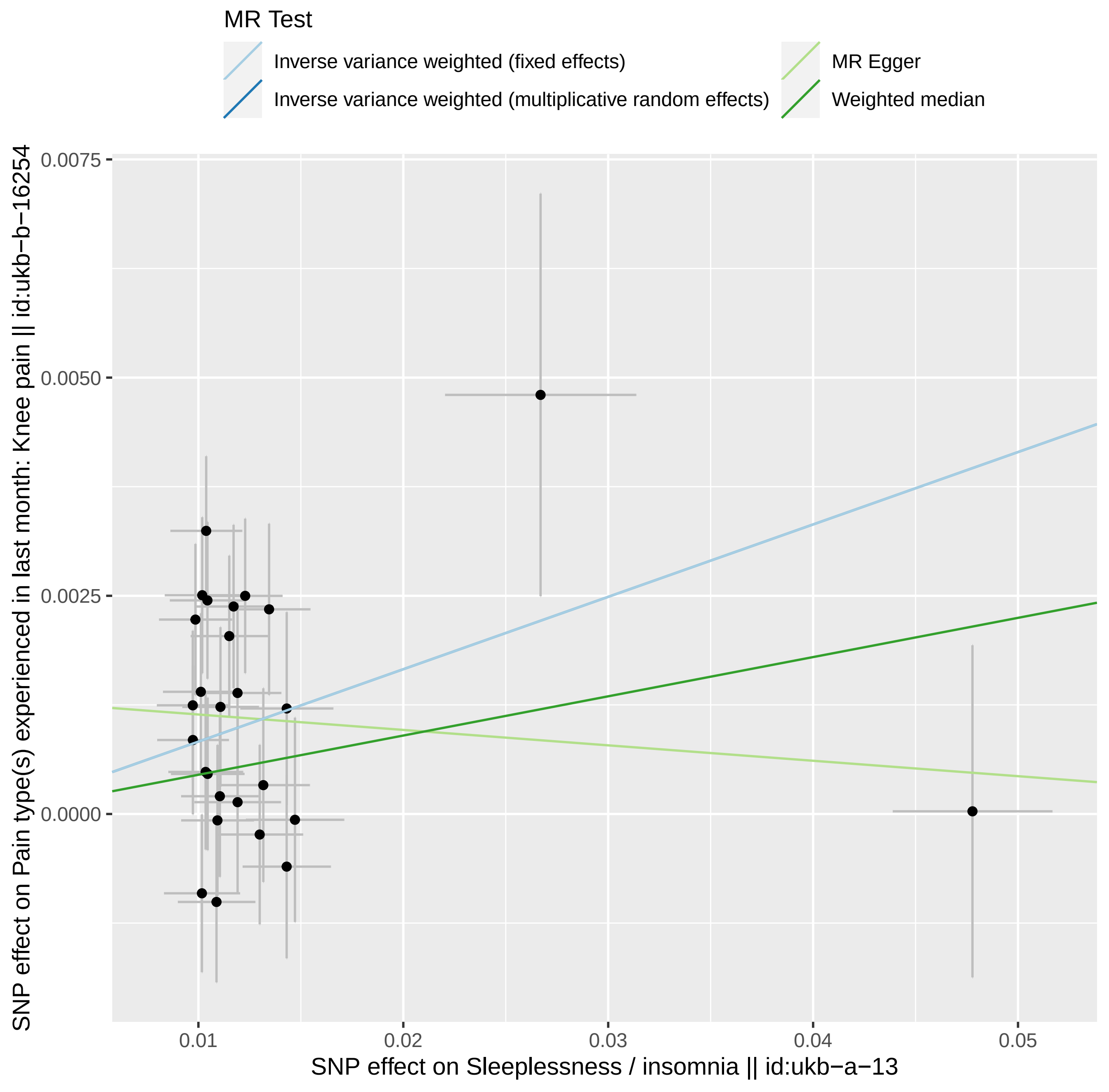

MR Test
Inverse variance weighted (fixed effects)
Inverse variance weighted (multiplicative random effects)
MR Egger
Weighted median
SNP effect on Pain type(s) experienced in last month: Knee pain || id:ukb−b−16254
0.0075
0.0050
0.0025
0.0000
0.01
0.02
0.03
0.04
0.05
SNP effect on Sleeplessness / insomnia || id:ukb−a−13

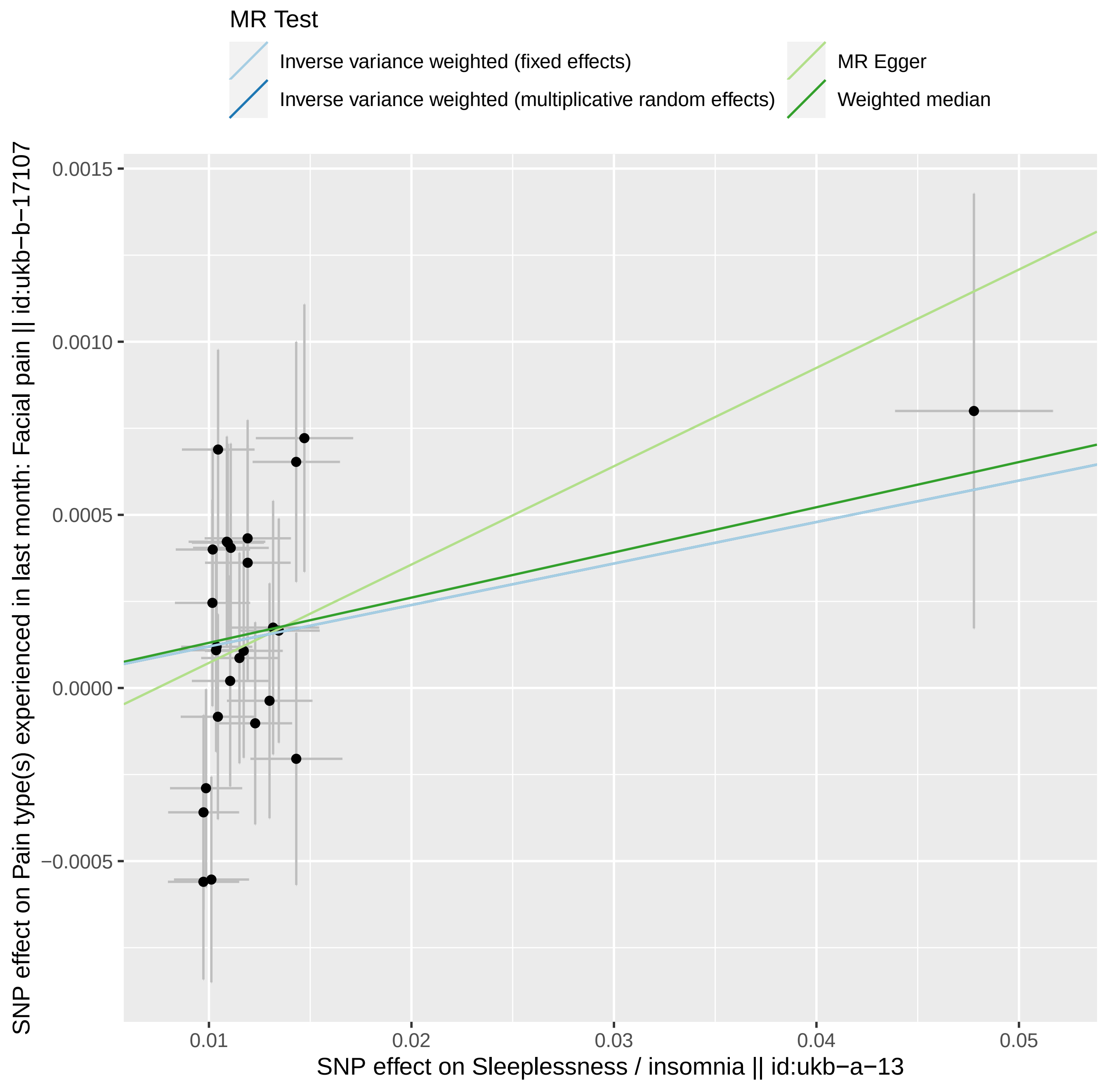

MR Test
Inverse variance weighted (fixed effects)
Inverse variance weighted (multiplicative random effects)
MR Egger
Weighted median
0.0015
0.0010
0.0005
0.0000
−0.0005
0.01
0.02
0.03
0.04
0.05
SNP effect on Sleeplessness / insomnia || id:ukb−a−13
SNP effect on Pain type(s) experienced in last month: Facial pain || id:ukb−b−17107

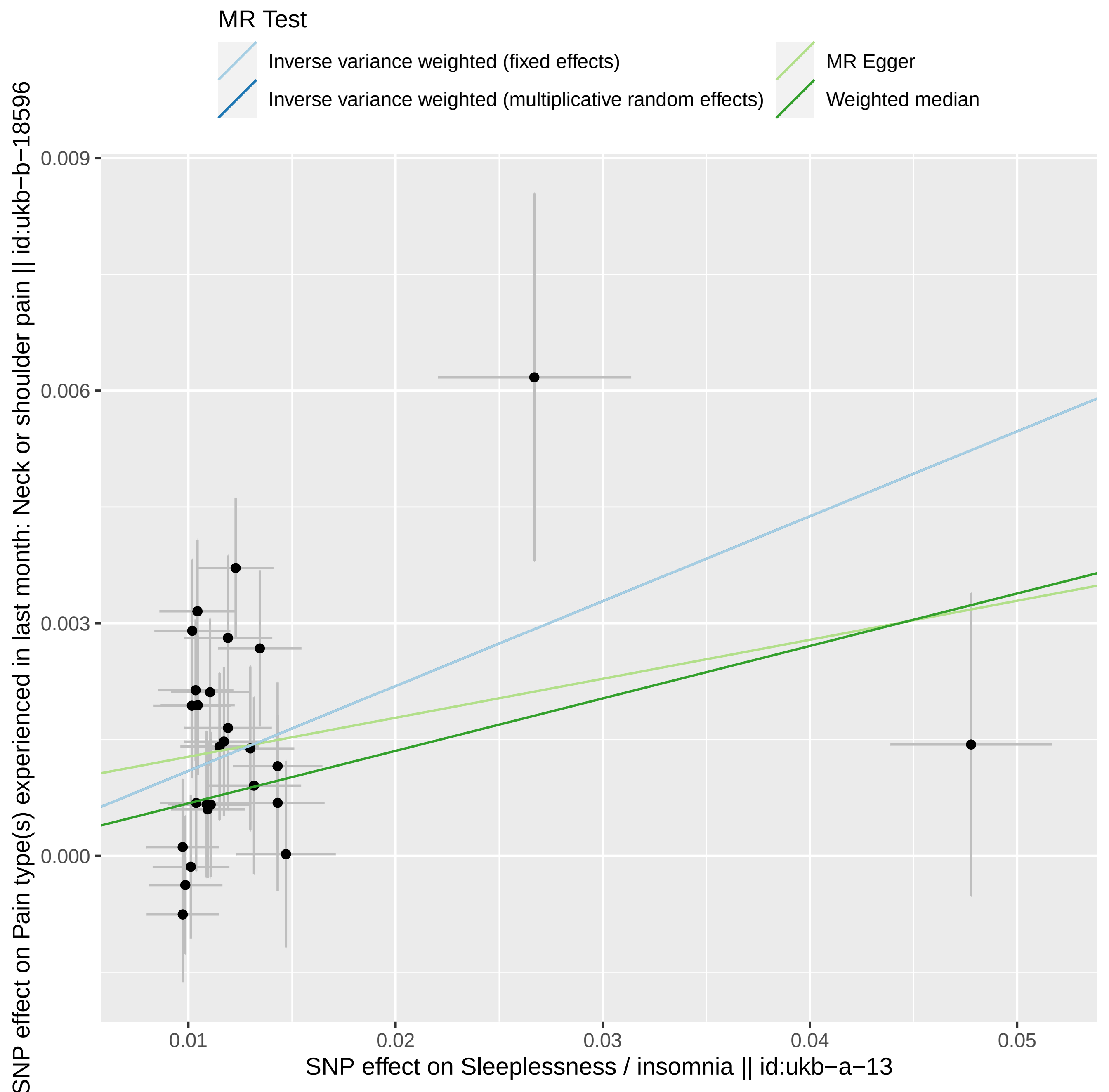

MR Test
Inverse variance weighted (fixed effects)
Inverse variance weighted (multiplicative random effects)
MR Egger
Weighted median
0.009
0.006
0.003
0.000
0.01
0.02
0.03
0.04
0.05
SNP effect on Sleeplessness / insomnia || id:ukb−a−13
SNP effect on Pain type(s) experienced in last month: Neck or shoulder pain || id:ukb−b−18596

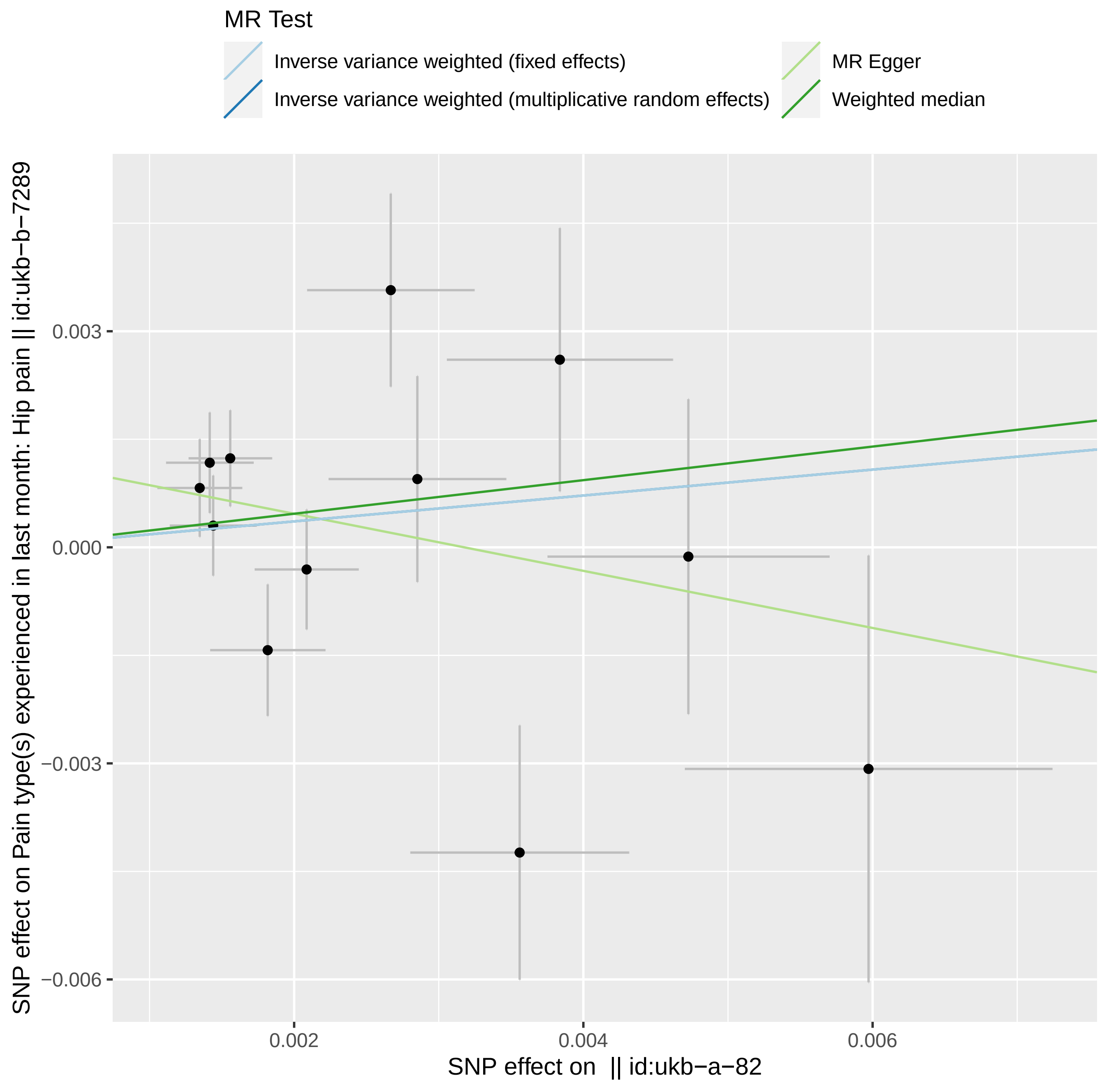
MR Test
Inverse variance weighted (fixed effects)
Inverse variance weighted (multiplicative random effects)
MR Egger
Weighted median
SNP effect on Pain type(s) experienced in last month: Hip pain || id:ukb−b−7289
0.003
0.000
−0.003
−0.006
0.002
0.004
0.006
SNP effect on || id:ukb−a−82

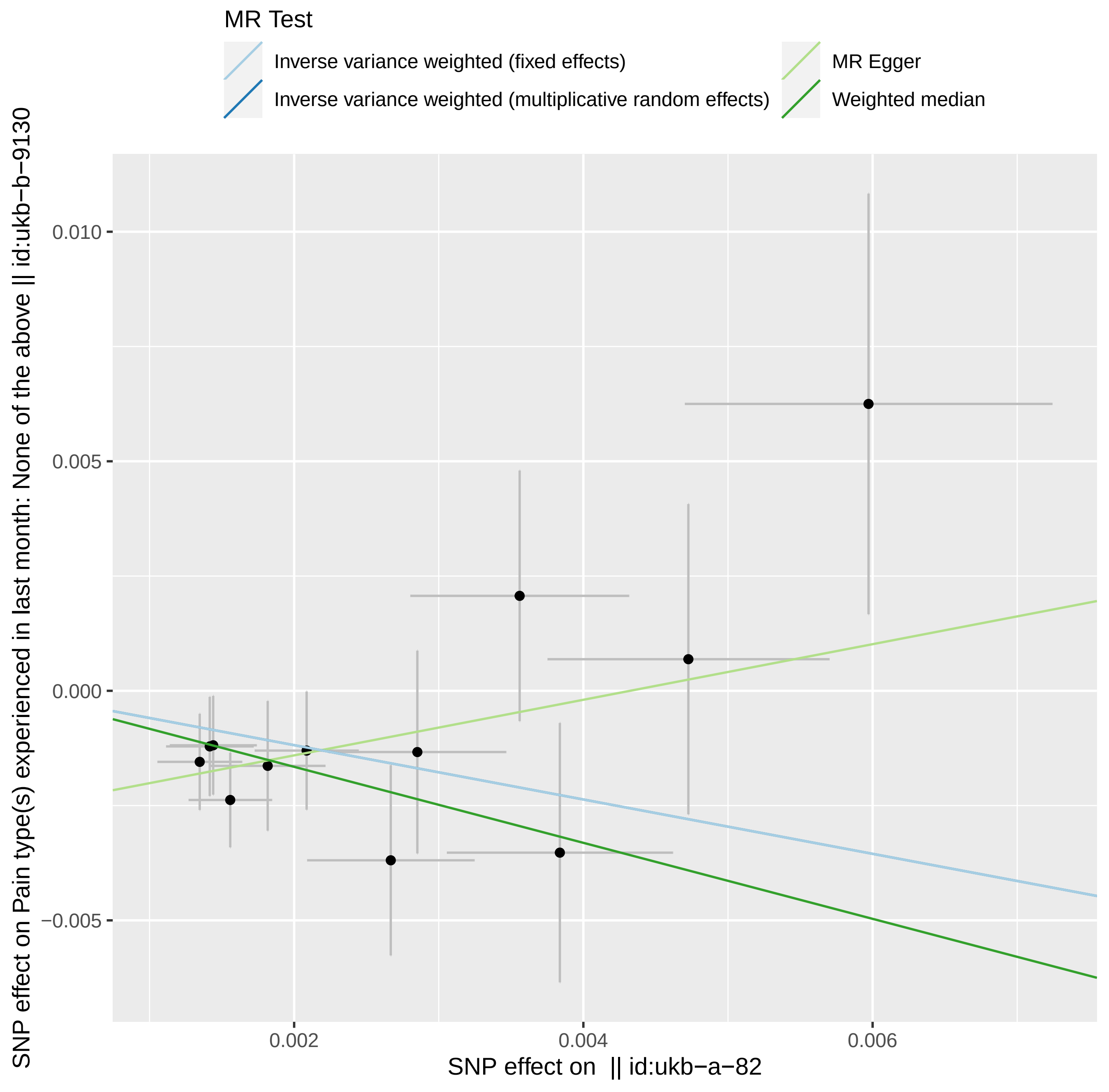
MR Test
Inverse variance weighted (fixed effects)
Inverse variance weighted (multiplicative random effects)
MR Egger
Weighted median
0.010
0.005
0.000
−0.005
0.002
0.004
0.006
SNP effect on Pain type(s) experienced in last month: None of the above || id:ukb−b−9130
SNP effect on || id:ukb−a−82

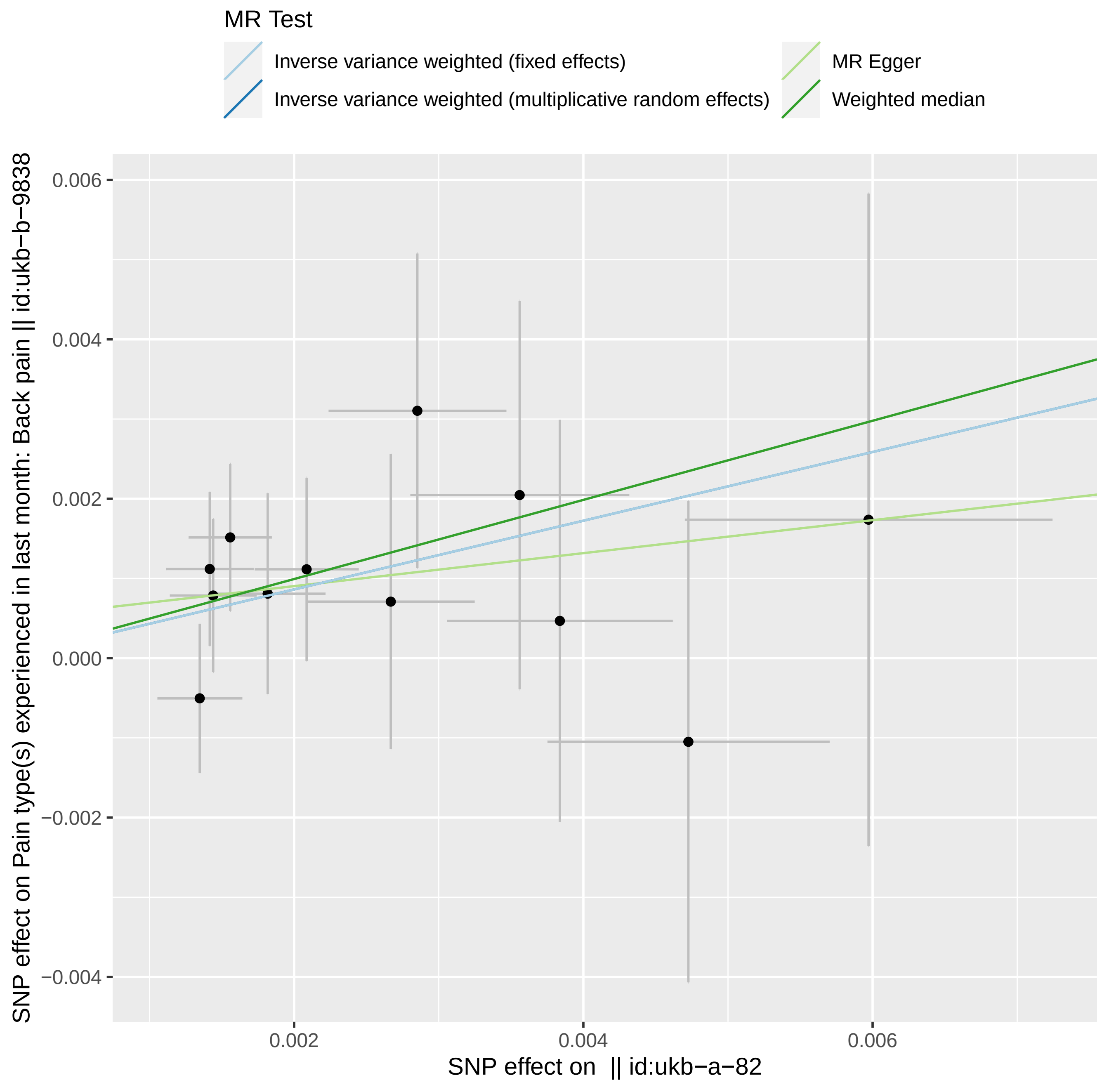

MR Test
Inverse variance weighted (fixed effects)
Inverse variance weighted (multiplicative random effects)
MR Egger
Weighted median
0.006
0.004
0.002
0.000
−0.002
−0.004
SNP effect on Pain type(s) experienced in last month: Back pain || id:ukb−b−9838
0.002
0.004
0.006
SNP effect on || id:ukb−a−82

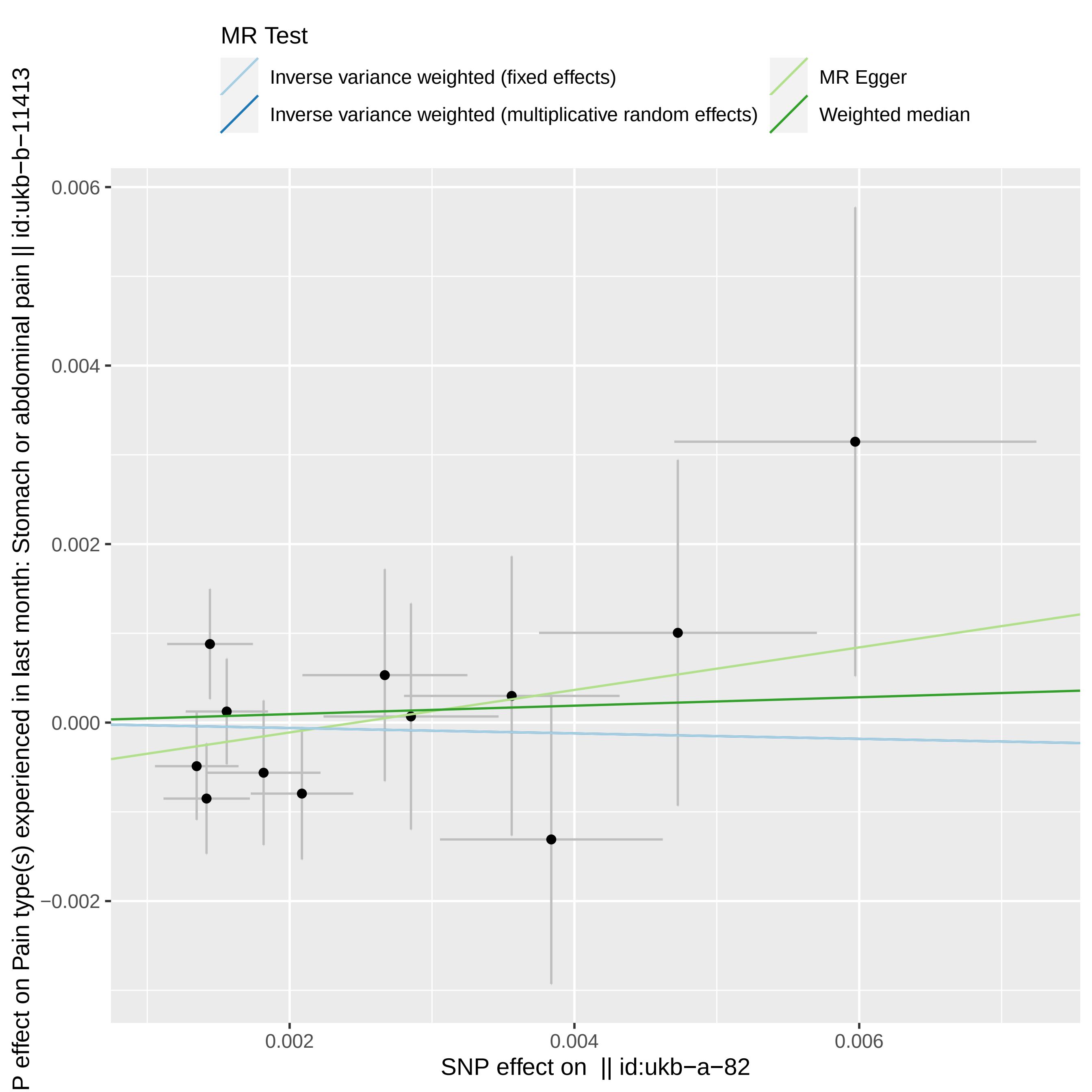

MR Test
Inverse variance weighted (fixed effects)
Inverse variance weighted (multiplicative random effects)
MR Egger
Weighted median
0.006
0.004
0.002
0.000
−0.002
0.002
0.004
0.006
SNP effect on || id:ukb−a−82
P effect on Pain type(s) experienced in last month: Stomach or abdominal pain || id:ukb−b−11413

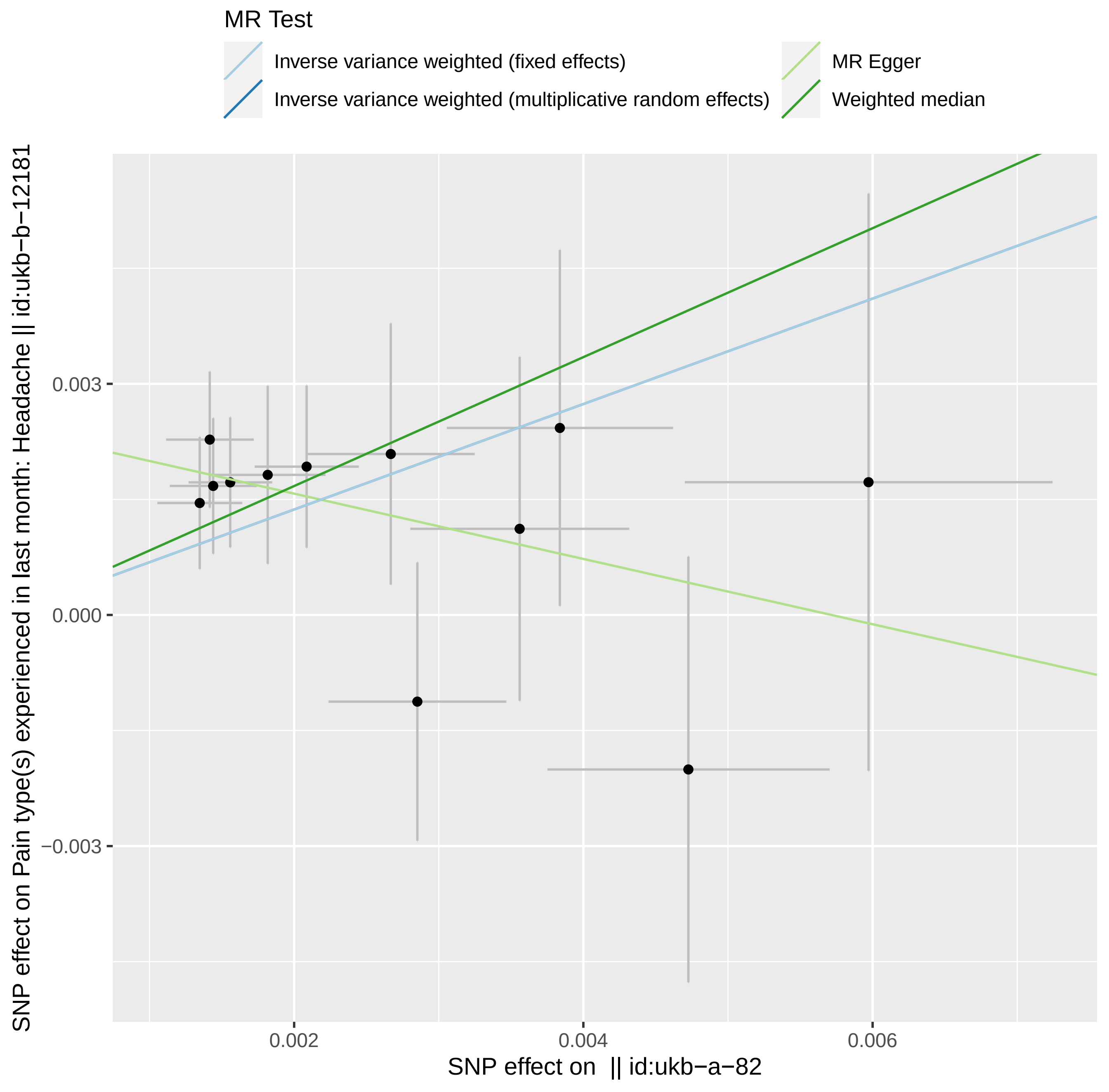

MR Test
Inverse variance weighted (fixed effects)
Inverse variance weighted (multiplicative random effects)
MR Egger
Weighted median
0.003
0.000
−0.003
0.002
0.004
0.006
SNP effect on Pain type(s) experienced in last month: Headache || id:ukb−b−12181
SNP effect on || id:ukb−a−82

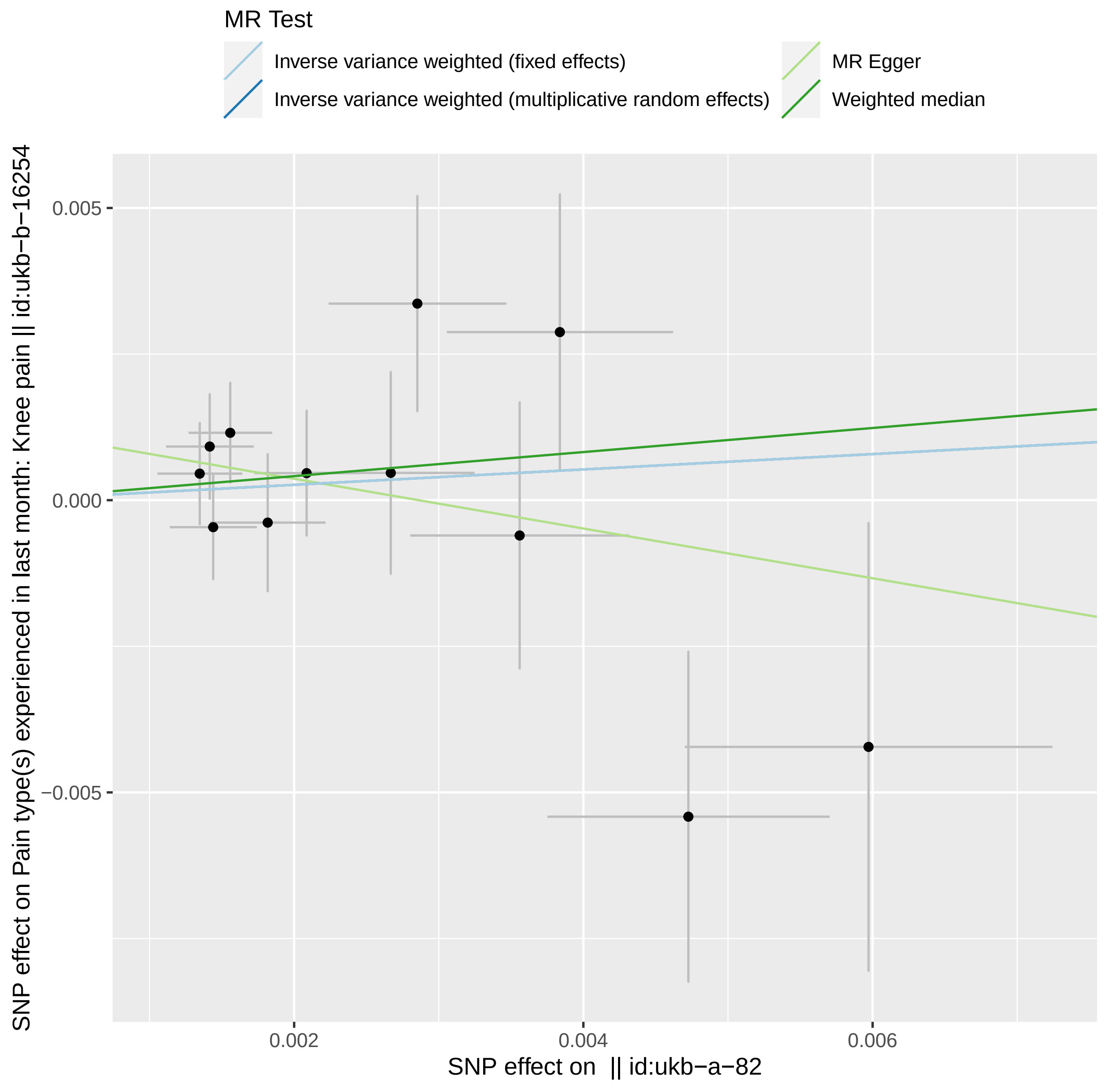

MR Test
Inverse variance weighted (fixed effects)
Inverse variance weighted (multiplicative random effects)
MR Egger
Weighted median
0.005
0.000
−0.005
0.002
0.004
0.006
SNP effect on Pain type(s) experienced in last month: Knee pain || id:ukb−b−16254
SNP effect on || id:ukb−a−82

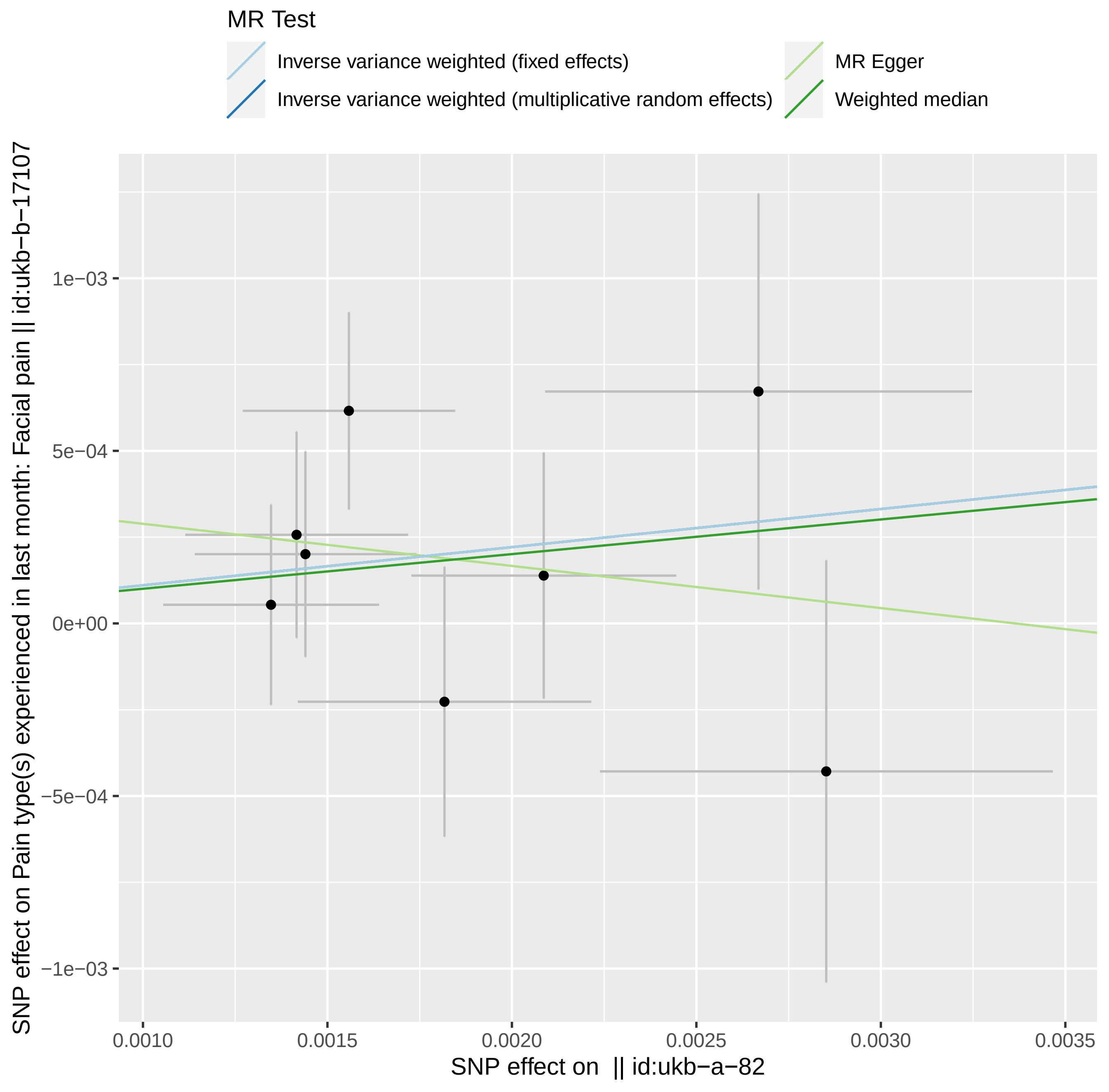
MR Test
Inverse variance weighted (fixed effects)
Inverse variance weighted (multiplicative random effects)
MR Egger
Weighted median
SNP effect on Pain type(s) experienced in last month: Facial pain || id:ukb−b−17107
1e−03
5e−04
0e+00
−5e−04
−1e−03
0.0010
0.0015
0.0020
0.0025
0.0030
0.0035
SNP effect on || id:ukb−a−82

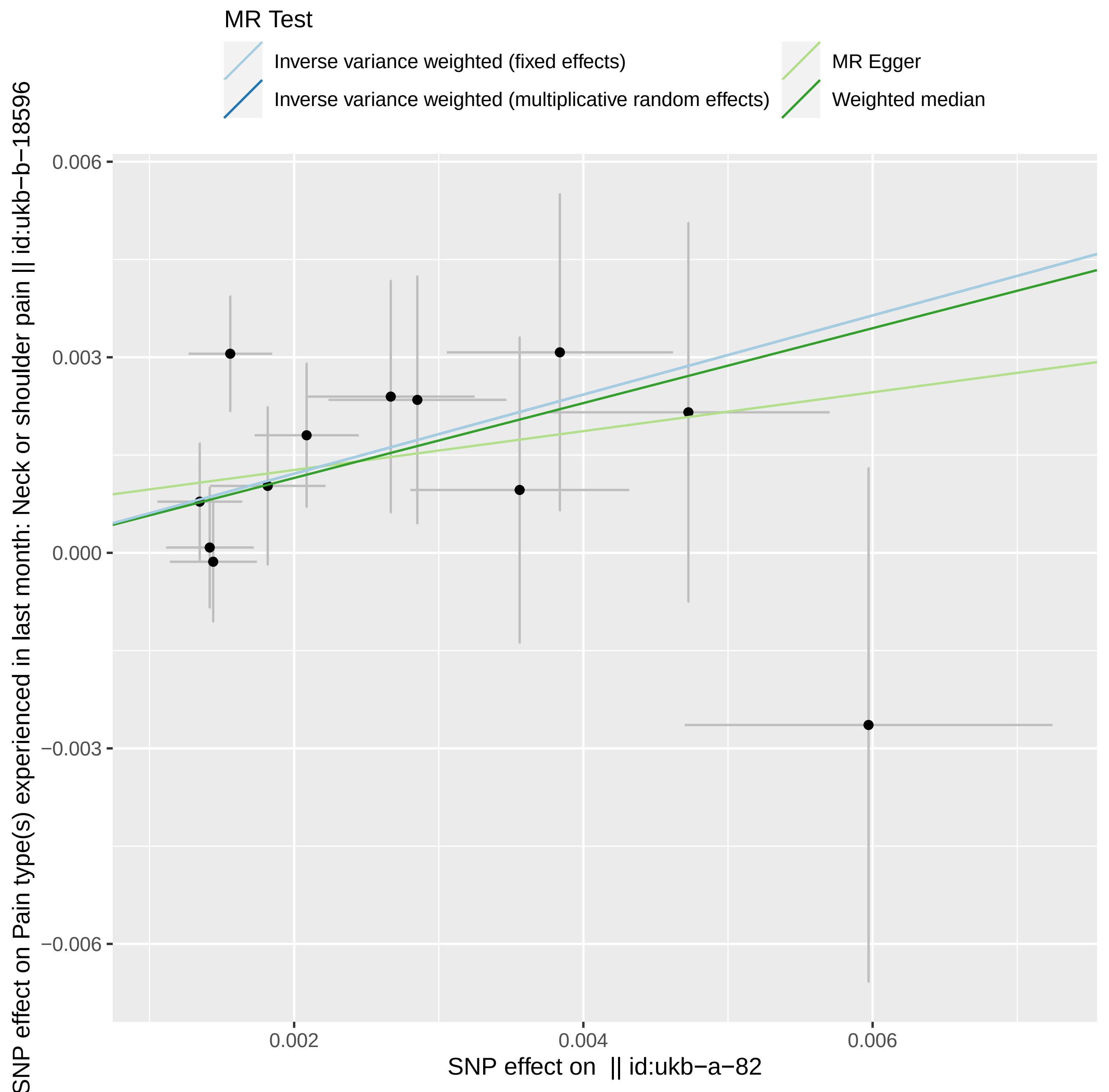

MR Test
Inverse variance weighted (fixed effects)
Inverse variance weighted (multiplicative random effects)
MR Egger
Weighted median
0.006
0.003
0.000
−0.003
−0.006
0.002
0.004
0.006
SNP effect on || id:ukb−a−82
SNP effect on Pain type(s) experienced in last month: Neck or shoulder pain || id:ukb−b−18596

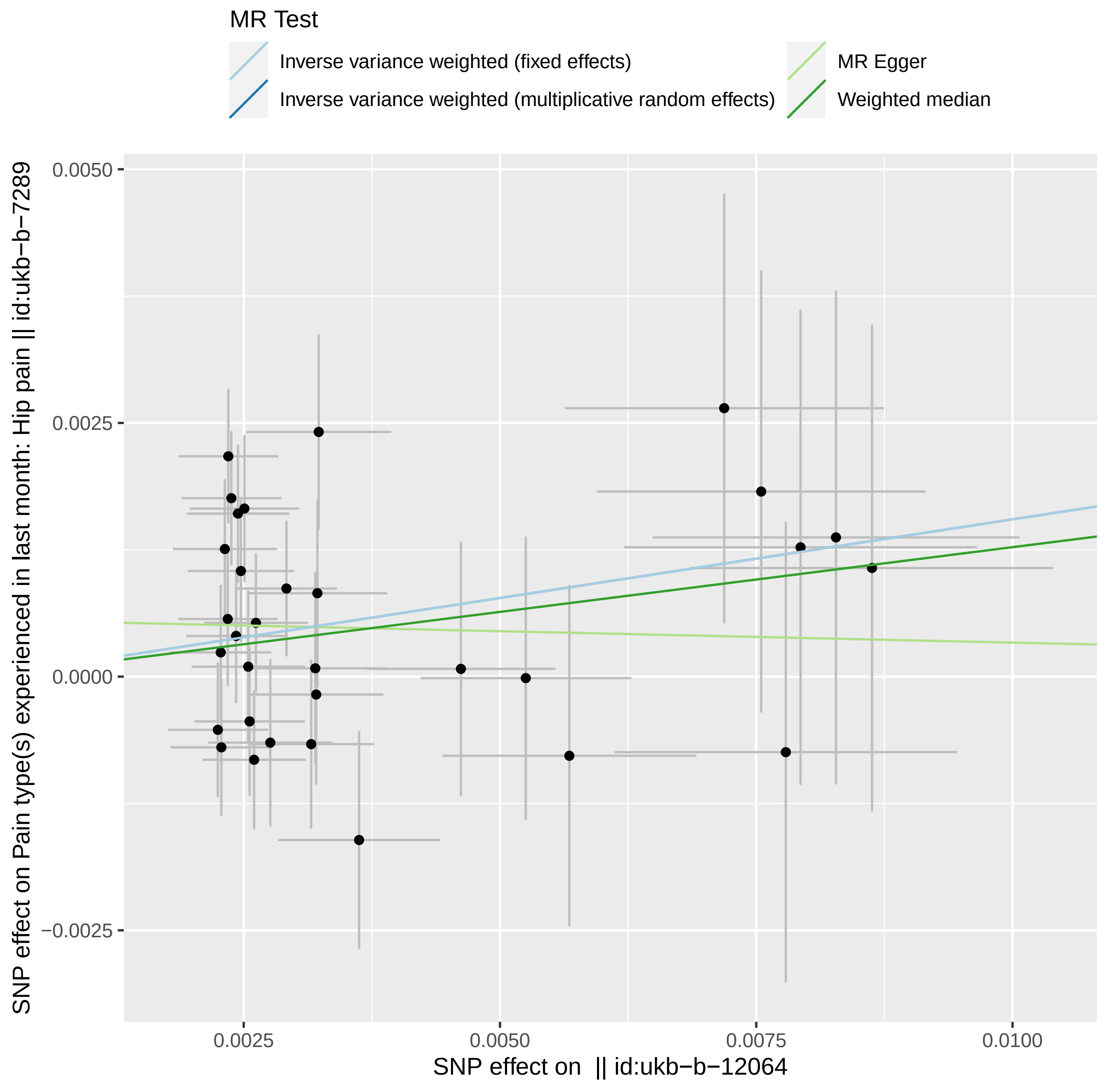
MR Test
Inverse variance weighted (fixed effects)
Inverse variance weighted (multiplicative random effects)
MR Egger
Weighted median
SNP effect on Pain type(s) experienced in last month: Hip pain || id:ukb−b−7289
0.0050
0.0025
0.0000
−0.0025
0.0025
0.0050
0.0075
0.0100
SNP effect on || id:ukb−b−12064

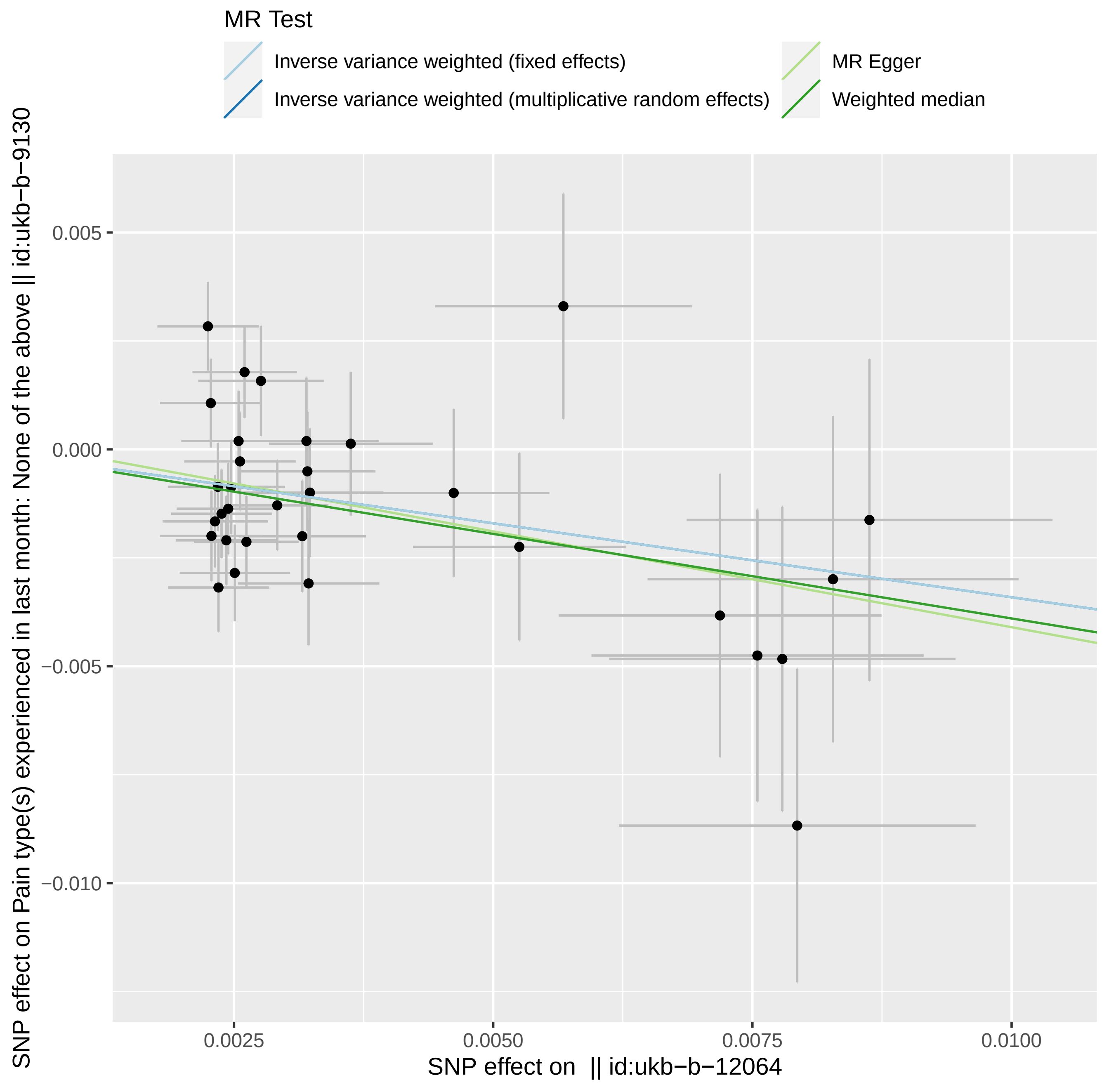

MR Test
Inverse variance weighted (fixed effects)
Inverse variance weighted (multiplicative random effects)
MR Egger
Weighted median
SNP effect on Pain type(s) experienced in last month: None of the above || id:ukb-b-9130
0.005
0.000
−0.005
−0.010
0.0025
0.0050
0.0075
0.0100
SNP effect on || id:ukb-b-12064

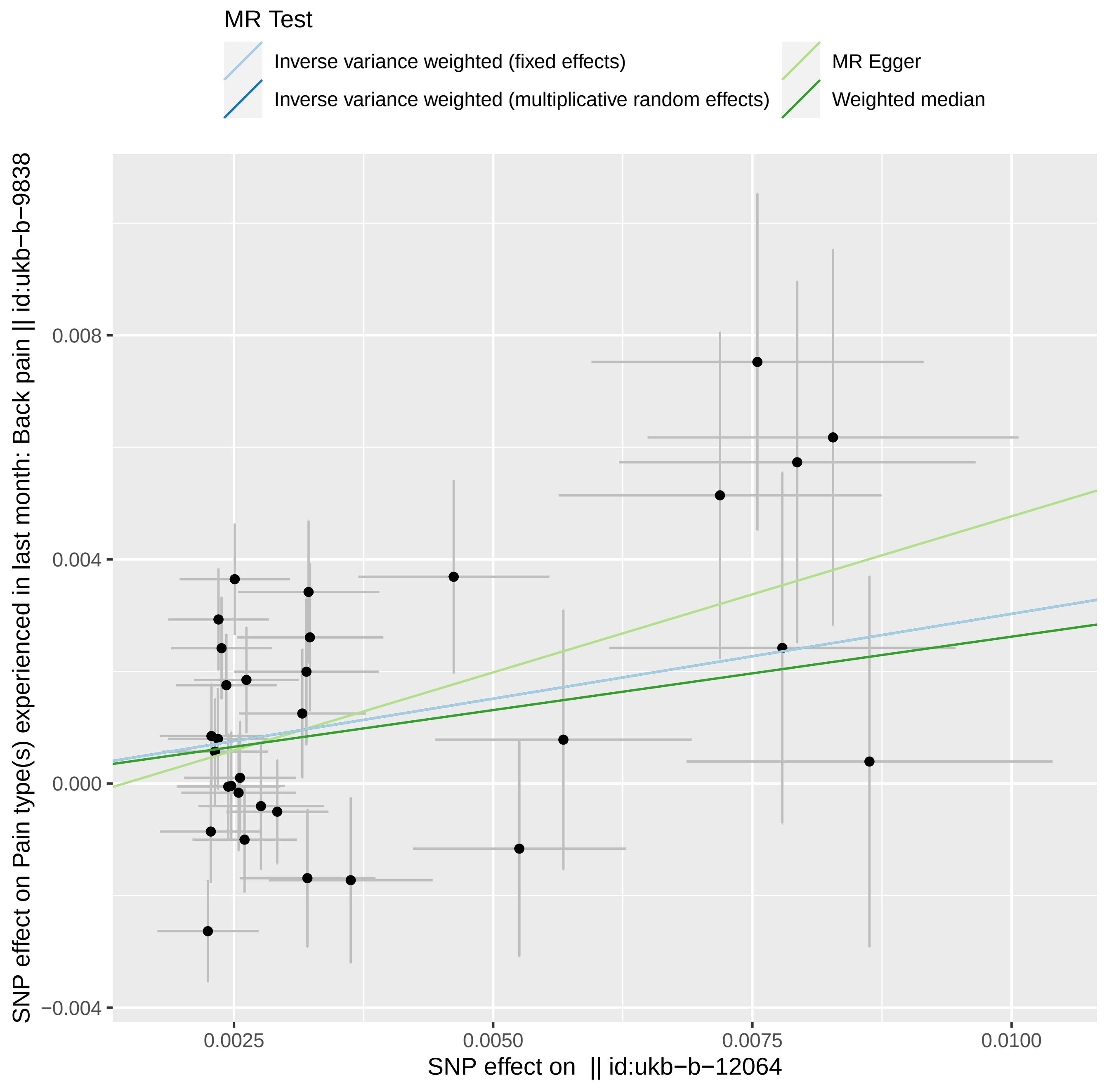

MR Test
Inverse variance weighted (fixed effects)
Inverse variance weighted (multiplicative random effects)
MR Egger
Weighted median
SNP effect on Pain type(s) experienced in last month: Back pain || id:ukb−b−9838
0.008
0.004
0.000
−0.004
0.0025
0.0050
0.0075
0.0100
SNP effect on || id:ukb−b−12064

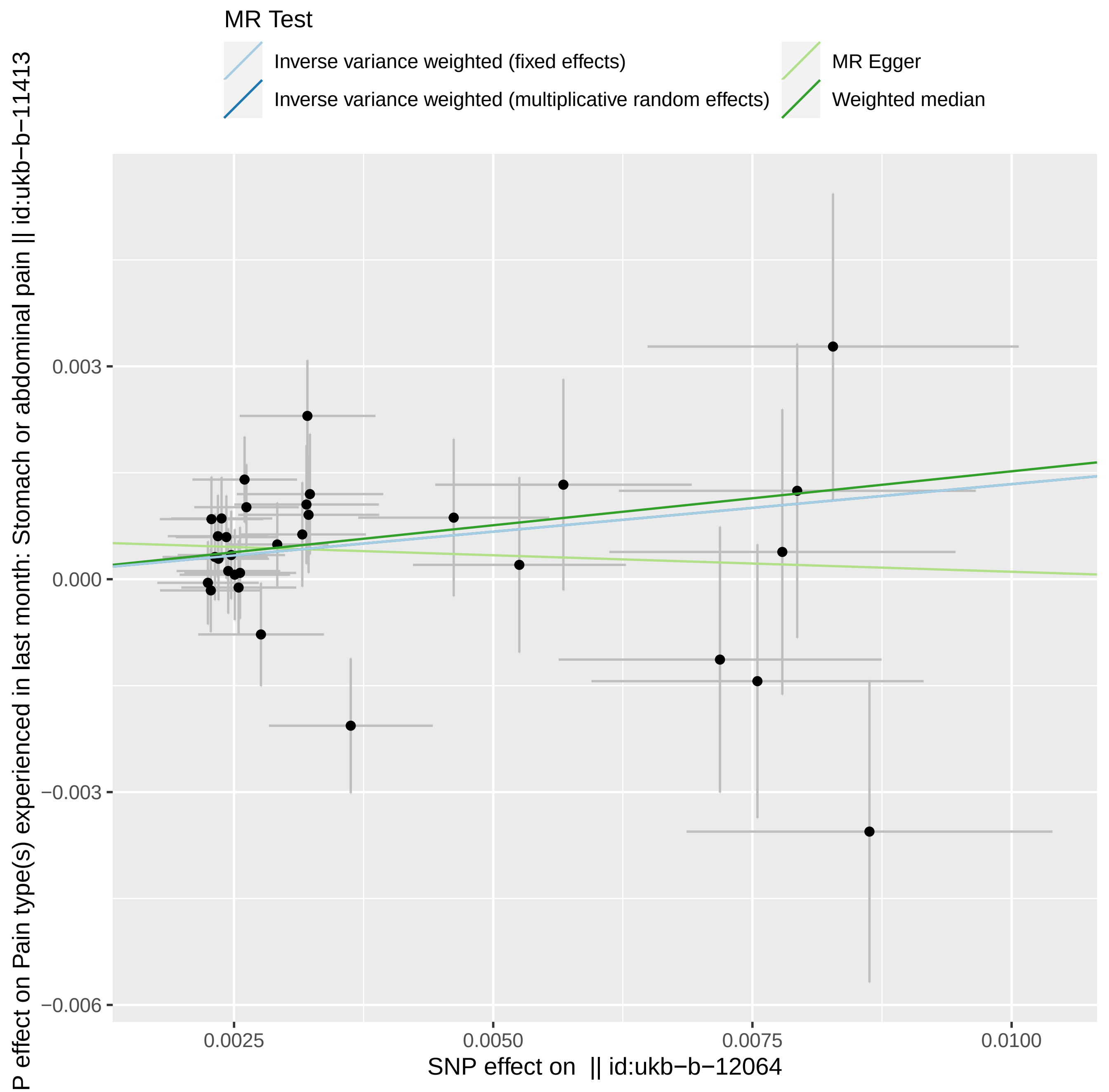
MR Test
Inverse variance weighted (fixed effects)
Inverse variance weighted (multiplicative random effects)
MR Egger
Weighted median
0.003
0.000
−0.003
−0.006
0.0025
0.0050
0.0075
0.0100
SNP effect on || id:ukb−b−12064
P effect on Pain type(s) experienced in last month: Stomach or abdominal pain || id:ukb−b−11413

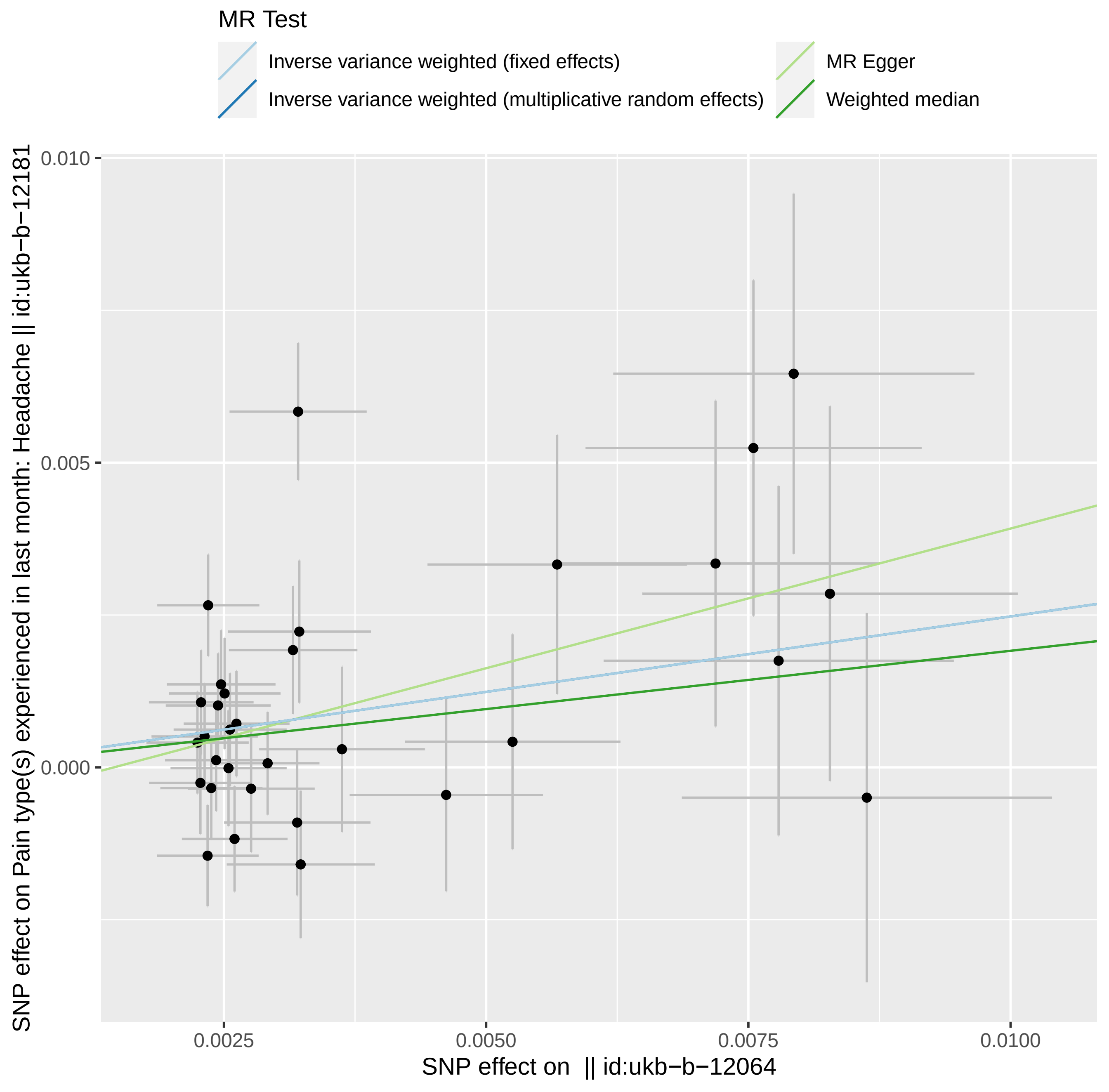

MR Test
Inverse variance weighted (fixed effects)
Inverse variance weighted (multiplicative random effects)
MR Egger
Weighted median
0.010
0.005
0.000
0.0025
0.0050
0.0075
0.0100
SNP effect on Pain type(s) experienced in last month: Headache || id:ukb−b−12181
SNP effect on || id:ukb−b−12064

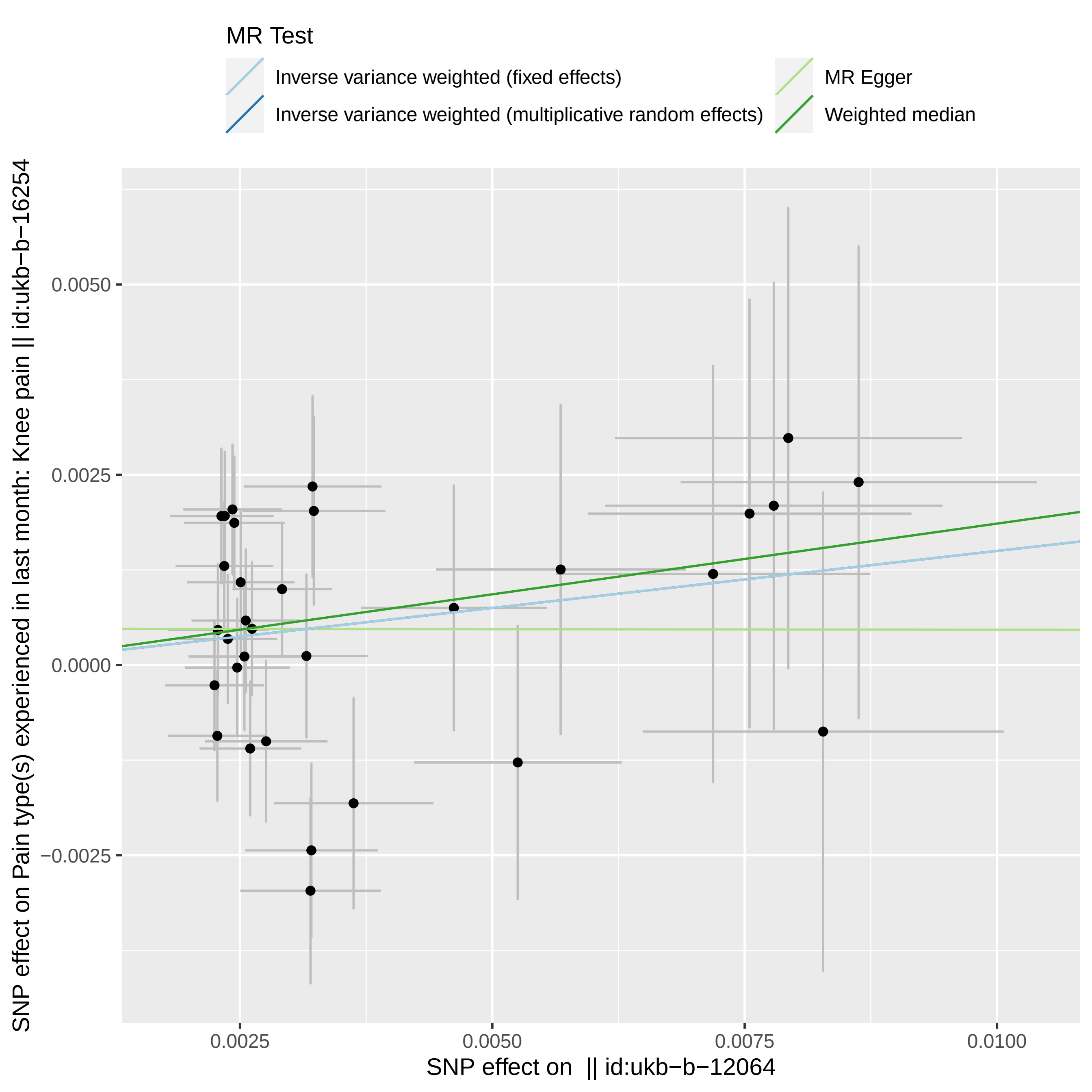

MR Test
Inverse variance weighted (fixed effects)
Inverse variance weighted (multiplicative random effects)
MR Egger
Weighted median
SNP effect on Pain type(s) experienced in last month: Knee pain || id:ukb−b−16254
0.0050
0.0025
0.0000
−0.0025
0.0025
0.0050
0.0075
0.0100
SNP effect on || id:ukb−b−12064

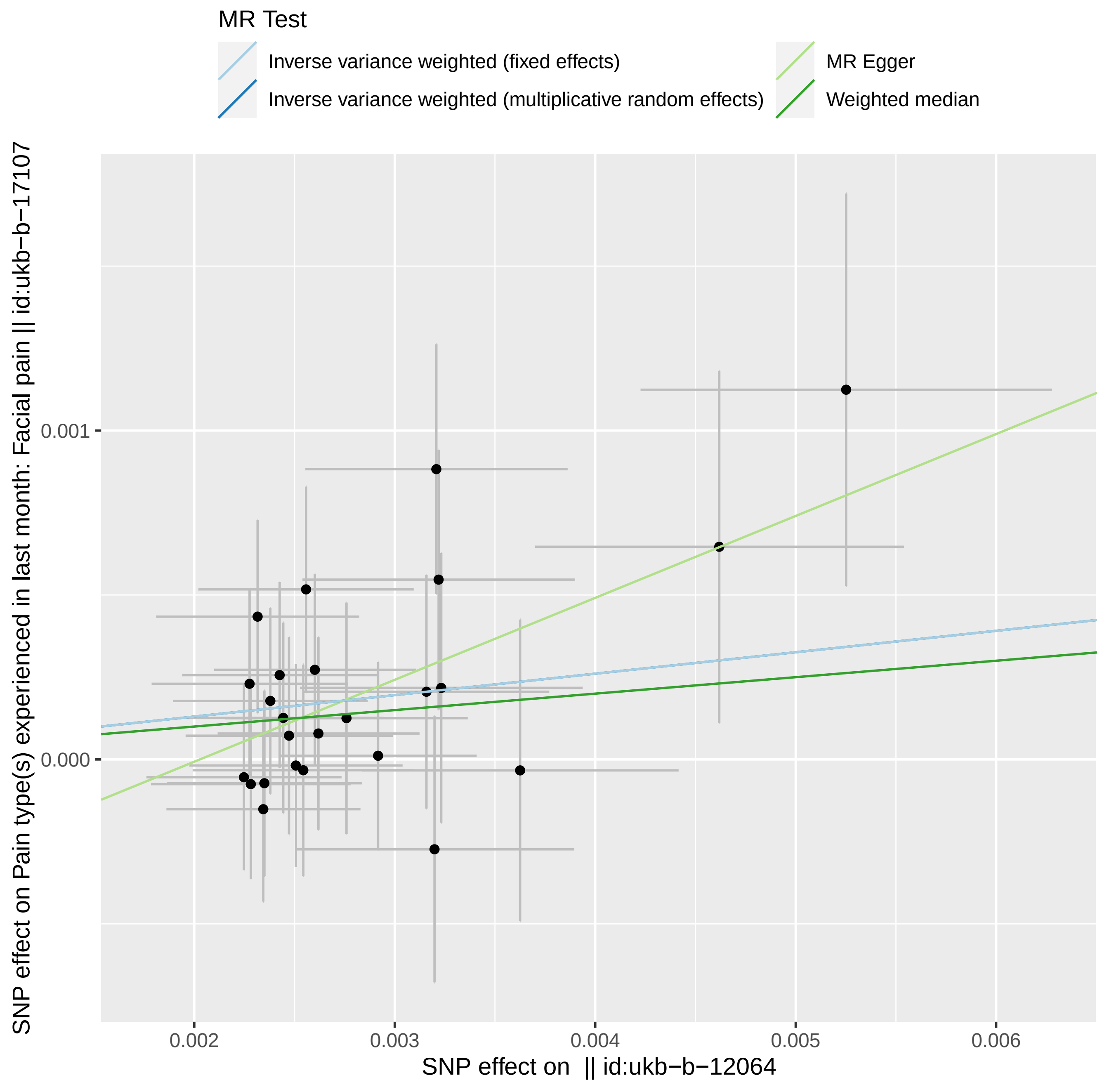

MR Test
Inverse variance weighted (fixed effects)
Inverse variance weighted (multiplicative random effects)
MR Egger
Weighted median
0.001
0.000
0.002
0.003
0.004
0.005
0.006
SNP effect on Pain type(s) experienced in last month: Facial pain || id:ukb−b−17107
SNP effect on || id:ukb−b−12064

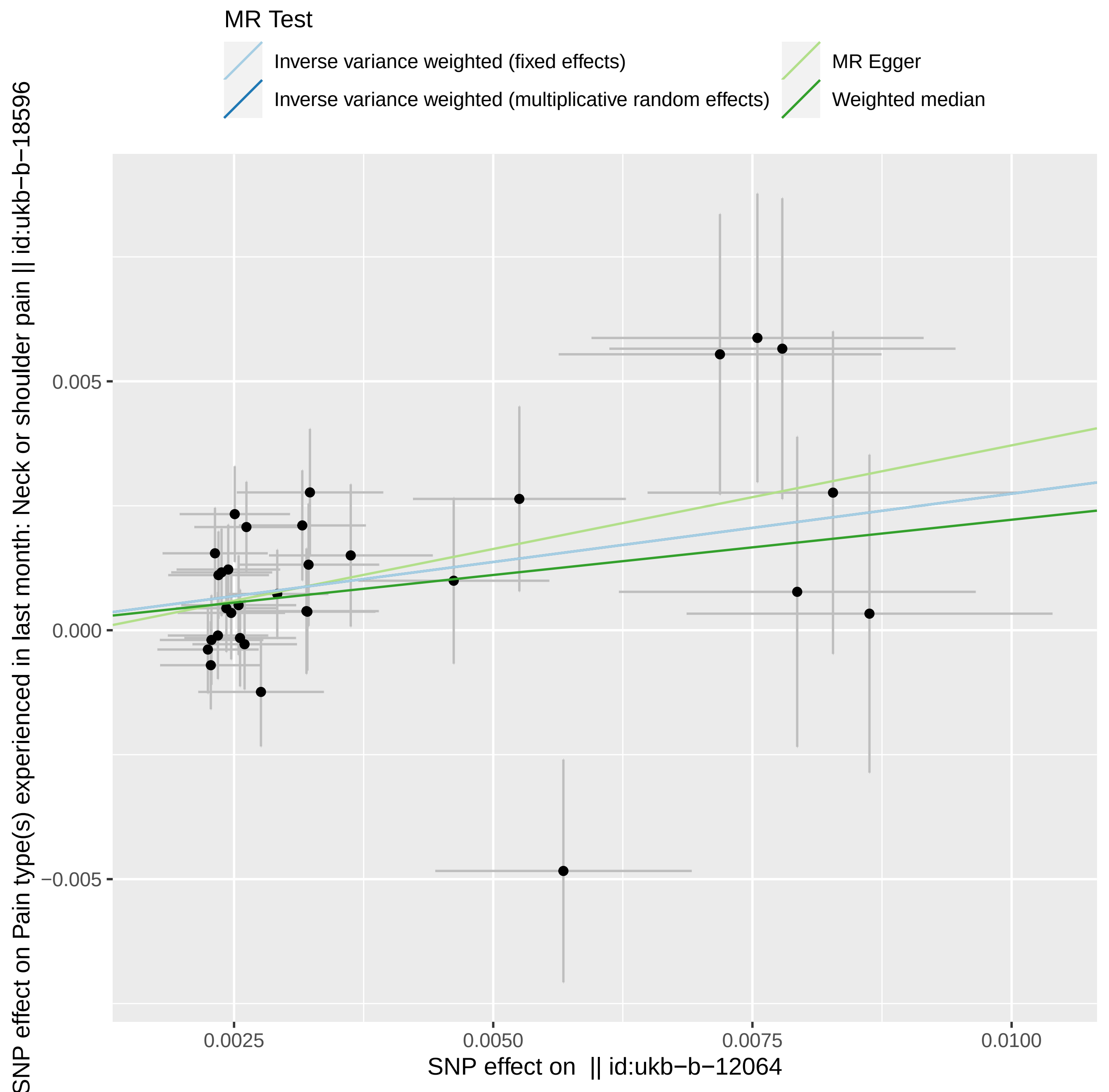

MR Test
Inverse variance weighted (fixed effects)
Inverse variance weighted (multiplicative random effects)
MR Egger
Weighted median
SNP effect on Pain type(s) experienced in last month: Neck or shoulder pain || id:ukb−b−18596
0.005
0.000
−0.005
0.0025
0.0050
0.0075
0.0100
SNP effect on || id:ukb−b−12064
