# Supplementary material for: Exploring the bidirectional relationship between pain and mental disorders: a comprehensive Mendelian randomization study
Source: J Headache Pain. 2023 Jul 7;24(1):82. doi: 10.1186/s10194-023-01612-2 (PMC10326936; doi:10.1186/s10194-023-01612-2)
Supplement: Supplementary file 5 — Additional file 5: Supplementary file 5. The heterogeneity and horizontal pleiotropy among genetic instruments. [file 10194_2023_1612_MOESM5_ESM.docx]

**Supplementary file 5**

The heterogeneity and horizontal pleiotropy among genetic instruments

**Heterogeneity statistics of the MR analysis - localized pain on the risk of sleeplessness/insomnia, anxiety/panic attacks and depression**

| Exposure | Method | Sleeplessness / insomnia | | | Anxiety/panic attacks | | | Depression | | |
| --- | --- | --- | --- | --- | --- | --- | --- | --- | --- | --- |
|  |  | **Q** | **Q_df** | ***P* value** | **Q** | **Q_df** | ***P* value** | **Q** | **Q_df** | ***P* value** |
| Headache | MR Egger | 86.93 | 43 | <0.001 | 63.06 | 43 | 0.02 | 72.23 | 43 | 0.003 |
|  | IVW | 90.31 | 44 | <0.001 | 63.2 | 44 | 0.03 | 73.76 | 44 | 0.003 |
| Facial pain | MR Egger | 15.04 | 14 | 0.38 | 22.51 | 14 | 0.07 | 16.17 | 14 | 0.30 |
|  | IVW | 16.06 | 15 | 0.38 | 23.53 | 15 | 0.07 | 16.19 | 15 | 0.37 |
| Neck or shoulder pain | MR Egger | 28.37 | 4 | <0.001 | 4.05 | 4 | 0.40 | 5.92 | 4 | 0.21 |
|  | IVW | 28.49 | 5 | <0.001 | 4.62 | 5 | 0.46 | 5.93 | 5 | 0.31 |
| Back pain | MR Egger | 28.71 | 18 | 0.05 | 14.95 | 18 | 0.67 | 26.19 | 17 | 0.07 |
|  | IVW (REM) | 29.33 | 19 | 0.06 | 15.45 | 19 | 0.69 | 30.34 | 18 | 0.03 |
| Stomach and abdominal pain | MR Egger | 63.64 | 28 | <0.001 | 40.40 | 28 | 0.06 | 18.77 | 26 | 0.85 |
|  | IVW | 78.18 | 29 | <0.001 | 44.88 | 29 | 0.03 | 18.85 | 27 | 0.88 |
| Hip pain | MR Egger | 3.00 | 2 | 0.22 | 0.47 | 2 | 0.79 | 9.17 | 2 | 0.01 |
|  | IVW | 6.44 | 3 | 0.09 | 0.76 | 3 | 0.86 | 9.37 | 3 | 0.02 |
| Knee pain | MR Egger | 34.24 | 9 | <0.001 | 5.06 | 9 | 0.83 | 5.66 | 9 | 0.77 |
|  | IVW | 40.55 | 10 | <0.001 | 5.20 | 10 | 0.88 | 5.69 | 10 | 0.84 |
| None of above | MR Egger | 66.03 | 34 | <0.001 | 29.3 | 34 | 0.70 | 32.61 | 35 | 0.58 |
|  | IVW | 68.14 | 35 | <0.001 | 29.3 | 35 | 0.74 | 33.7 | 36 | 0.58 |

Abbreviations: MR, mendelian randomization; IVW, inverse-variance weighted.

**Horizontal pleiotropy of the exposure SNPs in the MR analysis - localized pain on the risk of sleeplessness/insomnia, anxiety/panic attacks and depression**

| Exposure | Sleeplessness / insomnia | | | Anxiety/panic attacks | | | Depression | | |
| --- | --- | --- | --- | --- | --- | --- | --- | --- | --- |
|  | **ERI** | **SE** | **Directionality *P* value** | **ERI** | **SE** | **Directionality *P* value** | **ERI** | **SE** | **Directionality *P* value** |
| Headache | 0.002 | 0.001 | 0.20 | 0.00005 | <0.001 | 0.77 | 0.0003 | <0.001 | 0.35 |
| Facial pain | -0.003 | 0.003 | 0.35 | 0.0004 | <0.001 | 0.44 | 0.00009 | <0.001 | 0.90 |
| Neck or shoulder pain | -0.001 | 0.01 | 0.90 | -0.0004 | <0.001 | 0.50 | 0.00008 | 0.001 | 0.95 |
| Back pain | 0.002 | 0.002 | 0.54 | 0.0002 | <0.001 | 0.49 | 0.001 | <0.001 | 0.12 |
| Stomach and abdominal pain | 0.005 | 0.002 | 0.02 | 0.0004 | <0.001 | 0.09 | 0.0001 | <0.001 | 0.77 |
| Hip pain | -0.01 | 0.007 | 0.27 | -0.0005 | <0.001 | 0.64 | -0.0007 | 0.003 | 0.85 |
| Knee pain | 0.007 | 0.005 | 0.23 | -0.0002 | <0.001 | 0.71 | -0.0001 | <0.001 | 0.87 |
| None of above | -0.003 | 0.003 | 0.31 | 0.00001 | <0.001 | 0.97 | -0.0006 | <0.001 | 0.30 |

Abbreviations: SNP, single nucleotide polymorphism; MR, mendelian randomization; ERI, egger regression intercept; SE, standard error; GWAS, genome-wide association studies.

**Heterogeneity statistics of the MR analysis - sleeplessness/insomnia, anxiety/panic attacks and depression on the risk of localized pain**

| Outcome | Method | Sleeplessness / insomnia | | | Anxiety/panic attacks | | | Depression | | |
| --- | --- | --- | --- | --- | --- | --- | --- | --- | --- | --- |
|  |  | **Q** | **Q_df** | ***P* value** | **Q** | **Q_df** | ***P* value** | **Q** | **Q_df** | ***P* value** |
| Headache | MR Egger | 94 | 25 | <0.001 | 4.08 | 10 | 0.94 | 57.66 | 30 | 0.002 |
|  | IVW | 98.02 | 26 | <0.001 | 11.53 | 11 | 0.40 | 59.05 | 31 | 0.002 |
| Facial pain | MR Egger | 30.26 | 24 | 0.18 | 5.20 | 6 | 0.52 | 12.45 | 23 | 0.96 |
|  | IVW | 31.41 | 25 | 0.18 | 5.83 | 7 | 0.56 | 14.55 | 24 | 0.93 |
| Neck or shoulder pain | MR Egger | 40.61 | 25 | 0.02 | 10.45 | 10 | 0.40 | 35.12 | 30 | 0.24 |
|  | IVW | 42.32 | 26 | 0.02 | 10.97 | 11 | 0.45 | 35.69 | 31 | 0.26 |
| Back pain | MR Egger | 66.43 | 25 | <0.001 | 4.58 | 10 | 0.92 | 69.39 | 30 | <0.001 |
|  | IVW (REM) | 66.48 | 26 | <0.001 | 4.83 | 11 | 0.94 | 71.09 | 31 | <0.001 |
| Stomach and abdominal pain | MR Egger | 37.18 | 25 | 0.06 | 792 | 10 | 0.64 | 32.64 | 30 | 0.34 |
|  | IVW | 39.17 | 26 | 0.05 | 8.81 | 11 | 0.64 | 34.72 | 31 | 0.30 |
| Hip pain | MR Egger | 42.46 | 25 | 0.02 | 21.97 | 10 | 0.02 | 44.32 | 30 | 0.04 |
|  | IVW | 43.03 | 26 | 0.02 | 25.19 | 11 | 0.01 | 45.90 | 31 | 0.04 |
| Knee pain | MR Egger | 43.43 | 25 | 0.01 | 11.05 | 10 | 0.35 | 46.46 | 30 | 0.03 |
|  | IVW | 48.65 | 26 | 0.005 | 12.84 | 11 | 0.30 | 47.15 | 31 | 0.03 |
| None of above | MR Egger | 78.42 | 25 | <0.001 | 6.284 | 10 | 0.79 | 53.40 | 30 | 0.005 |
|  | IVW | 80.84 | 26 | <0.001 | 12.1 | 11 | 0.36 | 53.61 | 31 | 0.007 |

Abbreviations: MR, mendelian randomization; IVW, inverse-variance weighted.

**Horizontal pleiotropy of the exposure SNPs in the MR analysis - sleeplessness/insomnia, anxiety/panic attacks and depression on the risk of localized pain**

| Outcome | Sleeplessness / insomnia | | | Anxiety/panic attacks | | | Depression | | |
| --- | --- | --- | --- | --- | --- | --- | --- | --- | --- |
|  | **ERI** | **SE** | **Directionality *P* value** | **ERI** | **SE** | **Directionality *P* value** | **ERI** | **SE** | **Directionality *P* value** |
| Headache | 0.001 | 0.001 | 0.31 | 0.002 | <0.001 | 0.02 | -0.001 | <0.001 | 0.40 |
| Facial pain | -0.0002 | <0.001 | 0.35 | 0.0004 | <0.001 | 0.46 | -0.0005 | <0.001 | 0.16 |
| Neck or shoulder pain | 0.0008 | <0.001 | 0.31 | 0.001 | <0.001 | 0.50 | -0.0005 | 0.001 | 0.49 |
| Back pain | -0.0001 | 0.001 | 0.89 | 0.0005 | <0.001 | 0.62 | -0.0008 | <0.001 | 0.40 |
| Stomach and abdominal pain | 0.0006 | <0.001 | 0.26 | -0.001 | <0.001 | 0.37 | 0.001 | <0.001 | 0.18 |
| Hip pain | 0.0003 | <0.001 | 0.57 | 0.001 | 0.001 | 0.25 | 0.001 | 0.003 | 0.31 |
| Knee pain | 0.001 | 0.0008 | 0.10 | 0.001 | <0.001 | 0.23 | 0.0005 | <0.001 | 0.51 |
| None of above | -0.001 | 0.001 | 0.39 | -0.003 | 0.001 | 0.04 | 0.0004 | <0.001 | 0.73 |

Abbreviations: SNP, single nucleotide polymorphism; MR, mendelian randomization; ERI, egger regression intercept; SE, standard error; GWAS, genome-wide association studies.
