# Supplementary material for: Exploring the bidirectional relationship between pain and mental disorders: a comprehensive Mendelian randomization study
Source: J Headache Pain. 2023 Jul 7;24(1):82. doi: 10.1186/s10194-023-01612-2 (PMC10326936; doi:10.1186/s10194-023-01612-2)
Supplement: Supplementary file 6 — Additional file 6: Supplementary file 6. Results of Leave-one-out analysis. [file 10194_2023_1612_MOESM6_ESM.pdf]

Supplementary file 6

Results of Leave-one-out analysis

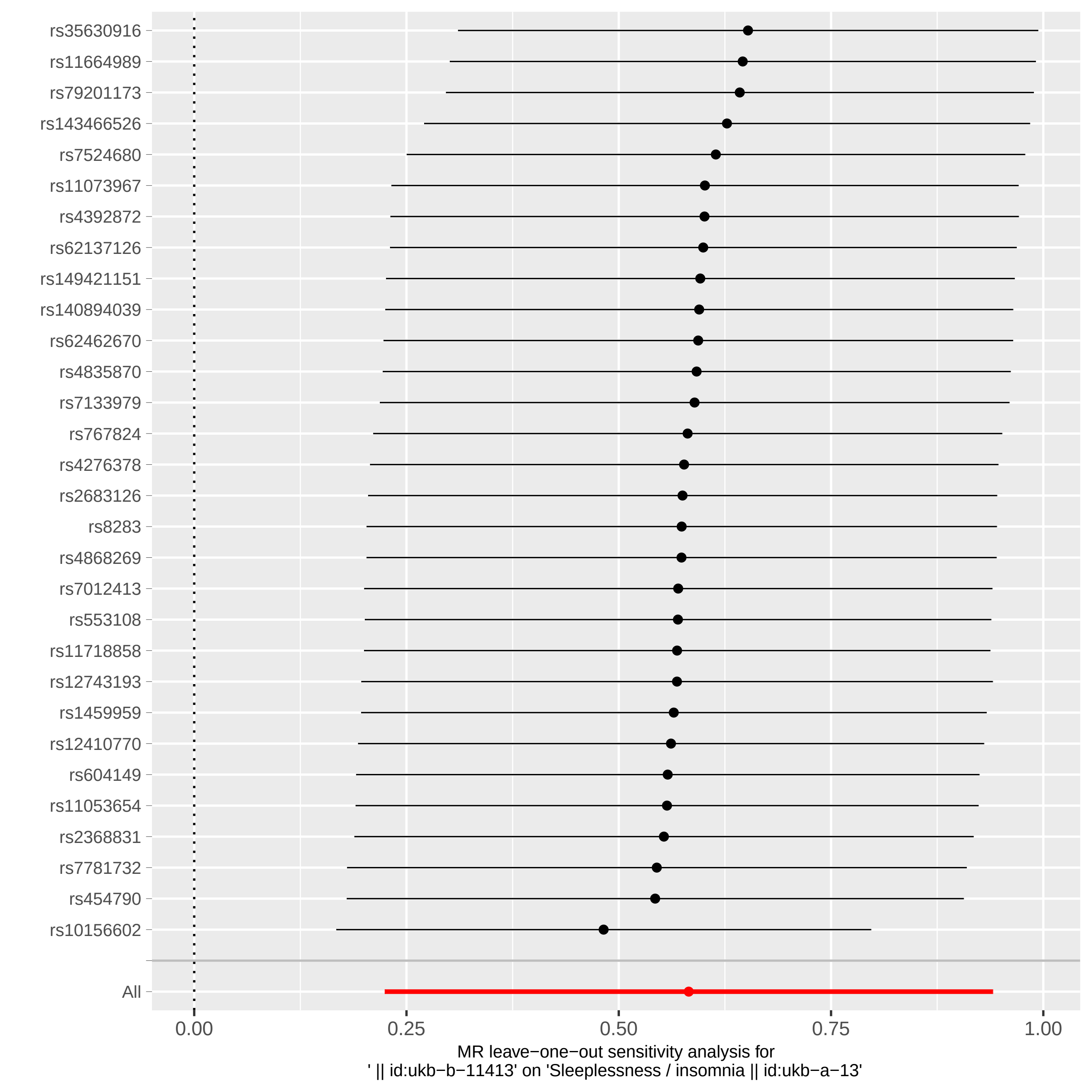
rs35630916
rs11664989
rs79201173
rs143466526
rs7524680
rs11073967
rs4392872
rs62137126
rs149421151
rs140894039
rs62462670
rs4835870
rs7133979
rs767824
rs4276378
rs2683126
rs8283
rs4868269
rs7012413
rs553108
rs11718858
rs12743193
rs1459959
rs12410770
rs604149
rs11053654
rs2368831
rs7781732
rs454790
rs10156602
All
0.00
0.25
0.50
0.75
1.00
MR leave−one−out sensitivity analysis for
' || id:ukb−b−11413' on 'Sleeplessness / insomnia || id:ukb−a−13'

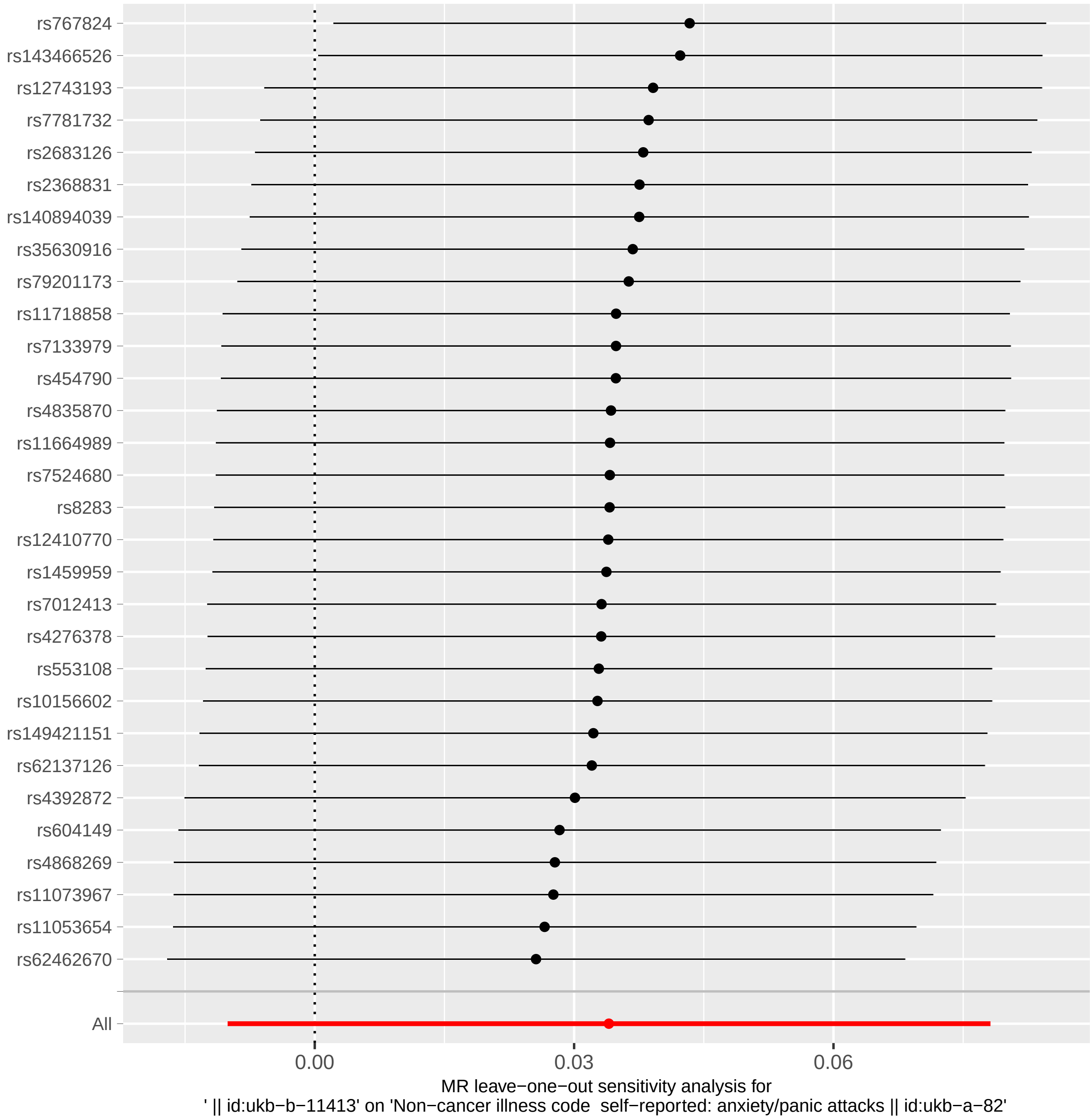
rs767824
rs143466526
rs12743193
rs7781732
rs2683126
rs2368831
rs140894039
rs35630916
rs79201173
rs11718858
rs7133979
rs454790
rs4835870
rs11664989
rs7524680
rs8283
rs12410770
rs1459959
rs7012413
rs4276378
rs553108
rs10156602
rs149421151
rs62137126
rs4392872
rs604149
rs4868269
rs11073967
rs11053654
rs62462670
All
0.00
0.03
0.06
MR leave−one−out sensitivity analysis for
' || id:ukb−b−11413' on 'Non−cancer illness code self−reported: anxiety/panic attacks || id:ukb−a−82'

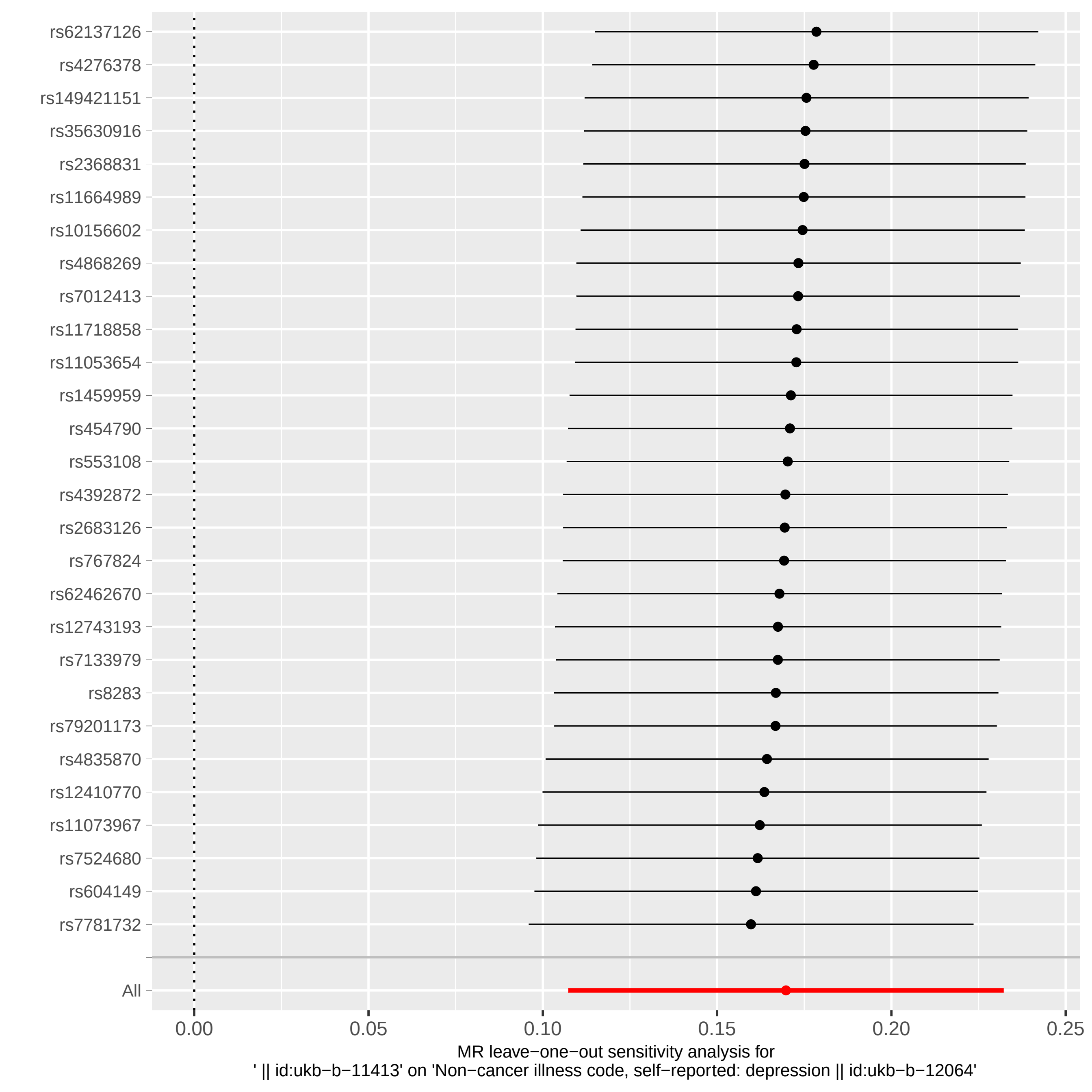
rs62137126
rs4276378
rs149421151
rs35630916
rs2368831
rs11664989
rs10156602
rs4868269
rs7012413
rs11718858
rs11053654
rs1459959
rs454790
rs553108
rs4392872
rs2683126
rs767824
rs62462670
rs12743193
rs7133979
rs8283
rs79201173
rs4835870
rs12410770
rs11073967
rs7524680
rs604149
rs7781732
All
0.00
0.05
0.10
0.15
0.20
0.25
MR leave−one−out sensitivity analysis for
' || id:ukb−b−11413' on 'Non−cancer illness code, self−reported: depression || id:ukb−b−12064'

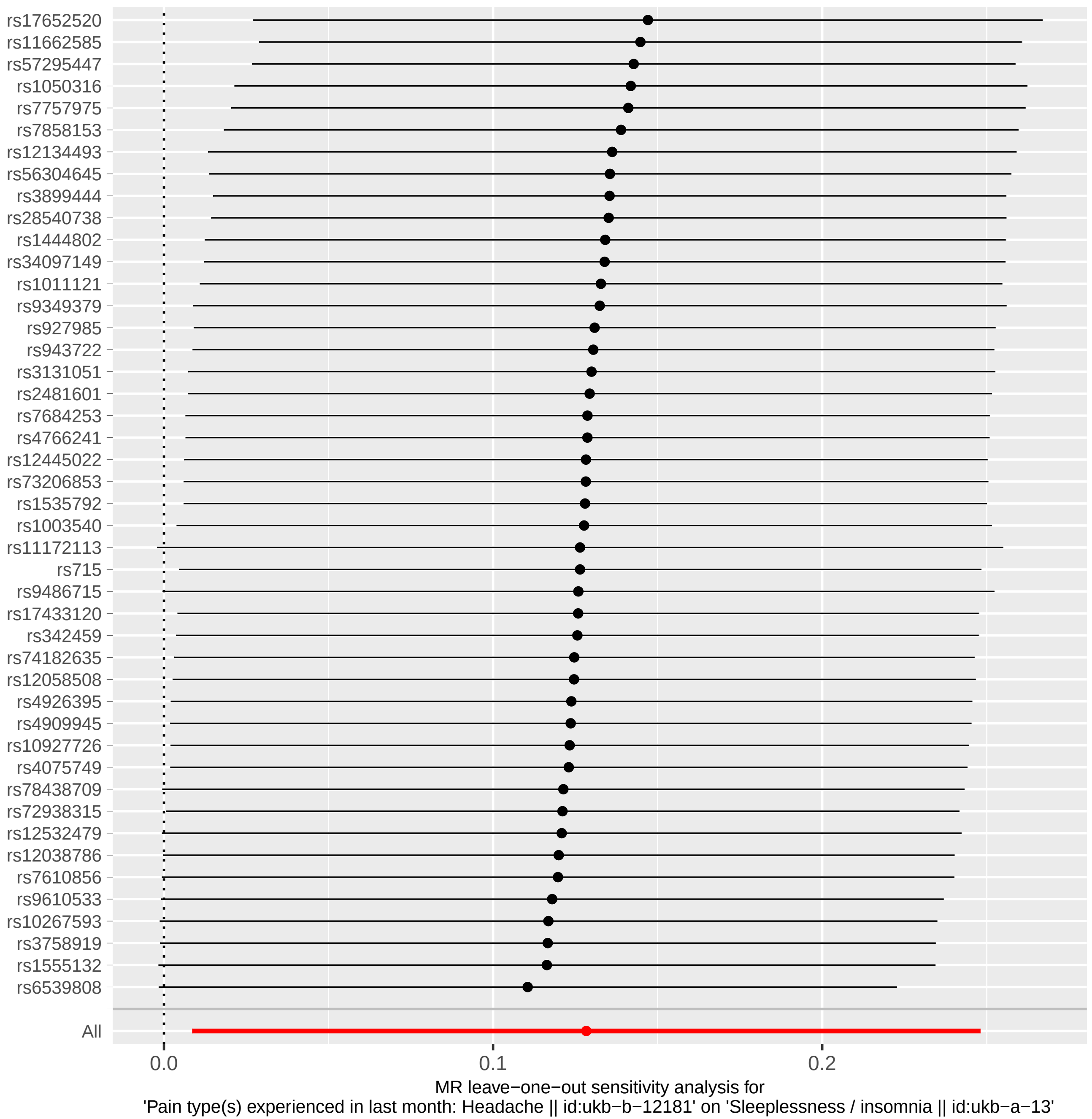

rs17652520
rs11662585
rs57295447
rs1050316
rs7757975
rs7858153
rs12134493
rs56304645
rs3899444
rs28540738
rs1444802
rs34097149
rs1011121
rs9349379
rs927985
rs943722
rs3131051
rs2481601
rs7684253
rs4766241
rs12445022
rs73206853
rs1535792
rs1003540
rs11172113
rs715
rs9486715
rs17433120
rs342459
rs74182635
rs12058508
rs4926395
rs4909945
rs10927726
rs4075749
rs78438709
rs72938315
rs12532479
rs12038786
rs7610856
rs9610533
rs10267593
rs3758919
rs1555132
rs6539808
All
0.0
0.1
0.2
MR leave−one−out sensitivity analysis for
'Pain type(s) experienced in last month: Headache || id:ukb−b−12181' on 'Sleeplessness / insomnia || id:ukb−a−13'

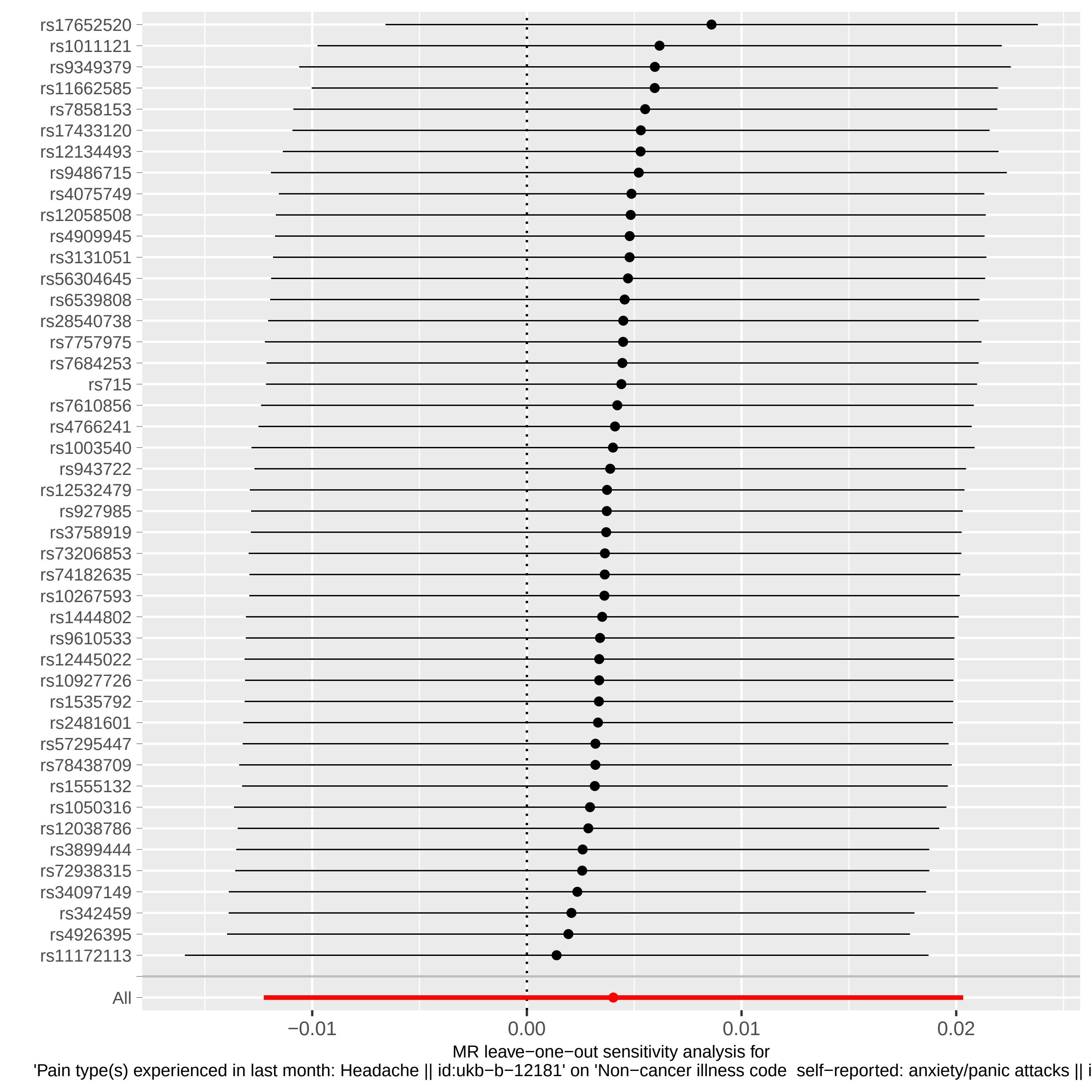
rs17652520
rs1011121
rs9349379
rs11662585
rs7858153
rs17433120
rs12134493
rs9486715
rs4075749
rs12058508
rs4909945
rs3131051
rs56304645
rs6539808
rs28540738
rs7757975
rs7684253
rs715
rs7610856
rs4766241
rs1003540
rs943722
rs12532479
rs927985
rs3758919
rs73206853
rs74182635
rs10267593
rs1444802
rs9610533
rs12445022
rs10927726
rs1535792
rs2481601
rs57295447
rs78438709
rs1555132
rs1050316
rs12038786
rs3899444
rs72938315
rs34097149
rs342459
rs4926395
rs11172113
All
−0.01
0.00
0.01
0.02
MR leave−one−out sensitivity analysis for
'Pain type(s) experienced in last month: Headache || id:ukb−b−12181' on 'Non−cancer illness code self−reported: anxiety/panic attacks || i

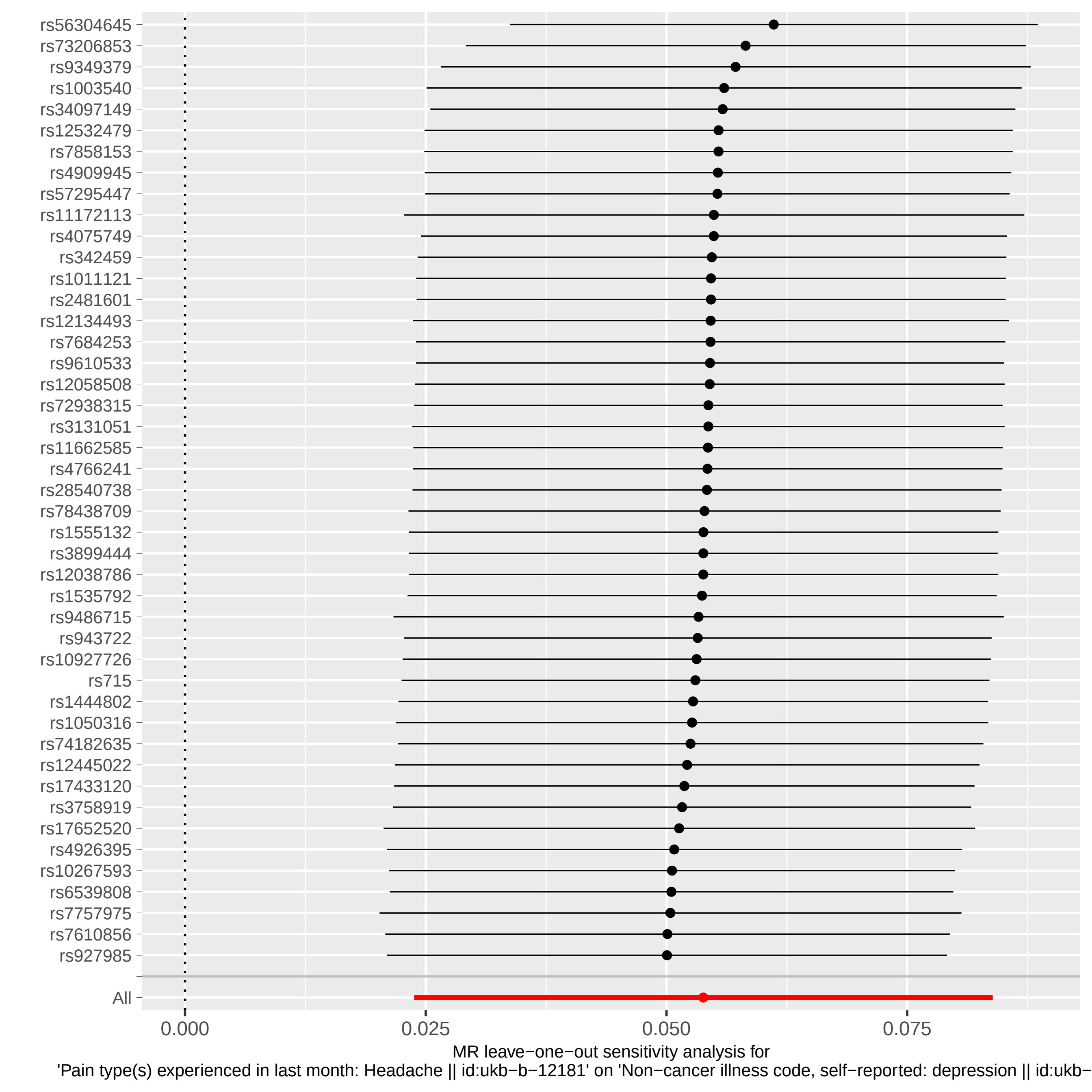
rs56304645
rs73206853
rs9349379
rs1003540
rs34097149
rs12532479
rs7858153
rs4909945
rs57295447
rs11172113
rs4075749
rs342459
rs1011121
rs2481601
rs12134493
rs7684253
rs9610533
rs12058508
rs72938315
rs3131051
rs11662585
rs4766241
rs28540738
rs78438709
rs1555132
rs3899444
rs12038786
rs1535792
rs9486715
rs943722
rs10927726
rs715
rs1444802
rs1050316
rs74182635
rs12445022
rs17433120
rs3758919
rs17652520
rs4926395
rs10267593
rs6539808
rs7757975
rs7610856
rs927985
All
0.000
0.025
0.050
0.075
MR leave−one−out sensitivity analysis for
'Pain type(s) experienced in last month: Headache || id:ukb−b−12181' on 'Non−cancer illness code, self−reported: depression || id:ukb−

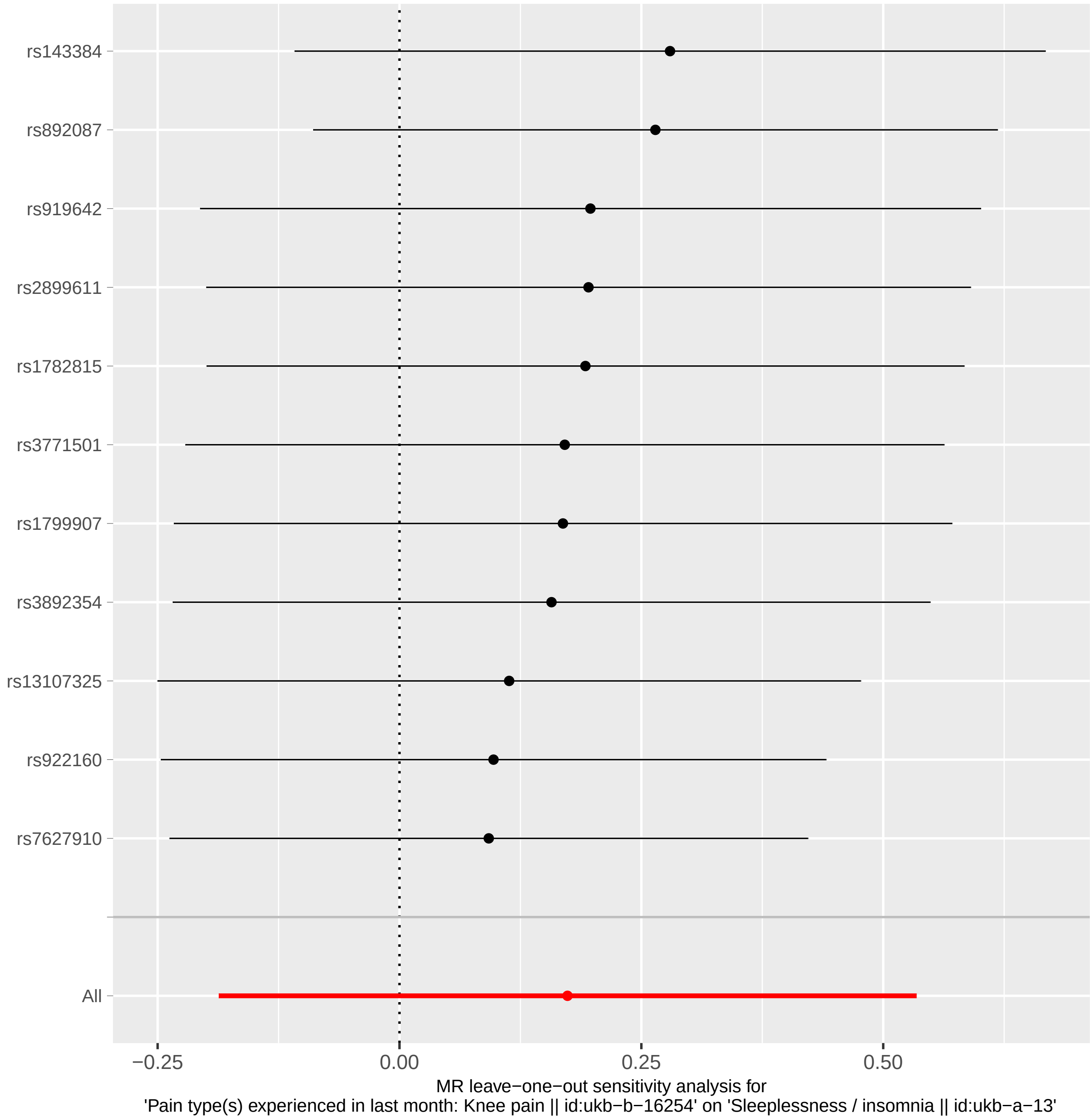
rs143384
rs892087
rs919642
rs2899611
rs1782815
rs3771501
rs1799907
rs3892354
rs13107325
rs922160
rs7627910
All
−0.25
0.00
0.25
0.50
MR leave−one−out sensitivity analysis for
'Pain type(s) experienced in last month: Knee pain || id:ukb−b−16254' on 'Sleeplessness / insomnia || id:ukb−a−13'

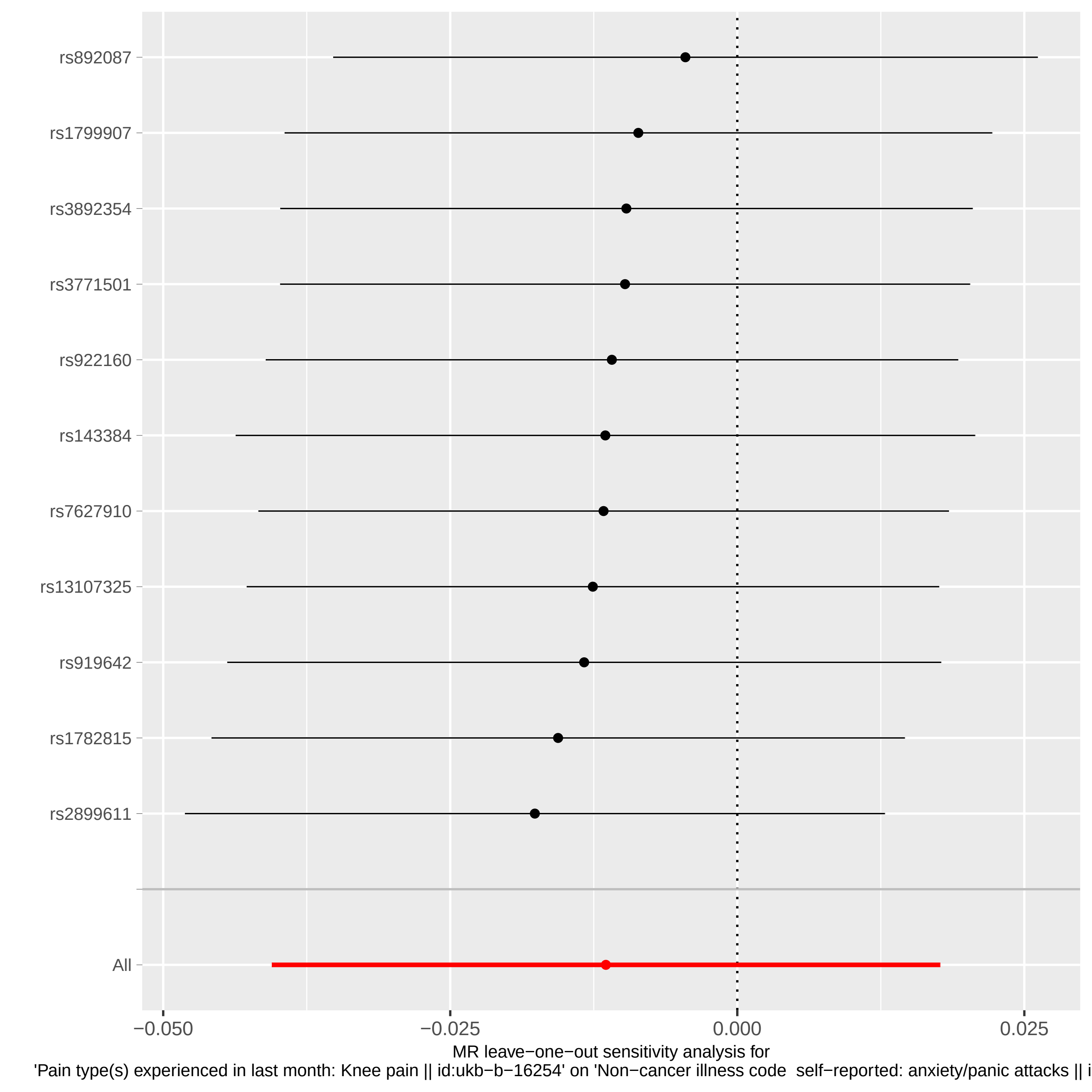

rs892087
rs1799907
rs3892354
rs3771501
rs922160
rs143384
rs7627910
rs13107325
rs919642
rs1782815
rs2899611
All
−0.050
−0.025
0.000
0.025
MR leave−one−out sensitivity analysis for
'Pain type(s) experienced in last month: Knee pain || id:ukb−b−16254' on 'Non−cancer illness code self−reported: anxiety/panic attacks || i

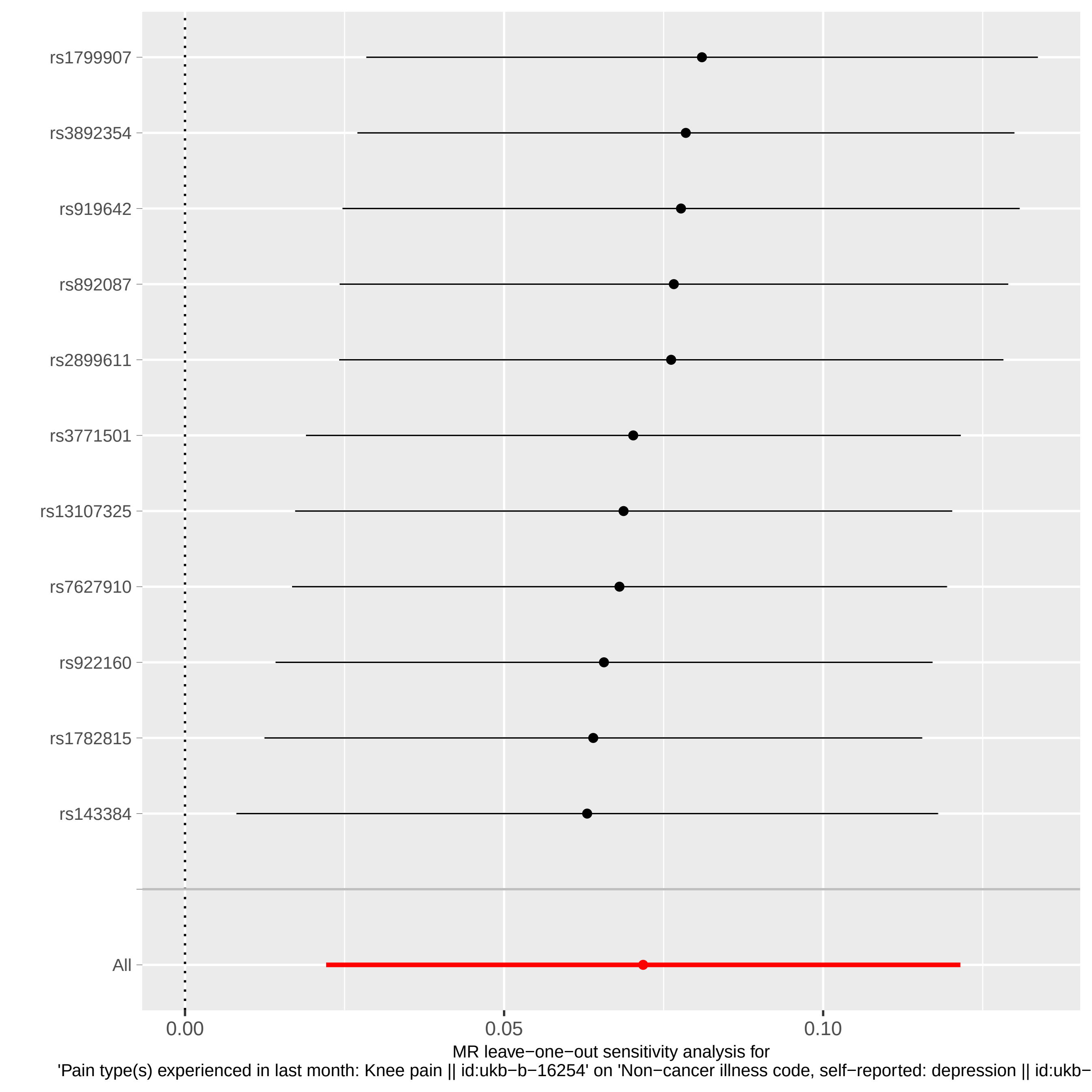

rs1799907
rs3892354
rs919642
rs892087
rs2899611
rs3771501
rs13107325
rs7627910
rs922160
rs1782815
rs143384
All
0.00
0.05
0.10
MR leave−one−out sensitivity analysis for
'Pain type(s) experienced in last month: Knee pain || id:ukb−b−16254' on 'Non−cancer illness code, self−reported: depression || id:ukb−

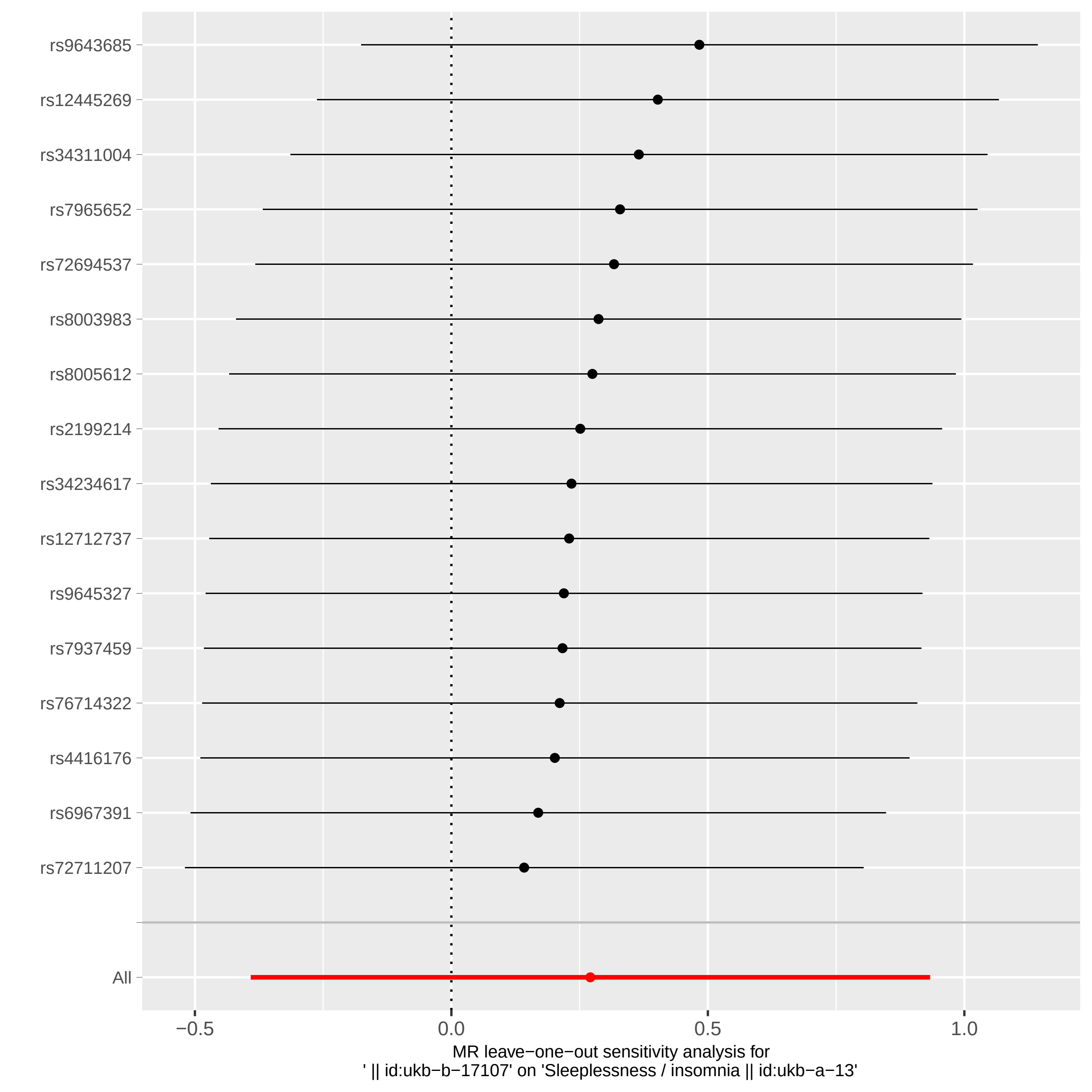
rs9643685
rs12445269
rs34311004
rs7965652
rs72694537
rs8003983
rs8005612
rs2199214
rs34234617
rs12712737
rs9645327
rs7937459
rs76714322
rs4416176
rs6967391
rs72711207
All
−0.5
0.0
0.5
1.0
MR leave−one−out sensitivity analysis for
' || id:ukb−b−17107' on 'Sleeplessness / insomnia || id:ukb−a−13'

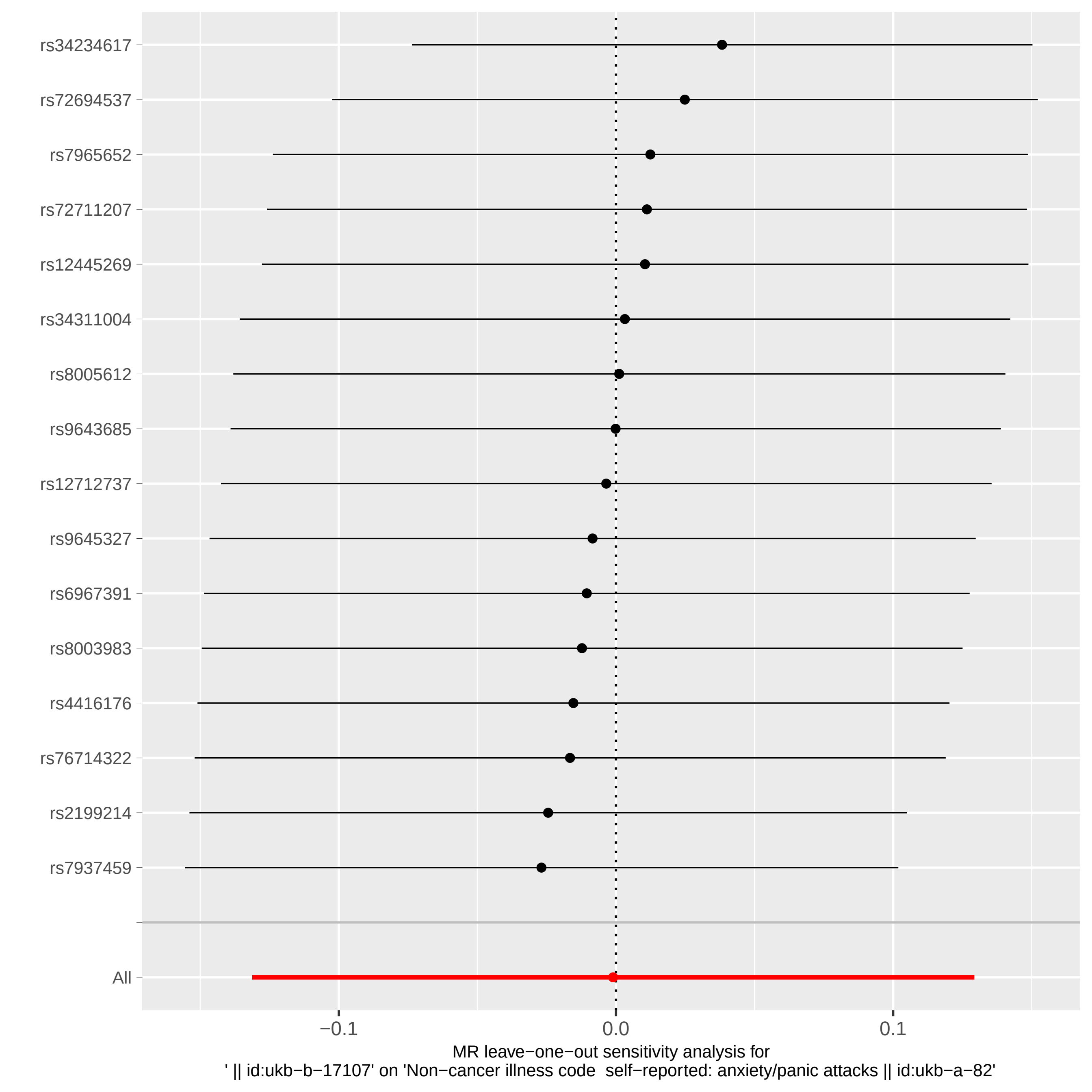

rs34234617
rs72694537
rs7965652
rs72711207
rs12445269
rs34311004
rs8005612
rs9643685
rs12712737
rs9645327
rs6967391
rs8003983
rs4416176
rs76714322
rs2199214
rs7937459
All
−0.1
0.0
0.1
MR leave−one−out sensitivity analysis for
' || id:ukb−b−17107' on 'Non−cancer illness code self−reported: anxiety/panic attacks || id:ukb−a−82'

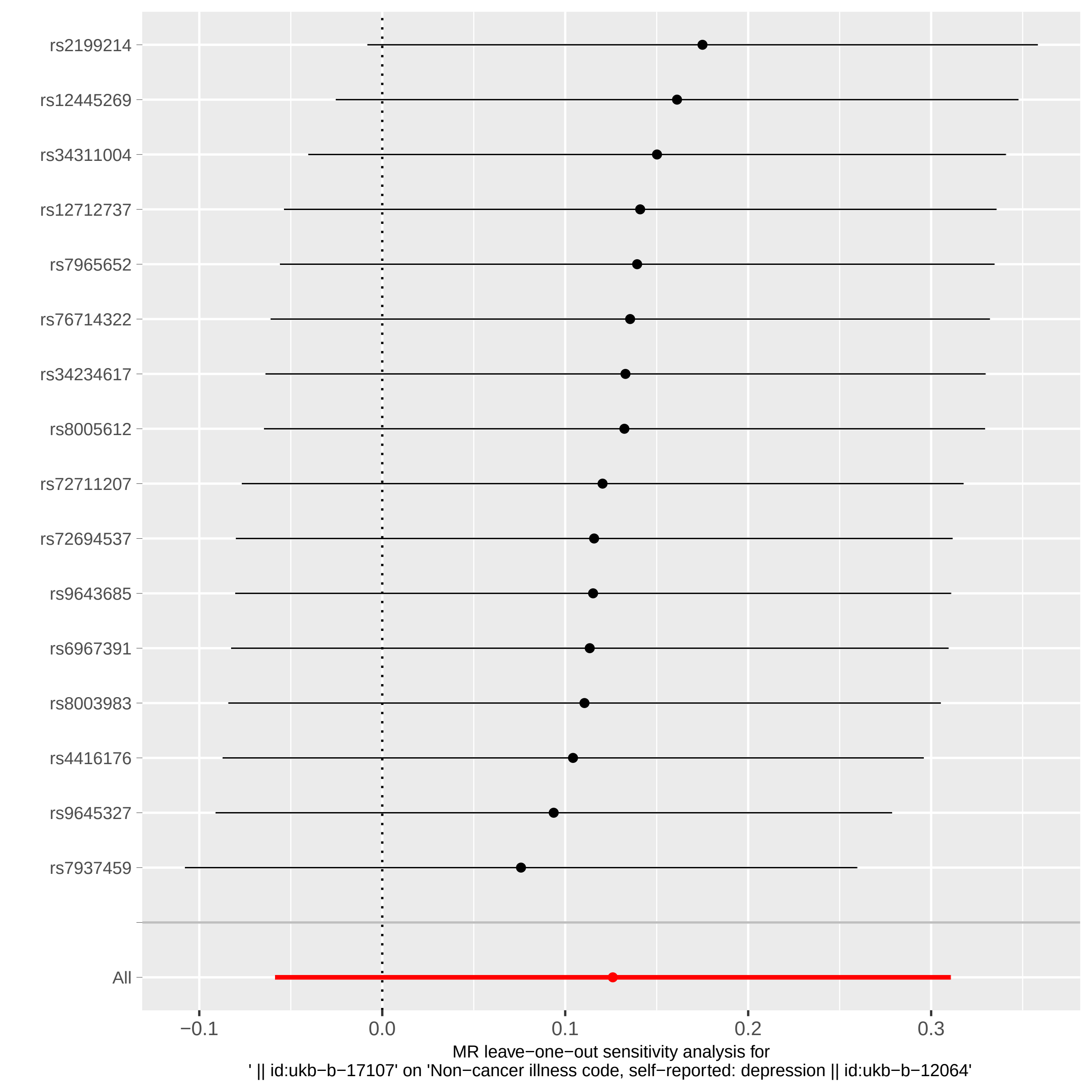
rs2199214
rs12445269
rs34311004
rs12712737
rs7965652
rs76714322
rs34234617
rs8005612
rs72711207
rs72694537
rs9643685
rs6967391
rs8003983
rs4416176
rs9645327
rs7937459
All
−0.1
0.0
0.1
0.2
0.3
MR leave−one−out sensitivity analysis for
' || id:ukb−b−17107' on 'Non−cancer illness code, self−reported: depression || id:ukb−b−12064'

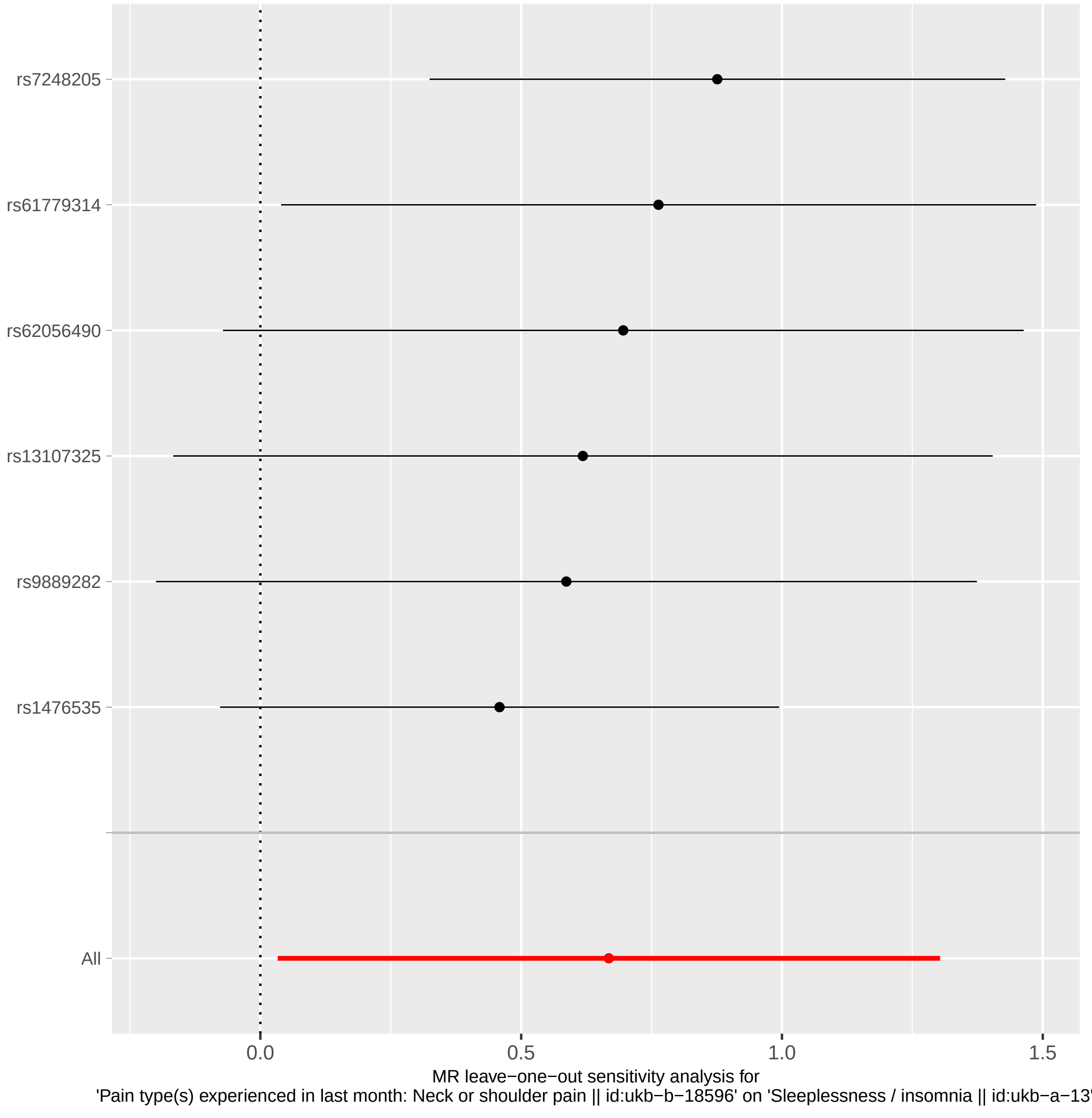
rs7248205
rs61779314
rs62056490
rs13107325
rs9889282
rs1476535
All
0.0
0.5
1.0
1.5
MR leave−one−out sensitivity analysis for
'Pain type(s) experienced in last month: Neck or shoulder pain || id:ukb−b−18596' on 'Sleeplessness / insomnia || id:ukb−a−13

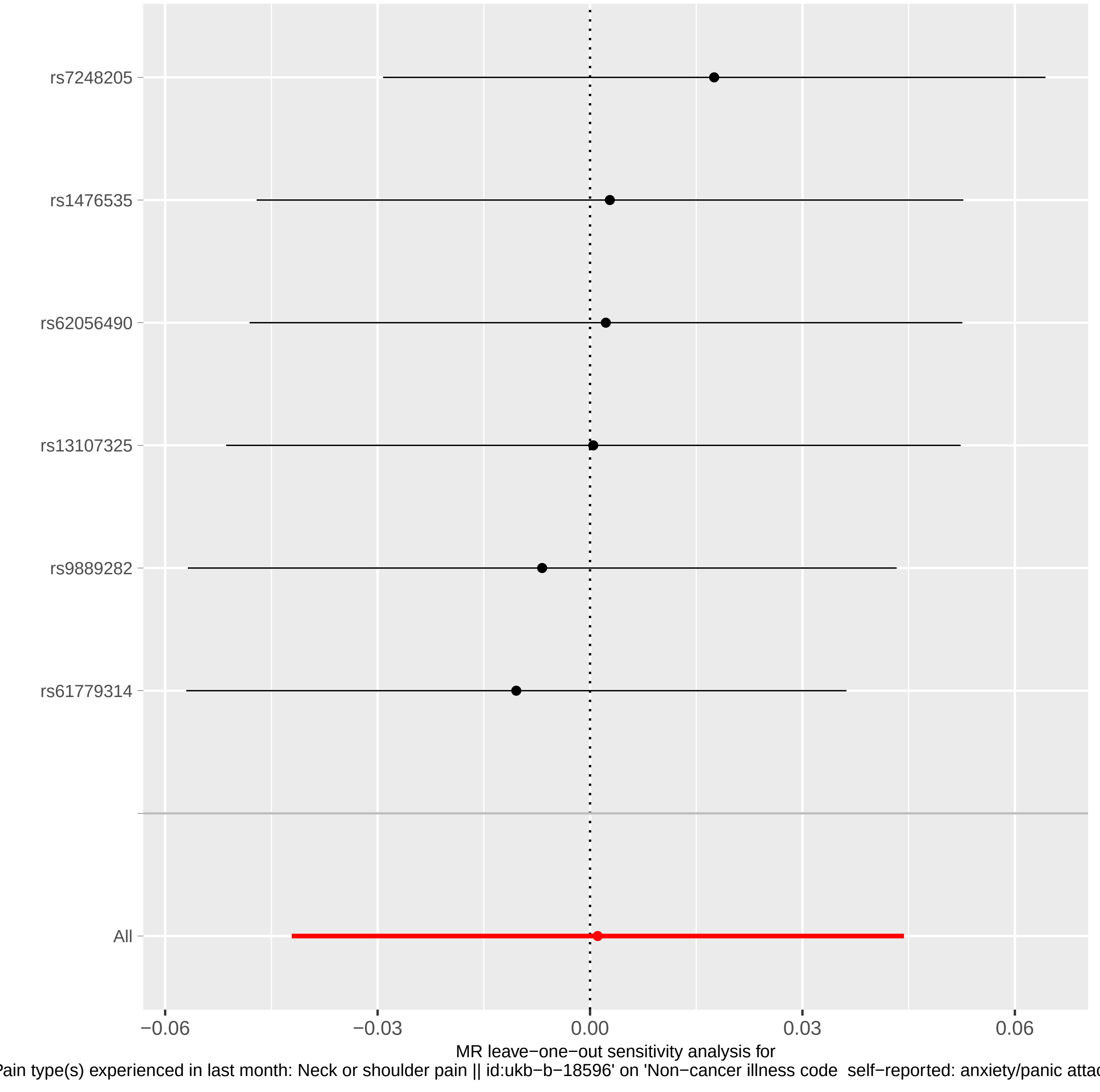

rs7248205
rs1476535
rs62056490
rs13107325
rs9889282
rs61779314
All
−0.06
−0.03
0.00
0.03
0.06
MR leave−one−out sensitivity analysis for
Pain type(s) experienced in last month: Neck or shoulder pain || id:ukb−b−18596' on 'Non−cancer illness code self−reported: anxiety/panic attac

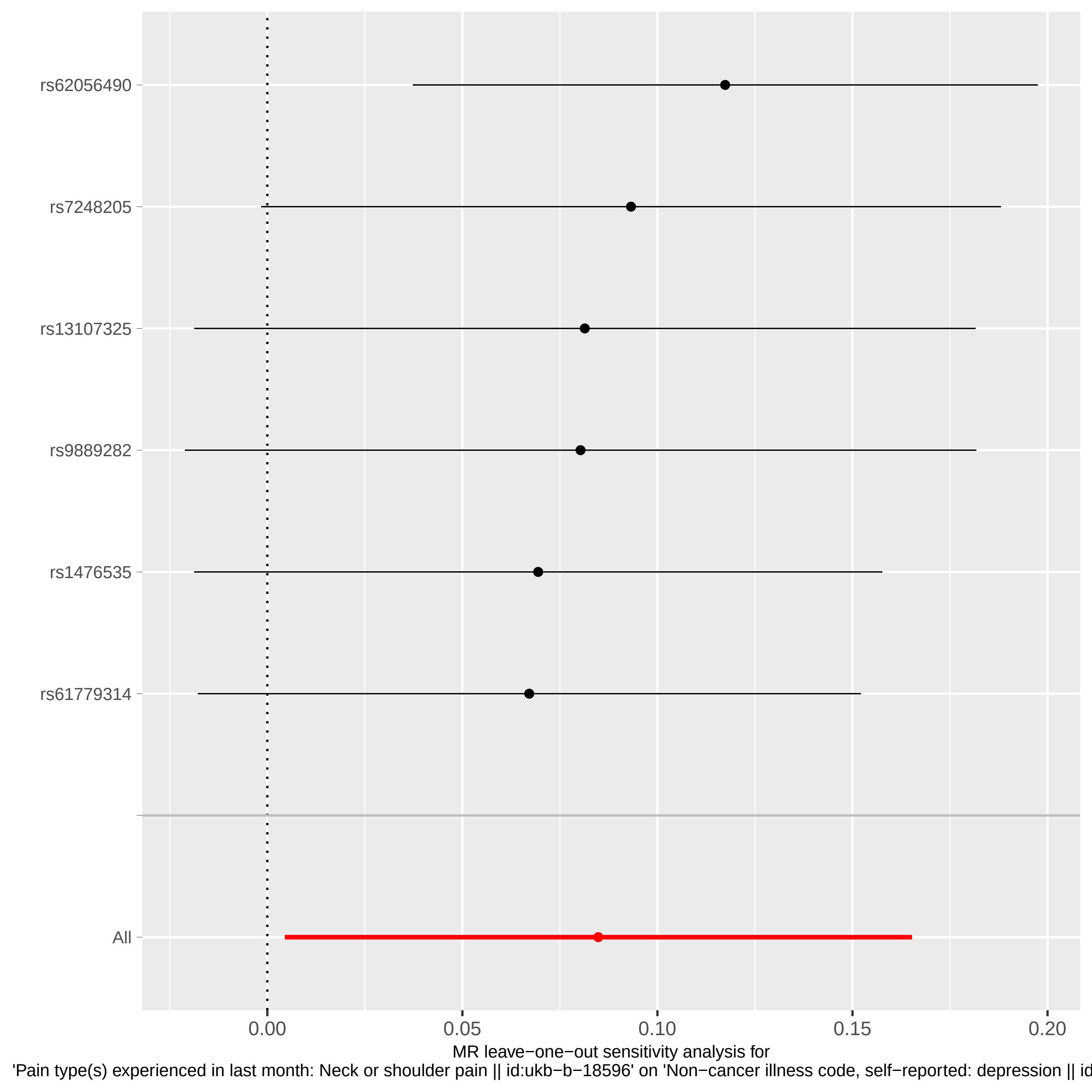
rs62056490
rs7248205
rs13107325
rs9889282
rs1476535
rs61779314
All
0.00
0.05
0.10
0.15
0.20
MR leave−one−out sensitivity analysis for
'Pain type(s) experienced in last month: Neck or shoulder pain || id:ukb−b−18596' on 'Non−cancer illness code, self−reported: depression || id

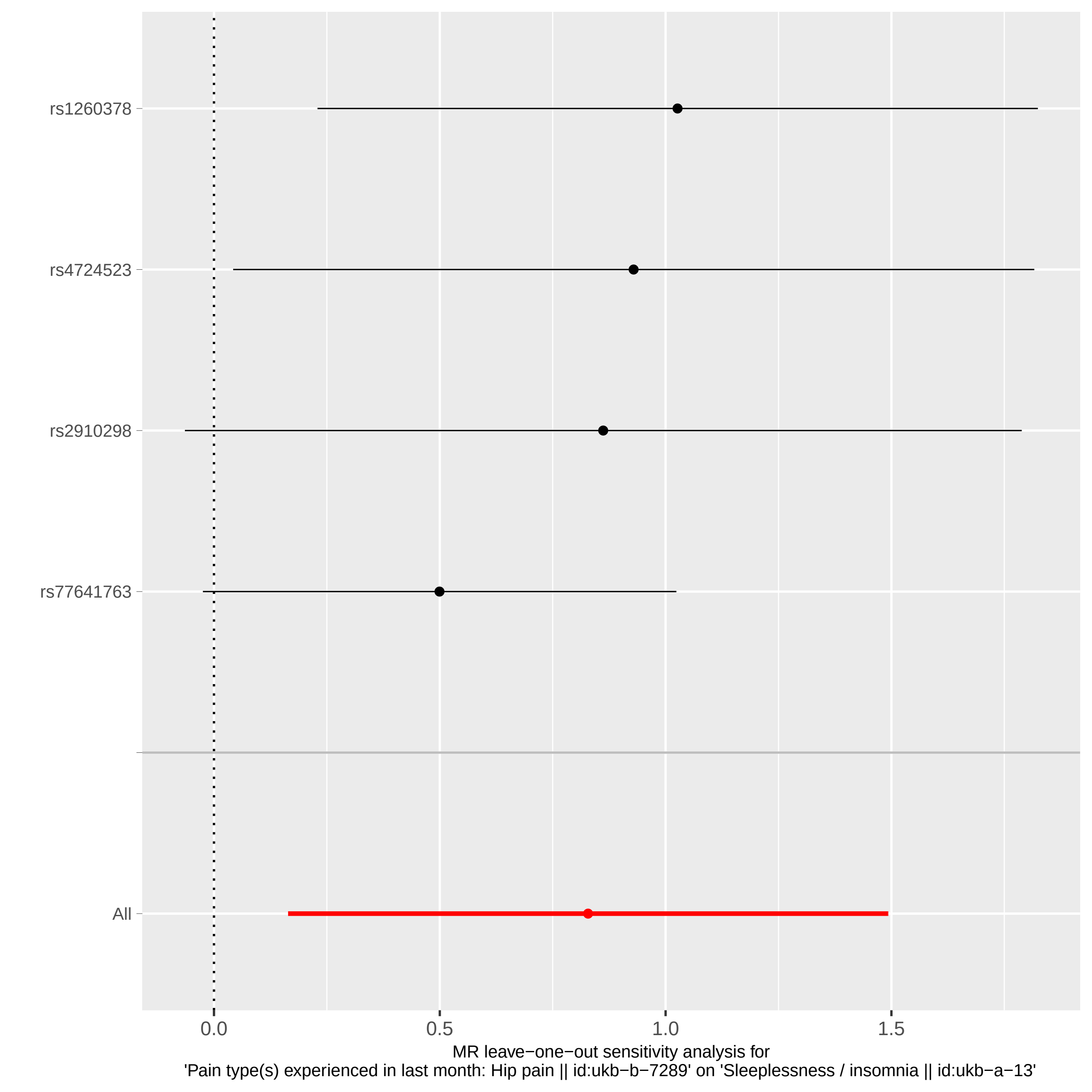
rs1260378
rs4724523
rs2910298
rs77641763
All
0.0
0.5
1.0
1.5
MR leave−one−out sensitivity analysis for
'Pain type(s) experienced in last month: Hip pain || id:ukb−b−7289' on 'Sleeplessness / insomnia || id:ukb−a−13'

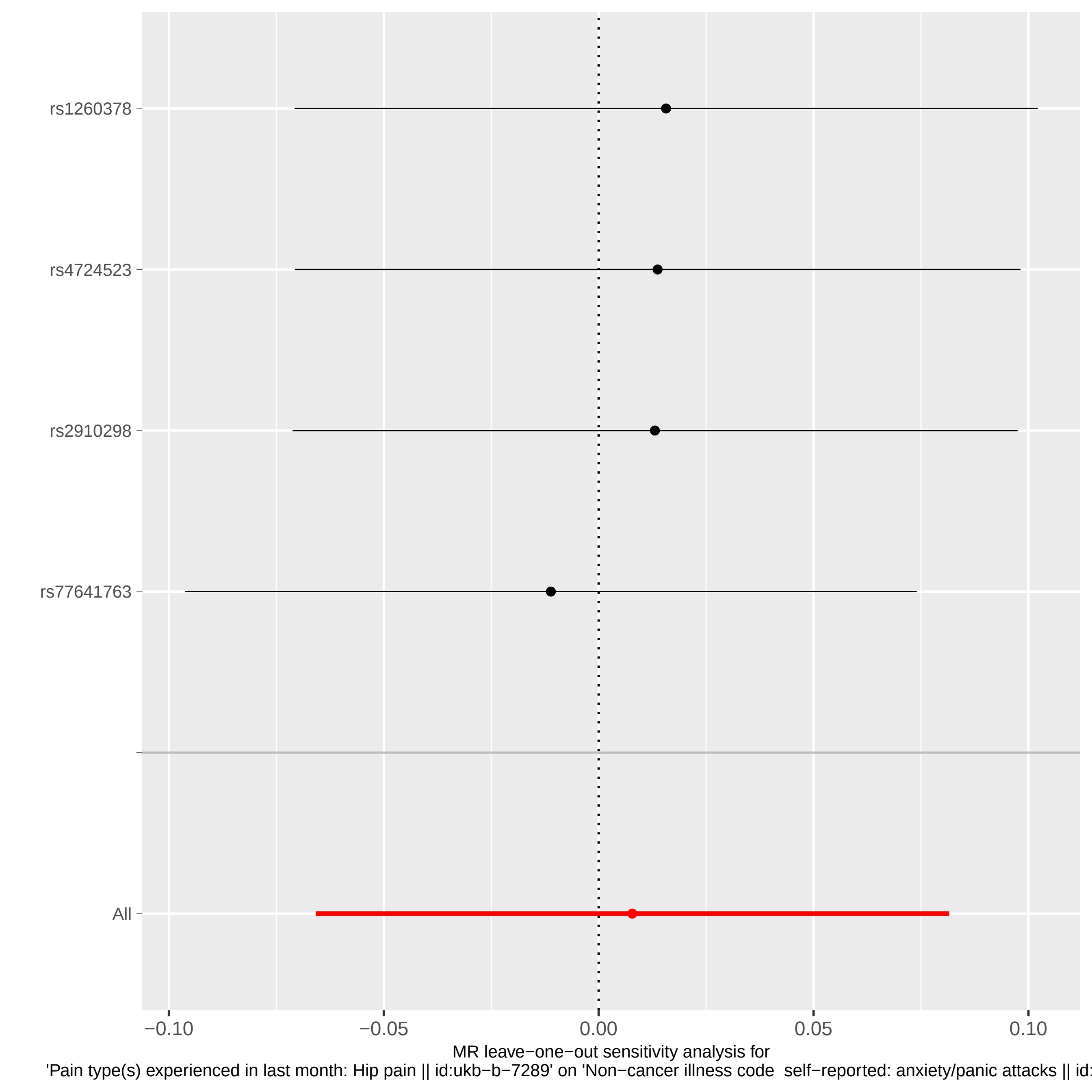

rs1260378
rs4724523
rs2910298
rs77641763
All
−0.10
−0.05
0.00
0.05
0.10
MR leave−one−out sensitivity analysis for
'Pain type(s) experienced in last month: Hip pain || id:ukb−b−7289' on 'Non−cancer illness code self−reported: anxiety/panic attacks || id:

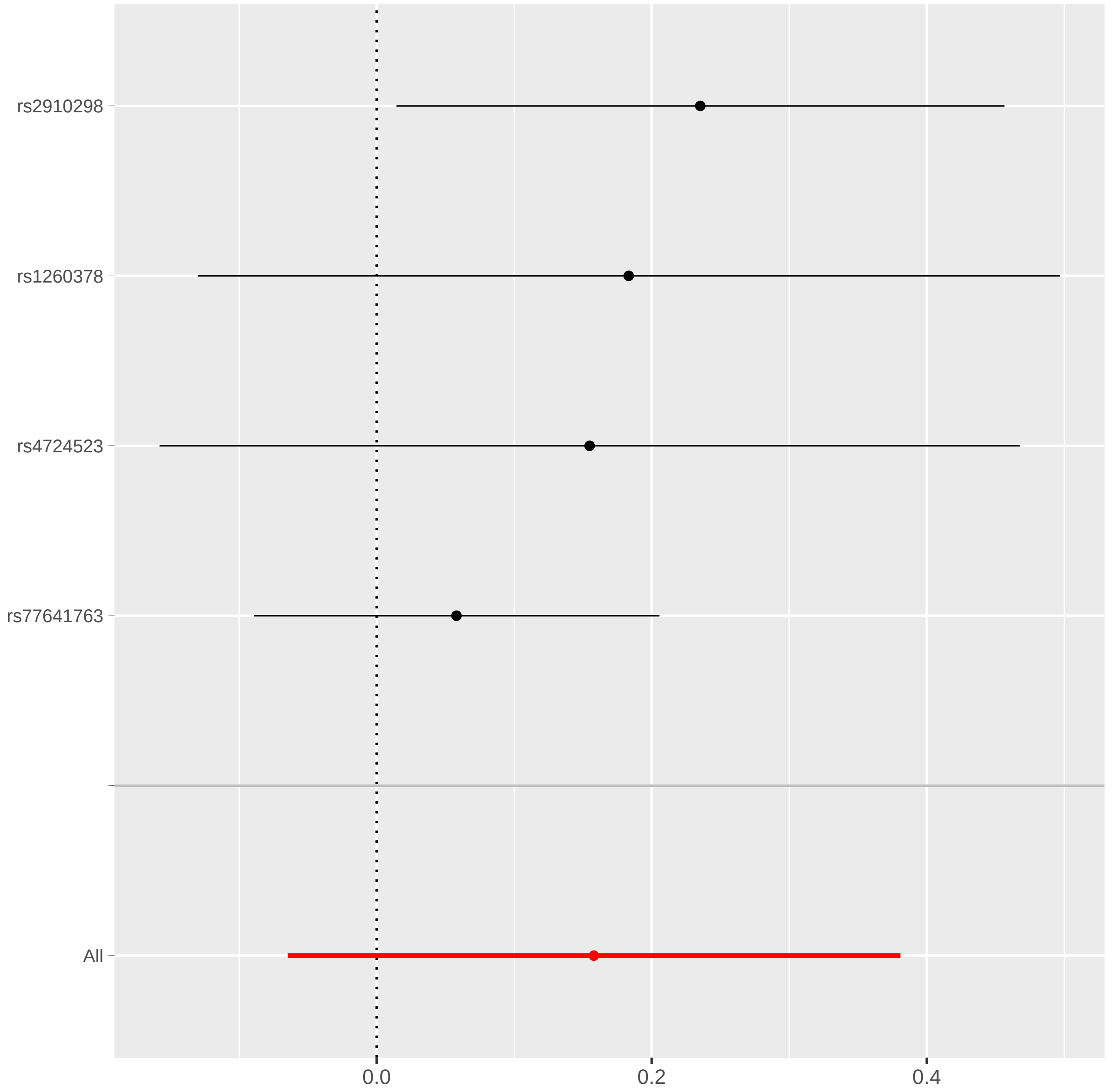

MR leave−one−out sensitivity analysis for
'Pain type(s) experienced in last month: Hip pain || id:ukb−b−7289' on 'Non−cancer illness code, self−reported: depression || id:ukb−b-

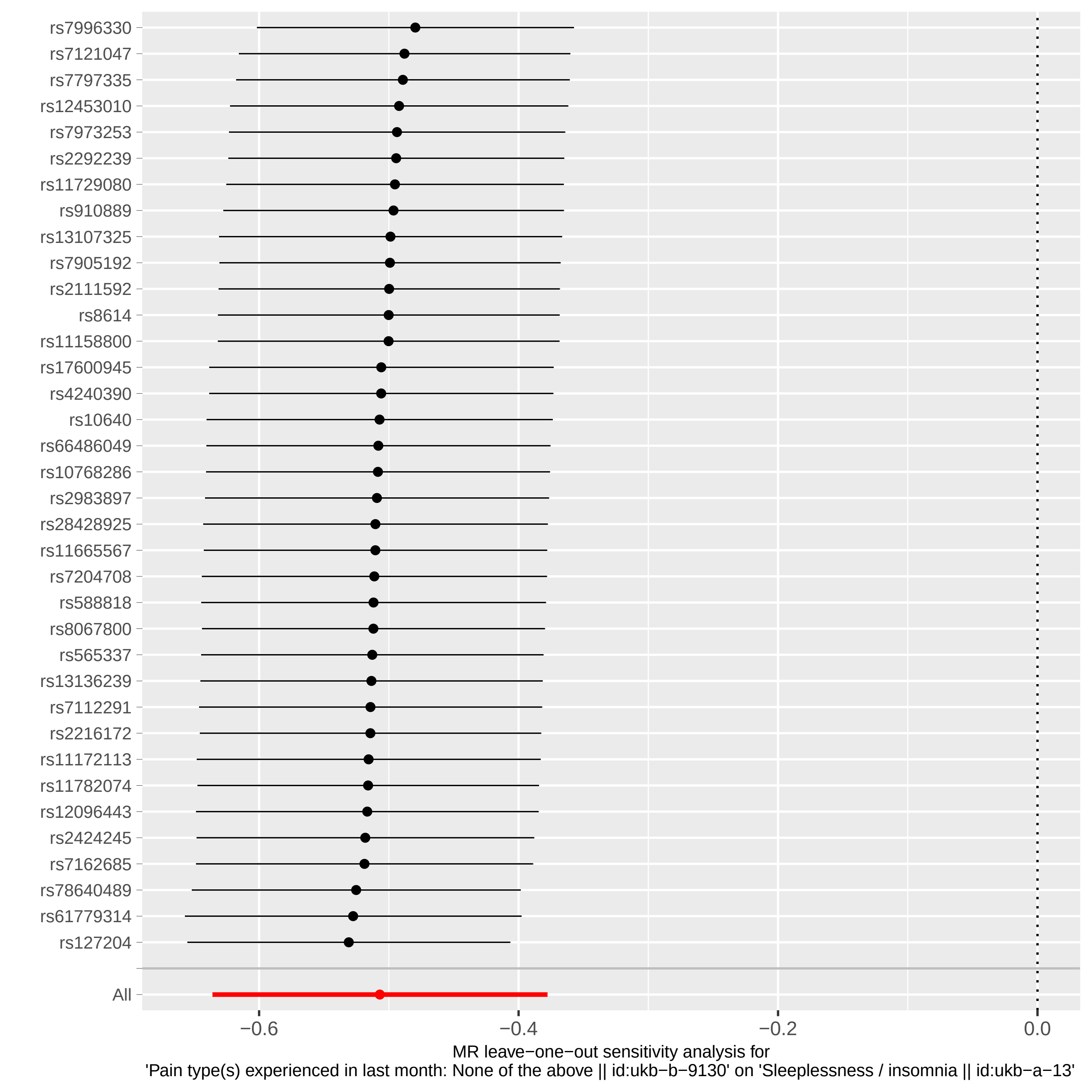
rs7996330
rs7121047
rs7797335
rs12453010
rs7973253
rs2292239
rs11729080
rs910889
rs13107325
rs7905192
rs2111592
rs8614
rs11158800
rs17600945
rs4240390
rs10640
rs66486049
rs10768286
rs2983897
rs28428925
rs11665567
rs7204708
rs588818
rs8067800
rs565337
rs13136239
rs7112291
rs2216172
rs11172113
rs11782074
rs12096443
rs2424245
rs7162685
rs78640489
rs61779314
rs127204
All
−0.6
−0.4
−0.2
0.0
MR leave−one−out sensitivity analysis for
'Pain type(s) experienced in last month: None of the above || id:ukb−b−9130' on 'Sleeplessness / insomnia || id:ukb−a−13'

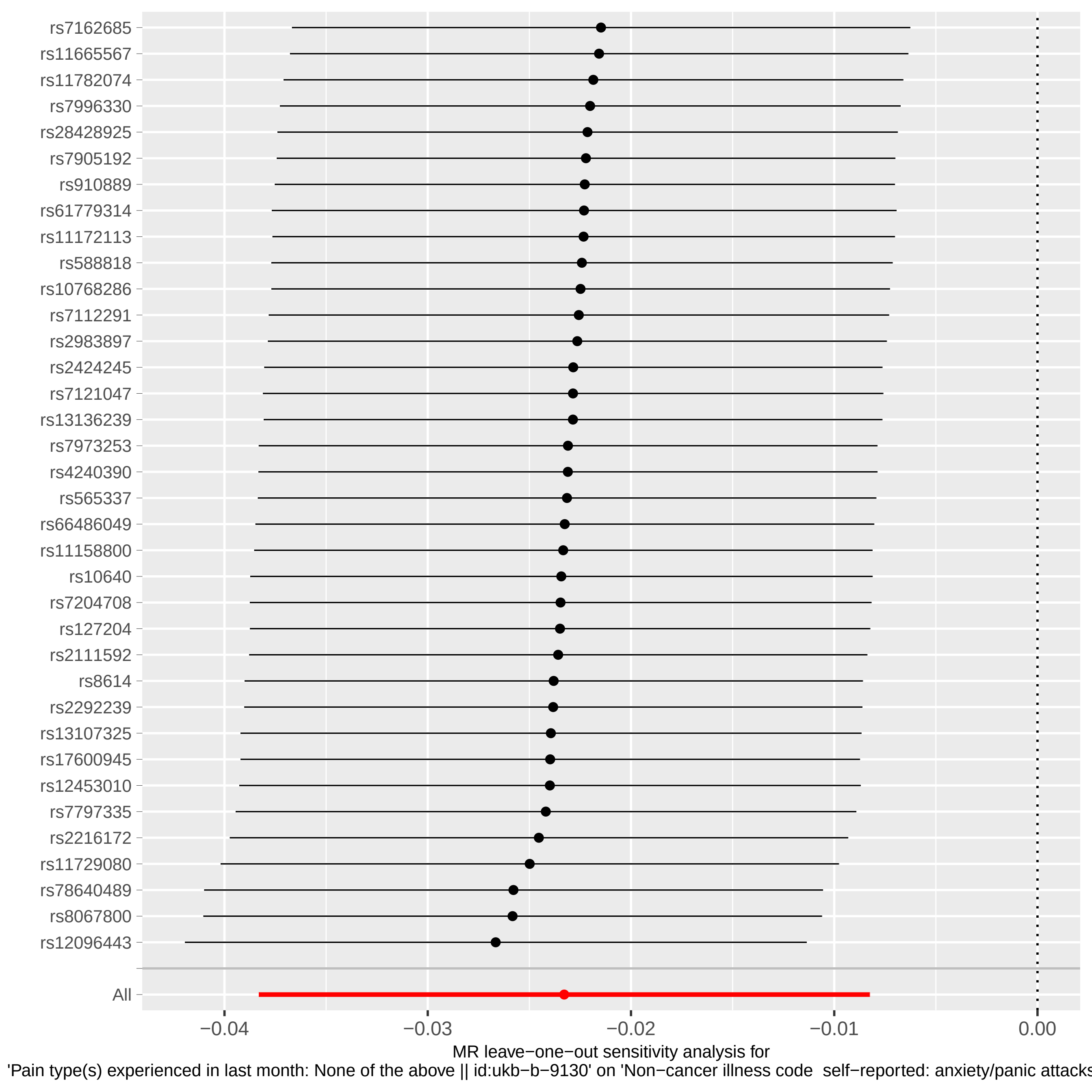

rs7162685
rs11665567
rs11782074
rs7996330
rs28428925
rs7905192
rs910889
rs61779314
rs11172113
rs588818
rs10768286
rs7112291
rs2983897
rs2424245
rs7121047
rs13136239
rs7973253
rs4240390
rs565337
rs66486049
rs11158800
rs10640
rs7204708
rs127204
rs2111592
rs8614
rs2292239
rs13107325
rs17600945
rs12453010
rs7797335
rs2216172
rs11729080
rs78640489
rs8067800
rs12096443
All
−0.04
−0.03
−0.02
−0.01
0.00
MR leave−one−out sensitivity analysis for
'Pain type(s) experienced in last month: None of the above || id:ukb−b−9130' on 'Non−cancer illness code self−reported: anxiety/panic attacks

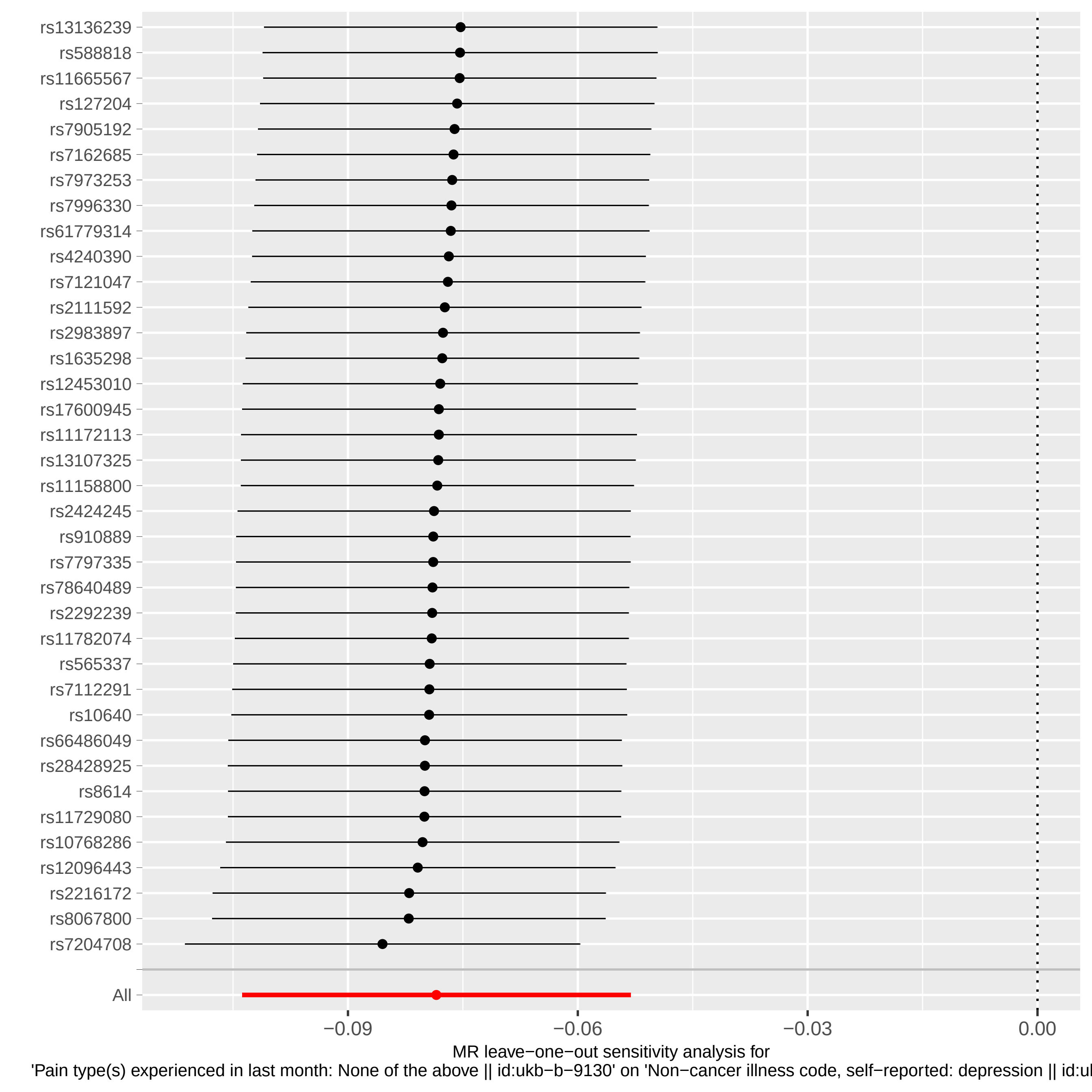
rs13136239
rs588818
rs11665567
rs127204
rs7905192
rs7162685
rs7973253
rs7996330
rs61779314
rs4240390
rs7121047
rs2111592
rs2983897
rs1635298
rs12453010
rs17600945
rs11172113
rs13107325
rs11158800
rs2424245
rs910889
rs7797335
rs78640489
rs2292239
rs11782074
rs565337
rs7112291
rs10640
rs66486049
rs28428925
rs8614
rs11729080
rs10768286
rs12096443
rs2216172
rs8067800
rs7204708
All
−0.09
−0.06
−0.03
0.00
MR leave−one−out sensitivity analysis for
'Pain type(s) experienced in last month: None of the above || id:ukb−b−9130' on 'Non−cancer illness code, self−reported: depression || id:uk

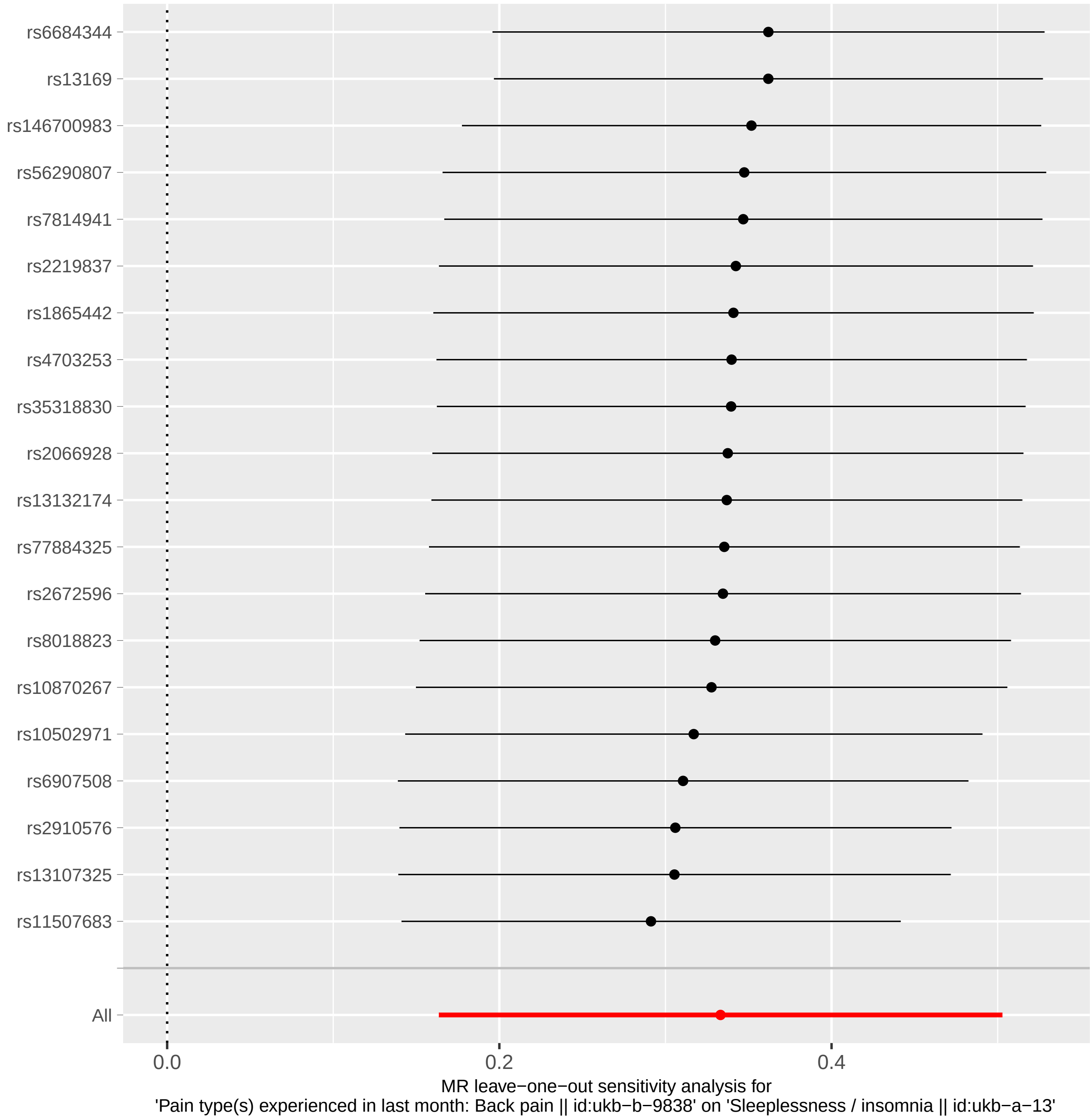
rs6684344
rs13169
rs146700983
rs56290807
rs7814941
rs2219837
rs1865442
rs4703253
rs35318830
rs2066928
rs13132174
rs77884325
rs2672596
rs8018823
rs10870267
rs10502971
rs6907508
rs2910576
rs13107325
rs11507683
All
0.0
0.2
0.4
MR leave−one−out sensitivity analysis for
'Pain type(s) experienced in last month: Back pain || id:ukb−b−9838' on 'Sleeplessness / insomnia || id:ukb−a−13'

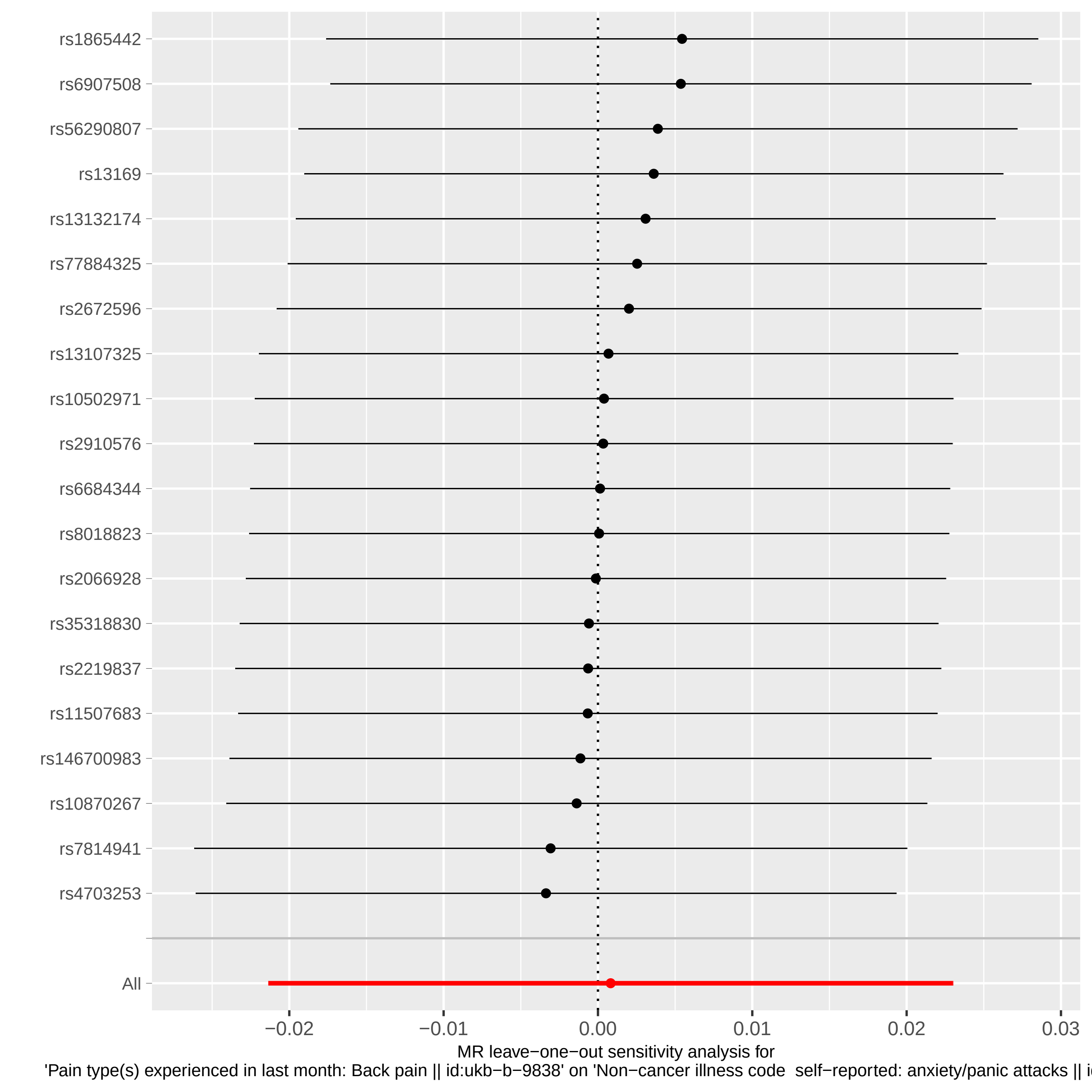
rs1865442
rs6907508
rs56290807
rs13169
rs13132174
rs77884325
rs2672596
rs13107325
rs10502971
rs2910576
rs6684344
rs8018823
rs2066928
rs35318830
rs2219837
rs11507683
rs146700983
rs10870267
rs7814941
rs4703253
All
−0.02
−0.01
0.00
0.01
0.02
0.03
MR leave−one−out sensitivity analysis for
'Pain type(s) experienced in last month: Back pain || id:ukb−b−9838' on 'Non−cancer illness code self−reported: anxiety/panic attacks || i

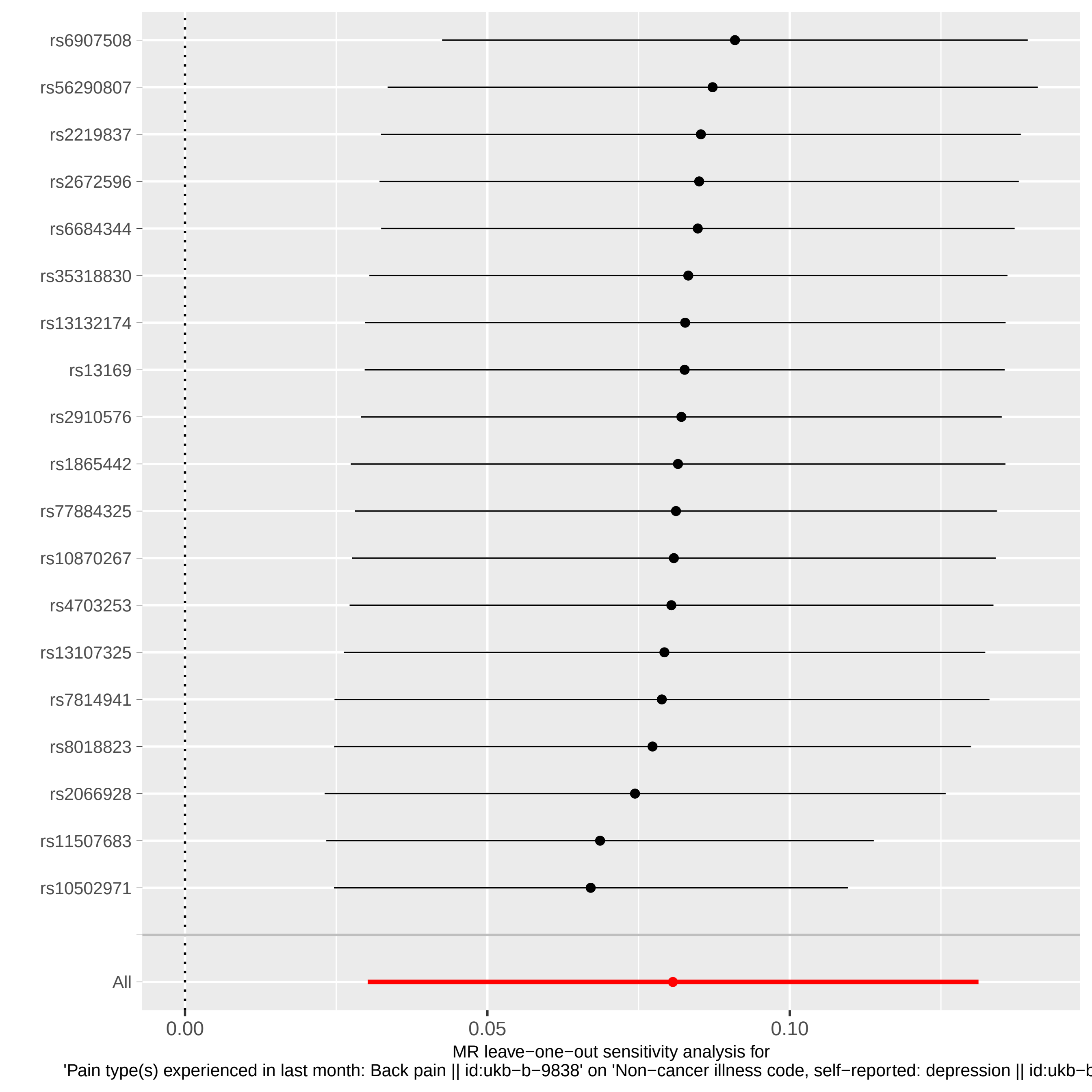
rs6907508
rs56290807
rs2219837
rs2672596
rs6684344
rs35318830
rs13132174
rs13169
rs2910576
rs1865442
rs77884325
rs10870267
rs4703253
rs13107325
rs7814941
rs8018823
rs2066928
rs11507683
rs10502971
All
0.00
0.05
0.10
MR leave−one−out sensitivity analysis for
'Pain type(s) experienced in last month: Back pain || id:ukb−b−9838' on 'Non−cancer illness code, self−reported: depression || id:ukb−b

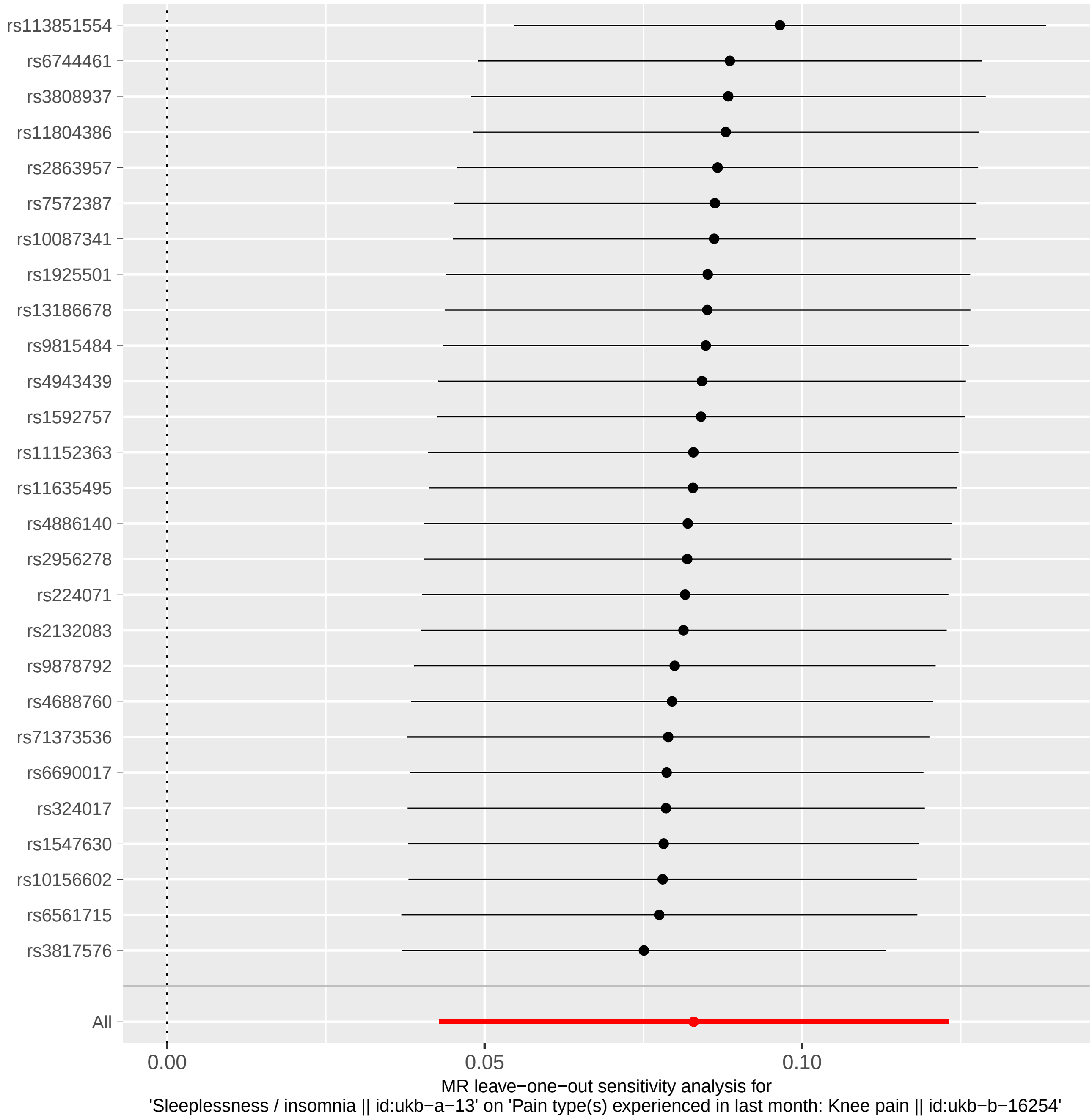
rs113851554
rs6744461
rs3808937
rs11804386
rs2863957
rs7572387
rs10087341
rs1925501
rs13186678
rs9815484
rs4943439
rs1592757
rs11152363
rs11635495
rs4886140
rs2956278
rs224071
rs2132083
rs9878792
rs4688760
rs71373536
rs6690017
rs324017
rs1547630
rs10156602
rs6561715
rs3817576
All
0.00
0.05
0.10
MR leave−one−out sensitivity analysis for
'Sleeplessness / insomnia || id:ukb−a−13' on 'Pain type(s) experienced in last month: Knee pain || id:ukb−b−16254'

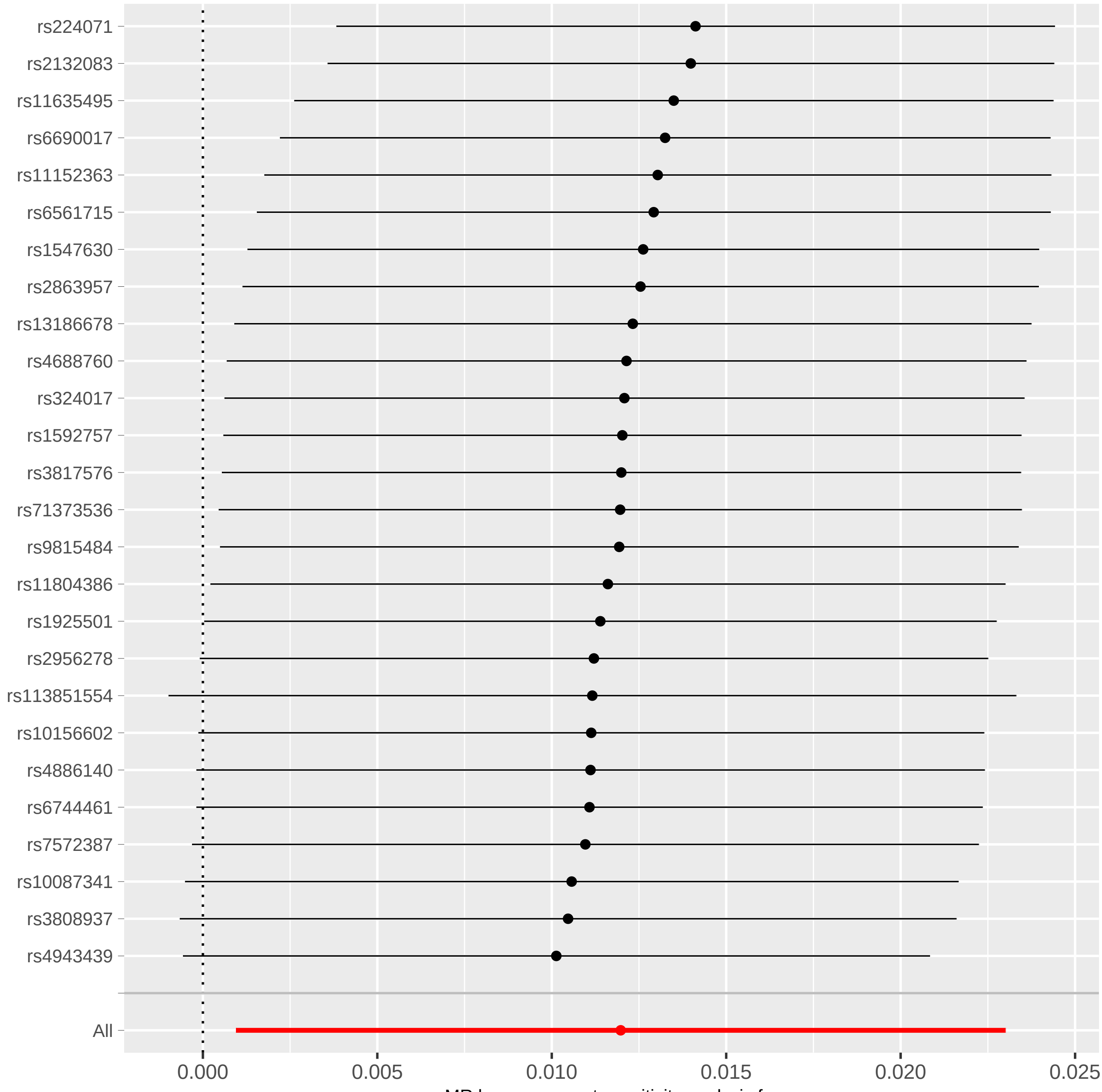

MR leave−one−out sensitivity analysis for
'Sleeplessness / insomnia || id:ukb−a−13' on 'Pain type(s) experienced in last month: Facial pain || id:ukb−b−17107'

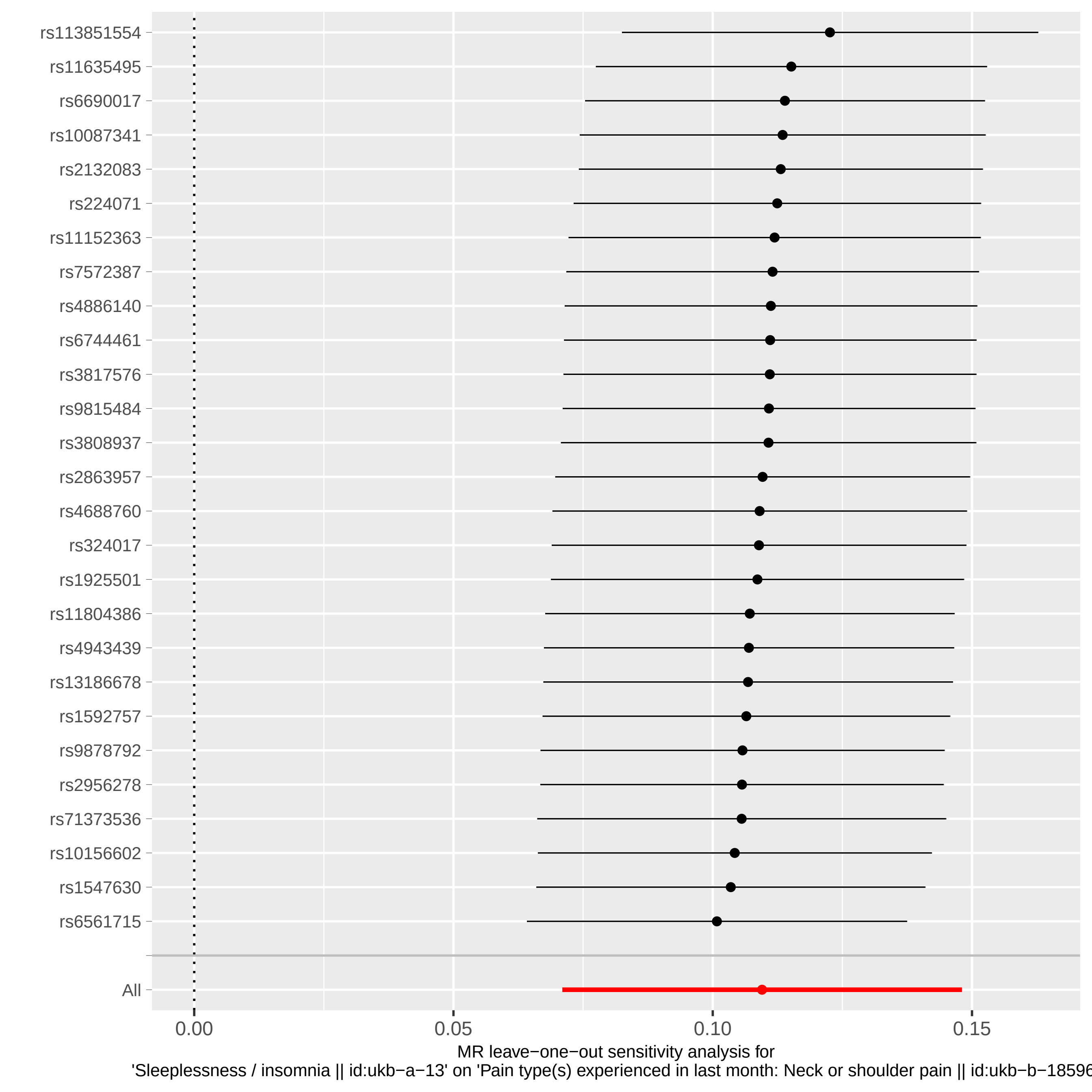

rs113851554
rs11635495
rs6690017
rs10087341
rs2132083
rs224071
rs11152363
rs7572387
rs4886140
rs6744461
rs3817576
rs9815484
rs3808937
rs2863957
rs4688760
rs324017
rs1925501
rs11804386
rs4943439
rs13186678
rs1592757
rs9878792
rs2956278
rs71373536
rs10156602
rs1547630
rs6561715
All
0.00
0.05
0.10
0.15
MR leave−one−out sensitivity analysis for
'Sleeplessness / insomnia || id:ukb−a−13' on 'Pain type(s) experienced in last month: Neck or shoulder pain || id:ukb−b−18596

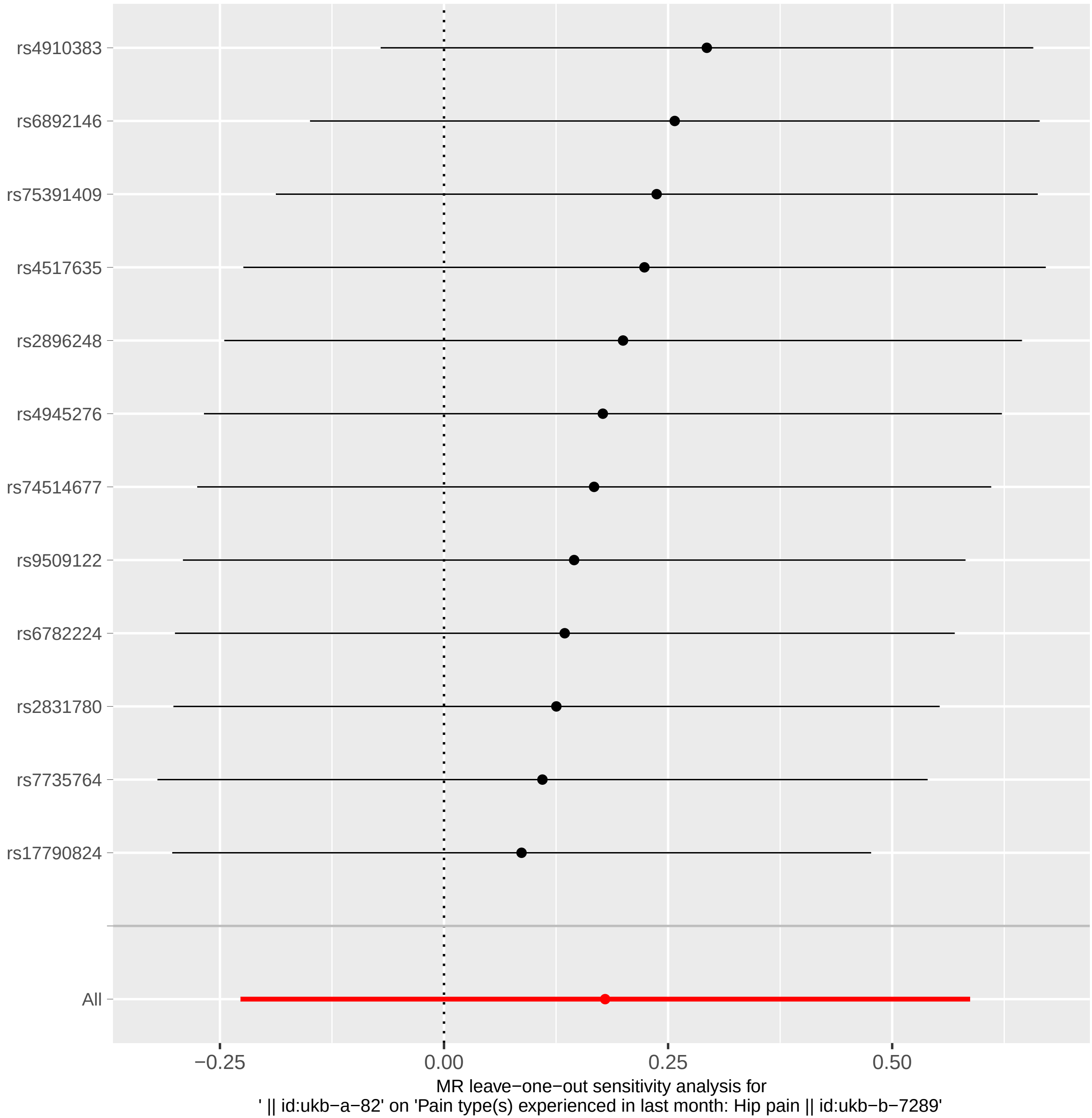

rs4910383
rs6892146
rs75391409
rs4517635
rs2896248
rs4945276
rs74514677
rs9509122
rs6782224
rs2831780
rs7735764
rs17790824
All
−0.25
0.00
0.25
0.50
MR leave−one−out sensitivity analysis for
' || id:ukb−a−82' on 'Pain type(s) experienced in last month: Hip pain || id:ukb−b−7289'

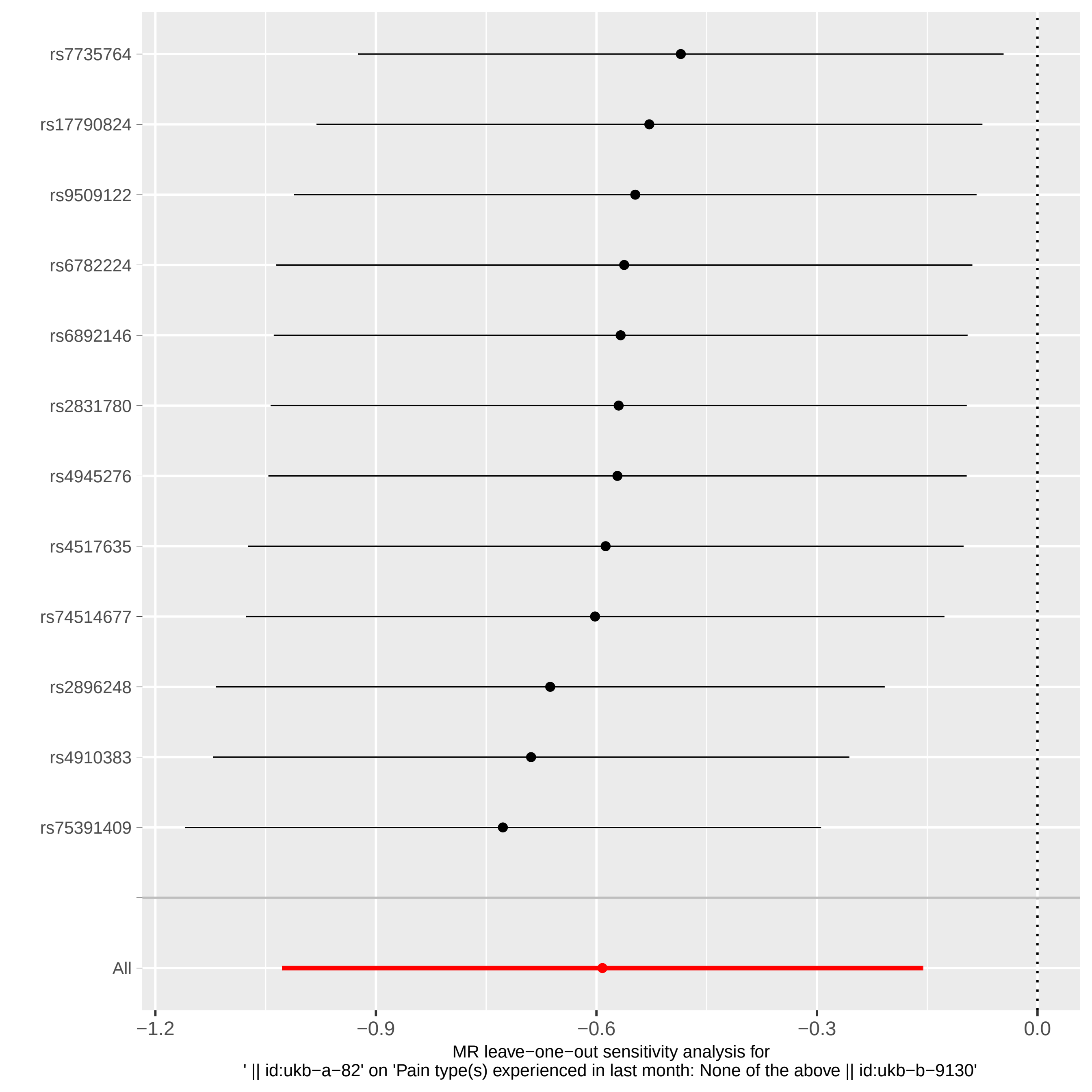

rs7735764
rs17790824
rs9509122
rs6782224
rs6892146
rs2831780
rs4945276
rs4517635
rs74514677
rs2896248
rs4910383
rs75391409
All
−1.2
−0.9
−0.6
−0.3
0.0
MR leave−one−out sensitivity analysis for
' || id:ukb−a−82' on 'Pain type(s) experienced in last month: None of the above || id:ukb−b−9130'

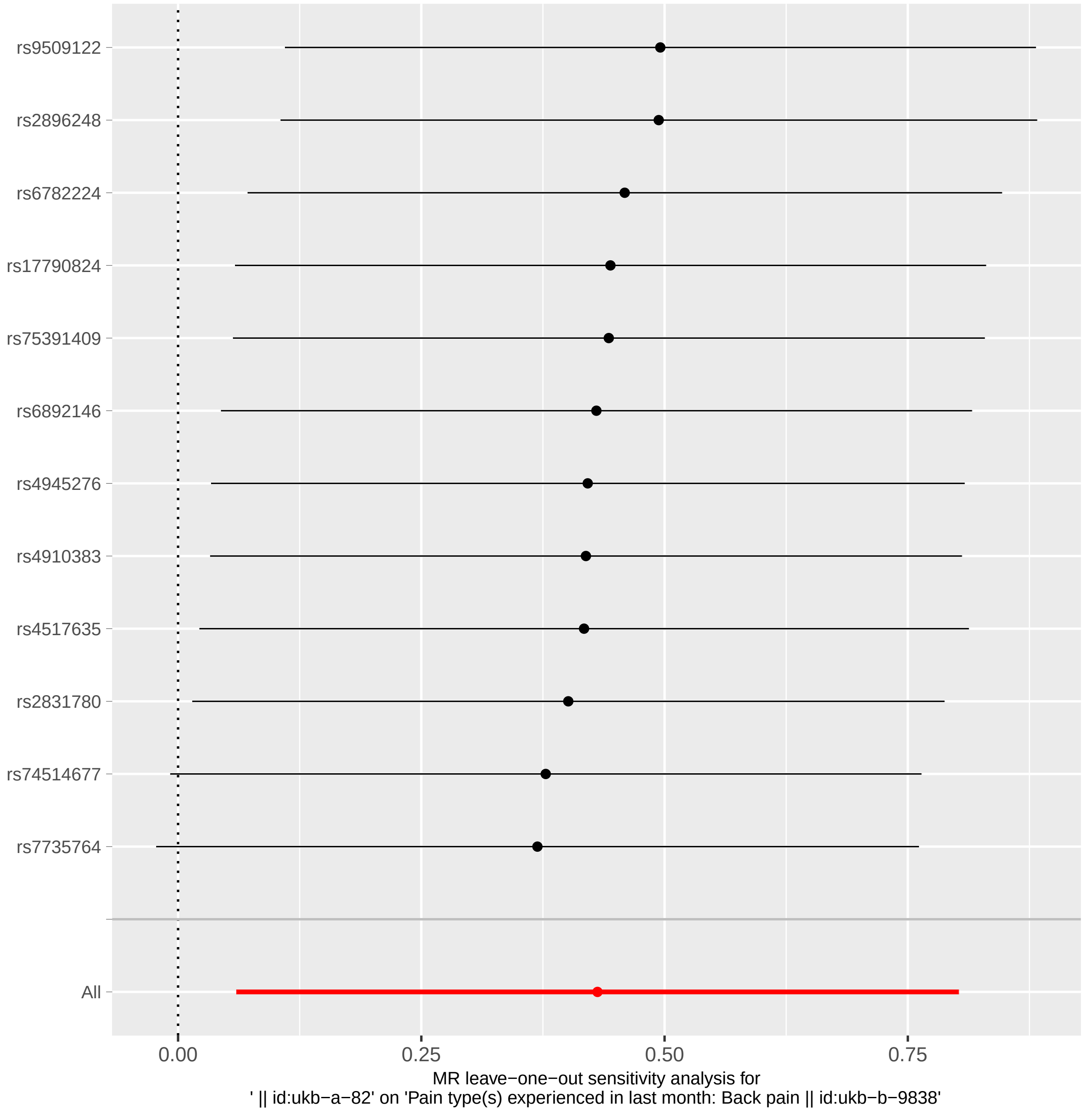
rs9509122
rs2896248
rs6782224
rs17790824
rs75391409
rs6892146
rs4945276
rs4910383
rs4517635
rs2831780
rs74514677
rs7735764
All
0.00
0.25
0.50
0.75
MR leave−one−out sensitivity analysis for
' || id:ukb−a−82' on 'Pain type(s) experienced in last month: Back pain || id:ukb−b−9838'

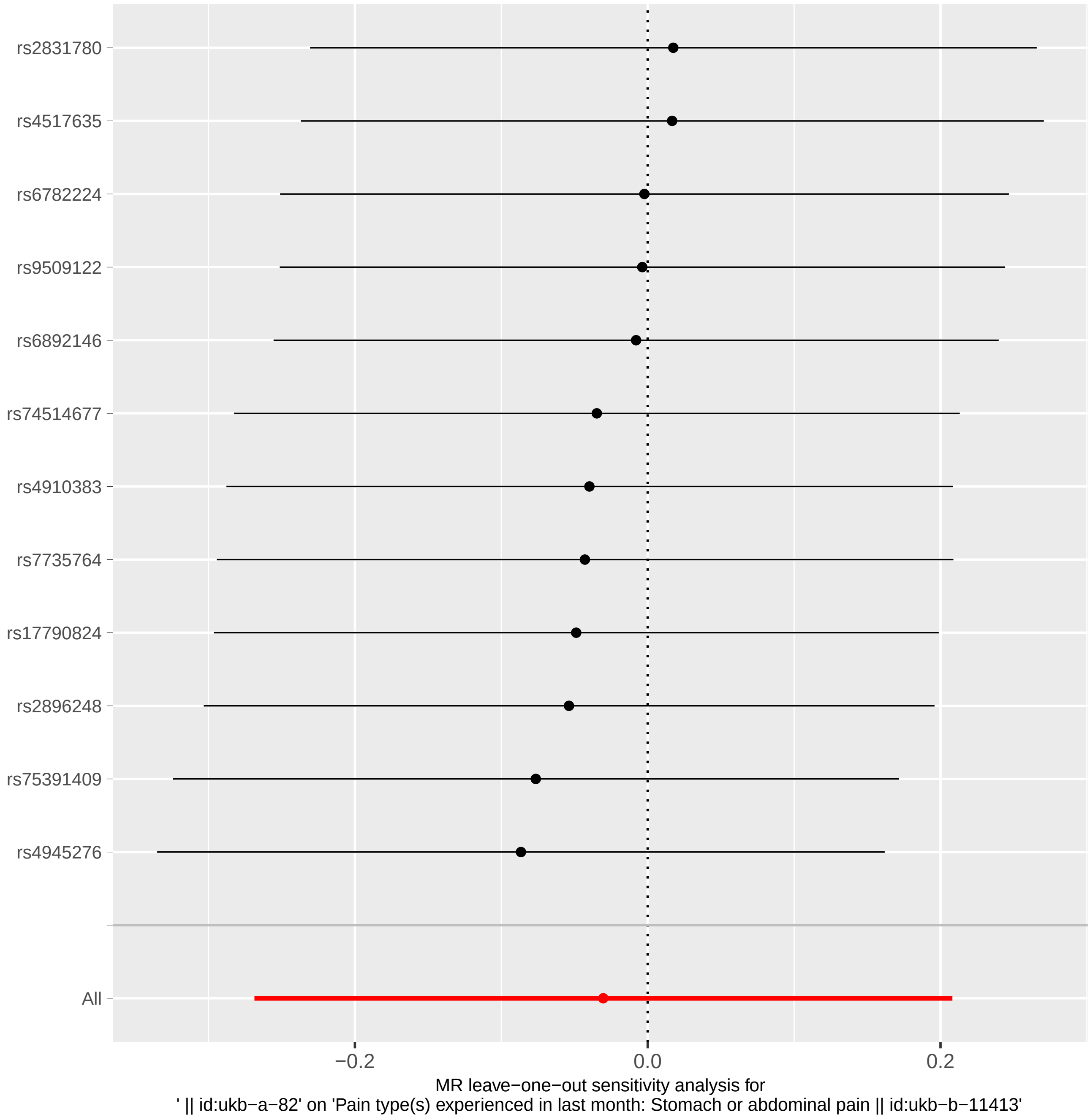

rs2831780
rs4517635
rs6782224
rs9509122
rs6892146
rs74514677
rs4910383
rs7735764
rs17790824
rs2896248
rs75391409
rs4945276
All
−0.2
0.0
0.2
MR leave−one−out sensitivity analysis for
' || id:ukb−a−82' on 'Pain type(s) experienced in last month: Stomach or abdominal pain || id:ukb−b−11413'

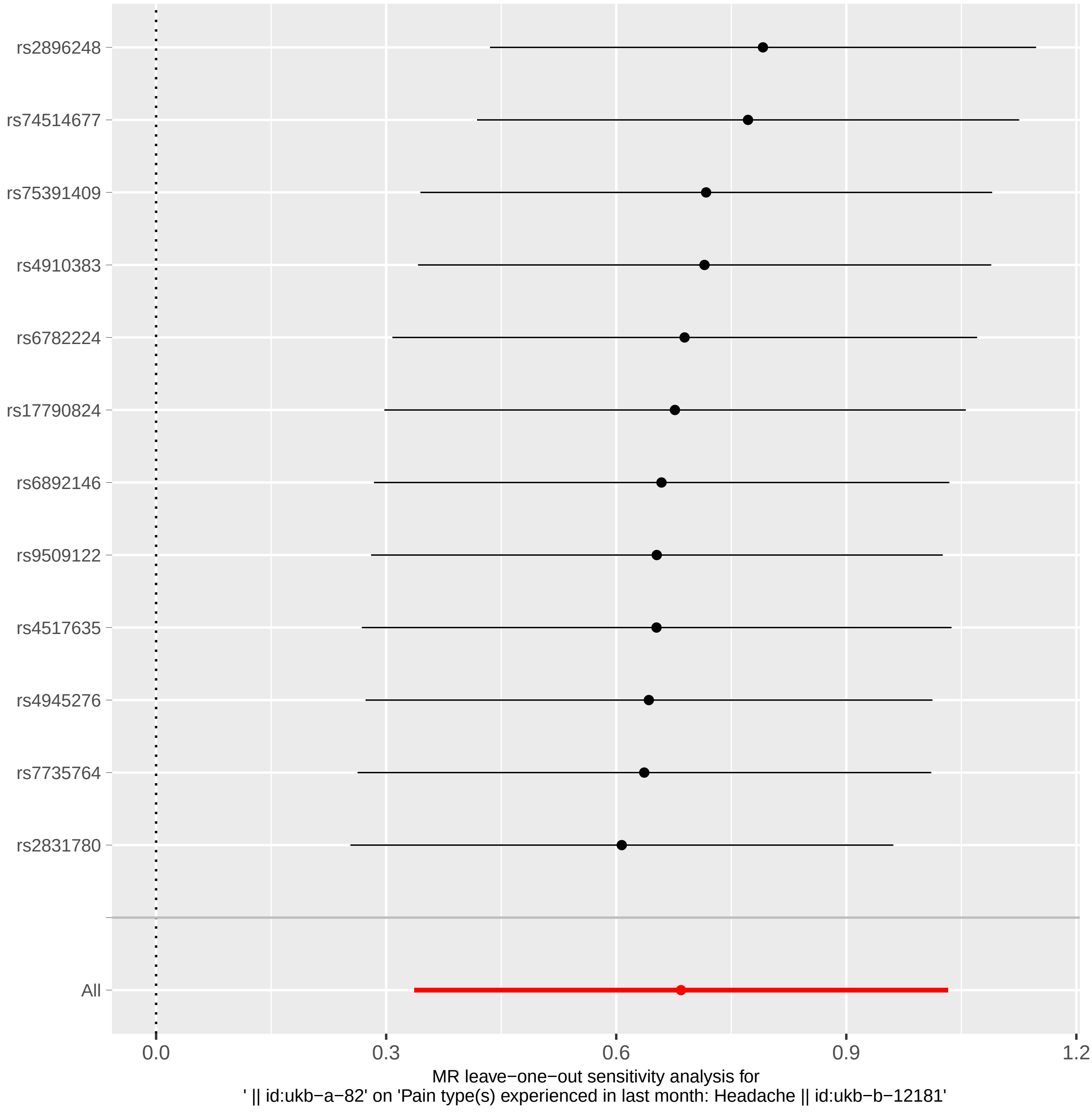

rs2896248
rs74514677
rs75391409
rs4910383
rs6782224
rs17790824
rs6892146
rs9509122
rs4517635
rs4945276
rs7735764
rs2831780
All
0.0
0.3
0.6
0.9
1.2
MR leave−one−out sensitivity analysis for
' || id:ukb−a−82' on 'Pain type(s) experienced in last month: Headache || id:ukb−b−12181'

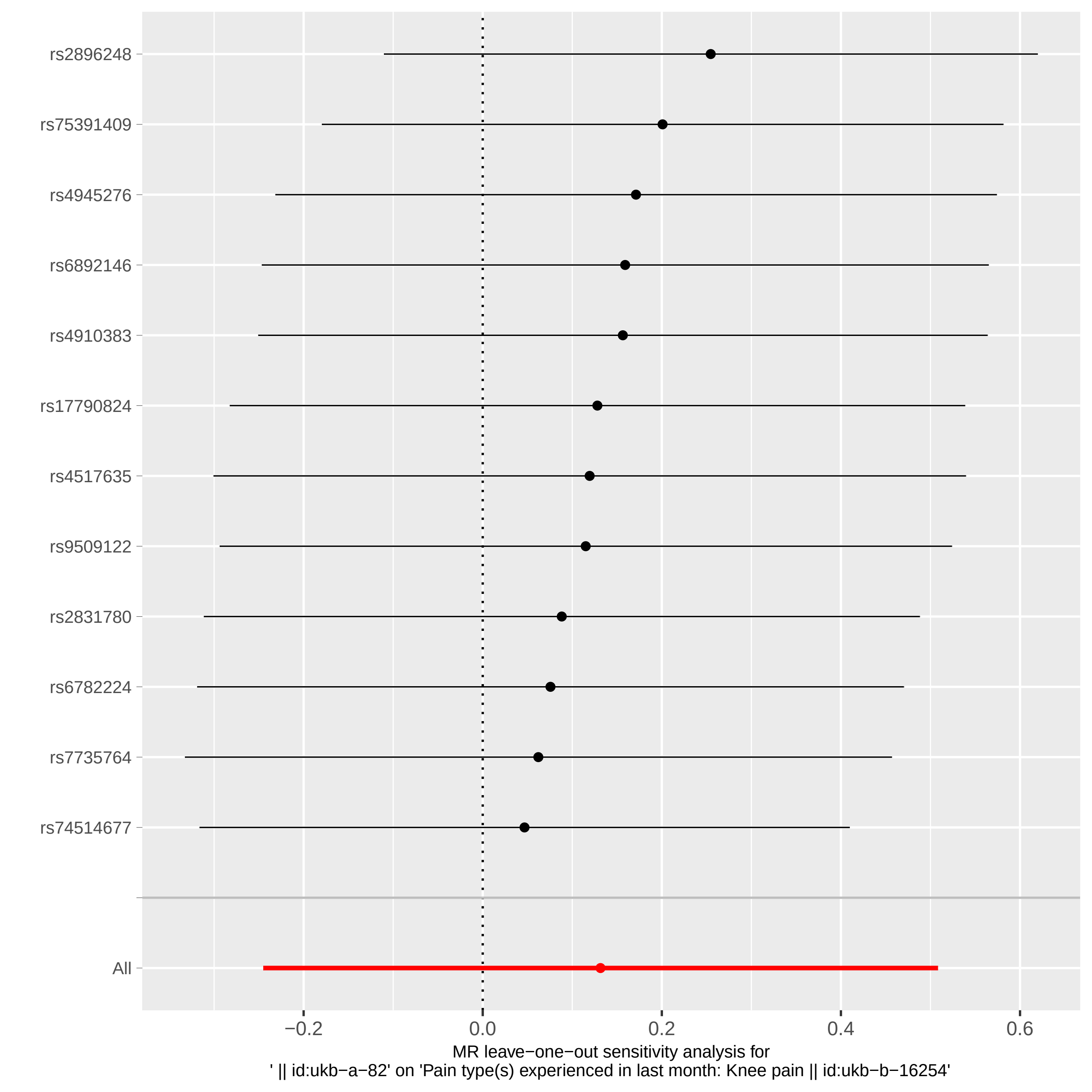

rs2896248
rs75391409
rs4945276
rs6892146
rs4910383
rs17790824
rs4517635
rs9509122
rs2831780
rs6782224
rs7735764
rs74514677
All
−0.2
0.0
0.2
0.4
0.6
MR leave−one−out sensitivity analysis for
' || id:ukb−a−82' on 'Pain type(s) experienced in last month: Knee pain || id:ukb−b−16254'

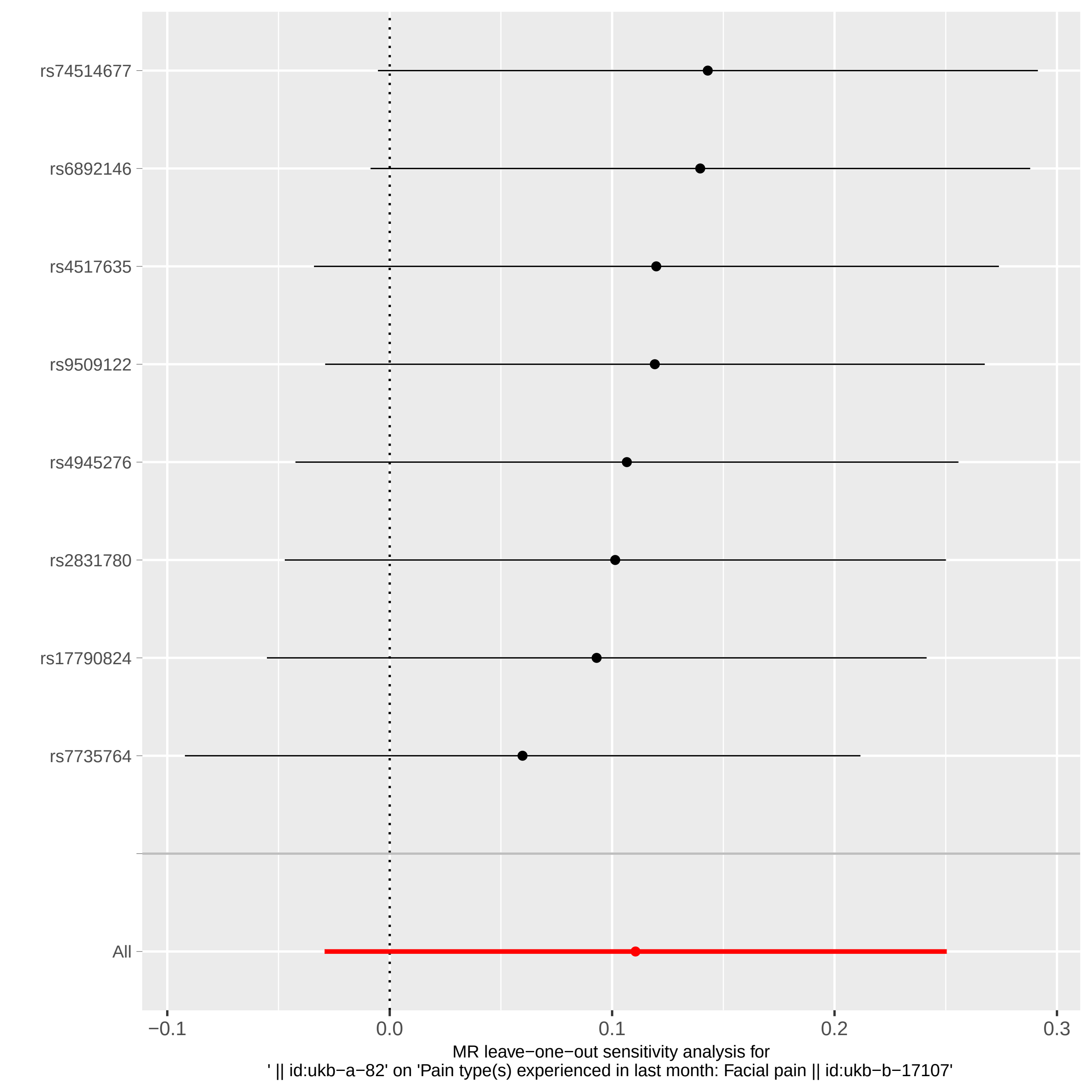
rs74514677
rs6892146
rs4517635
rs9509122
rs4945276
rs2831780
rs17790824
rs7735764
All
−0.1
0.0
0.1
0.2
0.3
MR leave−one−out sensitivity analysis for
' || id:ukb−a−82' on 'Pain type(s) experienced in last month: Facial pain || id:ukb−b−17107'

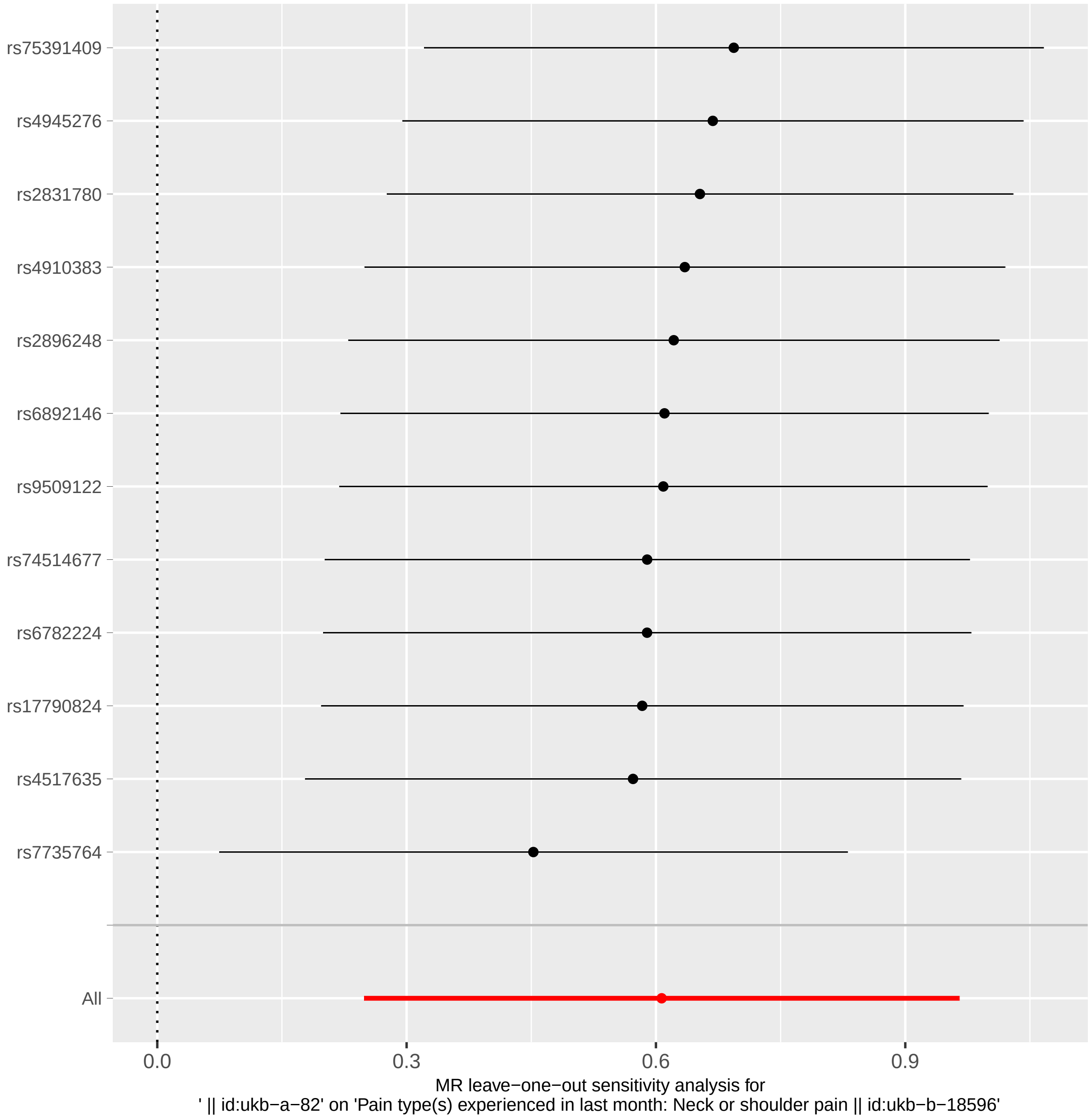
rs75391409
rs4945276
rs2831780
rs4910383
rs2896248
rs6892146
rs9509122
rs74514677
rs6782224
rs17790824
rs4517635
rs7735764
All
0.0
0.3
0.6
0.9
MR leave−one−out sensitivity analysis for
' || id:ukb−a−82' on 'Pain type(s) experienced in last month: Neck or shoulder pain || id:ukb−b−18596'

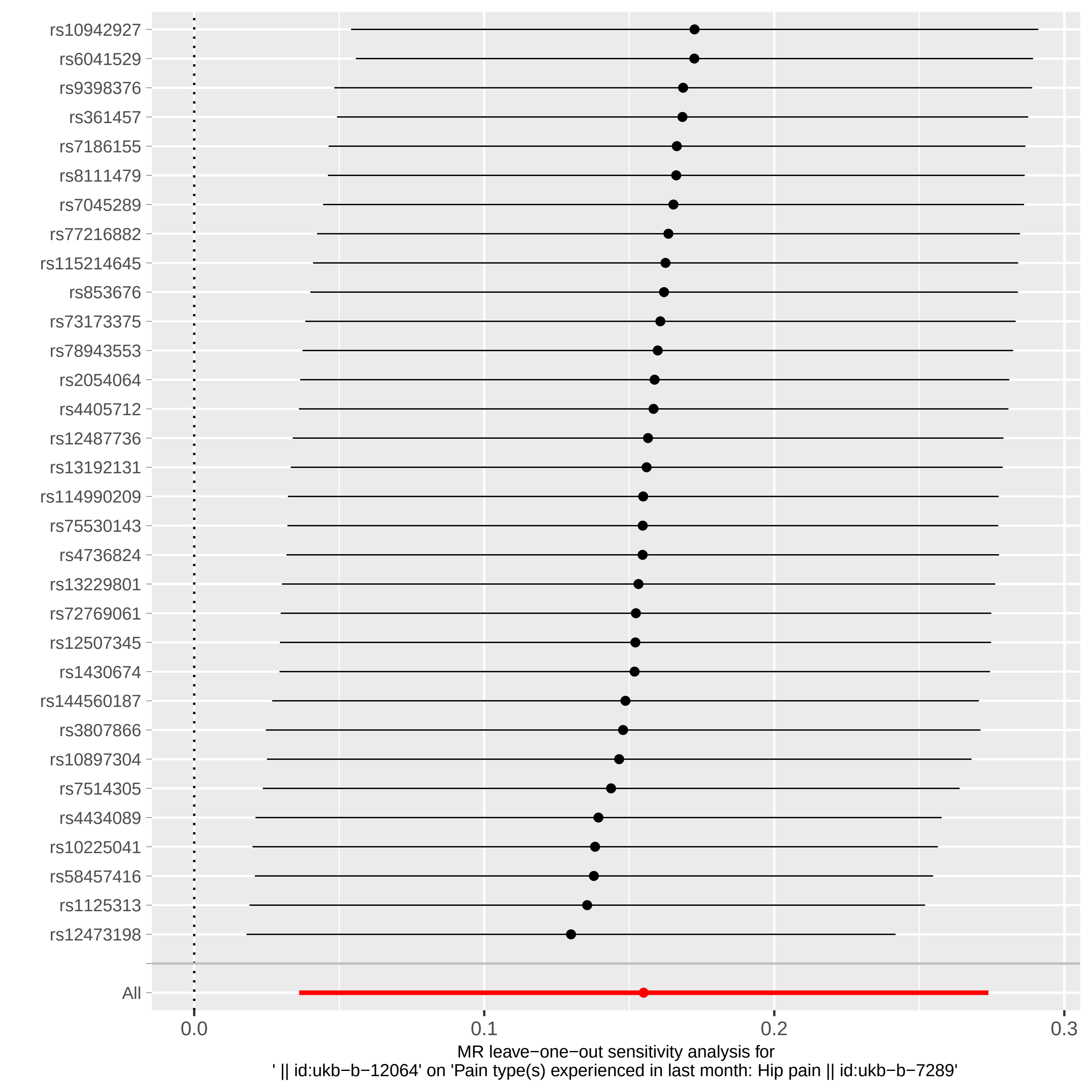
rs10942927
rs6041529
rs9398376
rs361457
rs7186155
rs8111479
rs7045289
rs77216882
rs115214645
rs853676
rs73173375
rs78943553
rs2054064
rs4405712
rs12487736
rs13192131
rs114990209
rs75530143
rs4736824
rs13229801
rs72769061
rs12507345
rs1430674
rs144560187
rs3807866
rs10897304
rs7514305
rs4434089
rs10225041
rs58457416
rs1125313
rs12473198
All
0.0
0.1
0.2
0.3
MR leave−one−out sensitivity analysis for
' || id:ukb−b−12064' on 'Pain type(s) experienced in last month: Hip pain || id:ukb−b−7289'

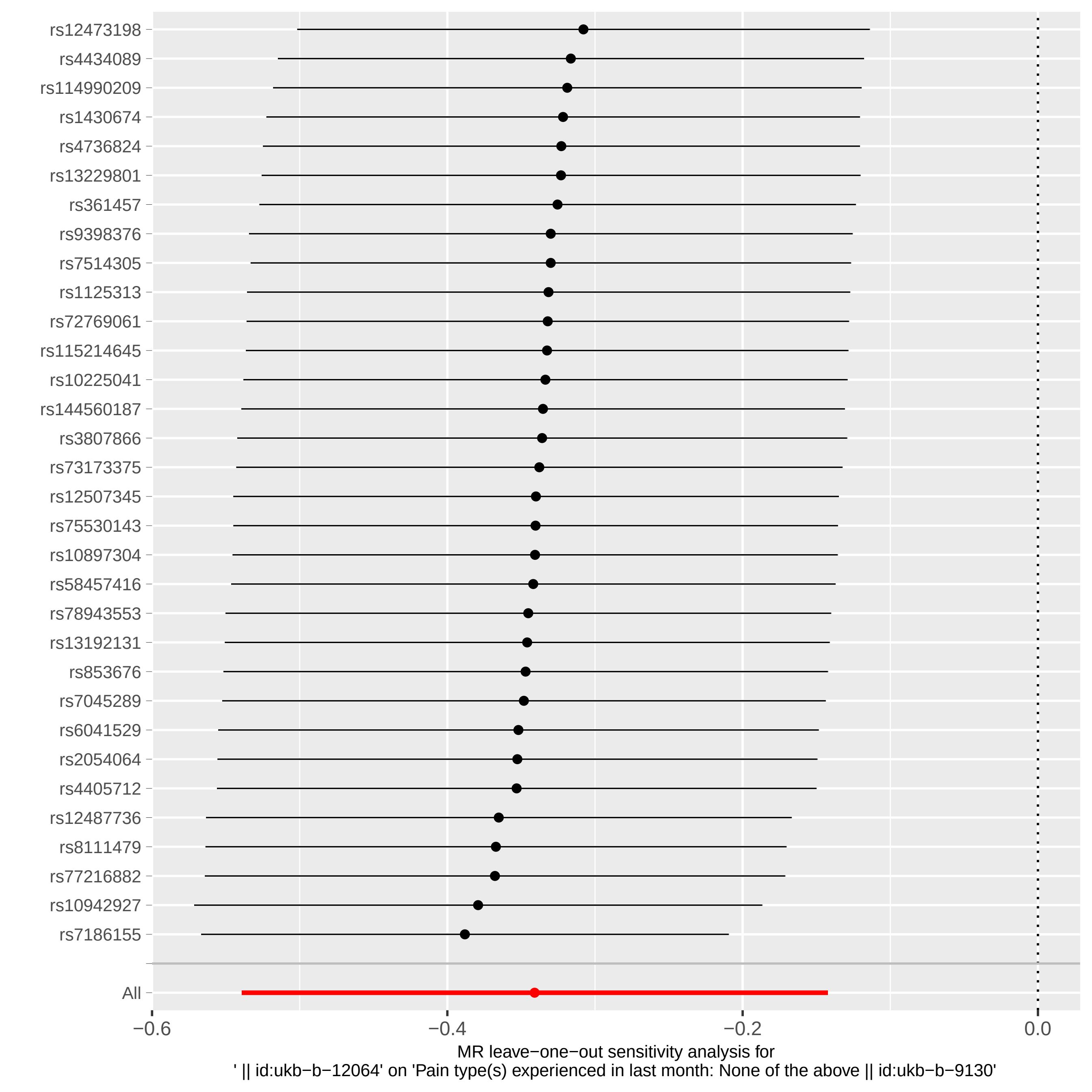
rs12473198
rs4434089
rs114990209
rs1430674
rs4736824
rs13229801
rs361457
rs9398376
rs7514305
rs1125313
rs72769061
rs115214645
rs10225041
rs144560187
rs3807866
rs73173375
rs12507345
rs75530143
rs10897304
rs58457416
rs78943553
rs13192131
rs853676
rs7045289
rs6041529
rs2054064
rs4405712
rs12487736
rs8111479
rs77216882
rs10942927
rs7186155
All
−0.6
−0.4
−0.2
0.0
MR leave−one−out sensitivity analysis for
' || id:ukb−b−12064' on 'Pain type(s) experienced in last month: None of the above || id:ukb−b−9130'

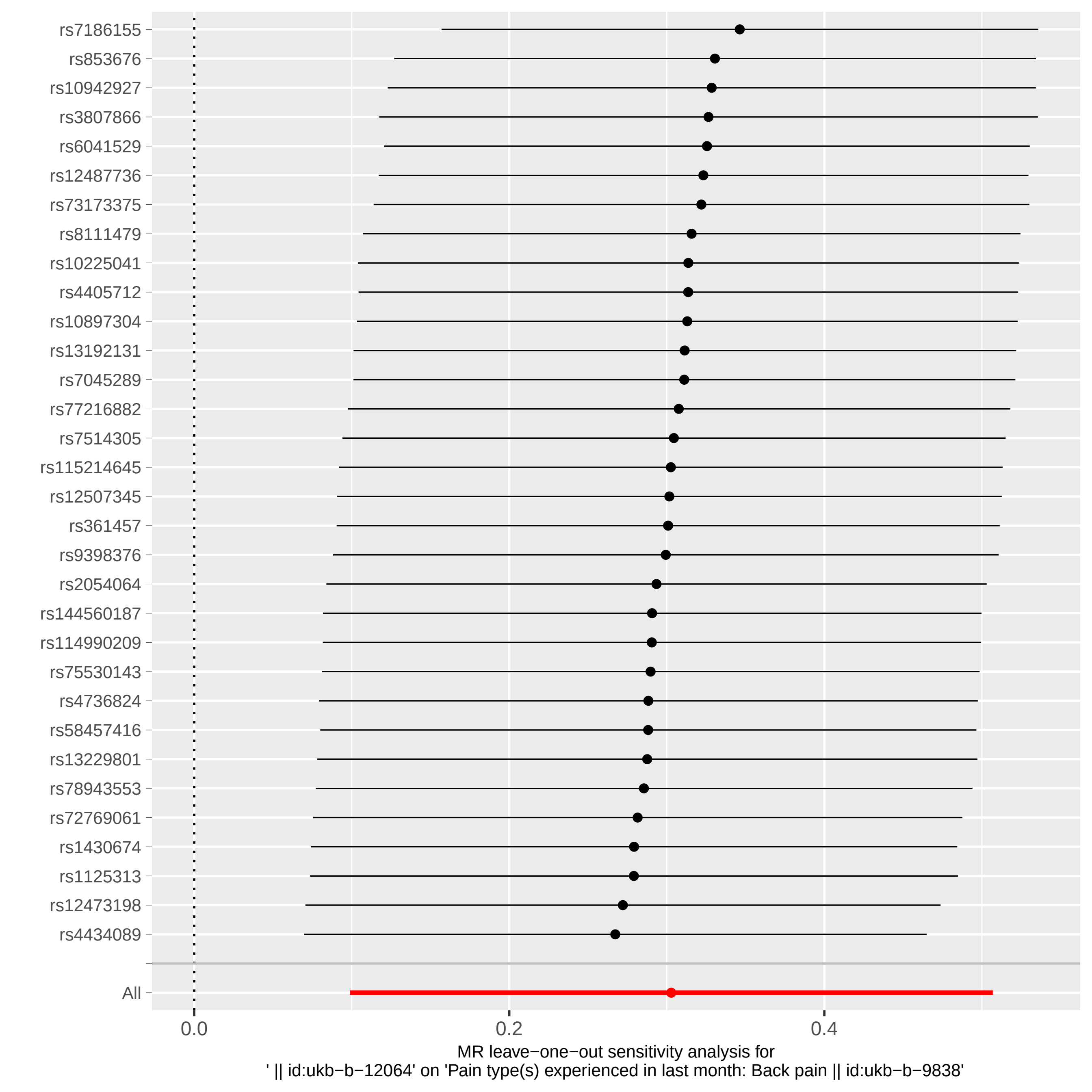

rs7186155
rs853676
rs10942927
rs3807866
rs6041529
rs12487736
rs73173375
rs8111479
rs10225041
rs4405712
rs10897304
rs13192131
rs7045289
rs77216882
rs7514305
rs115214645
rs12507345
rs361457
rs9398376
rs2054064
rs144560187
rs114990209
rs75530143
rs4736824
rs58457416
rs13229801
rs78943553
rs72769061
rs1430674
rs1125313
rs12473198
rs4434089
All
0.0
0.2
0.4
MR leave−one−out sensitivity analysis for
' || id:ukb−b−12064' on 'Pain type(s) experienced in last month: Back pain || id:ukb−b−9838'

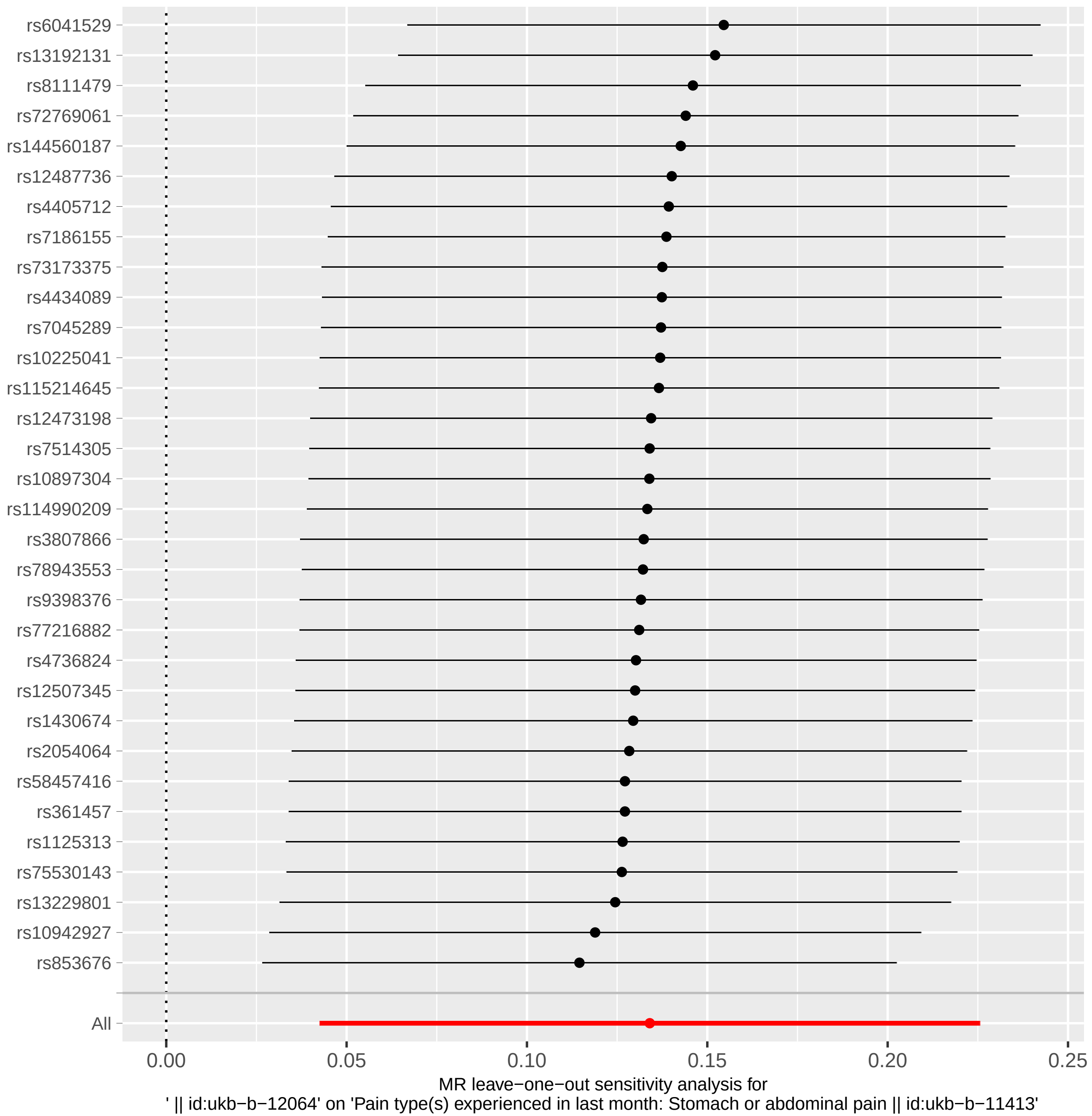
rs6041529
rs13192131
rs8111479
rs72769061
rs144560187
rs12487736
rs4405712
rs7186155
rs73173375
rs4434089
rs7045289
rs10225041
rs115214645
rs12473198
rs7514305
rs10897304
rs114990209
rs3807866
rs78943553
rs9398376
rs77216882
rs4736824
rs12507345
rs1430674
rs2054064
rs58457416
rs361457
rs1125313
rs75530143
rs13229801
rs10942927
rs853676
All
0.00
0.05
0.10
0.15
0.20
0.25
MR leave−one−out sensitivity analysis for
' || id:ukb−b−12064' on 'Pain type(s) experienced in last month: Stomach or abdominal pain || id:ukb−b−11413'

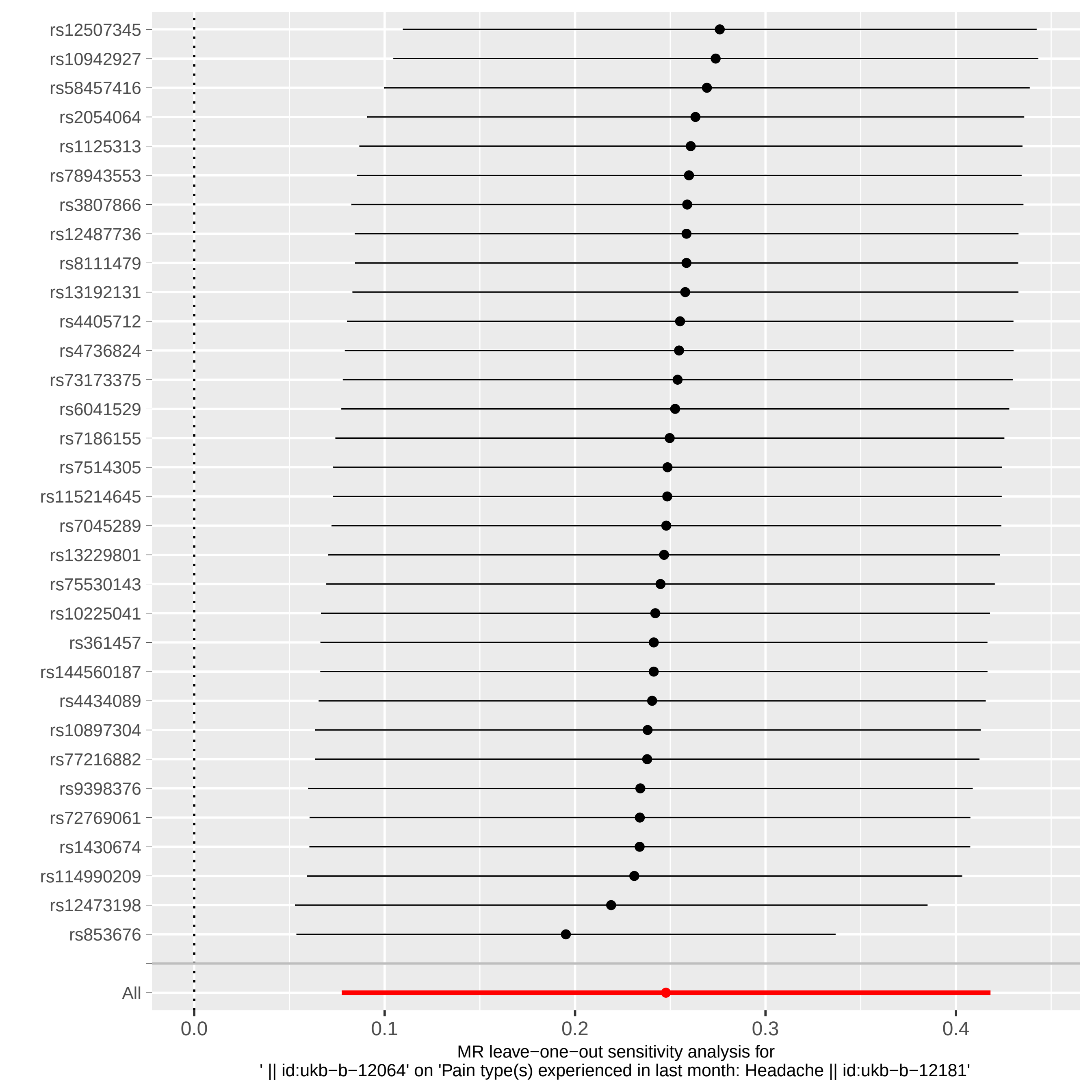
rs12507345
rs10942927
rs58457416
rs2054064
rs1125313
rs78943553
rs3807866
rs12487736
rs8111479
rs13192131
rs4405712
rs4736824
rs73173375
rs6041529
rs7186155
rs7514305
rs115214645
rs7045289
rs13229801
rs75530143
rs10225041
rs361457
rs144560187
rs4434089
rs10897304
rs77216882
rs9398376
rs72769061
rs1430674
rs114990209
rs12473198
rs853676
All
0.0
0.1
0.2
0.3
0.4
MR leave−one−out sensitivity analysis for
' || id:ukb−b−12064' on 'Pain type(s) experienced in last month: Headache || id:ukb−b−12181'

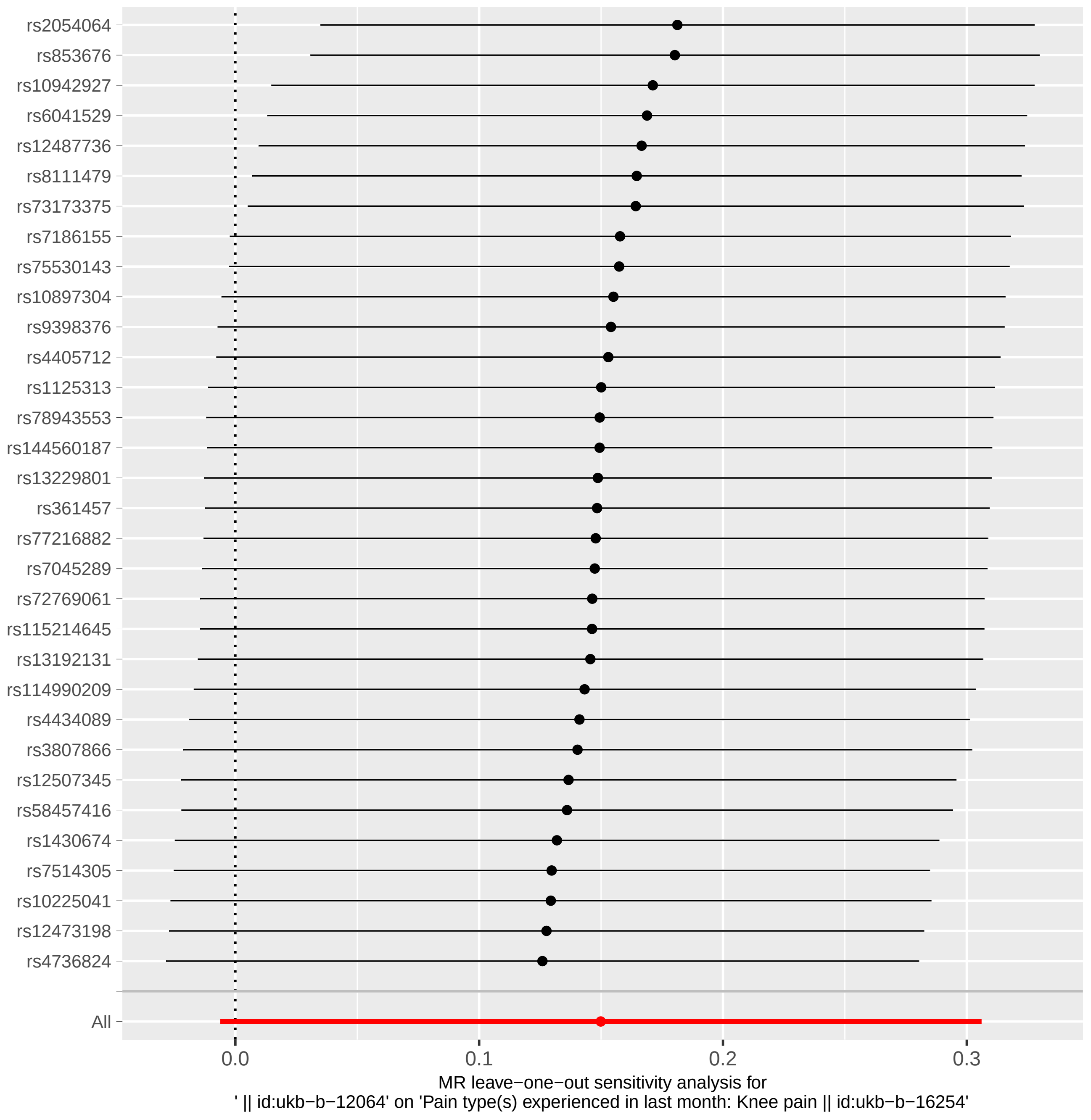

rs2054064
rs853676
rs10942927
rs6041529
rs12487736
rs8111479
rs73173375
rs7186155
rs75530143
rs10897304
rs9398376
rs4405712
rs1125313
rs78943553
rs144560187
rs13229801
rs361457
rs77216882
rs7045289
rs72769061
rs115214645
rs13192131
rs114990209
rs4434089
rs3807866
rs12507345
rs58457416
rs1430674
rs7514305
rs10225041
rs12473198
rs4736824
All
0.0
0.1
0.2
0.3
MR leave−one−out sensitivity analysis for
' || id:ukb−b−12064' on 'Pain type(s) experienced in last month: Knee pain || id:ukb−b−16254'

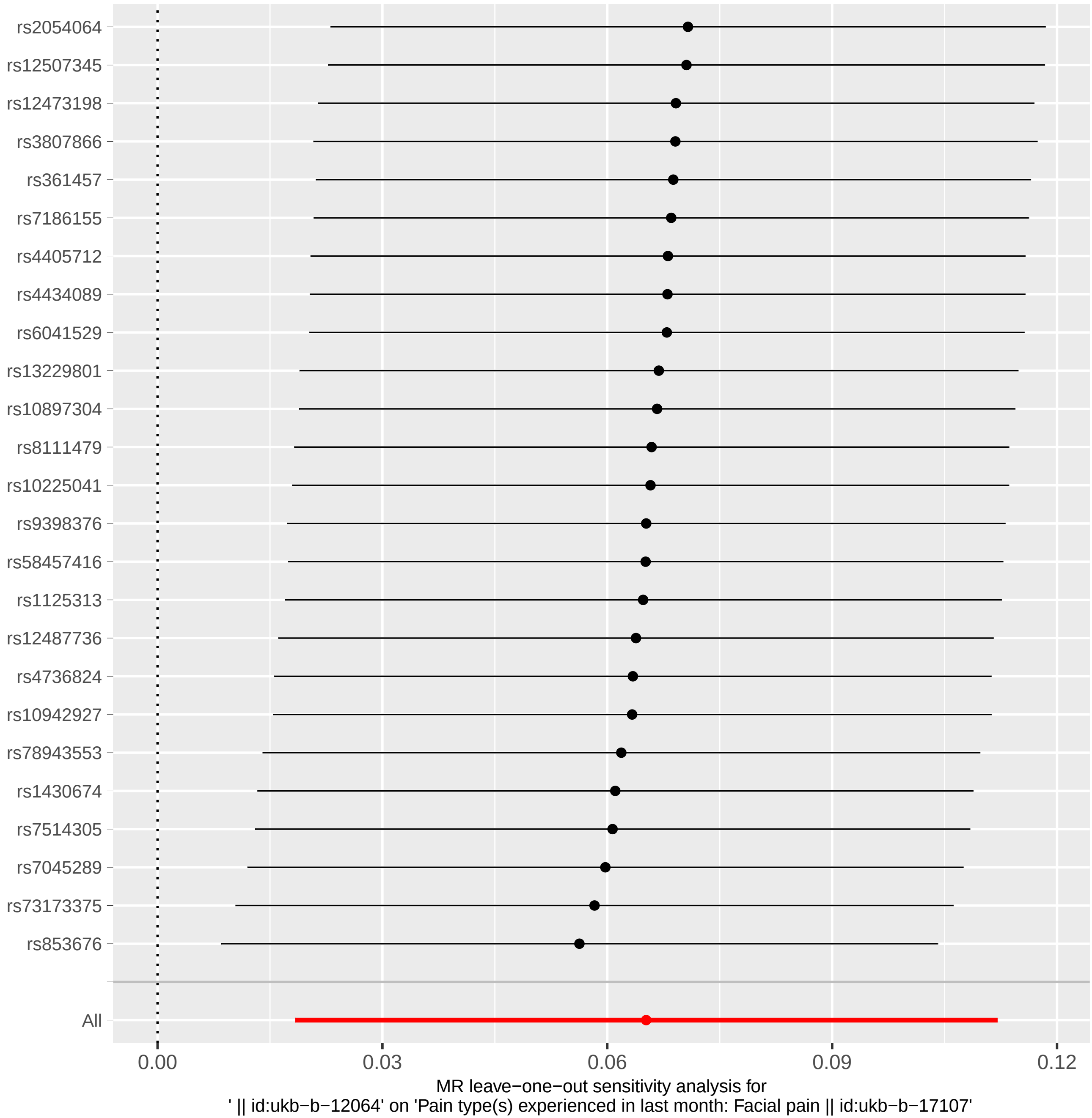
rs2054064
rs12507345
rs12473198
rs3807866
rs361457
rs7186155
rs4405712
rs4434089
rs6041529
rs13229801
rs10897304
rs8111479
rs10225041
rs9398376
rs58457416
rs1125313
rs12487736
rs4736824
rs10942927
rs78943553
rs1430674
rs7514305
rs7045289
rs73173375
rs853676
All
0.00
0.03
0.06
0.09
0.12
MR leave−one−out sensitivity analysis for
' || id:ukb−b−12064' on 'Pain type(s) experienced in last month: Facial pain || id:ukb−b−17107'

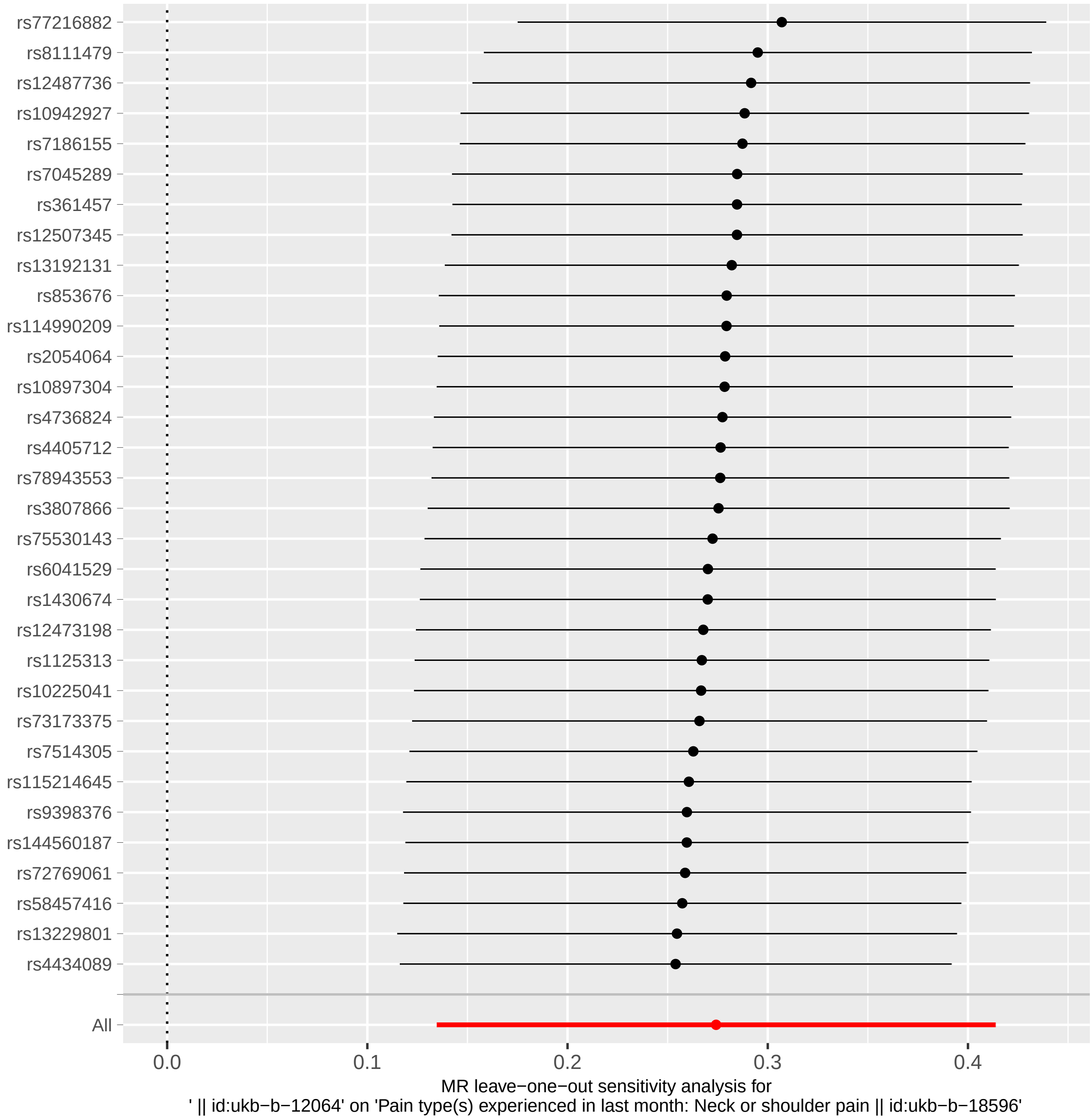
rs77216882
rs8111479
rs12487736
rs10942927
rs7186155
rs7045289
rs361457
rs12507345
rs13192131
rs853676
rs114990209
rs2054064
rs10897304
rs4736824
rs4405712
rs78943553
rs3807866
rs75530143
rs6041529
rs1430674
rs12473198
rs1125313
rs10225041
rs73173375
rs7514305
rs115214645
rs9398376
rs144560187
rs72769061
rs58457416
rs13229801
rs4434089
All
0.0
0.1
0.2
0.3
0.4
MR leave−one−out sensitivity analysis for
' || id:ukb−b−12064' on 'Pain type(s) experienced in last month: Neck or shoulder pain || id:ukb−b−18596'

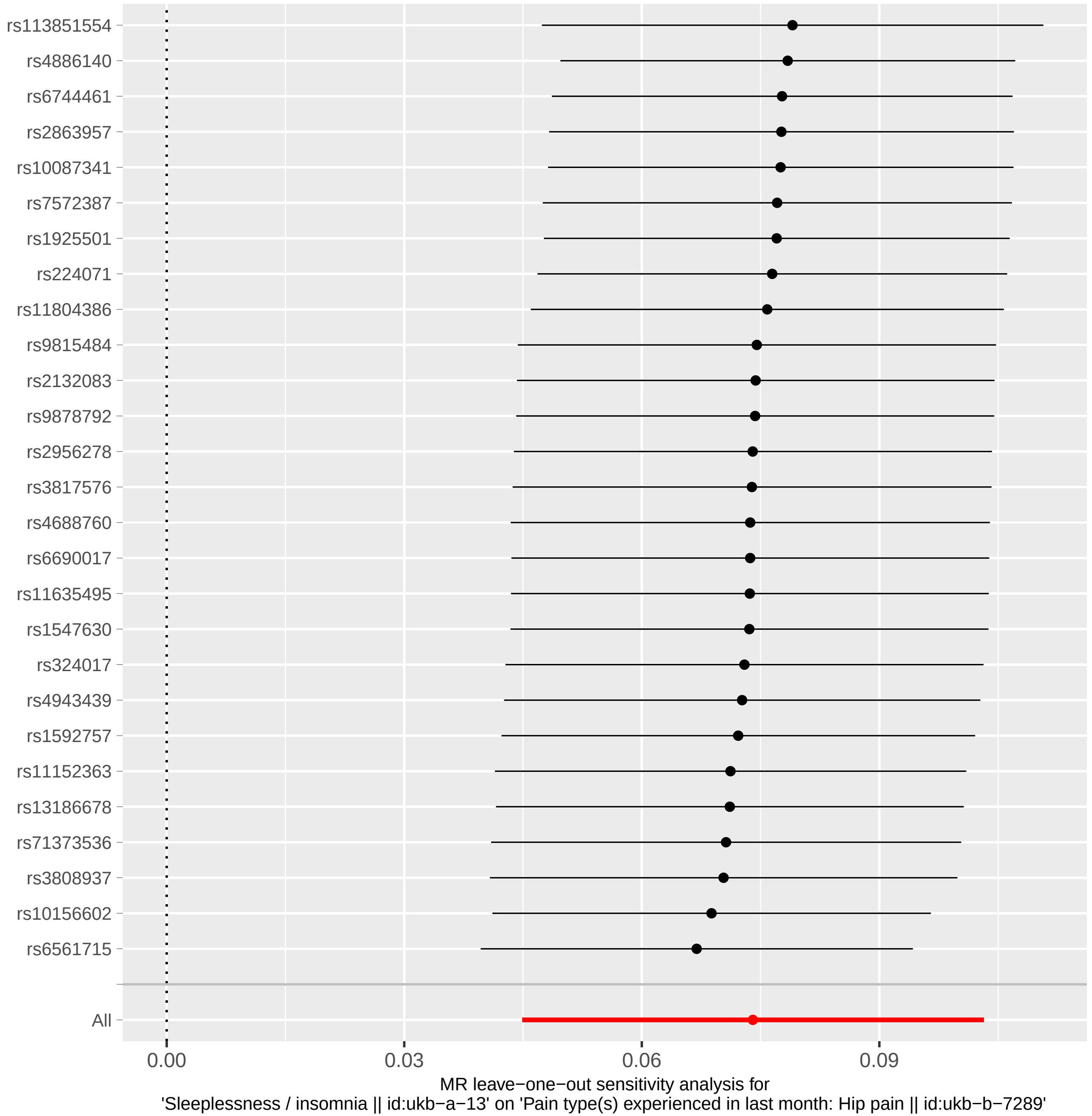

rs113851554
rs4886140
rs6744461
rs2863957
rs10087341
rs7572387
rs1925501
rs224071
rs11804386
rs9815484
rs2132083
rs9878792
rs2956278
rs3817576
rs4688760
rs6690017
rs11635495
rs1547630
rs324017
rs4943439
rs1592757
rs11152363
rs13186678
rs71373536
rs3808937
rs10156602
rs6561715
All
0.00
0.03
0.06
0.09
MR leave−one−out sensitivity analysis for
'Sleeplessness / insomnia || id:ukb−a−13' on 'Pain type(s) experienced in last month: Hip pain || id:ukb−b−7289'

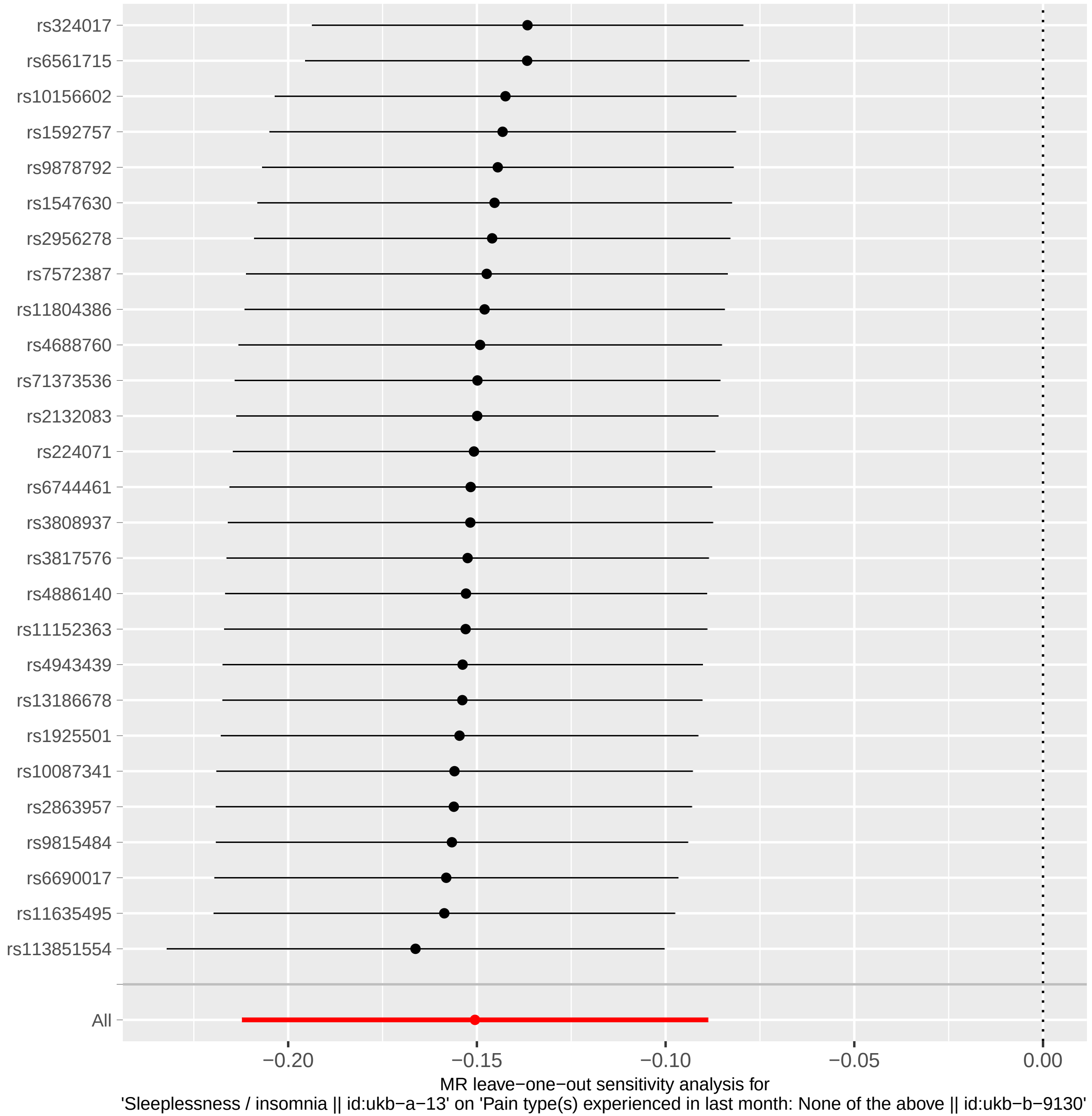

rs324017
rs6561715
rs10156602
rs1592757
rs9878792
rs1547630
rs2956278
rs7572387
rs11804386
rs4688760
rs71373536
rs2132083
rs224071
rs6744461
rs3808937
rs3817576
rs4886140
rs11152363
rs4943439
rs13186678
rs1925501
rs10087341
rs2863957
rs9815484
rs6690017
rs11635495
rs113851554
All
−0.20
−0.15
−0.10
−0.05
0.00
MR leave−one−out sensitivity analysis for
'Sleeplessness / insomnia || id:ukb−a−13' on 'Pain type(s) experienced in last month: None of the above || id:ukb−b−9130'

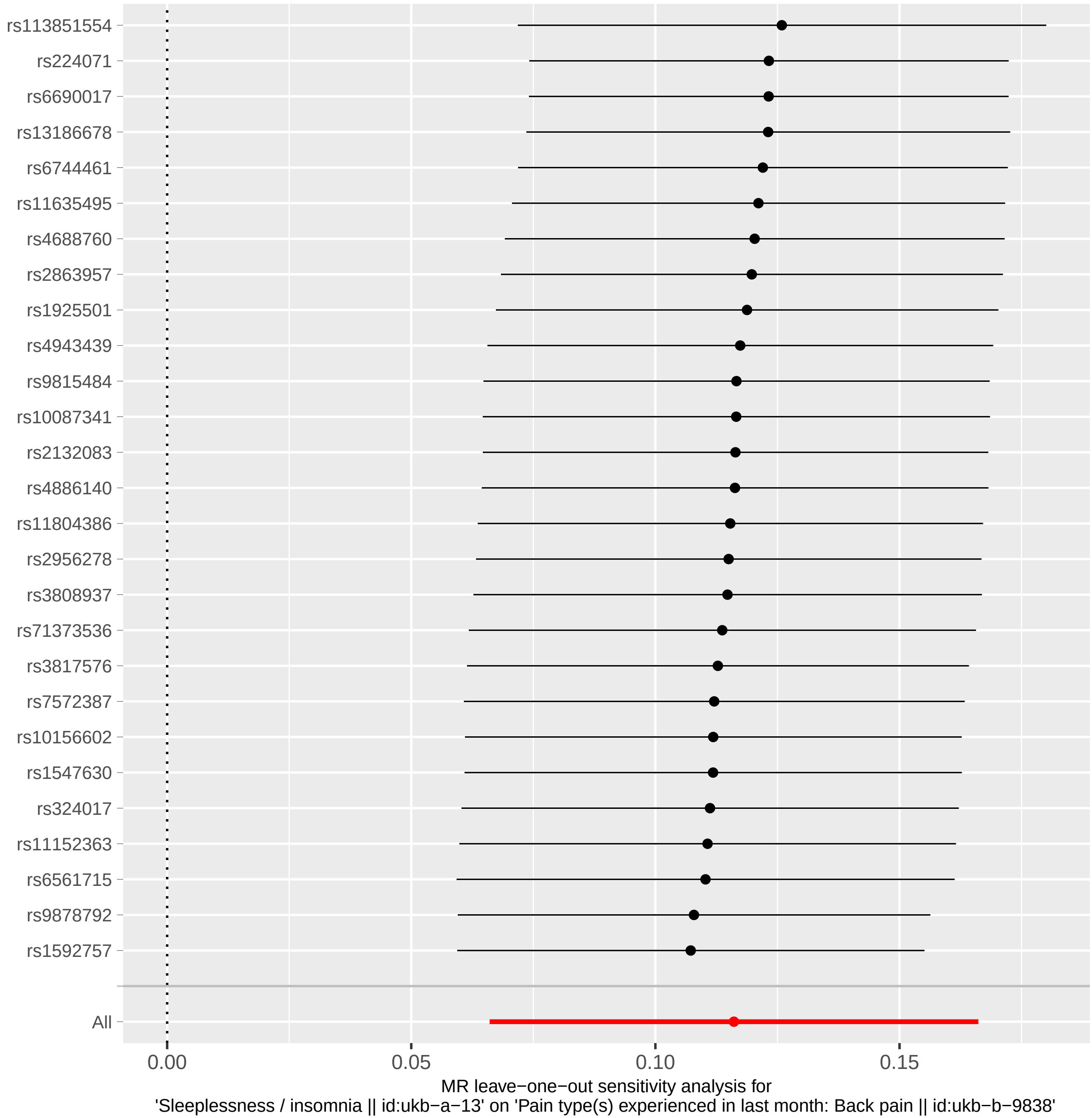
rs113851554
rs224071
rs6690017
rs13186678
rs6744461
rs11635495
rs4688760
rs2863957
rs1925501
rs4943439
rs9815484
rs10087341
rs2132083
rs4886140
rs11804386
rs2956278
rs3808937
rs71373536
rs3817576
rs7572387
rs10156602
rs1547630
rs324017
rs11152363
rs6561715
rs9878792
rs1592757
All
0.00
0.05
0.10
0.15
MR leave−one−out sensitivity analysis for
'Sleeplessness / insomnia || id:ukb−a−13' on 'Pain type(s) experienced in last month: Back pain || id:ukb−b−9838'

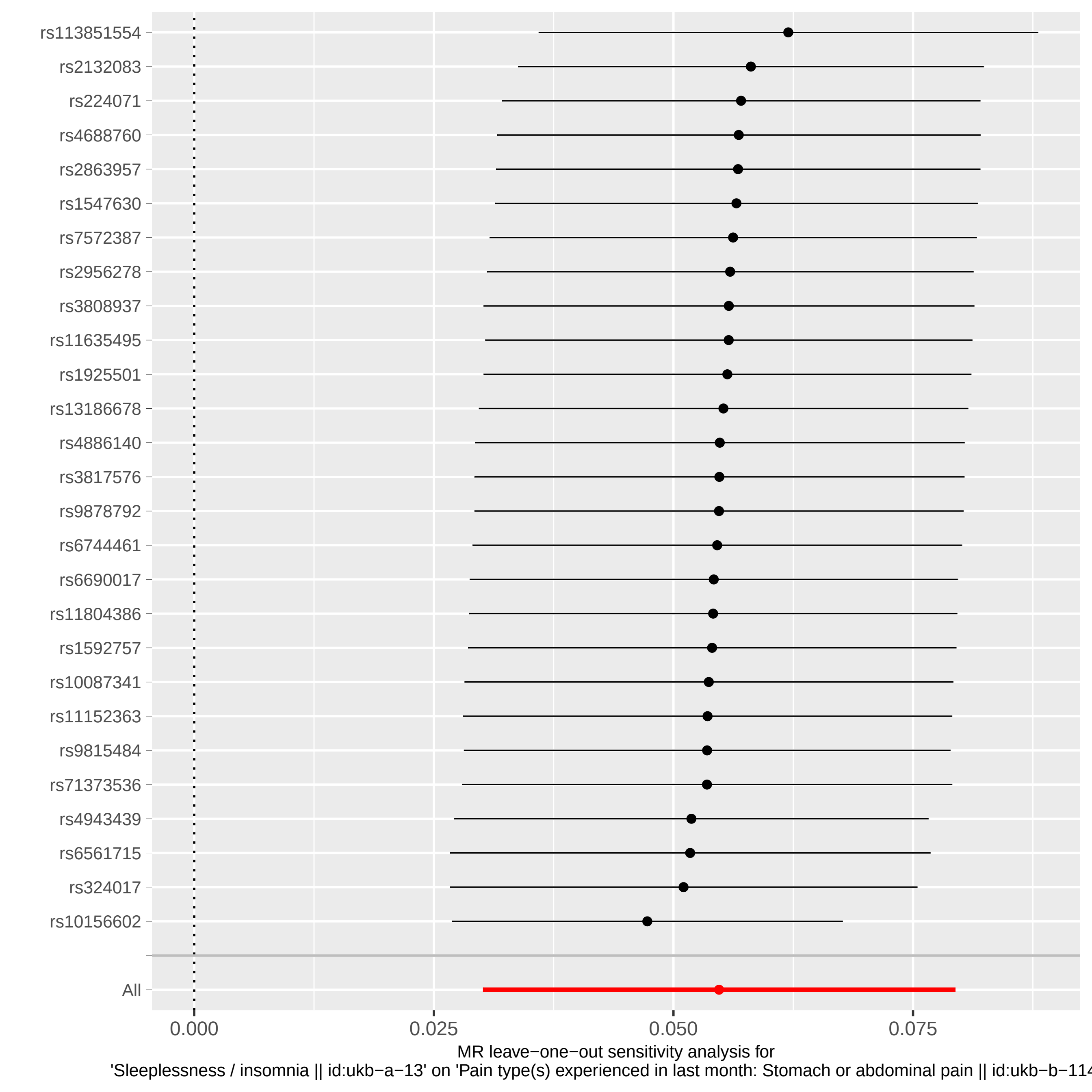
rs113851554
rs2132083
rs224071
rs4688760
rs2863957
rs1547630
rs7572387
rs2956278
rs3808937
rs11635495
rs1925501
rs13186678
rs4886140
rs3817576
rs9878792
rs6744461
rs6690017
rs11804386
rs1592757
rs10087341
rs11152363
rs9815484
rs71373536
rs4943439
rs6561715
rs324017
rs10156602
All
0.000
0.025
0.050
0.075
MR leave−one−out sensitivity analysis for
'Sleeplessness / insomnia || id:ukb−a−13' on 'Pain type(s) experienced in last month: Stomach or abdominal pain || id:ukb−b−114

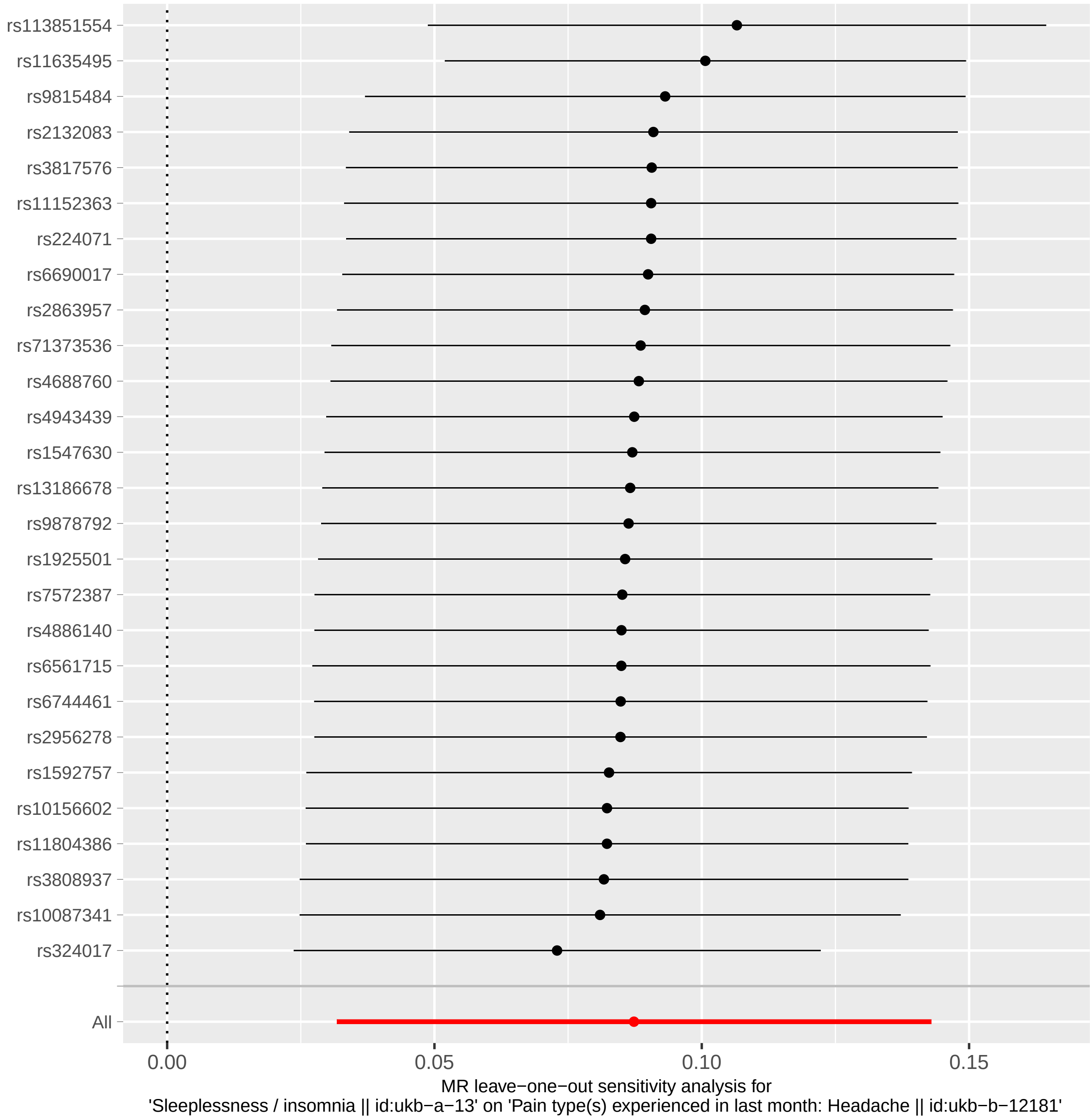

rs113851554
rs11635495
rs9815484
rs2132083
rs3817576
rs11152363
rs224071
rs6690017
rs2863957
rs71373536
rs4688760
rs4943439
rs1547630
rs13186678
rs9878792
rs1925501
rs7572387
rs4886140
rs6561715
rs6744461
rs2956278
rs1592757
rs10156602
rs11804386
rs3808937
rs10087341
rs324017
All
0.00
0.05
0.10
0.15
MR leave−one−out sensitivity analysis for
'Sleeplessness / insomnia || id:ukb−a−13' on 'Pain type(s) experienced in last month: Headache || id:ukb−b−12181'
