# Supplementary material for: Exploring the bidirectional relationship between pain and mental disorders: a comprehensive Mendelian randomization study
Source: J Headache Pain. 2023 Jul 7;24(1):82. doi: 10.1186/s10194-023-01612-2 (PMC10326936; doi:10.1186/s10194-023-01612-2)
Supplement: Supplementary file 7 — Additional file 7: Supplementary file 7. Estimated biases due to sample overlap. [file 10194_2023_1612_MOESM7_ESM.pdf]

Supplementary file 7

Estimated biases due to sample overlap

Bias and type 1 error rate for Mendelian randomization analysis of headache on sleeplessness/insomnia

Using specified value of concentration parameter			Using conservative value of concentration parameter		
Overlap proportion	Bias	Type 1 error rate	Overlap proportion	Bias	Type 1 error rate
0.0	0.000	0.05	0.0	0.000	0.05
0.1	0.000	0.05	0.1	0.000	0.05
0.2	0.000	0.05	0.2	0.000	0.05
0.3	0.001	0.05	0.3	0.001	0.05
0.4	0.001	0.05	0.4	0.001	0.05
0.5	0.001	0.05	0.5	0.001	0.05
0.6	0.001	0.05	0.6	0.001	0.05
0.7	0.001	0.05	0.7	0.001	0.05
0.8	0.002	0.05	0.8	0.002	0.05
0.9	0.002	0.05	0.9	0.002	0.05
1.0	0.002	0.05	1.0	0.002	0.05

Concentration parameter (expected value of F statistic) = 209.44
Conservative value of concentration parameter (lower limit of one-sided 95% confidence interval) = 209.43

Bias and type 1 error rate for Mendelian randomization analysis of facial pain on sleeplessness/insomnia

Using specified value of concentration parameter			Using conservative value of concentration parameter		
Overlap proportion	Bias	Type 1 error rate	Overlap proportion	Bias	Type 1 error rate
0.0	0.000	0.05	0.0	0.000	0.05
0.1	0.000	0.05	0.1	0.000	0.05
0.2	0.000	0.05	0.2	0.000	0.05
0.3	0.000	0.05	0.3	0.000	0.05
0.4	0.001	0.05	0.4	0.001	0.05
0.5	0.001	0.05	0.5	0.001	0.05
0.6	0.001	0.05	0.6	0.001	0.05
0.7	0.001	0.05	0.7	0.001	0.05
0.8	0.001	0.05	0.8	0.001	0.05
0.9	0.001	0.05	0.9	0.001	0.05
1.0	0.001	0.05	1.0	0.001	0.05

Concentration parameter (expected value of F statistic) = 291.57
Conservative value of concentration parameter (lower limit of one-sided 95% confidence interval) = 291.56

Bias and type 1 error rate for Mendelian randomization analysis of neck/shoulder pain on sleeplessness/insomnia

Using specified value of concentration parameter			Using conservative value of concentration parameter		
Overlap proportion	Bias	Type 1 error rate	Overlap proportion	Bias	Type 1 error rate
0.0	0.000	0.05	0.0	0.000	0.05
0.1	0.000	0.05	0.1	0.000	0.05
0.2	0.000	0.05	0.2	0.000	0.05
0.3	0.000	0.05	0.3	0.000	0.05
0.4	0.000	0.05	0.4	0.000	0.05
0.5	0.000	0.05	0.5	0.000	0.05
0.6	0.000	0.05	0.6	0.000	0.05
0.7	0.000	0.05	0.7	0.000	0.05
0.8	0.000	0.05	0.8	0.000	0.05
0.9	0.000	0.05	0.9	0.000	0.05
1.0	0.001	0.05	1.0	0.001	0.05

Concentration parameter (expected value of F statistic) = 777.53
Conservative value of concentration parameter (lower limit of one-sided 95% confidence interval) = 777.52

Bias and type 1 error rate for Mendelian randomization analysis of back pain on sleeplessness/insomnia

```
Using specified value of concentration parameter       Using conservative value of concentration parameter
Overlap proportion      Bias    Type 1 error rate      Overlap proportion      Bias    Type 1 error rate
------------------------------------------------       ------------------------------------------------
0.0                     0.000   0.05                   0.0                     0.000   0.05
0.1                     0.000   0.05                   0.1                     0.000   0.05
0.2                     0.000   0.05                   0.2                     0.000   0.05
0.3                     0.001   0.05                   0.3                     0.001   0.05
0.4                     0.001   0.05                   0.4                     0.001   0.05
0.5                     0.001   0.05                   0.5                     0.001   0.05
0.6                     0.001   0.05                   0.6                     0.001   0.05
0.7                     0.001   0.05                   0.7                     0.001   0.05
0.8                     0.001   0.05                   0.8                     0.001   0.05
0.9                     0.002   0.05                   0.9                     0.002   0.05
1.0                     0.002   0.05                   1.0                     0.002   0.05


Concentration parameter (expected value of F statistic) = 233.25
Conservative value of concentration parameter (lower limit of one-sided 95% confidence interval) = 233.25
```

Bias and type 1 error rate for Mendelian randomization analysis of stomach/abdominal pain on sleeplessness/insomnia

```
Using specified value of concentration parameter       Using conservative value of concentration parameter
Overlap proportion      Bias    Type 1 error rate      Overlap proportion      Bias    Type 1 error rate
------------------------------------------------       ------------------------------------------------
0.0                     0.000   0.05                   0.0                     0.000   0.05
0.1                     0.000   0.05                   0.1                     0.000   0.05
0.2                     0.000   0.05                   0.2                     0.000   0.05
0.3                     0.000   0.05                   0.3                     0.000   0.05
0.4                     0.001   0.05                   0.4                     0.001   0.05
0.5                     0.001   0.05                   0.5                     0.001   0.05
0.6                     0.001   0.05                   0.6                     0.001   0.05
0.7                     0.001   0.05                   0.7                     0.001   0.05
0.8                     0.001   0.05                   0.8                     0.001   0.05
0.9                     0.001   0.05                   0.9                     0.001   0.05
1.0                     0.001   0.05                   1.0                     0.001   0.05


Concentration parameter (expected value of F statistic) = 314.17
Conservative value of concentration parameter (lower limit of one-sided 95% confidence interval) = 314.16
```

Bias and type 1 error rate for Mendelian randomization analysis of hip pain on sleeplessness/insomnia

```
Using specified value of concentration parameter       Using conservative value of concentration parameter
Overlap proportion      Bias    Type 1 error rate      Overlap proportion      Bias    Type 1 error rate
------------------------------------------------       ------------------------------------------------
0.0                     0.000   0.05                   0.0                     0.000   0.05
0.1                     0.000   0.05                   0.1                     0.000   0.05
0.2                     0.000   0.05                   0.2                     0.000   0.05
0.3                     0.000   0.05                   0.3                     0.000   0.05
0.4                     0.000   0.05                   0.4                     0.000   0.05
0.5                     0.000   0.05                   0.5                     0.000   0.05
0.6                     0.000   0.05                   0.6                     0.000   0.05
0.7                     0.000   0.05                   0.7                     0.000   0.05
0.8                     0.000   0.05                   0.8                     0.000   0.05
0.9                     0.000   0.05                   0.9                     0.000   0.05
1.0                     0.000   0.05                   1.0                     0.000   0.05


Concentration parameter (expected value of F statistic) = 2356.39
Conservative value of concentration parameter (lower limit of one-sided 95% confidence interval) = 2356.38
```

Bias and type 1 error rate for Mendelian randomization analysis of knee pain on sleeplessness/insomnia

```
Using specified value of concentration parameter       Using conservative value of concentration parameter
Overlap proportion      Bias    Type 1 error rate       Overlap proportion      Bias    Type 1 error rate
-------------------------------------------------       -------------------------------------------------
0.0                     0.000   0.05                    0.0                     0.000   0.05
0.1                     0.000   0.05                    0.1                     0.000   0.05
0.2                     0.000   0.05                    0.2                     0.000   0.05
0.3                     0.000   0.05                    0.3                     0.000   0.05
0.4                     0.000   0.05                    0.4                     0.000   0.05
0.5                     0.000   0.05                    0.5                     0.000   0.05
0.6                     0.000   0.05                    0.6                     0.000   0.05
0.7                     0.000   0.05                    0.7                     0.000   0.05
0.8                     0.000   0.05                    0.8                     0.000   0.05
0.9                     0.000   0.05                    0.9                     0.000   0.05
1.0                     0.000   0.05                    1.0                     0.000   0.05


Concentration parameter (expected value of F statistic) = 856.86
Conservative value of concentration parameter (lower limit of one-sided 95% confidence interval) = 856.85
```

Bias and type 1 error rate for Mendelian randomization analysis of no pain on sleeplessness/insomnia

```
Using specified value of concentration parameter       Using conservative value of concentration parameter
Overlap proportion      Bias    Type 1 error rate       Overlap proportion      Bias    Type 1 error rate
-------------------------------------------------       -------------------------------------------------
0.0                     0.000   0.05                    0.0                     0.000   0.05
0.1                     0.000   0.05                    0.1                     0.000   0.05
0.2                     0.000   0.05                    0.2                     0.000   0.05
0.3                     0.000   0.05                    0.3                     0.000   0.05
0.4                     0.001   0.05                    0.4                     0.001   0.05
0.5                     0.001   0.05                    0.5                     0.001   0.05
0.6                     0.001   0.05                    0.6                     0.001   0.05
0.7                     0.001   0.05                    0.7                     0.001   0.05
0.8                     0.001   0.05                    0.8                     0.001   0.05
0.9                     0.001   0.05                    0.9                     0.001   0.05
1.0                     0.002   0.05                    1.0                     0.002   0.05


Concentration parameter (expected value of F statistic) = 254.73
Conservative value of concentration parameter (lower limit of one-sided 95% confidence interval) = 254.72
```

Bias and type 1 error rate for Mendelian randomization analysis of headache on anxiety/panic attacks

```
Using specified value of concentration parameter       Using conservative value of concentration parameter
Overlap proportion      Bias    Type 1 error rate       Overlap proportion      Bias    Type 1 error rate
-------------------------------------------------       -------------------------------------------------
0.0                     0.000   0.05                    0.0                     0.000   0.05
0.1                     0.000   0.05                    0.1                     0.000   0.05
0.2                     0.000   0.05                    0.2                     0.000   0.05
0.3                     0.001   0.05                    0.3                     0.001   0.05
0.4                     0.001   0.05                    0.4                     0.001   0.05
0.5                     0.001   0.05                    0.5                     0.001   0.05
0.6                     0.001   0.05                    0.6                     0.001   0.05
0.7                     0.001   0.05                    0.7                     0.001   0.05
0.8                     0.002   0.05                    0.8                     0.002   0.05
0.9                     0.002   0.05                    0.9                     0.002   0.05
1.0                     0.002   0.05                    1.0                     0.002   0.05


Concentration parameter (expected value of F statistic) = 209.44
Conservative value of concentration parameter (lower limit of one-sided 95% confidence interval) = 209.43
```

Bias and type 1 error rate for Mendelian randomization analysis of facial pain on anxiety/panic attacks

```
Using specified value of concentration parameter        Using conservative value of concentration parameter
Overlap proportion      Bias    Type 1 error rate       Overlap proportion      Bias    Type 1 error rate
------------------------------------------------        ------------------------------------------------
0.0                     0.000   0.05                    0.0                     0.000   0.05
0.1                     0.000   0.05                    0.1                     0.000   0.05
0.2                     0.000   0.05                    0.2                     0.000   0.05
0.3                     0.000   0.05                    0.3                     0.000   0.05
0.4                     0.000   0.05                    0.4                     0.000   0.05
0.5                     0.000   0.05                    0.5                     0.000   0.05
0.6                     0.000   0.05                    0.6                     0.000   0.05
0.7                     0.000   0.05                    0.7                     0.000   0.05
0.8                     0.001   0.05                    0.8                     0.001   0.05
0.9                     0.001   0.05                    0.9                     0.001   0.05
1.0                     0.001   0.05                    1.0                     0.001   0.05


Concentration parameter (expected value of F statistic) = 589.08
Conservative value of concentration parameter (lower limit of one-sided 95% confidence interval) = 589.08
```

Bias and type 1 error rate for Mendelian randomization analysis of neck/shoulder pain on anxiety/panic attacks

```
Using specified value of concentration parameter        Using conservative value of concentration parameter
Overlap proportion      Bias    Type 1 error rate       Overlap proportion      Bias    Type 1 error rate
------------------------------------------------        ------------------------------------------------
0.0                     0.000   0.05                    0.0                     0.000   0.05
0.1                     0.000   0.05                    0.1                     0.000   0.05
0.2                     0.000   0.05                    0.2                     0.000   0.05
0.3                     0.000   0.05                    0.3                     0.000   0.05
0.4                     0.000   0.05                    0.4                     0.000   0.05
0.5                     0.000   0.05                    0.5                     0.000   0.05
0.6                     0.000   0.05                    0.6                     0.000   0.05
0.7                     0.000   0.05                    0.7                     0.000   0.05
0.8                     0.000   0.05                    0.8                     0.000   0.05
0.9                     0.000   0.05                    0.9                     0.000   0.05
1.0                     0.000   0.05                    1.0                     0.000   0.05


Concentration parameter (expected value of F statistic) = 1570.92
Conservative value of concentration parameter (lower limit of one-sided 95% confidence interval) = 1570.91
```

Bias and type 1 error rate for Mendelian randomization analysis of back pain on anxiety/panic attacks

```
Using specified value of concentration parameter        Using conservative value of concentration parameter
Overlap proportion      Bias    Type 1 error rate       Overlap proportion      Bias    Type 1 error rate
------------------------------------------------        ------------------------------------------------
0.0                     0.000   0.05                    0.0                     0.000   0.05
0.1                     0.000   0.05                    0.1                     0.000   0.05
0.2                     0.000   0.05                    0.2                     0.000   0.05
0.3                     0.000   0.05                    0.3                     0.000   0.05
0.4                     0.000   0.05                    0.4                     0.000   0.05
0.5                     0.000   0.05                    0.5                     0.000   0.05
0.6                     0.001   0.05                    0.6                     0.001   0.05
0.7                     0.001   0.05                    0.7                     0.001   0.05
0.8                     0.001   0.05                    0.8                     0.001   0.05
0.9                     0.001   0.05                    0.9                     0.001   0.05
1.0                     0.001   0.05                    1.0                     0.001   0.05


Concentration parameter (expected value of F statistic) = 471.26
Conservative value of concentration parameter (lower limit of one-sided 95% confidence interval) = 471.26
```

Bias and type 1 error rate for Mendelian randomization analysis of stomach/abdominal pain on anxiety/panic attacks

Using specified value of concentration parameter			Using conservative value of concentration parameter		
Overlap proportion	Bias	Type 1 error rate	Overlap proportion	Bias	Type 1 error rate
0.0	0.000	0.05	0.0	0.000	0.05
0.1	0.000	0.05	0.1	0.000	0.05
0.2	0.000	0.05	0.2	0.000	0.05
0.3	0.000	0.05	0.3	0.000	0.05
0.4	0.001	0.05	0.4	0.001	0.05
0.5	0.001	0.05	0.5	0.001	0.05
0.6	0.001	0.05	0.6	0.001	0.05
0.7	0.001	0.05	0.7	0.001	0.05
0.8	0.001	0.05	0.8	0.001	0.05
0.9	0.001	0.05	0.9	0.001	0.05
1.0	0.001	0.05	1.0	0.001	0.05

Concentration parameter (expected value of F statistic) = 314.17
Conservative value of concentration parameter (lower limit of one-sided 95% confidence interval) = 314.16

Bias and type 1 error rate for Mendelian randomization analysis of hip pain on anxiety/panic attacks

Using specified value of concentration parameter			Using conservative value of concentration parameter		
Overlap proportion	Bias	Type 1 error rate	Overlap proportion	Bias	Type 1 error rate
0.0	0.000	0.05	0.0	0.000	0.05
0.1	0.000	0.05	0.1	0.000	0.05
0.2	0.000	0.05	0.2	0.000	0.05
0.3	0.000	0.05	0.3	0.000	0.05
0.4	0.000	0.05	0.4	0.000	0.05
0.5	0.000	0.05	0.5	0.000	0.05
0.6	0.000	0.05	0.6	0.000	0.05
0.7	0.000	0.05	0.7	0.000	0.05
0.8	0.000	0.05	0.8	0.000	0.05
0.9	0.000	0.05	0.9	0.000	0.05
1.0	0.000	0.05	1.0	0.000	0.05

Concentration parameter (expected value of F statistic) = 2356.39
Conservative value of concentration parameter (lower limit of one-sided 95% confidence interval) = 2356.38

Bias and type 1 error rate for Mendelian randomization analysis of knee pain on anxiety/panic attacks

Using specified value of concentration parameter			Using conservative value of concentration parameter		
Overlap proportion	Bias	Type 1 error rate	Overlap proportion	Bias	Type 1 error rate
0.0	0.000	0.05	0.0	0.000	0.05
0.1	0.000	0.05	0.1	0.000	0.05
0.2	0.000	0.05	0.2	0.000	0.05
0.3	0.000	0.05	0.3	0.000	0.05
0.4	0.000	0.05	0.4	0.000	0.05
0.5	0.000	0.05	0.5	0.000	0.05
0.6	0.000	0.05	0.6	0.000	0.05
0.7	0.000	0.05	0.7	0.000	0.05
0.8	0.000	0.05	0.8	0.000	0.05
0.9	0.000	0.05	0.9	0.000	0.05
1.0	0.000	0.05	1.0	0.000	0.05

Concentration parameter (expected value of F statistic) = 856.86
Conservative value of concentration parameter (lower limit of one-sided 95% confidence interval) = 856.85

Bias and type 1 error rate for Mendelian randomization analysis of no pain on anxiety/panic attacks

Using specified value of concentration parameter			Using conservative value of concentration parameter		
Overlap proportion	Bias	Type 1 error rate	Overlap proportion	Bias	Type 1 error rate
0.0	0.000	0.05	0.0	0.000	0.05
0.1	0.000	0.05	0.1	0.000	0.05
0.2	0.000	0.05	0.2	0.000	0.05
0.3	0.000	0.05	0.3	0.000	0.05
0.4	0.001	0.05	0.4	0.001	0.05
0.5	0.001	0.05	0.5	0.001	0.05
0.6	0.001	0.05	0.6	0.001	0.05
0.7	0.001	0.05	0.7	0.001	0.05
0.8	0.001	0.05	0.8	0.001	0.05
0.9	0.001	0.05	0.9	0.001	0.05
1.0	0.002	0.05	1.0	0.002	0.05

Concentration parameter (expected value of F statistic) = 254.73
Conservative value of concentration parameter (lower limit of one-sided 95% confidence interval) = 254.72

Bias and type 1 error rate for Mendelian randomization analysis of headache on depression

Using specified value of concentration parameter			Using conservative value of concentration parameter		
Overlap proportion	Bias	Type 1 error rate	Overlap proportion	Bias	Type 1 error rate
0.0	0.000	0.05	0.0	0.000	0.05
0.1	0.000	0.05	0.1	0.000	0.05
0.2	0.000	0.05	0.2	0.000	0.05
0.3	0.001	0.05	0.3	0.001	0.05
0.4	0.001	0.05	0.4	0.001	0.05
0.5	0.001	0.05	0.5	0.001	0.05
0.6	0.001	0.05	0.6	0.001	0.05
0.7	0.001	0.05	0.7	0.001	0.05
0.8	0.002	0.05	0.8	0.002	0.05
0.9	0.002	0.05	0.9	0.002	0.05
1.0	0.002	0.05	1.0	0.002	0.05

Concentration parameter (expected value of F statistic) = 209.44
Conservative value of concentration parameter (lower limit of one-sided 95% confidence interval) = 209.43

Bias and type 1 error rate for Mendelian randomization analysis of facial pain on depression

Using specified value of concentration parameter			Using conservative value of concentration parameter		
Overlap proportion	Bias	Type 1 error rate	Overlap proportion	Bias	Type 1 error rate
0.0	0.000	0.05	0.0	0.000	0.05
0.1	0.000	0.05	0.1	0.000	0.05
0.2	0.000	0.05	0.2	0.000	0.05
0.3	0.000	0.05	0.3	0.000	0.05
0.4	0.000	0.05	0.4	0.000	0.05
0.5	0.000	0.05	0.5	0.000	0.05
0.6	0.000	0.05	0.6	0.000	0.05
0.7	0.000	0.05	0.7	0.000	0.05
0.8	0.001	0.05	0.8	0.001	0.05
0.9	0.001	0.05	0.9	0.001	0.05
1.0	0.001	0.05	1.0	0.001	0.05

Concentration parameter (expected value of F statistic) = 589.08
Conservative value of concentration parameter (lower limit of one-sided 95% confidence interval) = 589.08

Bias and type 1 error rate for Mendelian randomization analysis of neck/shoulder pain on depression

Using specified value of concentration parameter

Overlap proportion	Bias	Type 1 error rate
0.0	0.000	0.05
0.1	0.000	0.05
0.2	0.000	0.05
0.3	0.000	0.05
0.4	0.000	0.05
0.5	0.000	0.05
0.6	0.000	0.05
0.7	0.000	0.05
0.8	0.000	0.05
0.9	0.000	0.05
1.0	0.000	0.05

Using conservative value of concentration parameter

Overlap proportion	Bias	Type 1 error rate
0.0	0.000	0.05
0.1	0.000	0.05
0.2	0.000	0.05
0.3	0.000	0.05
0.4	0.000	0.05
0.5	0.000	0.05
0.6	0.000	0.05
0.7	0.000	0.05
0.8	0.000	0.05
0.9	0.000	0.05
1.0	0.000	0.05

Concentration parameter (expected value of F statistic) = 1570.92
Conservative value of concentration parameter (lower limit of one-sided 95% confidence interval) = 1570.91

Bias and type 1 error rate for Mendelian randomization analysis of back pain on depression

Using specified value of concentration parameter

Overlap proportion	Bias	Type 1 error rate
0.0	0.000	0.05
0.1	0.000	0.05
0.2	0.000	0.05
0.3	0.000	0.05
0.4	0.000	0.05
0.5	0.000	0.05
0.6	0.000	0.05
0.7	0.001	0.05
0.8	0.001	0.05
0.9	0.001	0.05
1.0	0.001	0.05

Using conservative value of concentration parameter

Overlap proportion	Bias	Type 1 error rate
0.0	0.000	0.05
0.1	0.000	0.05
0.2	0.000	0.05
0.3	0.000	0.05
0.4	0.000	0.05
0.5	0.000	0.05
0.6	0.000	0.05
0.7	0.001	0.05
0.8	0.001	0.05
0.9	0.001	0.05
1.0	0.001	0.05

Concentration parameter (expected value of F statistic) = 496.07
Conservative value of concentration parameter (lower limit of one-sided 95% confidence interval) = 496.06

Bias and type 1 error rate for Mendelian randomization analysis of stomach/abdominal pain on depression

Using specified value of concentration parameter

Overlap proportion	Bias	Type 1 error rate
0.0	0.000	0.05
0.1	0.000	0.05
0.2	0.000	0.05
0.3	0.000	0.05
0.4	0.000	0.05
0.5	0.001	0.05
0.6	0.001	0.05
0.7	0.001	0.05
0.8	0.001	0.05
0.9	0.001	0.05
1.0	0.001	0.05

Using conservative value of concentration parameter

Overlap proportion	Bias	Type 1 error rate
0.0	0.000	0.05
0.1	0.000	0.05
0.2	0.000	0.05
0.3	0.000	0.05
0.4	0.000	0.05
0.5	0.001	0.05
0.6	0.001	0.05
0.7	0.001	0.05
0.8	0.001	0.05
0.9	0.001	0.05
1.0	0.001	0.05

Concentration parameter (expected value of F statistic) = 336.61
Conservative value of concentration parameter (lower limit of one-sided 95% confidence interval) = 336.61

Bias and type 1 error rate for Mendelian randomization analysis of hip pain on depression

	Using specified value of concentration parameter		Using conservative value of concentration parameter	
Overlap proportion	Bias	Type 1 error rate	Bias	Type 1 error rate
0.0	0.000	0.05	0.000	0.05
0.1	0.000	0.05	0.000	0.05
0.2	0.000	0.05	0.000	0.05
0.3	0.000	0.05	0.000	0.05
0.4	0.000	0.05	0.000	0.05
0.5	0.000	0.05	0.000	0.05
0.6	0.000	0.05	0.000	0.05
0.7	0.000	0.05	0.000	0.05
0.8	0.000	0.05	0.000	0.05
0.9	0.000	0.05	0.000	0.05
1.0	0.000	0.05	0.000	0.05

Concentration parameter (expected value of F statistic) = 2356.39
Conservative value of concentration parameter (lower limit of one-sided 95% confidence interval) = 2356.38

Bias and type 1 error rate for Mendelian randomization analysis of knee pain on depression

	Using specified value of concentration parameter		Using conservative value of concentration parameter	
Overlap proportion	Bias	Type 1 error rate	Bias	Type 1 error rate
0.0	0.000	0.05	0.000	0.05
0.1	0.000	0.05	0.000	0.05
0.2	0.000	0.05	0.000	0.05
0.3	0.000	0.05	0.000	0.05
0.4	0.000	0.05	0.000	0.05
0.5	0.000	0.05	0.000	0.05
0.6	0.000	0.05	0.000	0.05
0.7	0.000	0.05	0.000	0.05
0.8	0.000	0.05	0.000	0.05
0.9	0.000	0.05	0.000	0.05
1.0	0.000	0.05	0.000	0.05

Concentration parameter (expected value of F statistic) = 856.86
Conservative value of concentration parameter (lower limit of one-sided 95% confidence interval) = 856.85

Bias and type 1 error rate for Mendelian randomization analysis of no pain on depression

	Using specified value of concentration parameter		Using conservative value of concentration parameter	
Overlap proportion	Bias	Type 1 error rate	Bias	Type 1 error rate
0.0	0.000	0.05	0.000	0.05
0.1	0.000	0.05	0.000	0.05
0.2	0.000	0.05	0.000	0.05
0.3	0.000	0.05	0.000	0.05
0.4	0.001	0.05	0.001	0.05
0.5	0.001	0.05	0.001	0.05
0.6	0.001	0.05	0.001	0.05
0.7	0.001	0.05	0.001	0.05
0.8	0.001	0.05	0.001	0.05
0.9	0.001	0.05	0.001	0.05
1.0	0.002	0.05	0.002	0.05

Concentration parameter (expected value of F statistic) = 254.73
Conservative value of concentration parameter (lower limit of one-sided 95% confidence interval) = 254.72

Bias and type 1 error rate for Mendelian randomization analysis of sleeplessness/insomnia on headache

Using specified value of concentration parameter			Using conservative value of concentration parameter		
Overlap proportion	Bias	Type 1 error rate	Overlap proportion	Bias	Type 1 error rate
0.0	0.000	0.05	0.0	0.000	0.05
0.1	0.000	0.05	0.1	0.000	0.05
0.2	0.000	0.05	0.2	0.000	0.05
0.3	0.000	0.05	0.3	0.000	0.05
0.4	0.001	0.05	0.4	0.001	0.05
0.5	0.001	0.05	0.5	0.001	0.05
0.6	0.001	0.05	0.6	0.001	0.05
0.7	0.001	0.05	0.7	0.001	0.05
0.8	0.001	0.05	0.8	0.001	0.05
0.9	0.001	0.05	0.9	0.001	0.05
1.0	0.002	0.05	1.0	0.002	0.05

Bias and type 1 error rate for Mendelian randomization analysis of anxiety/panic attacks on headache

Using specified value of concentration parameter			Using conservative value of concentration parameter		
Overlap proportion	Bias	Type 1 error rate	Overlap proportion	Bias	Type 1 error rate
0.0	0.000	0.05	0.0	0.000	0.05
0.1	0.000	0.05	0.1	0.000	0.05
0.2	0.000	0.05	0.2	0.000	0.05
0.3	0.000	0.05	0.3	0.000	0.05
0.4	0.001	0.05	0.4	0.001	0.05
0.5	0.001	0.05	0.5	0.001	0.05
0.6	0.001	0.05	0.6	0.001	0.05
0.7	0.001	0.05	0.7	0.001	0.05
0.8	0.001	0.05	0.8	0.001	0.05
0.9	0.001	0.05	0.9	0.001	0.05
1.0	0.002	0.05	1.0	0.002	0.05

Concentration parameter (expected value of F statistic) = 254.82
Conservative value of concentration parameter (lower limit of one-sided 95% confidence interval) = 254.82

Bias and type 1 error rate for Mendelian randomization analysis of depression on headache

Using specified value of concentration parameter			Using conservative value of concentration parameter		
Overlap proportion	Bias	Type 1 error rate	Overlap proportion	Bias	Type 1 error rate
0.0	0.000	0.05	0.0	0.000	0.05
0.1	0.000	0.05	0.1	0.000	0.05
0.2	0.000	0.05	0.2	0.000	0.05
0.3	0.000	0.05	0.3	0.000	0.05
0.4	0.001	0.05	0.4	0.001	0.05
0.5	0.001	0.05	0.5	0.001	0.05
0.6	0.001	0.05	0.6	0.001	0.05
0.7	0.001	0.05	0.7	0.001	0.05
0.8	0.001	0.05	0.8	0.001	0.05
0.9	0.001	0.05	0.9	0.001	0.05
1.0	0.001	0.05	1.0	0.001	0.05

Concentration parameter (expected value of F statistic) = 295.22
Conservative value of concentration parameter (lower limit of one-sided 95% confidence interval) = 295.21

Bias and type 1 error rate for Mendelian randomization analysis of sleeplessness/insomnia on facial pain

Using specified value of concentration parameter			Using conservative value of concentration parameter		
Overlap proportion	Bias	Type 1 error rate	Overlap proportion	Bias	Type 1 error rate
0.0	0.000	0.05	0.0	0.000	0.05
0.1	0.000	0.05	0.1	0.000	0.05
0.2	0.000	0.05	0.2	0.000	0.05
0.3	0.000	0.05	0.3	0.000	0.05
0.4	0.001	0.05	0.4	0.001	0.05
0.5	0.001	0.05	0.5	0.001	0.05
0.6	0.001	0.05	0.6	0.001	0.05
0.7	0.001	0.05	0.7	0.001	0.05
0.8	0.001	0.05	0.8	0.001	0.05
0.9	0.001	0.05	0.9	0.001	0.05
1.0	0.002	0.05	1.0	0.002	0.05

Concentration parameter (expected value of F statistic) = 264.47
Conservative value of concentration parameter (lower limit of one-sided 95% confidence interval) = 264.47

Bias and type 1 error rate for Mendelian randomization analysis of depression on facial pain

Using specified value of concentration parameter			Using conservative value of concentration parameter		
Overlap proportion	Bias	Type 1 error rate	Overlap proportion	Bias	Type 1 error rate
0.0	0.000	0.05	0.0	0.000	0.05
0.1	0.000	0.05	0.1	0.000	0.05
0.2	0.000	0.05	0.2	0.000	0.05
0.3	0.000	0.05	0.3	0.000	0.05
0.4	0.000	0.05	0.4	0.000	0.05
0.5	0.001	0.05	0.5	0.001	0.05
0.6	0.001	0.05	0.6	0.001	0.05
0.7	0.001	0.05	0.7	0.001	0.05
0.8	0.001	0.05	0.8	0.001	0.05
0.9	0.001	0.05	0.9	0.001	0.05
1.0	0.001	0.05	1.0	0.001	0.05

Concentration parameter (expected value of F statistic) = 377.88
Conservative value of concentration parameter (lower limit of one-sided 95% confidence interval) = 377.88

Bias and type 1 error rate for Mendelian randomization analysis of anxiety/panic attacks on facial pain

Using specified value of concentration parameter			Using conservative value of concentration parameter		
Overlap proportion	Bias	Type 1 error rate	Overlap proportion	Bias	Type 1 error rate
0.0	0.000	0.05	0.0	0.000	0.05
0.1	0.000	0.05	0.1	0.000	0.05
0.2	0.000	0.05	0.2	0.000	0.05
0.3	0.000	0.05	0.3	0.000	0.05
0.4	0.000	0.05	0.4	0.000	0.05
0.5	0.000	0.05	0.5	0.000	0.05
0.6	0.000	0.05	0.6	0.000	0.05
0.7	0.000	0.05	0.7	0.000	0.05
0.8	0.000	0.05	0.8	0.000	0.05
0.9	0.000	0.05	0.9	0.000	0.05
1.0	0.000	0.05	1.0	0.000	0.05

Concentration parameter (expected value of F statistic) = 860.08
Conservative value of concentration parameter (lower limit of one-sided 95% confidence interval) = 860.07

Bias and type 1 error rate for Mendelian randomization analysis of sleeplessness/insomnia on neck/shoulder pain

Using specified value of concentration parameter			Using conservative value of concentration parameter		
Overlap proportion	Bias	Type 1 error rate	Overlap proportion	Bias	Type 1 error rate
0.0	0.000	0.05	0.0	0.000	0.05
0.1	0.000	0.05	0.1	0.000	0.05
0.2	0.000	0.05	0.2	0.000	0.05
0.3	0.000	0.05	0.3	0.000	0.05
0.4	0.001	0.05	0.4	0.001	0.05
0.5	0.001	0.05	0.5	0.001	0.05
0.6	0.001	0.05	0.6	0.001	0.05
0.7	0.001	0.05	0.7	0.001	0.05
0.8	0.001	0.05	0.8	0.001	0.05
0.9	0.001	0.05	0.9	0.001	0.05
1.0	0.002	0.05	1.0	0.002	0.05

Concentration parameter (expected value of F statistic) = 254.68
Conservative value of concentration parameter (lower limit of one-sided 95% confidence interval) = 254.67

Bias and type 1 error rate for Mendelian randomization analysis of anxiety/panic attacks on neck/shoulder pain

Using specified value of concentration parameter			Using conservative value of concentration parameter		
Overlap proportion	Bias	Type 1 error rate	Overlap proportion	Bias	Type 1 error rate
0.0	0.000	0.05	0.0	0.000	0.05
0.1	0.000	0.05	0.1	0.000	0.05
0.2	0.000	0.05	0.2	0.000	0.05
0.3	0.000	0.05	0.3	0.000	0.05
0.4	0.000	0.05	0.4	0.000	0.05
0.5	0.000	0.05	0.5	0.000	0.05
0.6	0.000	0.05	0.6	0.000	0.05
0.7	0.000	0.05	0.7	0.000	0.05
0.8	0.001	0.05	0.8	0.001	0.05
0.9	0.001	0.05	0.9	0.001	0.05
1.0	0.001	0.05	1.0	0.001	0.05

Concentration parameter (expected value of F statistic) = 573.38
Conservative value of concentration parameter (lower limit of one-sided 95% confidence interval) = 573.37

Bias and type 1 error rate for Mendelian randomization analysis of depression on neck/shoulder pain

Using specified value of concentration parameter			Using conservative value of concentration parameter		
Overlap proportion	Bias	Type 1 error rate	Overlap proportion	Bias	Type 1 error rate
0.0	0.000	0.05	0.0	0.000	0.05
0.1	0.000	0.05	0.1	0.000	0.05
0.2	0.000	0.05	0.2	0.000	0.05
0.3	0.000	0.05	0.3	0.000	0.05
0.4	0.001	0.05	0.4	0.001	0.05
0.5	0.001	0.05	0.5	0.001	0.05
0.6	0.001	0.05	0.6	0.001	0.05
0.7	0.001	0.05	0.7	0.001	0.05
0.8	0.001	0.05	0.8	0.001	0.05
0.9	0.001	0.05	0.9	0.001	0.05
1.0	0.001	0.05	1.0	0.001	0.05

Concentration parameter (expected value of F statistic) = 295.22
Conservative value of concentration parameter (lower limit of one-sided 95% confidence interval) = 295.21

Bias and type 1 error rate for Mendelian randomization analysis of sleeplessness/insomnia on back pain

Using specified value of concentration parameter

Overlap proportion	Bias	Type 1 error rate
0.0	0.000	0.05
0.1	0.000	0.05
0.2	0.000	0.05
0.3	0.000	0.05
0.4	0.001	0.05
0.5	0.001	0.05
0.6	0.001	0.05
0.7	0.001	0.05
0.8	0.001	0.05
0.9	0.001	0.05
1.0	0.002	0.05

Using conservative value of concentration parameter

Overlap proportion	Bias	Type 1 error rate
0.0	0.000	0.05
0.1	0.000	0.05
0.2	0.000	0.05
0.3	0.000	0.05
0.4	0.001	0.05
0.5	0.001	0.05
0.6	0.001	0.05
0.7	0.001	0.05
0.8	0.001	0.05
0.9	0.001	0.05
1.0	0.002	0.05

Concentration parameter (expected value of F statistic) = 254.68
Conservative value of concentration parameter (lower limit of one-sided 95% confidence interval) = 254.67

Bias and type 1 error rate for Mendelian randomization analysis of anxiety/panic attacks on back pain

Using specified value of concentration parameter

Overlap proportion	Bias	Type 1 error rate
0.0	0.000	0.05
0.1	0.000	0.05
0.2	0.000	0.05
0.3	0.000	0.05
0.4	0.000	0.05
0.5	0.000	0.05
0.6	0.000	0.05
0.7	0.000	0.05
0.8	0.001	0.05
0.9	0.001	0.05
1.0	0.001	0.05

Using conservative value of concentration parameter

Overlap proportion	Bias	Type 1 error rate
0.0	0.000	0.05
0.1	0.000	0.05
0.2	0.000	0.05
0.3	0.000	0.05
0.4	0.000	0.05
0.5	0.000	0.05
0.6	0.000	0.05
0.7	0.000	0.05
0.8	0.001	0.05
0.9	0.001	0.05
1.0	0.001	0.05

Concentration parameter (expected value of F statistic) = 573.38
Conservative value of concentration parameter (lower limit of one-sided 95% confidence interval) = 573.37

Bias and type 1 error rate for Mendelian randomization analysis of depression on back pain

Using specified value of concentration parameter

Overlap proportion	Bias	Type 1 error rate
0.0	0.000	0.05
0.1	0.000	0.05
0.2	0.000	0.05
0.3	0.000	0.05
0.4	0.001	0.05
0.5	0.001	0.05
0.6	0.001	0.05
0.7	0.001	0.05
0.8	0.001	0.05
0.9	0.001	0.05
1.0	0.001	0.05

Using conservative value of concentration parameter

Overlap proportion	Bias	Type 1 error rate
0.0	0.000	0.05
0.1	0.000	0.05
0.2	0.000	0.05
0.3	0.000	0.05
0.4	0.001	0.05
0.5	0.001	0.05
0.6	0.001	0.05
0.7	0.001	0.05
0.8	0.001	0.05
0.9	0.001	0.05
1.0	0.001	0.05

Concentration parameter (expected value of F statistic) = 295.22
Conservative value of concentration parameter (lower limit of one-sided 95% confidence interval) = 295.21

Bias and type 1 error rate for Mendelian randomization analysis of sleeplessness/insomnia on stomach/abdominal pain

Using specified value of concentration parameter			Using conservative value of concentration parameter		
Overlap proportion	Bias	Type 1 error rate	Overlap proportion	Bias	Type 1 error rate
0.0	0.000	0.05	0.0	0.000	0.05
0.1	0.000	0.05	0.1	0.000	0.05
0.2	0.000	0.05	0.2	0.000	0.05
0.3	0.000	0.05	0.3	0.000	0.05
0.4	0.001	0.05	0.4	0.001	0.05
0.5	0.001	0.05	0.5	0.001	0.05
0.6	0.001	0.05	0.6	0.001	0.05
0.7	0.001	0.05	0.7	0.001	0.05
0.8	0.001	0.05	0.8	0.001	0.05
0.9	0.001	0.05	0.9	0.001	0.05
1.0	0.002	0.05	1.0	0.002	0.05

Concentration parameter (expected value of F statistic) = 254.68
Conservative value of concentration parameter (lower limit of one-sided 95% confidence interval) = 254.67

Bias and type 1 error rate for Mendelian randomization analysis of anxiety/panic attacks on stomach/abdominal pain

Using specified value of concentration parameter			Using conservative value of concentration parameter		
Overlap proportion	Bias	Type 1 error rate	Overlap proportion	Bias	Type 1 error rate
0.0	0.000	0.05	0.0	0.000	0.05
0.1	0.000	0.05	0.1	0.000	0.05
0.2	0.000	0.05	0.2	0.000	0.05
0.3	0.000	0.05	0.3	0.000	0.05
0.4	0.001	0.05	0.4	0.001	0.05
0.5	0.001	0.05	0.5	0.001	0.05
0.6	0.001	0.05	0.6	0.001	0.05
0.7	0.001	0.05	0.7	0.001	0.05
0.8	0.001	0.05	0.8	0.001	0.05
0.9	0.001	0.05	0.9	0.001	0.05
1.0	0.002	0.05	1.0	0.002	0.05

Concentration parameter (expected value of F statistic) = 254.68
Conservative value of concentration parameter (lower limit of one-sided 95% confidence interval) = 254.67

Bias and type 1 error rate for Mendelian randomization analysis of depression on stomach/abdominal pain

Using specified value of concentration parameter			Using conservative value of concentration parameter		
Overlap proportion	Bias	Type 1 error rate	Overlap proportion	Bias	Type 1 error rate
0.0	0.000	0.05	0.0	0.000	0.05
0.1	0.000	0.05	0.1	0.000	0.05
0.2	0.000	0.05	0.2	0.000	0.05
0.3	0.000	0.05	0.3	0.000	0.05
0.4	0.001	0.05	0.4	0.001	0.05
0.5	0.001	0.05	0.5	0.001	0.05
0.6	0.001	0.05	0.6	0.001	0.05
0.7	0.001	0.05	0.7	0.001	0.05
0.8	0.001	0.05	0.8	0.001	0.05
0.9	0.001	0.05	0.9	0.001	0.05
1.0	0.001	0.05	1.0	0.001	0.05

Concentration parameter (expected value of F statistic) = 295.22
Conservative value of concentration parameter (lower limit of one-sided 95% confidence interval) = 295.21

Bias and type 1 error rate for Mendelian randomization analysis of sleeplessness/insomnia on hip pain

Using specified value of concentration parameter			Using conservative value of concentration parameter		
Overlap proportion	Bias	Type 1 error rate	Overlap proportion	Bias	Type 1 error rate
0.0	0.000	0.05	0.0	0.000	0.05
0.1	0.000	0.05	0.1	0.000	0.05
0.2	0.000	0.05	0.2	0.000	0.05
0.3	0.000	0.05	0.3	0.000	0.05
0.4	0.001	0.05	0.4	0.001	0.05
0.5	0.001	0.05	0.5	0.001	0.05
0.6	0.001	0.05	0.6	0.001	0.05
0.7	0.001	0.05	0.7	0.001	0.05
0.8	0.001	0.05	0.8	0.001	0.05
0.9	0.001	0.05	0.9	0.001	0.05
1.0	0.002	0.05	1.0	0.002	0.05

Concentration parameter (expected value of F statistic) = 254.68
Conservative value of concentration parameter (lower limit of one-sided 95% confidence interval) = 254.67

Bias and type 1 error rate for Mendelian randomization analysis of anxiety/panic attacks on hip pain

Using specified value of concentration parameter			Using conservative value of concentration parameter		
Overlap proportion	Bias	Type 1 error rate	Overlap proportion	Bias	Type 1 error rate
0.0	0.000	0.05	0.0	0.000	0.05
0.1	0.000	0.05	0.1	0.000	0.05
0.2	0.000	0.05	0.2	0.000	0.05
0.3	0.000	0.05	0.3	0.000	0.05
0.4	0.000	0.05	0.4	0.000	0.05
0.5	0.000	0.05	0.5	0.000	0.05
0.6	0.000	0.05	0.6	0.000	0.05
0.7	0.000	0.05	0.7	0.000	0.05
0.8	0.001	0.05	0.8	0.001	0.05
0.9	0.001	0.05	0.9	0.001	0.05
1.0	0.001	0.05	1.0	0.001	0.05

Concentration parameter (expected value of F statistic) = 573.38
Conservative value of concentration parameter (lower limit of one-sided 95% confidence interval) = 573.37

Bias and type 1 error rate for Mendelian randomization analysis of depression on hip pain

Using specified value of concentration parameter			Using conservative value of concentration parameter		
Overlap proportion	Bias	Type 1 error rate	Overlap proportion	Bias	Type 1 error rate
0.0	0.000	0.05	0.0	0.000	0.05
0.1	0.000	0.05	0.1	0.000	0.05
0.2	0.000	0.05	0.2	0.000	0.05
0.3	0.000	0.05	0.3	0.000	0.05
0.4	0.001	0.05	0.4	0.001	0.05
0.5	0.001	0.05	0.5	0.001	0.05
0.6	0.001	0.05	0.6	0.001	0.05
0.7	0.001	0.05	0.7	0.001	0.05
0.8	0.001	0.05	0.8	0.001	0.05
0.9	0.001	0.05	0.9	0.001	0.05
1.0	0.001	0.05	1.0	0.001	0.05

Concentration parameter (expected value of F statistic) = 295.22
Conservative value of concentration parameter (lower limit of one-sided 95% confidence interval) = 295.21

Bias and type 1 error rate for Mendelian randomization analysis of sleeplessness/insomnia on knee pain

Using specified value of concentration parameter			Using conservative value of concentration parameter		
Overlap proportion	Bias	Type 1 error rate	Overlap proportion	Bias	Type 1 error rate
0.0	0.000	0.05	0.0	0.000	0.05
0.1	0.000	0.05	0.1	0.000	0.05
0.2	0.000	0.05	0.2	0.000	0.05
0.3	0.000	0.05	0.3	0.000	0.05
0.4	0.001	0.05	0.4	0.001	0.05
0.5	0.001	0.05	0.5	0.001	0.05
0.6	0.001	0.05	0.6	0.001	0.05
0.7	0.001	0.05	0.7	0.001	0.05
0.8	0.001	0.05	0.8	0.001	0.05
0.9	0.001	0.05	0.9	0.001	0.05
1.0	0.002	0.05	1.0	0.002	0.05

Concentration parameter (expected value of F statistic) = 254.68
Conservative value of concentration parameter (lower limit of one-sided 95% confidence interval) = 254.67

Bias and type 1 error rate for Mendelian randomization analysis of anxiety/panic attacks on knee pain

Using specified value of concentration parameter			Using conservative value of concentration parameter		
Overlap proportion	Bias	Type 1 error rate	Overlap proportion	Bias	Type 1 error rate
0.0	0.000	0.05	0.0	0.000	0.05
0.1	0.000	0.05	0.1	0.000	0.05
0.2	0.000	0.05	0.2	0.000	0.05
0.3	0.000	0.05	0.3	0.000	0.05
0.4	0.000	0.05	0.4	0.000	0.05
0.5	0.000	0.05	0.5	0.000	0.05
0.6	0.000	0.05	0.6	0.000	0.05
0.7	0.000	0.05	0.7	0.000	0.05
0.8	0.001	0.05	0.8	0.001	0.05
0.9	0.001	0.05	0.9	0.001	0.05
1.0	0.001	0.05	1.0	0.001	0.05

Concentration parameter (expected value of F statistic) = 573.38
Conservative value of concentration parameter (lower limit of one-sided 95% confidence interval) = 573.37

Bias and type 1 error rate for Mendelian randomization analysis of depression on knee pain

Using specified value of concentration parameter			Using conservative value of concentration parameter		
Overlap proportion	Bias	Type 1 error rate	Overlap proportion	Bias	Type 1 error rate
0.0	0.000	0.05	0.0	0.000	0.05
0.1	0.000	0.05	0.1	0.000	0.05
0.2	0.000	0.05	0.2	0.000	0.05
0.3	0.000	0.05	0.3	0.000	0.05
0.4	0.001	0.05	0.4	0.001	0.05
0.5	0.001	0.05	0.5	0.001	0.05
0.6	0.001	0.05	0.6	0.001	0.05
0.7	0.001	0.05	0.7	0.001	0.05
0.8	0.001	0.05	0.8	0.001	0.05
0.9	0.001	0.05	0.9	0.001	0.05
1.0	0.001	0.05	1.0	0.001	0.05

Concentration parameter (expected value of F statistic) = 295.22
Conservative value of concentration parameter (lower limit of one-sided 95% confidence interval) = 295.21

Bias and type 1 error rate for Mendelian randomization analysis of sleeplessness/insomnia on no pain

Using specified value of concentration parameter			Using conservative value of concentration parameter		
Overlap proportion	Bias	Type 1 error rate	Overlap proportion	Bias	Type 1 error rate
0.0	0.000	0.05	0.0	0.000	0.05
0.1	0.000	0.05	0.1	0.000	0.05
0.2	0.000	0.05	0.2	0.000	0.05
0.3	0.000	0.05	0.3	0.000	0.05
0.4	0.001	0.05	0.4	0.001	0.05
0.5	0.001	0.05	0.5	0.001	0.05
0.6	0.001	0.05	0.6	0.001	0.05
0.7	0.001	0.05	0.7	0.001	0.05
0.8	0.001	0.05	0.8	0.001	0.05
0.9	0.001	0.05	0.9	0.001	0.05
1.0	0.002	0.05	1.0	0.002	0.05

Concentration parameter (expected value of F statistic) = 254.68
Conservative value of concentration parameter (lower limit of one-sided 95% confidence interval) = 254.67

Bias and type 1 error rate for Mendelian randomization analysis of anxiety/panic attacks on no pain

Using specified value of concentration parameter			Using conservative value of concentration parameter		
Overlap proportion	Bias	Type 1 error rate	Overlap proportion	Bias	Type 1 error rate
0.0	0.000	0.05	0.0	0.000	0.05
0.1	0.000	0.05	0.1	0.000	0.05
0.2	0.000	0.05	0.2	0.000	0.05
0.3	0.000	0.05	0.3	0.000	0.05
0.4	0.000	0.05	0.4	0.000	0.05
0.5	0.000	0.05	0.5	0.000	0.05
0.6	0.000	0.05	0.6	0.000	0.05
0.7	0.000	0.05	0.7	0.000	0.05
0.8	0.001	0.05	0.8	0.001	0.05
0.9	0.001	0.05	0.9	0.001	0.05
1.0	0.001	0.05	1.0	0.001	0.05

Concentration parameter (expected value of F statistic) = 573.38
Conservative value of concentration parameter (lower limit of one-sided 95% confidence interval) = 573.37

Bias and type 1 error rate for Mendelian randomization analysis of depression on no pain

Using specified value of concentration parameter			Using conservative value of concentration parameter		
Overlap proportion	Bias	Type 1 error rate	Overlap proportion	Bias	Type 1 error rate
0.0	0.000	0.05	0.0	0.000	0.05
0.1	0.000	0.05	0.1	0.000	0.05
0.2	0.000	0.05	0.2	0.000	0.05
0.3	0.000	0.05	0.3	0.000	0.05
0.4	0.001	0.05	0.4	0.001	0.05
0.5	0.001	0.05	0.5	0.001	0.05
0.6	0.001	0.05	0.6	0.001	0.05
0.7	0.001	0.05	0.7	0.001	0.05
0.8	0.001	0.05	0.8	0.001	0.05
0.9	0.001	0.05	0.9	0.001	0.05
1.0	0.001	0.05	1.0	0.001	0.05

Concentration parameter (expected value of F statistic) = 295.22
Conservative value of concentration parameter (lower limit of one-sided 95% confidence interval) = 295.21
